# Hybridization chain reaction enables a unified approach to multiplexed, quantitative, high-resolution immunohistochemistry and *in situ* hybridization

**DOI:** 10.1242/dev.199847

**Published:** 2021-11-18

**Authors:** Maayan Schwarzkopf, Mike C. Liu, Samuel J. Schulte, Rachel Ives, Naeem Husain, Harry M. T. Choi, Niles A. Pierce

**Affiliations:** 1 Division of Biology and Biological Engineering, California Institute of Technology, Pasadena, CA 91125, USA; 2 Molecular Instruments, Los Angeles, CA 90041, USA; 3 Division of Engineering & Applied Science, California Institute of Technology, Pasadena, CA 91125, USA

**Keywords:** Immunofluorescence (IF), RNA fluorescence *in situ* hybridization (RNA-FISH), qHCR imaging, Formalin-fixed paraffin-embedded (FFPE) mouse brain and human breast tissue sections, Whole-mount zebrafish embryos

## Abstract

RNA *in situ* hybridization based on the mechanism of the hybridization chain reaction (HCR) enables multiplexed, quantitative, high-resolution RNA imaging in highly autofluorescent samples, including whole-mount vertebrate embryos, thick brain slices and formalin-fixed paraffin-embedded tissue sections. Here, we extend the benefits of one-step, multiplexed, quantitative, isothermal, enzyme-free HCR signal amplification to immunohistochemistry, enabling accurate and precise protein relative quantitation with subcellular resolution in an anatomical context. Moreover, we provide a unified framework for simultaneous quantitative protein and RNA imaging with one-step HCR signal amplification performed for all target proteins and RNAs simultaneously.

## Abstract (precis)

**Summary:** Signal amplification based on the mechanism of hybridization chain reaction enables multiplexed, quantitative, high-resolution imaging of protein and RNA targets in highly autofluorescent tissues.

## INTRODUCTION

Biological circuits encoded in the genome of each organism direct development, maintain integrity in the face of attacks, control responses to environmental stimuli and sometimes malfunction to cause disease. RNA *in situ* hybridization (RNA-ISH) methods (Harrison et al., 1973; Tautz and Pfeifle, 1989; Qian et al., 2004) and immunohistochemistry (IHC) methods (Coons et al., 1941; Ramos-Vara, 2005; Kim et al., 2016) provide biologists, drug developers and pathologists with crucial windows into the spatial organization of this circuitry, enabling imaging of RNA and protein expression in an anatomical context. Although it is desirable to perform multiplexed experiments in which a panel of targets are imaged quantitatively at high resolution in a single specimen, using traditional RNA-ISH and IHC methods in highly autofluorescent samples including whole-mount vertebrate embryos and FFPE tissue sections, multiplexing is cumbersome, staining is non-quantitative and spatial resolution is routinely compromised by diffusion of reporter molecules. These multi-decade technological shortcomings are significant impediments to biological research, as well as to the advancement of drug development and pathology assays, hindering high-dimensional, quantitative, high-resolution analyses of developmental and disease-related regulatory networks in an anatomical context.

RNA-ISH methods detect RNA targets using nucleic acid probes and IHC methods detect protein targets using antibody probes. In either case, probes can be directly labeled with reporter molecules (Kislauskis et al., 1993; Femino et al., 1998; Kosman et al., 2004; Chan et al., 2005; Raj et al., 2008), but to increase the signal-to-background ratio, are more often used to mediate signal amplification in the vicinity of the probe (Qian and Lloyd, 2003; Ramos-Vara and Miller, 2014). A variety of *in situ* amplification approaches have been developed, including immunological methods (Macechko et al., 1997; Hughes and Krause, 1998; Kosman et al., 2004), branched DNA methods (Player et al., 2001; Wang et al., 2012; Kishi et al., 2019; Saka et al., 2019), *in situ* PCR methods (Nuovo et al., 1992; Martínez et al., 1995; Wiedorn et al., 1999) and rolling circle amplification methods (Gusev et al., 2001; Zhou et al., 2001; Larsson et al., 2010). However, for both RNA-ISH (Tautz and Pfeifle, 1989; Harland, 1991; Lehmann and Tautz, 1994; Kerstens et al., 1995; Nieto et al., 1996; Thisse et al., 2004; Piette et al., 2008; Thisse and Thisse, 2008; Wang et al., 2012) and IHC (Takakura et al., 1997; Sillitoe and Hawkes, 2002; Ahnfelt-Ronne et al., 2007; Fujisawa et al., 2015; Staudt et al., 2015), traditional *in situ* amplification based on enzyme-mediated catalytic reporter deposition (CARD) remains the dominant approach for achieving high signal-to-background in highly autofluorescent samples, including whole-mount vertebrate embryos and FFPE tissue sections. CARD is widely used despite three significant drawbacks: multiplexing is cumbersome due to the lack of orthogonal deposition chemistries, necessitating serial amplification for one target after another (Denkers et al., 2004; Kosman et al., 2004; Clay and Ramakrishnan, 2005; Barroso-Chinea et al., 2007; Tóth and Mezey, 2007; Glass et al., 2009; Stack et al., 2014; Mitchell et al., 2014; Tsujikawa et al., 2017); staining is qualitative rather than quantitative; and spatial resolution is often compromised by diffusion of reporter molecules before deposition (Tautz and Pfeifle, 1989; Takakura et al., 1997; Sillitoe and Hawkes, 2002; Thisse et al., 2004; Acloque et al., 2008; Weiszmann et al., 2009).

**Fig. 1.**
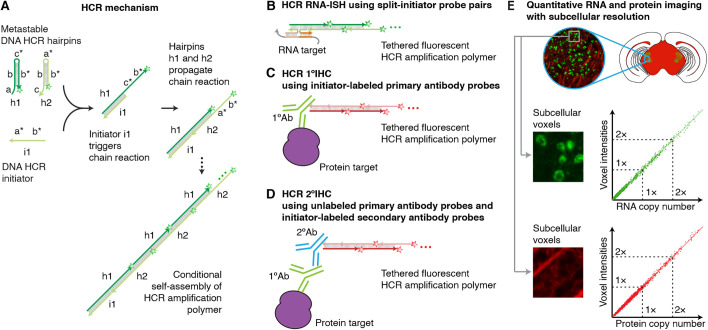
**A unified framework for multiplexed, quantitative, high-resolution protein and RNA imaging using HCR 1°IHC + HCR RNA-ISH or HCR 2°IHC + HCR RNA-ISH** (A) One-step, isothermal, enzyme-free signal amplification via hybridization chain reaction (HCR) (Dirks and Pierce, 2004). Kinetically trapped hairpins h1 and h2 co-exist metastably in solution on lab time scales, storing the energy to drive a conditional self-assembly cascade upon exposure to a cognate initiator sequence i1. Stars indicate fluorophores. (B) HCR RNA-ISH using split-initiator probe pairs that hybridize to adjacent binding sites on the target RNA to colocalize a full HCR initiator and trigger HCR. (C) HCR 1°IHC using initiator-labeled primary antibody probes. (D) HCR 2°IHC using unlabeled primary antibody probes and initiator-labeled secondary antibody probes. (E) Conceptual schematic: HCR signal scales approximately linearly with the abundance of a target RNA (green channel) or protein (red channel), enabling accurate and precise relative quantitation with subcellular resolution in an anatomical context.

In the context of RNA-ISH, *in situ* amplification based on the mechanism of hybridization chain reaction (HCR; Fig. 1A) (Dirks and Pierce, 2004) overcomes the longstanding shortcomings of CARD to enable multiplexed, quantitative, high-resolution imaging of RNA expression in diverse organisms and sample types, including highly autofluorescent samples (Choi et al., 2010, 2014, 2016, 2018; Shah et al., 2016; Trivedi et al., 2018) (e.g. see Table S1). To image RNA expression, targets are detected by nucleic acid probes that trigger isothermal enzyme-free chain reactions in which fluorophore-labeled HCR hairpins self-assemble into tethered fluorescent amplification polymers (Fig. 1B). Orthogonal HCR amplifiers operate independently within the sample so the experimental timeline for multiplexed experiments is independent of the number of target RNAs (Choi et al., 2010, 2014). The amplified HCR signal scales approximately linearly with the number of target molecules (Fig. 1E), enabling accurate and precise RNA relative quantitation with subcellular resolution in the anatomical context of whole-mount vertebrate embryos (Trivedi et al., 2018; Choi et al., 2018). Amplification polymers remain tethered to their initiating probes, enabling imaging of RNA expression with subcellular or single-molecule resolution as desired (Choi et al., 2014, 2016, 2018; Shah et al., 2016).

These properties that make HCR signal amplification well-suited for RNA-ISH appear equally favorable in the context of IHC, suggesting the approach of combining HCR signal amplification with antibody probes (Koos et al., 2015; Husain, 2016; Lin et al., 2018b). Here, we extend the benefits of one-step, quantitative, enzyme-free signal amplification from RNA-ISH to IHC, validating multiplexed, quantitative, high-resolution imaging of protein expression with high signal-to-background in highly autofluorescent samples, thus overcoming the longstanding shortcomings of IHC using CARD. Moreover, we establish a unified framework for simultaneous multiplexed, quantitative, high-resolution IHC and RNA-ISH, with one-step HCR signal amplification performed for all targets simultaneously.

## RESULTS

For protein imaging with HCR we pursue two complementary approaches. Using HCR 1°IHC, protein targets are detected using primary antibody probes labeled with one or more HCR initiators (Fig. 1C). For multiplexed experiments, the probes for different targets are labeled with different HCR initiators that trigger orthogonal HCR amplifiers labeled with spectrally distinct fluorophores. Researchers have the flexibility to detect different targets using primary antibody probes raised in the same host species (or a variety of host species, as convenient). On the other hand, each new initiator-labeled primary antibody probe must be validated, as there is the potential for oligo conjugation to interfere with epitope binding in an antibody- or crosslinker-dependent fashion. Using HCR 2°IHC, protein targets are detected using unlabeled primary antibody probes that are in turn detected by secondary antibody probes labeled with one or more HCR initiators (Fig. 1D). This approach has the advantage that validation of a small library of initiator-labeled secondary antibodies (e.g. five secondaries targeting different host species) enables immediate use of large libraries of primary antibody probes (e.g. 10^5^ commercially available primaries) without modification. On the other hand, for multiplexed experiments, each target must be detected using a primary antibody raised in a different host species to enable subsequent detection by an anti-host secondary antibody probe that triggers an orthogonal spectrally distinct HCR amplifier. Hence, depending on the available antibody probes, one may prefer HCR 1°IHC in one instance and HCR 2°IHC in another.

### Multiplexed protein imaging using HCR 1°IHC or HCR 2°IHC

**Fig. 2.**
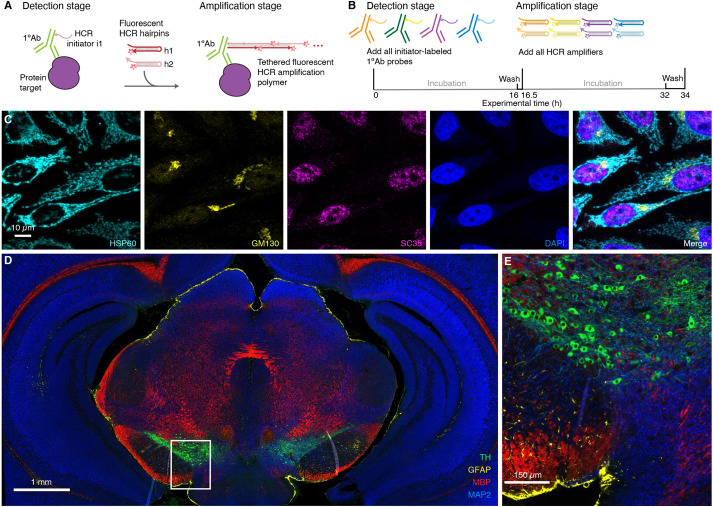
**Multiplexed protein imaging via HCR 1°IHC using initiator-labeled primary antibody probes and simultaneous HCR signal amplification for all targets.** (A) Two-stage HCR 1°IHC protocol. Detection stage: initiator-labeled primary antibody probes bind to protein targets; wash. Amplification stage: initiators trigger self-assembly of fluorophore-labeled HCR hairpins into tethered fluorescent amplification polymers; wash. (B) Multiplexing timeline. The same two-stage protocol is used independent of the number of target proteins. (C) Confocal image of 3-plex protein imaging in mammalian cells on a slide; 0.2×0.2 µm pixels; maximum intensity *z*-projection. Target proteins: HSP60 (Alexa488), GM130 (Alexa647) and SC35 (Alexa546). Sample: HeLa cells. (D) Epifluorescence image of 4-plex protein imaging in FFPE mouse brain sections; 0.3×0.3 µm pixels. Target proteins: TH (Alexa488), GFAP (Alexa546), MBP (Alexa647) and MAP2 (Alexa750). (E) Zoom of indicated region in D. Sample: FFPE C57BL/6 mouse brain section (coronal); 5 µm thickness. See section S5.2 of the supplementary information for additional data.

**Fig. 3.**
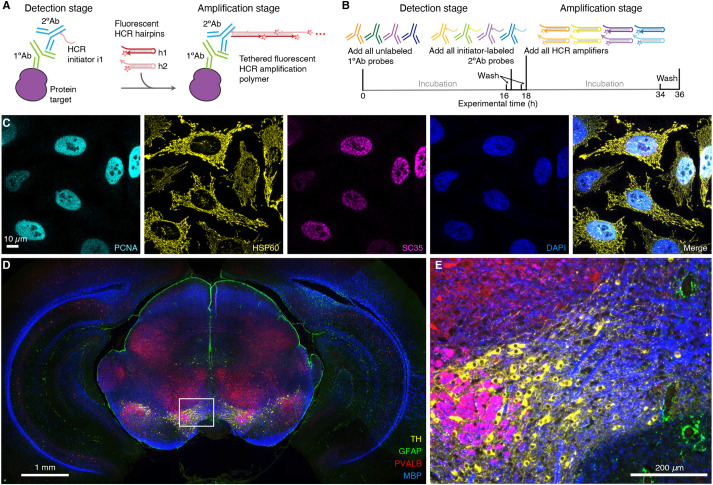
**Multiplexed protein imaging via HCR 2°IHC using unlabeled primary antibody probes, initiator-labeled secondary antibody probes and simultaneous HCR signal amplification for all targets.** (A) Two-stage HCR 2°IHC protocol. Detection stage: unlabeled primary antibody probes bind to protein targets; wash; initiator-labeled secondary antibody probes bind to primary antibody probes; wash. Amplification stage: initiators trigger self-assembly of fluorophore-labeled HCR hairpins into tethered fluorescent amplification polymers; wash. (B) Multiplexing timeline. The same two-stage protocol is used independent of the number of target proteins. (C) Confocal image of 3-plex protein imaging in mammalian cells on a slide; 0.14×0.14 µm pixels; maximum intensity *z*-projection. Target proteins: PCNA (Alexa647), HSP60 (Alexa546) and SC35 (Alexa488). Sample: HeLa cells. (D) Epifluorescence image of 4-plex protein imaging in FFPE mouse brain sections; 0.6×0.6 µm pixels. Target proteins: TH (Alexa488), GFAP (Alexa546), PVALB (Alexa647) and MBP (Alexa750). (E) Zoom of indicated region in D. Sample: FFPE C57BL/6 mouse brain section (coronal); 5 µm thickness. See sections S5.3 and S5.4 of the supplementary information for additional data.

Fig. 2 demonstrates multiplexed protein imaging via HCR 1°IHC using initiator-labeled primary antibody probes. Fig. 3 demonstrates multiplexed protein imaging via HCR 2°IHC using unlabeled primary antibody probes and initiator-labeled secondary antibody probes. Both methods achieve high signal-to-background for 3-plex protein imaging in mammalian cells and for 4-plex protein imaging in FFPE mouse brain sections. Across 21 protein imaging scenarios (six in mammalian cells, ten in FFPE mouse brain sections, four in FFPE human breast tissue sections and one in whole-mount zebrafish embryos; nine using HCR 1°IHC and 12 using HCR 2°IHC; 11 using confocal microscopy and ten using epifluorescence microscopy), the estimated signal-to-background ratio for protein targets ranged from 15 to 609 with a median of 90 (see Tables S9 and S10 for additional details). The level of performance demonstrated in Figs 2 and 3 was achieved for all targets simultaneously in 4-channel and 5-channel images (including a DAPI channel in each case) using fluorophores that compete with lower autofluorescence (Alexa647) as well as with higher autofluorescence (Alexa488) and in samples with lower autofluorescence (mammalian cells) and higher autofluorescence (FFPE mouse brain sections).

Using HCR signal amplification, the amplification gain corresponds to the number of fluorophore-labeled hairpins per amplification polymer. Hence, we were curious to measure the mean HCR polymer length in the context of HCR 1°IHC and HCR 2°IHC experiments. We can estimate HCR amplification gain by comparing the signal intensity in HCR experiments using h1 and h2 hairpins together (enabling polymerization to proceed as normal) versus using only hairpin h1 (so that each HCR initiator can bind only one HCR hairpin and polymerization cannot proceed). Across four measurement scenarios (two in mammalian cells and two in FFPE mouse brain sections; two using HCR 1°IHC and two using HCR 2°IHC), we observed a median polymer length of ≈180 hairpins (see section S5.5 in the supplementary information). It is this amplification gain that boosts the signal above autofluorescence to yield a high signal-to-background ratio even in FFPE tissues and whole-mount vertebrate embryos.

### qHCR imaging: protein relative quantitation with subcellular resolution in an anatomical context

**Fig. 4.**
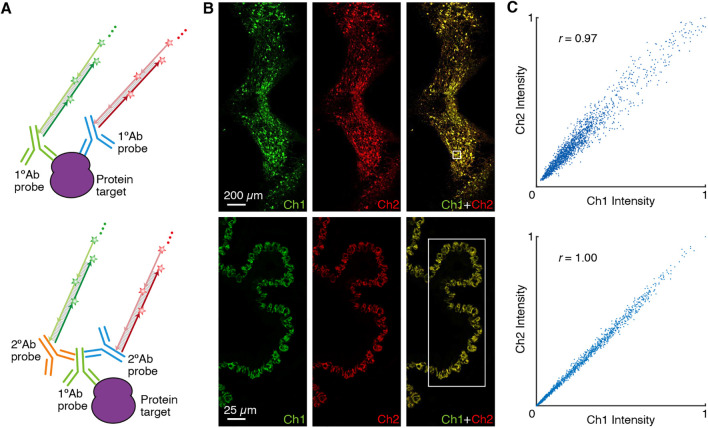
**qHCR imaging: protein relative quantitation with subcellular resolution in an anatomical context using HCR 1°IHC or HCR 2°IHC.** (A) Two-channel redundant detection of a target protein. Top: target protein detected using two primary antibody probes that bind different epitopes, each initiating an orthogonal spectrally distinct HCR amplifier (Ch1, Alexa647; Ch2, Alexa750). Bottom: target protein detected using an unlabeled primary antibody probe and two batches of secondary antibody probes that initiate orthogonal spectrally distinct HCR amplifiers (Ch1, Alexa546; Ch2, Alexa647). (B) Top: epifluorescence image of FFPE mouse brain section; 0.16×0.16 µm pixels. Target protein: TH. Sample: FFPE C57BL/6 mouse brain section (coronal); 5 µm thickness. Bottom: confocal image of FFPE human breast tissue; 0.3×0.3 µm pixels; single optical section. Target protein: KRT17. Sample: FFPE human breast tissue section; 5 µm thickness. (C) High accuracy and precision for protein relative quantitation in an anatomical context. Highly correlated normalized signal (Pearson correlation coefficient, *r*) for subcellular voxels in the indicated region in B (top: 2×2 µm voxels in a 5 µm section using epifluorescence microscopy; bottom: 2.0×2.0×2.5 µm voxels using confocal microscopy). Accuracy: linearity with zero intercept. Precision: scatter around the line. See section S5.6 of the supplementary information for additional data.

We have previously demonstrated that HCR RNA-ISH overcomes the historical tradeoff between RNA quantitation and anatomical context, enabling mRNA relative quantitation (qHCR imaging) with subcellular resolution within whole-mount vertebrate embryos (Trivedi et al., 2018; Choi et al., 2018). Here, we demonstrate that HCR IHC enables analogous subcellular quantitation of proteins in an anatomical context. To test protein relative quantitation, we first redundantly detected a target protein using two primary antibody probes that bind different epitopes on the same protein and trigger different spectrally distinct HCR amplifiers (Fig. 4A; top), yielding a two-channel image (Fig. 4B; top). If HCR signal scales approximately linearly with the number of target proteins per voxel, a two-channel scatter plot of normalized voxel intensities will yield a tight linear distribution with approximately zero intercept (Trivedi et al., 2018). On the other hand, observing a tight linear distribution with approximately zero intercept (Fig. 4C; top), we conclude that the HCR signal scales approximately linearly with the number of target proteins per imaging voxel, after first ruling out potential systematic crowding effects that could permit pairwise voxel intensities to slide undetected along a line (Fig. S24). Using one initiator-labeled primary antibody probe per channel, we observe high accuracy (linearity with zero intercept) and precision (scatter around the line) for subcellular 2×2 µm voxels within 5 µm FFPE mouse brain sections using epifluorescence microscopy. This redundant detection experiment provides a conservative characterization of quantitative performance as there is the risk that two antibody probes may interfere with each other to some extent when attempting to bind different epitopes on the same target protein. As a further test of quantitative imaging characteristics, we detected a protein target with unlabeled primary antibody probes that are subsequently detected by two batches of secondary antibody probes that trigger different spectrally distinct HCR amplifiers (Fig. 4A; bottom). This experiment is testing the accuracy and precision of the secondary antibody probes and HCR signal amplification, but not that of the primary antibody probes. In FFPE human breast tissue sections using confocal microscopy (Fig. 4B; bottom), a two-channel scatter plot of voxel intensities for subcellular 2.0×2.0×2.5 µm voxels again reveals a tight linear distribution with approximately zero intercept (Fig. 4C; bottom). Based on these two studies, we conclude that qHCR imaging enables accurate and precise relative quantitation of protein targets in an anatomical context with subcellular resolution, just as it does for mRNA targets (Trivedi et al., 2018; Choi et al., 2018).

### Simultaneous multiplexed protein and RNA imaging using HCR 1°IHC + HCR RNA-ISH or HCR 2°IHC + HCR RNA-ISH

**Fig. 5.**
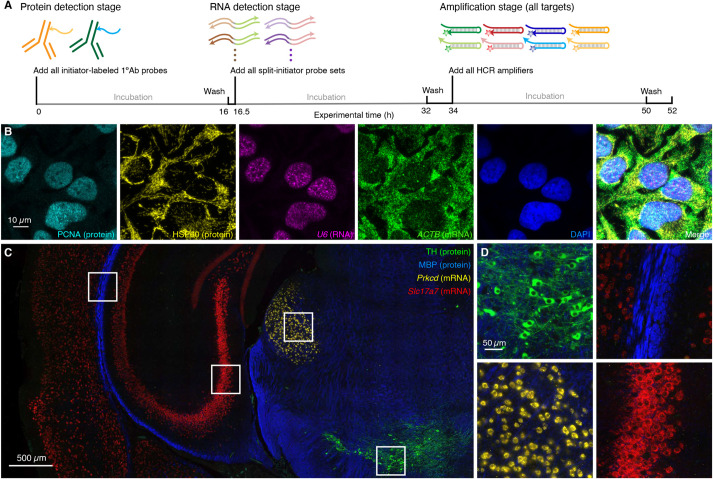
**Simultaneous multiplexed protein and RNA imaging via HCR 1°IHC + HCR RNA-ISH using initiator-labeled primary antibody probes for protein targets, split-initiator DNA probes for RNA targets, and simultaneous HCR signal amplification for all targets.** (A) Three-stage HCR 1°IHC + HCR RNA-ISH protocol. Protein detection stage: initiator-labeled primary antibody probes bind to protein targets; wash. RNA detection stage: split-initiator DNA probes bind to RNA targets; wash. Amplification stage: initiators trigger self-assembly of fluorophore-labeled HCR hairpins into tethered fluorescent amplification polymers; wash. For multiplexed experiments, the same three-stage protocol is used independent of the number of target proteins and RNAs. (B) Confocal image of 4-plex protein and RNA imaging in mammalian cells on a slide; 0.13×0.13 µm pixels; maximum intensity *z*-projection. Targets: PCNA (protein; Alexa488), HSP60 (protein; Alexa546), *U6* (RNA; Alexa594) and *ACTB* (mRNA; Alexa647). Sample: HeLa cells. (C) Epifluorescence image of 4-plex protein and RNA imaging in FFPE mouse brain sections; 0.16×0.16 µm pixels. Targets: TH (protein; Alexa488), MBP (protein; Alexa546), *Prkcd* (mRNA; Alexa647) and *Slc17a7* (mRNA; Alexa750). Sample: FFPE C57BL/6 mouse brain section (coronal); 5 µm thickness. (D) Zooms of indicated regions in C. See sections S5.7 and S5.8 of the supplementary information for additional data.

**Fig. 6.**
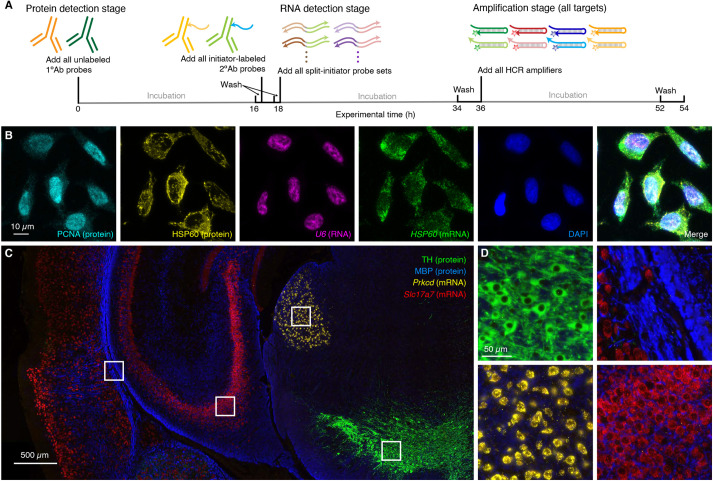
**Simultaneous multiplexed protein and RNA imaging via HCR 2°IHC + HCR RNA-ISH using unlabeled primary antibody probes and initiator-labeled secondary antibody probes for protein targets, split-initiator DNA probes for RNA targets, and simultaneous HCR signal amplification for all targets.** (A) Three-stage HCR 2°IHC + HCR RNA-ISH protocol. Protein detection stage: unlabeled primary antibody probes bind to protein targets; wash; initiator-labeled secondary antibody probes bind to primary antibody probes; wash. RNA detection stage: split-initiator DNA probes bind to RNA targets; wash. Amplification stage: initiators trigger self-assembly of fluorophore-labeled HCR hairpins into tethered fluorescent amplification polymers; wash. For multiplexed experiments, the same three-stage protocol is used independent of the number of target proteins and RNAs. (B) Confocal image of 4-plex protein and RNA imaging in mammalian cells on a slide; 0.13×0.13 µm pixels; maximum intensity *z*-projection. Targets: PCNA (protein; Alexa488), HSP60 (protein; Alexa546), *U6* (RNA; Alexa594) and *HSP60* (mRNA; Alexa647). Sample: HeLa cells. (C) Epifluorescence image of 4-plex protein and RNA imaging in FFPE mouse brain sections; 0.16×0.16 µm pixels. Targets: TH (protein; Alexa488), MBP (protein; Alexa546), *Prkcd* (mRNA; Alexa647) and *Slc17a7* (mRNA; Alexa750). Sample: FFPE C57BL/6 mouse brain section (coronal); 5 µm thickness. (D) Zooms of indicated regions in C. See sections S5.9 and S5.10 of the supplementary information for additional data.

It is important for biologists, drug developers and pathologists to have the flexibility to image proteins and RNAs simultaneously so as to enable interrogation of both levels of gene expression in the same specimen. Here, we demonstrate that HCR 1°IHC and HCR 2°IHC are both compatible with HCR RNA-ISH, enabling multiplexed quantitative protein and RNA imaging with high signal-to-background. Fig. 5 demonstrates HCR 1°IHC + HCR RNA-ISH (2-plex protein + 2-plex RNA) in mammalian cells and FFPE mouse brain sections using initiator-labeled primary antibody probes for protein targets, split-initiator DNA probes for RNA targets, and simultaneous HCR signal amplification for all targets. Fig. 6 demonstrates HCR 2°IHC + HCR RNA-ISH (2-plex protein + 2-plex RNA) in mammalian cells and FFPE mouse brain sections using unlabeled primary antibody probes and initiator-labeled secondary antibody probes for protein targets, split-initiator DNA probes for RNA targets, and simultaneous HCR signal amplification for all targets. Across 16 protein and RNA imaging scenarios (eight in mammalian cells and eight in FFPE mouse brain sections; eight using HCR 1°IHC + HCR RNA-ISH and eight using HCR 2°IHC + HCR RNA-ISH; eight using confocal microscopy and eight using epifluorescence microscopy), the estimated signal-to-background ratio for each target protein or RNA ranged from 20 to 700, with a median of 100 (see Tables S9 and S11 for additional details).

## DISCUSSION

qHCR imaging enables a unified approach to multiplexed quantitative IHC and RNA-ISH. A single experiment yields accurate and precise relative quantitation of both protein and RNA targets with subcellular resolution in the anatomical context of highly autofluorescent samples. No extra work is necessary to perform quantitative imaging – it is a natural property of HCR signal amplification. Here, we validated two complementary approaches for HCR IHC. Using HCR 1°IHC (initiator-labeled primary antibody probes), each target protein in a multiplexed experiment can be detected with antibodies raised in the same host species, which is often convenient based on available antibody libraries. However, antibody-initiator conjugation must be validated for each new primary antibody probe. Alternatively, using HCR 2°IHC (unlabeled primary antibody probes and initiator-labeled secondary antibody probes), each target protein in a multiplexed experiment must be detected with primary antibodies raised in different host species, thus enabling subsequent binding by initiator-labeled secondary antibodies that react with those different host species. This approach has the benefit that a small library of initiator-labeled secondary antibodies can be validated *a priori* and then used with large libraries of (unmodified) validated primary antibodies, enabling a plug-and-play approach using validated reagents. For simultaneous protein and RNA imaging: during the protein detection stage, M target proteins are detected in parallel; during the RNA detection stage, N target RNAs are detected in parallel; and during the amplification stage, one-step quantitative HCR signal amplification is performed for all M+N protein and RNA targets simultaneously. In 4-plex experiments in FFPE tissue sections, protein and RNA targets are simultaneously imaged with high signal-to-background in all four channels using fluorophores that compete with varying degrees of autofluorescence. For protein imaging using HCR 1°IHC or HCR 2°IHC, we favor protocols with two overnight incubations (Figs 2B and 3B), and for simultaneous protein and RNA imaging using HCR 1°IHC + HCR RNA-ISH or HCR 2°IHC + HCR RNA-ISH, we favor protocols with three overnight incubations (Figs 5A and 6A), allowing researchers to maintain a normal sleep schedule.

HCR RNA-ISH provides automatic background suppression throughout the protocol, ensuring that reagents will not generate amplified background even if they bind non-specifically within the sample (Choi et al., 2018). During the detection stage, each RNA target is detected by a probe set comprising one or more pairs of split-initiator probes, each carrying a fraction of HCR initiator i1 (Fig. 1B). For a given probe pair, probes that hybridize specifically to their adjacent binding sites on the target RNA colocalize full initiator i1, enabling cooperative initiation of HCR signal amplification. Meanwhile, any individual probes that bind non-specifically in the sample do not colocalize full initiator i1, do not trigger HCR and thus suppress generation of amplified background. During the amplification stage, automatic background suppression is inherent to HCR hairpins because polymerization is conditional on the presence of the initiator i1; individual h1 or h2 hairpins that bind non-specifically in the sample do not trigger formation of an amplification polymer. For HCR IHC, during the detection stage, each target protein is detected using primary or secondary antibody probes carrying one or more full i1 initiators (Fig. 1C,D). Hence, if an antibody probe binds non-specifically in the sample, initiator i1 will nonetheless trigger HCR, generating amplified background. As a result, it is important to use antibody probes that are highly selective for their targets, and to wash unused antibody probes from the sample. Nonetheless, during the amplification stage, kinetically trapped HCR hairpins provide automatic background suppression for protein targets just as they do for RNA targets, ensuring that any hairpins that bind non-specifically in the sample do not trigger growth of an HCR amplification polymer. For experiments using HCR IHC + HCR RNA-ISH to image protein and RNA targets simultaneously, RNA targets enjoy automatic background suppression throughout the protocol, whereas protein targets rely on selective antibody binding to suppress background during the detection stage, combined with automatic background suppression during the amplification stage.

For RNA targets, we have previously shown that multiplexed qHCR imaging enables bi-directional quantitative discovery (Trivedi et al., 2018): read-out from anatomical space to expression space to discover co-expression relationships in selected regions of the sample; read-in from expression space to anatomical space to discover those anatomical locations in which selected gene co-expression relationships occur. Here, by validating high-accuracy, high-precision, high-resolution qHCR imaging for protein targets, read-out/read-in analyses can now be performed for RNA and protein targets simultaneously, offering biologists, drug developers and pathologists a significantly expanded window for analyzing biological circuits in an anatomical context.

## MATERIALS AND METHODS

### Probes, amplifiers and buffers

Details on the probes, amplifiers and buffers for each experiment are displayed in Table S2 for HCR 1°IHC, in Table S3 for HCR 2°IHC and in Table S4 for HCR RNA-ISH. HCR initiators were conjugated to antibody probes using the Antibody-Oligonucleotide All-in-One Conjugation Kit (Vector Laboratories, A-9202) according to the manufacturer's instructions.

### HCR IHC with/without HCR RNA-ISH

HCR 1°IHC with/without HCR RNA-ISH was performed using the protocols detailed in section S3 in the supplementary information. HCR 2°IHC with/without HCR RNA-ISH was performed using the protocols detailed in section S4 in the supplementary information. These IHC protocols with/without HCR RNA-ISH were developed starting from HCR RNA-ISH protocols (Choi et al., 2018). The optional autofluorescence bleaching protocol for FFPE mouse brain tissue sections, combining photo- (Duong and Han, 2013) and chemical (Lin et al., 2018a) bleaching, was used only for the HCR IHC + HCR RNA-ISH studies of Figs 5C,D and 6C,D, and the associated replicates in Figs S35, S36, S43 and S44. Strictly speaking, the cultured cell studies represent immunocytochemistry (ICC) rather than IHC; for notational simplicity, we use the term IHC uniformly in the main text but denote protocols for cultured cells as ICC in the supplementary information. For five-channel imaging of HeLa cells (Figs 5B, S33, S34, 6B, S41, S42) the above protocols were modified as follows to enable imaging on an upright confocal microscope: cells were grown on a chambered slide with removable chambers (Ibidi, 81201); prior to imaging, the silicone chambers were removed and cells were mounted with ProLong glass antifade mountant with NucBlue (Thermo Fisher Scientific, P36981) according to the manufacturer's instructions.

Experiments were performed in HeLa cells (ATCC, CRM-CCL-2), FFPE C57BL/6 mouse brain sections (coronal; thickness 5 µm, Acepix Biosciences 7011-0120), FFPE human breast tissue sections (thickness 5 µm; Acepix Biosciences, 7310-0620) and whole-mount zebrafish embryos (wildtype *Danio rerio* strain AB; fixed at 27 hpf). Procedures for the care and use of zebrafish embryos were approved by the Caltech IACUC.

### Confocal microscopy

Confocal microscopy was performed using a Zeiss LSM 800 inverted confocal microscope or a Zeiss LSM 880 with Fast Airyscan upright confocal microscope. All confocal images are displayed without background subtraction. See Table S5 for details on the microscope, objective, excitation lasers, beam splitters and emission bandpass filters used for each experiment.

### Epifluorescence microscopy

Epifluorescence microscopy was performed using a Leica THUNDER Imager 3D cell culture epifluorescence microscope equipped with a Leica LED8 multi-LED light source and sCMOS camera (Leica DFC9000 GTC). All epifluorescence images were acquired without THUNDER computational clearing and are displayed with instrument noise subtracted but without background subtraction. See Table S6 for details on the objective, excitation wavelengths and filters used for each experiment.

### Image analysis

Image analysis was performed as detailed in section S2.6 of the supplementary information, including: definition of raw pixel intensities; measurement of signal, background and signal-to-background; measurement of background components and calculation of normalized subcellular voxel intensities for qHCR imaging.

## Supplementary Material

Supplementary information

## References

[DEV199847C1] Acloque, H., Wilkinson, D. G. and Nieto, M. A. (2008). In situ hybridization analysis of chick embryos in whole-mount and tissue sections. In *Avian Embryology*, 2nd edn. (ed. M. Bronner-Fraser), pp. 169-185. San Diego, CA: Elsevier Academic Press. 10.1016/S0091-679X(08)00209-4 18485297

[DEV199847C2] Ahnfelt-Ronne, J., Jorgensen, M. C., Hald, J., Madsen, O. D., Serup, P. and Hecksher-Sorensen, J. (2007). An improved method for three-dimensional reconstruction of protein expression patterns in intact mouse and chicken embryos and organs. *J. Histochem. Cytochem.* 55, 925-930. 10.1369/jhc.7A7226.2007 17478445

[DEV199847C3] Barroso-Chinea, P., Aymerich, M. S., Castle, M. M., Pérez-Manso, M., Tuñón, T., Erro, E. and Lanciego, J. L. (2007). Detection of two different mRNAs in a single section by dual in situ hybridization: a comparison between colorimetric and fluorescent detection. *J. Neurosci. Methods* 162, 119-128. 10.1016/j.jneumeth.2006.12.017 17306886

[DEV199847C4] Chan, P. M., Yuen, T., Ruf, F., Gonzalez-Maeso, J. and Sealfon, S. C. (2005). Method for multiplex cellular detection of mRNAs using quantum dot fluorescent in situ hybridization. *Nucleic Acids Res.* 33, e161. 10.1093/nar/gni162 16224100 PMC1258180

[DEV199847C5] Choi, H. M. T., Chang, J. Y., Trinh, L. A., Padilla, J. E., Fraser, S. E. and Pierce, N. A. (2010). Programmable in situ amplification for multiplexed imaging of mRNA expression. *Nat. Biotechnol.* 28, 1208-1212. 10.1038/nbt.1692 21037591 PMC3058322

[DEV199847C6] Choi, H. M. T., Beck, V. A. and Pierce, N. A. (2014). Next-generation in situ hybridization chain reaction: higher gain, lower cost, greater durability. *ACS Nano* 8, 4284-4294. 10.1021/nn405717p 24712299 PMC4046802

[DEV199847C7] Choi, H. M. T., Calvert, C. R., Husain, N., Huss, D., Barsi, J. C., Deverman, B. E., Hunter, R. C., Kato, M., Lee, S. M., Abelin, A. C. T. et al. (2016). Mapping a multiplexed zoo of mRNA expression. *Development* 143, 3632-3637. 10.1242/dev.140137 27702788 PMC5087610

[DEV199847C8] Choi, H. M. T., Schwarzkopf, M., Fornace, M. E., Acharya, A., Artavanis, G., Stegmaier, J., Cunha, A. and Pierce, N. A. (2018). Third-generation in situ hybridization chain reaction: multiplexed, quantitative, sensitive, versatile, robust. *Development* 145, dev165753. 10.1242/dev.165753 29945988 PMC6031405

[DEV199847C9] Clay, H. and Ramakrishnan, L. (2005). Multiplex fluorescent in situ hybridization in zebrafish embryos using tyramide signal amplification. *Zebrafish* 2, 105-111. 10.1089/zeb.2005.2.105 18248170

[DEV199847C10] Coons, A. H., Creech, H. J. and Jones, R. N. (1941). Immunological properties of an antibody containing a fluorescent group. *Proc. Soc. Exp. Biol. Med.* 47, 200-202. 10.3181/00379727-47-13084P

[DEV199847C11] Denkers, N., García-Villalba, P., Rodesch, C. K., Nielson, K. R. and Mauch, T. J. (2004). FISHing for chick genes: triple–label whole–mount fluorescence in situ hybridization detects simultaneous and overlapping gene expression in avian embryos. *Dev. Dyn.* 229, 651-657. 10.1002/dvdy.20005 14991720

[DEV199847C12] Dirks, R. M. and Pierce, N. A. (2004). Triggered amplification by hybridization chain reaction. *Proc. Natl. Acad. Sci. USA* 101, 15275-15278. 10.1073/pnas.0407024101 15492210 PMC524468

[DEV199847C13] Duong, H. and Han, M. (2013). A multispectral LED array for the reduction of background autofluorescence in brain tissue. *J. Neurosci. Methods* 220, 46-54. 10.1016/j.jneumeth.2013.08.018 23994358 PMC3856220

[DEV199847C14] Femino, A. M., Fay, F. S., Fogarty, K. and Singer, R. H. (1998). Visualization of single RNA transcripts in situ. *Science* 280, 585-590. 10.1126/science.280.5363.585 9554849

[DEV199847C15] Fujisawa, S., Yarilin, D., Fan, N., Turkekul, M., Xu, K., Barlas, A. and Manova-Todorova, K. (2015). Understanding the three-dimensional world from two-dimensional immunofluorescent adjacent sections. *J. Pathol. Inform.* 6, 27. 10.4103/2153-3539.158052 26110094 PMC4470010

[DEV199847C16] Glass, G., Papin, J. A. and Mandell, J. W. (2009). SIMPLE: a sequential immunoperoxidase labeling and erasing method. *J. Histochem. Cytochem.* 57, 899-905. 10.1369/jhc.2009.953612 19365090 PMC2746723

[DEV199847C17] Gusev, Y., Sparkowski, J., Raghunathan, A., Ferguson, H., Jr, Montano, J., Bogdan, N., Schweitzer, B., Wiltshire, S., Kingsmore, S. F., Maltzman, W. et al. (2001). Rolling circle amplification: a new approach to increase sensitivity for immunohistochemistry and flow cytometry. *Am. J. Pathol.* 159, 63-69. 10.1016/S0002-9440(10)61674-4 11438455 PMC1850404

[DEV199847C18] Harland, R. M. (1991). In situ hybridization: an improved whole-mount method for *Xenopus* embryos. *Methods Cell Biol.* 36, 685-695. 10.1016/S0091-679X(08)60307-6 1811161

[DEV199847C19] Harrison, P. R., Conkie, D. and Paul, J. (1973). Localisation of cellular globin messenger RNA by in situ hybridisation to complementary DNA. *FEBS Lett.* 32, 109-112. 10.1016/0014-5793(73)80749-5 4123728

[DEV199847C20] Hughes, S. C. and Krause, H. M. (1998). Double labeling with fluorescence in situ hybridization in *Drosophila* whole-mount embryos. *BioTechniques* 24, 530-532. 10.2144/98244bm01 9564514

[DEV199847C21] Husain, N. (2016). Mapping mRNA and protein expression with high signal-to-background in diverse organisms. *PhD Thesis*. California Institute of Technology.

[DEV199847C22] Kerstens, H. M. J., Poddighe, P. J. and Hanselaar, A. G. J. M. (1995). A novel *in-situ* hybridization signal amplification method based on the deposition of biotinylated tyramine. *J. Histochem. Cytochem.* 43, 347-352. 10.1177/43.4.7897179 7897179

[DEV199847C23] Kim, S.-W., Roh, J. and Park, C.-S. (2016). Immunohistochemistry for pathologists: protocols, pitfalls, and tips. *J. Pathol. Transl. Med.* 50, 411-418. 10.4132/jptm.2016.08.08 27809448 PMC5122731

[DEV199847C24] Kishi, J. Y., Lapan, S. W., Beliveau, B. J., West, E. R., Zhu, A., Sasaki, H. M., Saka, S. K., Wang, Y., Cepko, C. L. and Yin, P. (2019). SABER amplifies FISH: enhanced multiplexed imaging of RNA and DNA in cells and tissues. *Nat. Methods* 16, 533-544. 10.1038/s41592-019-0404-0 31110282 PMC6544483

[DEV199847C25] Kislauskis, E. H., Li, Z., Singer, R. H. and Taneja, K. L. (1993). Isoform-specific 3′-untranslated sequences sort α-cardiac and β-cytoplasmic actin messenger RNAs to different cytoplasmic compartments. *J. Cell Biol.* 123, 165-172. 10.1083/jcb.123.1.165 8408195 PMC2119818

[DEV199847C26] Koos, B., Cane, G., Grannas, K., Löf, L., Arngården, L., Heldin, J., Clausson, C.-M., Klaesson, A., Hirvonen, M. K., de Oliveira, F. M. S. et al. (2015). Proximity-dependent initiation of hybridization chain reaction. *Nat. Commun.* 6, 7294. 10.1038/ncomms8294 26065580 PMC4490387

[DEV199847C27] Kosman, D., Mizutani, C. M., Lemons, D., Cox, W. G., McGinnis, W. and Bier, E. (2004). Multiplex detection of RNA expression in *Drosophila* embryos. *Science* 305, 846. 10.1126/science.1099247 15297669

[DEV199847C28] Larsson, C., Grundberg, I., Söderberg, O. and Nilsson, M. (2010). In situ detection and genotyping of individual mRNA molecules. *Nat. Methods* 7, 395-397. 10.1038/nmeth.1448 20383134

[DEV199847C29] Lehmann, R. and Tautz, D. (1994). In situ hybridization to RNA. In *Drosophila Melanogaster: Practical Uses in Cell and Molecular Biology* (ed. L. S. B. Goldstein and E. A. Fyrberg), pp. 575-598. San Diego, CA: Elsevier Academic Press. 10.1016/s0091-679x(08)60933-4 7535885

[DEV199847C30] Lin, J.-R., Izar, B., Wang, S., Yapp, C., Mei, S., Shah, P. M., Santagata, S. and Sorger, P. K. (2018a). Highly multiplexed immunofluorescence imaging of human tissues and tumors using t-CyCIF and conventional optical microscopes. *eLife* 7, e31657. 10.7554/eLife.31657 29993362 PMC6075866

[DEV199847C31] Lin, R., Feng, Q., Li, P., Zhou, P., Wang, R., Liu, Z., Wang, Z., Qi, X., Tang, N., Shao, F. et al. (2018b). A hybridization-chain-reaction-based method for amplifying immunosignals. *Nat. Methods* 15, 275-278. 10.1038/nmeth.4611 29481551

[DEV199847C32] Macechko, P. T., Krueger, L., Hirsch, B. and Erlandsen, S. L. (1997). Comparison of immunological amplification versus enzymatic deposition of fluorochrome-conjugated tyramide as detection systems for FISH. *J. Histochem. Cytochem.* 45, 359-363. 10.1177/002215549704500303 9071317

[DEV199847C33] Martínez, A., Miller, M. J., Quinn, K., Unsworth, E. J., Ebina, M. and Cuttitta, F. (1995). Non-radioactive localization of nucleic acids by direct in situ PCR and in situ RT-PCR in paraffin-embedded sections. *J. Histochem. Cytochem.* 43, 739-747. 10.1177/43.8.7542678 7542678

[DEV199847C34] Mitchell, R. T., Camacho-Moll, M. E., Macdonald, J., Anderson, R. A., Kelnar, C. J. H., O'Donnell, M., Sharpe, R. M., Smith, L. B., Grigor, K. M., Wallace, W. H. B. et al. (2014). Intratubular germ cell neoplasia of the human testis: heterogeneous protein expression and relation to invasive potential. *Mod. Pathol.* 27, 1255-1266. 10.1038/modpathol.2013.246 24457464 PMC4012991

[DEV199847C35] Nieto, M. A., Patel, K. and Wilkinson, D. G. (1996). In situ hybridization analysis of chick embryos in whole mount and tissue sections. In *Methods in Avian Embryology* (ed. M. Bronner-Fraser), pp. 219-235. San Diego, CA: Elsevier Academic Press. 10.1016/s0091-679x(08)60630-5 8722478

[DEV199847C36] Nuovo, G. J., Gorgone, G. A., MacConnell, P., Margiotta, M. and Gorevic, P. D. (1992). In situ localization of PCR-amplified human and viral cDNAs. *Genome Res.* 2, 117-123. 10.1101/gr.2.2.117 1282436

[DEV199847C37] Piette, D., Hendrickx, M., Willems, E., Kemp, C. R. and Leyns, L. (2008). An optimized procedure for whole-mount in situ hybridization on mouse embryos and embryoid bodies. *Nat. Protoc.* 3, 1194-1201. 10.1038/nprot.2008.103 18600225

[DEV199847C38] Player, A. N., Shen, L.-P., Kenny, D., Antao, V. P. and Kolberg, J. A. (2001). Single-copy gene detection using branched DNA (bDNA) in situ hybridization. *J. Histochem. Cytochem.* 49, 603-611. 10.1177/002215540104900507 11304798

[DEV199847C39] Qian, X. and Lloyd, R. V. (2003). Recent developments in signal amplification methods for in situ hybridization. *Diagn. Mol. Pathol.* 12, 1-13. 10.1097/00019606-200303000-00001 12605030

[DEV199847C40] Qian, X., Jin, L. and Lloyd, R. V. (2004). In situ hybridization: basic approaches and recent development. *J. Histotechnol.* 27, 53-67. 10.1179/his.2004.27.1.53

[DEV199847C41] Raj, A., van den Bogaard, P., Rifkin, S. A., van Oudenaarden, A. and Tyagi, S. (2008). Imaging individual mRNA molecules using multiple singly labeled probes. *Nat. Methods* 5, 877-879. 10.1038/nmeth.1253 18806792 PMC3126653

[DEV199847C42] Ramos-Vara, J. A. (2005). Technical aspects of immunohistochemistry. *Vet. Pathol.* 42, 405-426. 10.1354/vp.42-4-405 16006601

[DEV199847C43] Ramos-Vara, J. A. and Miller, M. A. (2014). When tissue antigens and antibodies get along: revisiting the technical aspects of immunohistochemistry-the red, brown, and blue technique. *Vet. Pathol.* 51, 42-87. 10.1177/0300985813505879 24129895

[DEV199847C44] Saka, S. K., Wang, Y., Kishi, J. Y., Zhu, A., Zeng, Y., Xie, W., Kirli, K., Yapp, C., Cicconet, M., Beliveau, B. J. et al. (2019). Immuno-SABER enables highly multiplexed and amplified protein imaging in tissues. *Nat. Biotechnol.* 37, 1080-1090. 10.1038/s41587-019-0207-y 31427819 PMC6728175

[DEV199847C45] Shah, S., Lubeck, E., Schwarzkopf, M., He, T.-F., Greenbaum, A., Sohn, C. H., Lignell, A., Choi, H. M. T., Gradinaru, V., Pierce, N. A. et al. (2016). Single-molecule RNA detection at depth via hybridization chain reaction and tissue hydrogel embedding and clearing. *Development* 143, 2862-2867. 10.1242/dev.138560 27342713 PMC5004914

[DEV199847C46] Sillitoe, R. V. and Hawkes, R. (2002). Whole-mount immunohistochemistry: a high-throughput screen for patterning defects in the mouse cerebellum. *J. Histochem. Cytochem.* 50, 235-244. 10.1177/002215540205000211 11799142

[DEV199847C47] Stack, E. C., Wang, C. C., Roman, K. A. and Hoyt, C. C. (2014). Multiplexed immunohistochemistry, imaging, and quantitation: A review, with an assessment of Tyramide signal amplification, multispectral imaging and multiplex analysis. *Methods* 70, 46-58. 10.1016/j.ymeth.2014.08.016 25242720

[DEV199847C48] Staudt, N., Müller-Sienerth, N., Fane-Dremucheva, A., Yusaf, S. P., Millrine, D. and Wright, G. J. (2015). A panel of recombinant monoclonal antibodies against zebrafish neural receptors and secreted proteins suitable for wholemount immunostaining. *Biochem. Biophys. Res. Commun.* 456, 527-533. 10.1016/j.bbrc.2014.11.123 25490391 PMC4297863

[DEV199847C49] Takakura, N., Yoshida, H., Ogura, Y., Kataoka, H., Nishikawa, S.-I. and Nishikawa, S. (1997). PDGFRα expression during mouse embryogenesis: immunolocalization analyzed by whole-mount immunohistostaining using the monoclonal anti-mouse PDGFRα antibody APA5. *J. Histochem. Cytochem.* 45, 883-893. 10.1177/002215549704500613 9199674

[DEV199847C50] Tautz, D. and Pfeifle, C. (1989). A non-radioactive in situ hybridization method for the localization of specific RNAs in *Drosophila* embryos reveals translational control of the segmentation gene hunchback. *Chromosoma* 98, 81-85. 10.1007/BF00291041 2476281

[DEV199847C51] Thisse, C. and Thisse, B. (2008). High-resolution in situ hybridization to whole-mount zebrafish embryos. *Nat. Protoc.* 3, 59-69. 10.1038/nprot.2007.514 18193022

[DEV199847C52] Thisse, B., Heyer, V., Lux, A., Alunni, V., Degrave, A., Seiliez, I., Kirchner, J., Parkhill, J. P. and Thisse, C. (2004). Spatial and temporal expression of the zebrafish genome by large-scale in situ hybridization screening. In *The Zebrafish: 2nd Edition Genetics Genomics and Informatics* (ed. H. W. D. Detrich III, L. I. Zon and M. Westerfield), pp. 505-519. San Diego, CA: Elsevier Academic Press. 10.1016/s0091-679x(04)77027-2 15602929

[DEV199847C53] Tóth, Z. E. and Mezey, E. (2007). Simultaneous visualization of multiple antigens with tyramide signal amplification using antibodies from the same species. *J. Histochem. Cytochem.* 55, 545-554. 10.1369/jhc.6A7134.2007 17242468

[DEV199847C54] Trivedi, V., Choi, H. M. T., Fraser, S. E. and Pierce, N. A. (2018). Multidimensional quantitative analysis of mRNA expression within intact vertebrate embryos. *Development* 145, dev156869. 10.1242/dev.156869 29311262 PMC5825878

[DEV199847C55] Tsujikawa, T., Kumar, S., Borkar, R. N., Azimi, V., Thibault, G., Chang, Y. H., Balter, A., Kawashima, R., Choe, G., Sauer, D. et al. (2017). Quantitative multiplex immunohistochemistry reveals myeloid-inflamed tumor-immune complexity associated with poor prognosis. *Cell Rep.* 19, 203-217. 10.1016/j.celrep.2017.03.037 28380359 PMC5564306

[DEV199847C56] Wang, F., Flanagan, J., Su, N., Wang, L.-C., Bui, S., Nielson, A., Wu, X. Y., Vo, H.-T., Ma, X.-J. and Luo, Y. L. (2012). RNAscope: a novel in situ RNA analysis platform for formalin-fixed, paraffin-embedded tissues. *J. Mol. Diagn.* 14, 22-29. 10.1016/j.jmoldx.2011.08.002 22166544 PMC3338343

[DEV199847C57] Weiszmann, R., Hammonds, A. S. and Celniker, S. E. (2009). Determination of gene expression patterns using high-throughput RNA in situ hybridization to whole-mount *Drosophila* embryos. *Nat. Protoc.* 4, 605-618. 10.1038/nprot.2009.55 19360017 PMC2780369

[DEV199847C58] Wiedorn, K. H., Kühl, H., Galle, J., Caselitz, J. and Vollmer, E. (1999). Comparison of *in-situ* hybridization, direct and indirect *in-situ* PCR as well as tyramide signal amplification for the detection of HPV. *Histochem. Cell Biol.* 111, 89-95. 10.1007/s004180050338 10090569

[DEV199847C59] Zhou, Y., Calciano, M., Hamann, S., Leamon, J. H., Strugnell, T., Christian, M. W. and Lizardi, P. M. (2001). In situ detection of messenger RNA using digoxigenin-labeled oligonucleotides and rolling circle amplification. *Exp. Mol. Pathol.* 70, 281-288. 10.1006/exmp.2001.2365 11418007

