# Supplementary material for: Hybridization chain reaction enables a unified approach to multiplexed, quantitative, high-resolution immunohistochemistry and in situ hybridization
Source: Development. 2021 Nov 18;148(22):dev199847. doi: 10.1242/dev.199847 (PMC8645210; doi:10.1242/dev.199847)
Supplement: Supplementary information [file develop-148-199847-s1.pdf]

Supplementary Information

Hybridization chain reaction enables a unified approach to multiplexed, quantitative, high-resolution immunohistochemistry and in situ hybridization

Maayan Schwarzkopf,[1,†] Mike C. Liu,[2,†] Samuel J. Schulte,[1,‡] Rachel Ives,[2,‡] Naeem Husain,[1]
Harry M.T. Choi[2,*], and Niles A. Pierce[1,3,*]

Contents

S1 HCR RNA-ISH in diverse organisms and sample types **7**

S2 Additional materials and methods **9**
- S2.1 Probe and amplifier details for protein targets using HCR 1°IHC 9
- S2.2 Probe and amplifier details for protein targets using HCR 2°IHC 10
- S2.3 Probe and amplifier details for RNA targets using HCR RNA-ISH 11
- S2.4 Confocal microscope settings 12
- S2.5 Epifluorescence microscope settings 13
- S2.6 Image analysis 14
  - S2.6.1 Raw pixel intensities 14
  - S2.6.2 Measurement of signal, background, noise, and signal-to-background for HCR 1°IHC, HCR 2°IHC, and HCR RNA-ISH 14
  - S2.6.3 Measurement of background components for HCR 1°IHC and HCR 2°IHC 16
  - S2.6.4 Measurement of HCR amplification gain (i.e., amplification polymer length) 17
  - S2.6.5 Normalized voxel intensities for qHCR imaging: protein relative quantitation with subcellular resolution in an anatomical context 19

S3 Protocols for HCR 1°IHC with/without HCR RNA-ISH **20**
- S3.1 Protocols for mammalian cells on a chambered slide 20
  - S3.1.1 Preparation of fixed mammalian cells on a chambered slide 20
  - S3.1.2 Multiplexed HCR 1°ICC with/without HCR RNA-ISH using initiator-labeled primary antibody probes for protein targets, split-initiator DNA probes for RNA targets, and simultaneous HCR signal amplification for all targets 21
  - S3.1.3 Buffers for HCR 1°ICC with/without HCR RNA-ISH 23
  - S3.1.4 Reagents and supplies 23
- S3.2 Protocols for FFPE mouse brain tissue sections 24
  - S3.2.1 Preparation of formalin-fixed paraffin-embedded (FFPE) mouse brain tissue sections 24
  - S3.2.2 Buffer recipes for sample preparation 25
  - S3.2.3 Autofluorescence bleaching protocol 26
  - S3.2.4 Buffer recipes for autofluorescence bleaching protocol 26

[1]Division of Biology & Biological Engineering, California Institute of Technology, Pasadena, CA 91125, USA. [2]Molecular Instruments, Inc., Los Angeles, CA 90041, USA. [3]Division of Engineering & Applied Science, California Institute of Technology, Pasadena, CA 91125, USA. [†]Authors contributed equally. [‡]Authors contributed equally. [*]Email: niles@caltech.edu and harry@molecularinstruments.com

S3.2.5 Multiplexed HCR 1°IHC with/without HCR RNA-ISH using initiator-labeled primary antibody probes for protein targets, split-initiator DNA probes for RNA targets, and simultaneous HCR signal amplification for all targets . . . 27
S3.2.6 Buffer for HCR 1°IHC with/without HCR RNA-ISH . . . 30
S3.2.7 Reagents and supplies . . . 30

S4 Protocols for HCR 2°IHC with/without HCR RNA-ISH **31**
S4.1 Protocols for mammalian cells on a chambered slide . . . 31
S4.1.1 Preparation of fixed mammalian cells on a chambered slide . . . 31
S4.1.2 HCR 2°ICC with/without HCR RNA-ISH using unlabeled primary antibody probes and initiator-labeled secondary antibody probes for protein targets, split-initiator DNA probes for RNA targets, and simultaneous HCR signal amplification for all targets . . . 32
S4.1.3 Buffers for HCR 2°ICC with/without HCR RNA-ISH . . . 34
S4.1.4 Reagents and supplies . . . 34
S4.2 Protocols for FFPE mouse brain tissue sections . . . 35
S4.2.1 Preparation of formalin-fixed paraffin-embedded (FFPE) mouse brain tissue sections . . . 35
S4.2.2 Buffer recipes for sample preparation . . . 36
S4.2.3 Autofluorescence bleaching protocol . . . 37
S4.2.4 Buffer recipes for autofluorescence bleaching protocol . . . 37
S4.2.5 Multiplexed HCR 2°IHC with/without HCR RNA-ISH using unlabeled primary antibody probes and initiator-labeled secondary antibody probes for protein targets, split-initiator DNA probes for RNA targets, and simultaneous HCR signal amplification for all targets . . . 38
S4.2.6 Buffers for HCR 2°IHC with/without HCR RNA-ISH . . . 41
S4.2.7 Reagents and supplies . . . 41
S4.3 Protocols for FFPE human breast tissue sections . . . 42
S4.3.1 Preparation of formalin-fixed paraffin-embedded (FFPE) human breast tissue sections . . . 42
S4.3.2 Buffer recipes for sample preparation . . . 42
S4.3.3 Multiplexed HCR 2°IHC using unlabeled primary antibody probes and initiator-labeled secondary probes with simultaneous HCR signal amplification for all targets . . . 43
S4.3.4 Buffers for HCR 2°IHC . . . 45
S4.3.5 Reagents and supplies . . . 45
S4.4 Protocols for whole-mount zebrafish embryos . . . 46
S4.4.1 Preparation of whole-mount zebrafish embryos . . . 46
S4.4.2 Buffer recipes for sample preparation . . . 46
S4.4.3 Multiplexed HCR 2°IHC using unlabeled primary antibody probes and initiator-labeled secondary antibody probes with simultaneous HCR signal amplification for all targets . . . 47
S4.4.4 Sample mounting for microscopy . . . 48
S4.4.5 Buffers for HCR 2°IHC . . . 49
S4.4.6 Reagents and supplies . . . 49

S5 Additional studies **50**
S5.1 Summary of signal-to-background estimates for HCR 1°IHC, HCR 2°IHC, and/or HCR RNA-ISH 50
S5.2 Replicates, signal, background, background components, and noise for multiplexed HCR 1°IHC (cf. Figure 2) . . . 52
S5.2.1 Mammalian cells on a slide . . . 52
S5.2.2 FFPE mouse brain sections . . . 58
S5.3 Replicates, signal, background, background components, and noise for multiplexed HCR 2°IHC (cf. Figure 3) . . . 65
S5.3.1 Mammalian cells on a slide . . . 65
S5.3.2 FFPE mouse brain sections . . . 71

Development • Supplementary information

S5.4 Protein imaging with high signal-to-background in whole-mount zebrafish embryos using HCR 2°IHC . . . 78
S5.5 Estimating HCR IHC polymer length (cf. Figures 2 and 3) . . . 80
S5.5.1 HCR 1°ICC in mammalian cells on a slide . . . 81
S5.5.2 HCR 2°ICC in mammalian cells on a slide . . . 83
S5.5.3 HCR 1°IHC in FFPE mouse brain sections . . . 85
S5.5.4 HCR 2°IHC in FFPE mouse brain sections . . . 86
S5.6 qHCR imaging: protein relative quantitation with subcellular resolution in an anatomical context (cf. Figure 4) . . . 87
S5.6.1 Testing for a crowding effect . . . 87
S5.6.2 Redundant 2-channel imaging of target protein TH using HCR 1°IHC in FFPE mouse brain sections . . . 93
S5.6.3 Redundant 2-channel imaging of target proteins KRT17 and KRT19 using HCR 2°IHC in FFPE human breast tissue sections . . . 96
S5.7 Replicates and signal-to-background measurements for simultaneous multiplexed protein and mRNA imaging using HCR 1°IHC and HCR RNA-ISH (cf. Figure 5) . . . 101
S5.7.1 Mammalian cells on a slide . . . 101
S5.7.2 FFPE mouse brain sections . . . 105
S5.8 Testing whether protein imaging using HCR 1°IHC is affected by RNA imaging using HCR RNA-ISH and vice versa (cf. Figure 5) . . . 109
S5.8.1 Mammalian cells on a slide . . . 110
S5.8.2 FFPE mouse brain sections . . . 114
S5.9 Replicates and signal-to-background measurements for simultaneous multiplexed protein and mRNA imaging using HCR 2°IHC and HCR RNA-ISH (cf. Figure 6) . . . 118
S5.9.1 Mammalian cells on a slide . . . 118
S5.9.2 FFPE mouse brain sections . . . 122
S5.10 Testing whether protein imaging using HCR 2°IHC is affected by RNA imaging using HCR RNA-ISH and vice versa (cf. Figure 6) . . . 126
S5.10.1 Mammalian cells on a slide . . . 127
S5.10.2 FFPE mouse brain sections . . . 131

References **135**

List of Figures

S1 Replicates for 3-plex protein imaging using HCR 1°ICC in mammalian cells on a slide (cf. Figures 2C) . . . 53
S2 Measurement of signal, background, and background components for target protein HSP60 using HCR 1°ICC in mammalian cells on a slide (cf. Figure 2C) . . . 54
S3 Measurement of signal, background, and background components for target protein GM130 using HCR 1°ICC in mammalian cells on a slide (cf. Figure 2C) . . . 55
S4 Measurement of signal, background, and background components for target protein SC35 using HCR 1°ICC in mammalian cells on a slide (cf. Figure 2C) . . . 56
S5 Replicates for 4-plex protein imaging using HCR 1°IHC in FFPE mouse brain sections (cf. Figures 2DE) . . . 59
S6 Measurement of signal, background, background components, and noise for target protein TH using HCR 1°IHC in FFPE mouse brain sections (cf. Figures 2DE) . . . 60
S7 Measurement of signal, background, background components, and noise for target protein GFAP using HCR 1°IHC in FFPE mouse brain sections (cf. Figures 2DE) . . . 61

Development • Supplementary information

S8 Measurement of signal, background, background components, and noise for target protein MBP using HCR 1°IHC in FFPE mouse brain sections (cf. Figures 2DE) 62
S9 Measurement of signal, background, background components, and noise for target protein MAP2 using HCR 1°IHC in FFPE mouse brain sections (cf. Figures 2DE) 63
S10 Replicates for 3-plex protein imaging using HCR 2°ICC in mammalian cells on a slide (cf. Figures 3C) 66
S11 Measurement of signal, background, and background components for target protein PCNA using HCR 2°ICC in mammalian cells on a slide (cf. Figure 3C) 67
S12 Measurement of signal, background, and background components for target protein HSP60 using HCR 2°ICC in mammalian cells on a slide (cf. Figure 3C) 68
S13 Measurement of signal, background, and background components for target protein SC35 using HCR 2°ICC in mammalian cells on a slide (cf. Figure 3C) 69
S14 Replicates for 4-plex protein imaging using HCR 2°IHC in FFPE mouse brain sections (cf. Figures 3DE) 72
S15 Measurement of signal, background, background components, and noise for protein target TH using HCR 2°IHC in FFPE mouse brain sections (cf. Figures 3DE) 73
S16 Measurement of signal, background, background components, and noise for protein target GFAP using HCR 2°IHC in FFPE mouse brain sections (cf. Figures 3DE) 74
S17 Measurement of signal, background, background components, and noise for protein target PVALB using HCR 2°IHC in FFPE mouse brain sections (cf. Figures 3DE) 75
S18 Measurement of signal, background, background components, and noise for protein target MBP using HCR 2°IHC in FFPE mouse brain sections (cf. Figures 3DE) 76
S19 Measurement of signal and background for protein imaging using HCR 2°IHC in whole-mount zebrafish embryos (cf. Figure 3) 79
S20 Measurement of HCR amplification gain (mean polymer length) for HCR 1°ICC in mammalian cells on a slide (cf. Figure 2C) 81
S21 Measurement of HCR amplification gain (mean polymer length) for HCR 2°ICC in mammalian cells on a slide (cf. Figure 3C) 83
S22 Measurement of HCR amplification gain (mean polymer length) for HCR 1°IHC in FFPE mouse brain sections (cf. Figures 2DE) 85
S23 Measurement of HCR amplification gain (mean polymer length) for HCR 2°IHC in FFPE mouse brain sections (cf. Figure 3DE) 86
S24 Comparison of fluorescence intensity distributions for one-target and two-target experiments 88
S25 Characterizing signal plus background for SC35 and PCNA in a 2-target experiment. 89
S26 Characterizing signal plus background for SC35 in a 1-target experiment 90
S27 Characterizing signal plus background for PCNA in a 1-target experiment 91
S28 Characterizing background for SC35 and PCNA 92
S29 Redundant 2-channel detection of target protein TH in FFPE mouse brain sections (cf. Figure 4) . . 94
S30 Redundant 2-channel detection of target protein KRT17 in FFPE human breast tissue sections (cf. Figure 4) 97
S31 Redundant 2-channel detection of target protein KRT19 in FFPE human breast tissue sections (cf. Figure 4) 98
S32 Measurement of signal and background for redundant 2-channel detection of target proteins KRT17 and KRT19 in FFPE human breast tissue sections (cf. Figure 4) 99
S33 Replicates for 4-plex simultaneous protein and mRNA imaging using HCR 1°ICC and HCR RNA-ISH in mammalian cells on slides (cf. Figures 5B) 102
S34 Measurement of signal and background for 4-plex simultaneous protein and mRNA imaging using HCR 1°ICC and HCR RNA-ISH in mammalian cells on a slide (cf. Figure 5B) 104
S35 Replicates for 4-plex simultaneous protein and mRNA imaging using HCR 1°IHC and HCR RNA-ISH in FFPE mouse brain sections (cf. Figures 5CD) 106

S36 Measurement of signal, background, and noise for 4-plex simultaneous protein and mRNA imaging using HCR 1°IHC and HCR RNA-ISH in FFPE mouse brain sections (cf. Figure 5CD) 107
S37 Measurement of signal and background for target proteins using HCR 1°ICC with or without HCR RNA-ISH in mammalian cells on a slide 111
S38 Measurement of signal and background for target RNAs using HCR RNA-ISH with or without HCR 1°ICC in mammalian cells on a slide 112
S39 Measurement of signal and background for target proteins using HCR 1°IHC with or without HCR RNA-ISH in FFPE mouse brain sections 115
S40 Measurement of signal and background for target RNAs using HCR RNA-ISH with or without HCR 1°IHC in FFPE mouse brain sections 116
S41 Replicates for 4-plex simultaneous protein and mRNA imaging using HCR 2°ICC and HCR RNA-ISH in mammalian cells on slides (cf. Figures 6B) 119
S42 Measurement of signal and background for 4-plex simultaneous protein and mRNA imaging using HCR 2°ICC and HCR RNA-ISH in mammalian cells on a slide (cf. Figure 6B) 121
S43 Replicates for 4-plex simultaneous protein and mRNA imaging using HCR 2°IHC and HCR RNA-ISH in FFPE mouse brain sections (cf. Figures 6CD) 123
S44 Measurement of signal, background, and noise for 4-plex simultaneous protein and mRNA imaging using HCR 2°IHC and HCR RNA-ISH in FFPE mouse brain sections (cf. Figure 6CD) 124
S45 Measurement of signal and background for target proteins using HCR 2°ICC with or without HCR RNA-ISH in mammalian cells on a slide 128
S46 Measurement of signal and background for target RNAs using HCR RNA-ISH with or without HCR 2°ICC in mammalian cells on a slide 129
S47 Measurement of signal and background for target proteins using HCR 2°IHC with or without HCR RNA-ISH in FFPE mouse brain sections 132
S48 Measurement of signal and background for target RNAs using HCR RNA-ISH with or without HCR 2°IHC in FFPE mouse brain sections 133

List of Tables

S1 Examples of HCR RNA-ISH in diverse organisms and sample types. 8
S2 Organism, sample type, target protein, 1°Ab probe details, HCR amplifier details, and figure numbers for HCR 1°IHC 9
S3 Organism, sample type, target protein, 1°Ab probe details, 2°Ab probe details, HCR amplifier details, and figure numbers for HCR 2°IHC 10
S4 Organism, sample type, target RNA, probe set details, HCR amplifier details, and figure numbers for HCR RNA-ISH 11
S5 Confocal microscope settings 12
S6 Epifluorescence microscope settings 13
S7 Experiment types for HCR IHC using initiator-labeled primary antibody probes 18
S8 Experiment types for HCR IHC using unlabeled primary antibody probes and initiator-labeled secondary antibody probes 18
S9 Signal-to-background summary for protein imaging using HCR 1°IHC or HCR 2°IHC and for simultaneous protein and RNA imaging using HCR 1°IHC + HCR RNA-ISH or HCR 2°IHC + HCR RNA-ISH 50
S10 Signal-to-background summary for protein imaging using HCR 1°IHC or HCR 2°IHC in mammalian cells on a slide, FFPE mouse brain sections, FFPE human breast sections, and whole-mount zebrafish embryos 51
S11 Signal-to-background summary for simultaneous protein and RNA imaging using HCR 1°IHC + HCR RNA-ISH or HCR 2°IHC + HCR RNA-ISH in mammalian cells on a slide or FFPE mouse brain sections 51

Development • Supplementary information

S12 Estimated signal-to-background and background components for 3-plex protein imaging using HCR 1°ICC in mammalian cells on a slide (cf. Figure 2C) 57
S13 Estimated signal-to-background, background components, and noise for 4-plex protein imaging using HCR 1°IHC in FFPE mouse brain sections (cf. Figures 2DE) 64
S14 Estimated signal-to-background and background components for 3-plex protein imaging using HCR 2°ICC in mammalian cells on a slide (cf. Figure 3C) 70
S15 Estimated signal-to-background, background components, and noise for 4-plex protein imaging using HCR 2°IHC in FFPE mouse brain sections (cf. Figures 3DE) 77
S16 Estimated signal-to-background for protein imaging using HCR 2°IHC in whole-mount zebrafish embryos (cf. Figure 3) 79
S17 Estimates of HCR amplification gain (mean polymer length) in the context of HCR 1°IHC and HCR 2°IHC in mammalian cells on a slide and FFPE mouse brain sections 80
S18 Estimate of HCR amplification gain (mean polymer length) for HCR 1°ICC in mammalian cells on a slide (cf. Figure 2C) 82
S19 Estimate of HCR amplification gain (mean polymer length) for HCR 2°ICC in mammalian cells on a slide (cf. Figure 3C) 84
S20 Estimate of HCR amplification gain (mean polymer length) for HCR 1°IHC in FFPE mouse brain sections (cf. Figure 2DE) 85
S21 Estimate of HCR amplification gain (mean polymer length) for HCR 2°IHC in FFPE mouse brain sections (cf. Figure 3DE) 86
S22 Estimated signal-to-background for redundant 2-channel detection of target protein TH in FFPE mouse brain sections (cf. Figure 4) 95
S23 BOT and TOP values used to calculate normalized voxel intensities for scatter plots of Figures 4C, S30C, and S31C using methods of Section S2.6.5. 98
S24 Estimated signal-to-background for redundant 2-channel detection of target proteins KRT17 and KRT19 in FFPE human breast tissue sections (cf. Figure 4) 100
S25 Estimated signal-to-background for 4-plex simultaneous protein and mRNA imaging using HCR 1°ICC and HCR RNA-ISH in mammalian cells on a slide (cf. Figure 5B) 103
S26 Estimated signal-to-background for 4-plex simultaneous protein and mRNA imaging using HCR 1°IHC and HCR RNA-ISH in FFPE mouse brain sections (cf. Figure 5CD) 108
S27 Summary of signal, background, and signal-to-background for protein imaging using HCR 1°IHC, RNA imaging using HCR RNA-ISH, or both (cf. Figure 5) 109
S28 Estimated signal, background, and signal-to-background for protein imaging using HCR 1°ICC, RNA imaging using HCR RNA-ISH, or both in mammalian cells on a slide (cf. Figure 5B) 113
S29 Estimated signal, background, and signal-to-background for protein imaging using HCR 1°IHC, RNA imaging using HCR RNA-ISH, or both in FFPE mouse brain sections (cf. Figure 5CD) 117
S30 Estimated signal-to-background for 4-plex simultaneous protein and mRNA imaging using HCR 2°ICC and HCR RNA-ISH in mammalian cells on a slide (cf. Figure 6B) 120
S31 Estimated signal-to-background for 4-plex simultaneous protein and mRNA imaging using HCR 2°IHC and HCR RNA-ISH in FFPE mouse brain sections (cf. Figure 6CD) 125
S32 Summary of signal, background, and signal-to-background for protein imaging using HCR 2°IHC, RNA imaging using HCR RNA-ISH, or both (cf. Figure 6) 126
S33 Estimated signal, background, and signal-to-background for protein imaging using HCR 2°ICC, RNA imaging using HCR RNA-ISH, or both in mammalian cells on a slide (cf. Figure 6B) 130
S34 Estimated signal, background, and signal-to-background for protein imaging using HCR 2°IHC, RNA imaging using HCR RNA-ISH, or both in FFPE mouse brain sections (cf. Figure 6CD) 134

S1 HCR RNA-ISH in diverse organisms and sample types

Organism	Sample types
Human	FFPE thin breast tissue sections (Choi *et al.*, 2016), FFPE thin brain tissue sections (Glineburg *et al.*, 2021), cultured cells on a slide (Choi *et al.*, 2018; Nandagopal *et al.*, 2019; Emert *et al.*, 2021; Glineburg *et al.*, 2021; Grancharova *et al.*, 2021), cultured cell flow cytometry (Choi *et al.*, 2018; Gasperini *et al.*, 2019), thin brain tissue sections (Kamermans *et al.*, 2019), thin intestine tissue sections (May-Zhang *et al.*, 2021), expanded cultured cells on a slide (Alon *et al.*, 2021), thick brain tissue sections (Kumar *et al.*, 2021)
Marmoset	thick brain tissue sections (Krienen *et al.*, 2020)
Rat	thin rat brain tissue sections (Sui *et al.*, 2016), thick rat brain tissue sections (Chen *et al.*, 2021)
Mouse	whole-mount embryos (Huss *et al.*, 2015; Choi *et al.*, 2016; Anderson *et al.*, 2020), cleared thick brain tissue sections (Shah *et al.*, 2016b; Sylwestrak *et al.*, 2016; Park *et al.*, 2018; Kramer *et al.*, 2018; Kahan *et al.*, 2021; Kumar *et al.*, 2021; Mich *et al.*, 2021), thin brain tissue sections (Shah *et al.*, 2016a; Askary *et al.*, 2020; Ren *et al.*, 2019; Carriere *et al.*, 2020; Young & Song, 2020; Cleary *et al.*, 2021; Mayerl *et al.*, 2021; Mu *et al.*, 2021), thin nose tissue sections (Baxter *et al.*, 2021), cultured cells on a slide (Shah *et al.*, 2016b; Nandagopal *et al.*, 2019; Rodriguez *et al.*, 2020; Alon *et al.*, 2021; Glineburg *et al.*, 2021), expanded thick brain tissue sections (Chen *et al.*, 2016; Arshadi *et al.*, 2021; Alon *et al.*, 2021), thick brain tissue sections (Patriarchi *et al.*, 2018; Krienen *et al.*, 2020; Chen *et al.*, 2021; Michael *et al.*, 2020), whole-mount retina (Anderson *et al.*, 2019), thin spinal cord tissue sections (Li *et al.*, 2020), thin intestine tissue sections (May-Zhang *et al.*, 2021), thin retina tissue sections (Zhuang *et al.*, 2020), thin whole-embryo tissue sections (Liu *et al.*, 2020), thin trigeminal ganglia tissue sections (von Buchholtz *et al.*, 2020), gastruloids (van den Brink *et al.*, 2020), expanded thin brain tissue sections (Alon *et al.*, 2021), thin FFPE liver tissue sections (Wells *et al.*, 2021), whole-mount mouse cochlea (Diaz & Heller, 2021)
Zebrafish	whole-mount embryos (Choi *et al.*, 2010; Choi *et al.*, 2014; Shah *et al.*, 2016b; Trivedi *et al.*, 2018; Ton *et al.*, 2018; Meinecke *et al.*, 2018; Gallagher *et al.*, 2017; Tsai *et al.*, 2020; Cayuso *et al.*, 2019; Wang *et al.*, 2019; Kinney *et al.*, 2020; Howard *et al.*, 2021; Pond *et al.*, 2021; Bruce *et al.*, 2021), whole-mount larvae (Choi *et al.*, 2016; Andalman *et al.*, 2019; Callahan *et al.*, 2019; Lovett-Barron *et al.*, 2020; O'Brown *et al.*, 2019; Weinberger *et al.*, 2020; Wurster *et al.*, 2021; Jimenez *et al.*, 2021) thin brain tissue sections (O'Brown *et al.*, 2019), FFPE thin heart tissue sections (Simões *et al.*, 2020), whole-mount embryo tails (Thomson *et al.*, 2021)
Chicken	whole-mount embryos (McLennan *et al.*, 2015; Choi *et al.*, 2016; Lignell *et al.*, 2017; Choi *et al.*, 2018; Ling & Sauka-Spengler, 2019; Williams *et al.*, 2019; Gandhi *et al.*, 2020; Gandhi *et al.*, 2021; Mantri *et al.*, 2021), thin whole-embryo tissue sections (Askary *et al.*, 2020; Mantri *et al.*, 2021), thin heart tissue sections (Mantri *et al.*, 2021), thick cochlea tissue sections (Benkafadar *et al.*, 2021; Janesick *et al.*, 2021)
Quail	whole-mount embryos (Huss *et al.*, 2019)
Xenopus	whole-mount tadpole tails and limbs (Aztekin *et al.*, 2021)
Sea urchin	whole-mount embryos (Choi *et al.*, 2016)
Octopus	thin FFPE transversal sections (Deryckere *et al.*, 2021)
Axolotl	thin tail tissue sections (Freitas *et al.*, 2019), thin FFPE lung tissue sections (Jensen *et al.*, 2021), limb buds (Schloissnig *et al.*, 2021)
Little skate	thin FFPE tissue sections (Marconi *et al.*, 2020; Criswell & Gillis, 2020)
Sea lamprey	whole-mount embryos (Hockman *et al.*, 2019)
Fruit fly	whole-mount embryos (Choi *et al.*, 2016; Domsch *et al.*, 2021; Bruce *et al.*, 2021), whole-mount imaginal discs (Bruce *et al.*, 2021),whole-mount larvae (Ali *et al.*, 2019), whole-mount brains (Lacin *et al.*, 2019; Michki *et al.*, 2021), whole-mount ovaries (Tu *et al.*, 2021)
Beetle	whole-mount embryos (Bruce & Patel, 2020; Bruce *et al.*, 2021; Tidswell *et al.*, 2021), whole-mount heads (Crabtree *et al.*, 2020), whole-mount genitalia (Crabtree *et al.*, 2020), whole-mount ovaries (Tidswell *et al.*, 2021), thin thoracic tissue sections (Hu *et al.*, 2019)
Deep-sea anemone	thin tissue sections (Goffredi *et al.*, 2021)
Killifish	thin coronal sections (van Houcke *et al.*, 2021)
Brine shrimp	naupili and adults (Bruce *et al.*, 2021)
Amphipod crustacean	whole-mount embryos (Bruce & Patel, 2020; Bruce *et al.*, 2021)

Development • Supplementary information

Organism	Sample types
Butterfly	whole-mount embryos and imaginal discs (Bruce *et al.*, 2021)
Moth	whole-mount pupal wings (Bruce *et al.*, 2021)
Tarantula	whole-mount embryos (Bruce *et al.*, 2021)
Water flea	whole-mount embryos (Bruce *et al.*, 2021)
Basal chordate	whole-mount (Kourakis *et al.*, 2019)
Lancelet	whole-mount embryos (Herrera-Úbeda *et al.*, 2019)
Blood fluke	whole-mount (Diaz Soria *et al.*, 2020)
Daddy long legs	whole-mount embryos (Gainett *et al.*, 2021)
Nematode	whole-mount larvae (Choi *et al.*, 2016)
Bacteria	bacteria on termite gut protozoa (Rosenthal *et al.*, 2013), bacteria in environmental samples (Yamaguchi *et al.*, 2015), cultured bacteria on a slide (Choi *et al.*, 2016), bacteria in cleared sputum (DePas *et al.*, 2016), cultured bacterial flow cytometry (Choi *et al.*, 2018), aggregates (Jorth *et al.*, 2019), bacterial symbionts within whole-mount juvenile squid light organ (Bennett *et al.*, 2020; Moriano-Gutierrez *et al.*, 2020), bacteria on cleared plant roots (Dar *et al.*, 2020), bacteria in cleared whole-mount mouse intestines (Gallego-Hernandez *et al.*, 2020), symbionts of the giant tube worms (Hinzke *et al.*, 2021)
Viruses	SARS-CoV-2 in human airway epithelial cultures (Milewska *et al.*, 2020)
Multi-kingdom	whole-mount juvenile squid light organ and bacterial symbionts (Nikolakakis *et al.*, 2015), consortia of archaea and bacteria (Metcalfe *et al.*, 2021)

Table S1: Examples of HCR RNA-ISH in diverse organisms and sample types. Tissue sections are classified as "thick" for thickness $\geq 50\ \mu$m and "thin" otherwise.

Development • Supplementary information

S2 Additional materials and methods

S2.1 Probe and amplifier details for protein targets using HCR 1°IHC

Species	Sample	Protein target	1° Ab probe (initiator-labeled)	Working concentration (μg/mL)	Supplier (catalog #)	HCR amplifier	Figures
H. sapiens sapiens	HeLa cells	HSP60	1°mAb rabbit IgG anti-HSP60	1	Ab (ab224528)	B3-Alexa488	2C, S1, S2
	HeLa cells	GM130	1°mAb rabbit IgG anti-GM130	1	Ab (ab215966)	B2-Alexa647	2C, S1, S3
	HeLa cells	SC35	1°mAb mouse IgG1 anti-SC35	1	Ab (ab11826)	B4-Alexa546	2C, S1, S4
	HeLa cells	HSP60	1°mAb rabbit IgG anti-HSP60	1	Ab (ab224528)	B3-Alexa546	5B, S33, S34, S37
	HeLa cells	PCNA	1°mAb mouse IgG2a anti-PCNA	1	Ab (ab264494)	B5-Alexa488	5B, S33, S34
	HeLa cells	PCNA	1°mAb mouse IgG2a anti-PCNA	1	Ab (ab264494)	B5-Alexa647	S20, S37
M. musculus	brain section	TH	1°mAb rabbit IgG anti-TH	1.4	Ab (ab219729)	B1-Alexa488	2DE, S5, S6
	brain section	GFAP	1°mAb rabbit IgG anti-GFAP	1.9	Ab (ab223127)	B3-Alexa546	2DE, S5, S7
	brain section	MBP	1°mAb rabbit IgG anti-MBP	0.4	Ab (ab230378)	B5-Alexa546	5CD, S35, S36, S39
	brain section	MBP	1°mAb rabbit IgG anti-MBP	0.4	Ab (ab230378)	B5-Alexa647	2DE, S5, S8
	brain section	MAP2	1°mAb rabbit IgG anti-MAP2	1	Ab (ab236033)	B4-Alexa750	2DE, S5, S9
	brain section	TH	1°mAb rabbit IgG anti-TH	0.2	Ab (ab219729)	B1-Alexa647	4B, S29
	brain section	TH	1°mAb rabbit IgG anti-TH	0.1	Ab (ab220218)	B3-Alexa750	4B, S29
	brain section	TH	1°mAb rabbit IgG anti-TH	0.2	Ab (ab220218)	B3-Alexa488	5CD, S35, S36
	brain section	TH	1°mAb rabbit IgG anti-TH	0.2	Ab (ab219729)	B1-Alexa488	S39
	brain section	TH	1°mAb rabbit IgG anti-TH	0.1	Ab (ab220218)	B3-Alexa647	S22

Table S2. Organism, sample type, target protein, 1°Ab probe details, HCR amplifier details, and figure numbers for HCR 1°IHC. For HCR 1°IHC, initiator-labeled primary antibody probes, HCR amplifiers, and buffers (antibody buffer, wash buffer) were obtained from Molecular Technologies (MT) within the Beckman Institute at Caltech (HeLa cells) or from Molecular Instruments (MI) (FFPE mouse brain sections). Ab: Abcam.

Development • Supplementary information

S2.2 Probe and amplifier details for protein targets using HCR 2°IHC

Species	Sample	Protein target	1°Ab probe (unlabeled)<br>2°Ab probe (initiator-labeled)	Working concentration (μg/mL)	Supplier (catalog #)	HCR amplifier	Figures
H. sapiens sapiens	HeLa cells	PCNA	1°mAb mouse IgG2a anti-PCNA	0.1	Ab (ab265585)		
			2°pAb goat anti-mouse IgG2a-B5	1	MT (A9112-B5)	B5-Alexa647	3C, S10, S11, S21, S24, S25, S27, S28, S45
	HeLa cells	HSP60	1°mAb rabbit IgG anti-HSP60	2.4	Ab (ab190828)		
			2°pAb donkey anti-rabbit IgG-B3	1	MT (A9230-B3)	B3-Alexa546	3C, S10, S12, S45
	HeLa cells	SC35	1°mAb mouse IgG1 anti-SC35	5	Ab (ab11826)		
			2°pAb goat anti-mouse IgG1-B2	1	MT (A9111-B2)	B2-Alexa488	3C, S10, S13
	HeLa cells	SC35	1°mAb mouse IgG1 anti-SC35	5	Ab (ab11826)		
			2°pAb goat anti-mouse IgG1-B2	1	MT (A9111-B2)	B2-Alexa546	S24–S26, S28
	HeLa cells	PCNA	1°mAb mouse IgG2a anti-PCNA	1.26	CST (2586)		
			2°pAb goat anti-mouse IgG2a-B5	1	MT (A9112-B5)	B5-Alexa488	6B, S41, S42
	HeLa cells	HSP60	1°mAb rabbit IgG anti-HSP60	2.4	Ab (ab190828)		
			2°pAb donkey anti-rabbit IgG-B4	1	MT (A9230-B4)	B4-Alexa546	6B, S41, S42
	FFPE breast section	KRT17	1°pAb rabbit IgG anti-KRT17	20	Ab (ab53707)		
			2°pAb donkey anti-rabbit IgG-B4	1	MT (A9230-B4)	B4-Alexa546	4B, S30, S32
	FFPE breast section	KRT17	1°pAb rabbit IgG anti-KRT17	20	Ab (ab53707)		
			2°pAb donkey anti-rabbit IgG-B3	1	MT (A9230-B3)	B3-Alexa647	4B, S30, S32
	FFPE breast section	KRT19	1°mAb mouse IgG1 anti-KRT19	10	Ab (ab9221)		
			2°pAb goat anti-mouse IgG1-B2	1	MT (A9111-B2)	B2-Alexa546	S31, S32
	FFPE breast section	KRT19	1°mAb mouse IgG1 anti-KRT19	10	Ab (ab9221)		
			2°pAb goat anti-mouse IgG1-B5	1	MT (A9111-B5)	B5-Alexa647	S31, S32
M. musculus	brain section	TH	1°pAb sheep IgG2 anti-TH	0.15	Ab (ab113)		
			2°pAb donkey anti-sheep IgG-B4	4	MI (12-017-01-B4)	B4-Alexa488	3DE, 6CD, S14, S15, S43, S44, S47
	brain section	TH	1°mAb rabbit IgG anti-TH	0.2	Ab (ab220218)		
			2°pAb donkey anti-rabbit IgG-B3	3.4	MI (12-015-01-B3)	B3-Alexa647	S23
	brain section	GFAP	1°pAb chicken IgY anti-GFAP	0.1	TFS (PA1-10004)		
			2°pAb donkey anti-chicken IgG-B1	2.5	MI (12-018-01-B1)	B1-Alexa546	3DE, S14, S16
	brain section	PVALB	1°mAb rabbit IgG anti-PVALB	1.1	Ab (ab243695)		
			2°pAb donkey anti-rabbit IgG-B5	2.5	MI (12-015-01-B5)	B5-Alexa647	3DE, S14, S17
	brain section	MBP	1°mAb rat IgG2a anti-MBP	0.2	Ab (ab7349)		
			2°pAb donkey anti-rat IgG-B3	1.1	MI (12-019-01-B3)	B3-Alexa750	3DE, S14, S18
			2°pAb donkey anti-rat IgG-B3	1.1	MI (12-019-01-B3)	B3-Alexa546	6CD, S43, S44, S47
D. rerio	whole-mount	Elavl3/Elavl4	1°mAb mouse IgG2b anti-Elavl3/Elavl4	5	TFS (A-21271)		
	27 hpf embryo		2°pAb goat anti-mouse IgG2b-B1	1	MT (A9113-B1)	B1-Alexa647	S19

Table S3. Organism, sample type, target protein, 1°Ab probe details, 2°Ab probe details, HCR amplifier details, and figure numbers for HCR 2°IHC. For HCR 2°IHC, initiator-labeled secondary antibody probes, HCR amplifiers, and buffers (antibody buffer, wash buffer) were obtained from Molecular Technologies (MT) within the Beckman Institute at Caltech (HeLa cells, FFPE human breast tissue sections, and whole-mount zebrafish embryos) or from Molecular Instruments (MI) (FFPE mouse brain sections). Ab: Abcam. CST: Cell Signaling Technology. TFS: Thermo Fisher Scientific.

S2.3 Probe and amplifier details for RNA targets using HCR RNA-ISH

Species	Sample	RNA target	Split-initiator probe pairs	Supplier (catalog #)	HCR amplifier	Figures
H. sapiens sapiens	HeLa cells	*ACTB*	10	MT (4226/A506)	B2-Alexa647	5B, S33, S34
	HeLa cells	*ACTB*	10	MT (4226/A506)	B2-Alexa546	S38, S46
	HeLa cells	*U6*	2	MT (4138/E294)	B1-Alexa594	5B, 6B, S33, S34, S41, S42
	HeLa cells	*U6*	2	MT (4138/E294)	B1-Alexa647	S38, S46
	HeLa cells	*HSP60*	18	MT (4069/E216)	B2-Alexa647	6B, S41, S42
M. musculus	brain section	*Prkcd*	31	MI (PRH342)	B2-Alexa647	S40
	brain section	*Prkcd*	31	MI (PRB518)	B1-Alexa647	5CD, 6CD, S35, S36, S43, S44, S48
	brain section	*Slc17a7*	36	MI (PRB315)	B4-Alexa750	S40
	brain section	*Slc17a7*	36	MI (PRF033)	B2-Alexa750	5CD, 6CD, S35, S36, S43, S44, S48

Table S4. Organism, sample type, target RNA, probe set details, HCR amplifier details, and figure numbers for HCR RNA-ISH. For HCR RNA-ISH, HCR probe sets, amplifiers, and buffers (probe hybridization buffer, probe wash buffer, amplification buffer) were obtained from Molecular Technologies (MT) within the Beckman Institute at Caltech (HeLa cells) or from Molecular Instruments (MI) (FFPE mouse brain sections).

Development • Supplementary information

S2.4 Confocal microscope settings

Sample	Target	Microscope	Objective	Fluorophore	Laser (nm)	Beam splitter	Filter (nm)	Pixel size $(x \times y \times z\ \mu m)$	Figures
HeLa cells	HSP60	Zeiss LSM 800	63×	Alexa488	488	MBS 405/488/561/640 (T10/R90)	490–541	0.1981 × 0.1981 × 0.90	2C, S1, S2
	SC35			Alexa546	561	MBS 405/488/561/640 (T10/R90)	565–600	0.1981 × 0.1981 × 0.90	2C, S1, S4
	GM130			Alexa647	640	MBS 405/488/561/640 (T10/R90)	656–700	0.1981 × 0.1981 × 0.90	2C, S1, S3
	—			DAPI	405	MBS 405/488/561/640 (T10/R90)	410–470	0.1981 × 0.1981 × 0.90	2C, S1–S4
	SC35	Zeiss LSM 800	63×	Alexa488	488	MBS 405/488/561/640 (T10/R90)	490–541	0.1415 × 0.1415 × 0.90	3C, S10, S13
	HSP60			Alexa546	561	MBS 405/488/561/640 (T10/R90)	565–600	0.1415 × 0.1415 × 0.90	3C, S10, S12
	PCNA			Alexa647	640	MBS 405/488/561/640 (T10/R90)	656–700	0.1415 × 0.1415 × 0.90	3C, S10, S11
	—			DAPI	405	MBS 405/488/561/640 (T10/R90)	410–470	0.1415 × 0.1415 × 0.90	3C, S10–S13
	PCNA	Zeiss LSM 800	63×	Alexa647	640	MBS 405/488/561/640 (T10/R90)	656–700	0.1981 × 0.1981 × 0.90	S20, S21
	—			DAPI	405	MBS 405/488/561/640 (T10/R90)	410–470	0.1981 × 0.1981 × 0.90	S20, S21
	SC35	Zeiss LSM 800	20×	Alexa546	561	MBS 405/488/561/640 (T10/R90)	564–610	0.312 × 0.312 × 2.5	S24–S28
	PCNA			Alexa647	640	MBS 405/488/561/640 (T10/R90)	656–700	0.312 × 0.312 × 2.5	S24–S28
	—			DAPI	405	MBS 405/488/561/640 (T10/R90)	410–470	0.312 × 0.312 × 2.5	S24–S28
	PCNA	Zeiss LSM 880	63×	Alexa488	488	MBS 488/561/633	499–554	0.1318 × 0.1318 × 0.90	5B, S33, S34
	HSP60			Alexa546	561	MBS 488/561/633	561–589	0.1318 × 0.1318 × 0.90	5B, S33, S34
	U6			Alexa594	594	MBS 458/514/594	615–645	0.1318 × 0.1318 × 0.90	5B, S33, S34
	ACTB			Alexa647	633	MBS 488/561/633	669–696	0.1318 × 0.1318 × 0.90	5B, S33, S34
	—			DAPI	405	MBS 405	410–471	0.1318 × 0.1318 × 0.90	5B, S33, S34
	PCNA	Zeiss LSM 800	63×	Alexa647	640	MBS 405/488/561/640 (T10/R90)	656–700	0.099 × 0.099 × 0.43	S37, S45
	ACTB			Alexa546	561	MBS 405/488/561/640 (T10/R90)	564–610	0.099 × 0.099 × 0.43	S38, S46
	—			DAPI	405	MBS 405/488/561/640 (T10/R90)	410–470	0.099 × 0.099 × 0.43	S37, S38, S45, S46
	HSP60			Alexa546	561	MBS 405/488/561/640 (T10/R90)	564–610	0.1415 × 0.1415 × 0.43	S37, S45
	U6			Alexa647	640	MBS 405/488/561/640 (T10/R90)	656–700	0.1415 × 0.1415 × 0.43	S38, S46
	—			DAPI	405	MBS 405/488/561/640 (T10/R90)	410–470	0.1415 × 0.1415 × 0.43	S37, S38, S45, S46
	PCNA	Zeiss LSM 880	63×	Alexa488	488	MBS 488/561/633	499–554	0.1318 × 0.1318 × 1.0	6B, S41, S42
	HSP60			Alexa546	561	MBS 488/561/633	561–589	0.1318 × 0.1318 × 1.0	6B, S41, S42
	U6			Alexa594	594	MBS 458/514/594	615–645	0.1318 × 0.1318 × 1.0	6B, S41, S42
	HSP60			Alexa647	633	MBS 488/561/633	669–696	0.1318 × 0.1318 × 1.0	6B, S41, S42
	—			DAPI	405	MBS 405	410–471	0.1318 × 0.1318 × 1.0	6B, S41, S42
FFPE human breast section	KRT17	Zeiss LSM 800	20×	Alexa546	561	MBS 405/488/561/640 (T10/R90)	568–615	0.312 × 0.312 × 2.5	4B, S30, S32
	KRT17			Alexa647	640	MBS 405/488/561/640 (T10/R90)	656–700	0.312 × 0.312 × 2.5	4B, S30, S32
	KRT19			Alexa546	561	MBS 405/488/561/640 (T10/R90)	568–615	0.312 × 0.312 × 2.5	S31, S32
	KRT19			Alexa647	640	MBS 405/488/561/640 (T10/R90)	656–700	0.312 × 0.312 × 2.5	S31, S32
Zebrafish embryos	Elavl3/Elavl4	Zeiss LSM 800	20×	Alexa647	640	MBS 405/488/561/640 (T10/R90)	656–700	0.312 × 0.312 × 2.5	S19

Table S5. Confocal microscope settings. Confocal microscopy was performed with a Zeiss LSM 800 inverted confocal microscope or a Zeiss LSM880 with Fast Airyscan upright confocal microscope. Objectives were as follows: Plan-Apochromat 63×/1.4 Oil DIC M27 (Zeiss LSM 800), Plan-Apochromat 20×/0.8 M27 (Zeiss LSM 800), Plan-Apochromat 63×/1.4 Oil DIC (Zeiss LSM 880).

Development • Supplementary information

S2.5 Epifluorescence microscope settings

Sample	Target	Objective	Fluorophore	LED (nm)	Ex/Em Filter	External filter wheel: emission filter center wavelength/bandwidth (nm)	Pixel size ($x \times y$ μm)	LED intensity (%)	Exposure time (ms)	Figures
FFPE mouse brain section	TH	20×	Alexa488	438	DFT51011	510/40	0.3238 × 0.3238	60	150	2DE, S5, S6
	GFAP		Alexa546	555	DFT51011	590/50	0.3238 × 0.3238	15	50	2DE, S5, S7
	MBP		Alexa647	640	DFT51011	700/75	0.3238 × 0.3238	60	200	2DE, S5, S8
	MAP2		Alexa750	730	Y7	NA	0.3238 × 0.3238	60	650	2DE, S5, S9
	—		DAPI	395	DFT51011	440/40	0.3238 × 0.3238	60	50	S6, S7, S8, S9
	TH	10×	Alexa488	438	DFT51011	510/40	0.6451 × 0.6451	60	350	3DE, S14, S15
	GFAP		Alexa546	555	DFT51011	590/50	0.6451 × 0.6451	50	100	3DE, S14, S16
	PVALB		Alexa647	640	DFT51011	700/75	0.6451 × 0.6451	60	250	3DE, S14, S17
	MBP		Alexa750	730	Y7	NA	0.6451 × 0.6451	80	750	3DE, S14, S18
	—		DAPI	395	DFT51011	440/40	0.6451 × 0.6451	60	50	S15, S16, S17, S18
	TH	40×	Alexa647	640	DFT51011	700/75	0.1612 × 0.1612	19	100	4B, S29
	TH		Alexa750	730	Y7	NA	0.1612 × 0.1612	75	650	4B, S29
	TH	40×	Alexa488	438	DFT51011	510/40	0.1619 × 0.1619	60	250	5CD, S35, S36, S39
	MBP		Alexa546	555	DFT51011	590/50	0.1619 × 0.1619	60	250	5CD, S35, S36, S39
	Prkcd		Alexa647	640	DFT51011	700/75	0.1619 × 0.1619	60	550	5CD, S35, S36, S40
	Slc17a7		Alexa750	730	Y7	NA	0.1619 × 0.1619	91	850	5CD, S35, S36, S40
	—		DAPI	395	DFT51011	440/40	0.1619 × 0.1619	60	50	S36, S39, S40
	TH	40×	Alexa488	438	DFT51011	510/40	0.1619 × 0.1619	20	50	6CD, S43, S44, S47
	MBP		Alexa546	555	DFT51011	590/50	0.1619 × 0.1619	10	50	6CD, S43, S44, S47
	Prkcd		Alexa647	640	DFT51011	700/75	0.1619 × 0.1619	60	400	6CD, S43, S44, S48
	Slc17a7		Alexa750	730	Y7	NA	0.1619 × 0.1619	80	950	6CD, S43, S44, S48
	—		DAPI	395	DFT51011	440/40	0.1619 × 0.1619	50	150	S44, S47, S48
	TH	40×	Alexa647	640	DFT51011	700/75	0.1619 × 0.1619	4	100	S23
	TH		Alexa647	640	DFT51011	700/75	0.1619 × 0.1619	60	100	S22
	—		DAPI	395	DFT51011	440/40	0.1619 × 0.1619	60	50	S22, S23

Table S6. Epifluorescence microscope settings. Epifluorescence microscopy was performed with a Leica THUNDER Imager 3D cell culture epifluorescence microscope equipped with a Leica LED8 multi-LED light source and an sCMOS camera (Leica DFC9000 GTC). Objectives were as follows: Leica HC PL APO 20×/0.80, Leica HC PL APO 10×/0.45, Leica HC PL APO 40x/1.30 oil. Section thickness: 5 μm. The DFT51011 filter set comprises: excitation filter with center wavelengths/bandwidths (nm): 391/32, 479/33, 554/24, 638/31; dichroic mirror with wavelengths (nm): 415, 500, 572, 660; emission filter with center wavelengths/bandwidths (nm): 435/30, 519/25, 594/32, 695/58. The DFT51011 filter set is used in conjunction with an external filter wheel as detailed above. The Y7 filter comprises: excitation filter with center wavelength/bandwidth (nm): 710/75; dichroic mirror with wavelength (nm): 750; emission filter with center wavelength/bandwidth (nm): 810/90. Images were acquired without THUNDER computational clearing.

Development • Supplementary information

S2.6 Image analysis

We build on an image analysis framework developed over a series of publications (Choi *et al.*, 2010; Choi *et al.*, 2014; Choi *et al.*, 2016; Choi *et al.*, 2018; Trivedi *et al.*, 2018). For convenience, here we provide a self-contained description of the details relevant to the present work.

S2.6.1 Raw pixel intensities

The total fluorescence within a pixel is a combination of signal, background, and instrument noise. Instrument noise (NOISE) in each channel corresponds to a non-zero reading in the absence of sample.* Fluorescent background (BACK) arises from three sources in each channel:

- autofluorescence (AF): fluorescence inherent to the sample.
- non-specific detection (NSD): probes that bind non-specifically in the sample and subsequently trigger HCR amplification. For experiments that use both primary antibody probes and secondary antibody probes, NSD_{1° arises from non-specific binding of primary antibody probes and NSD_{2° arises from non-specific binding of secondary antibody probes, with NSD = $\text{NSD}_{1^\circ} + \text{NSD}_{2^\circ}$.
- non-specific amplification (NSA): HCR hairpins that bind non-specifically in the sample.

Fluorescent signal (SIG) in each channel corresponds to:

- signal (SIG): probes that bind specifically to the target and subsequently trigger HCR amplification.

For pixel i of replicate sample n, we denote the background

$$X_{n,i}^{\text{BACK}} = X_{n,i}^{\text{NSD}} + X_{n,i}^{\text{NSA}} + X_{n,i}^{\text{AF}}, \tag{S1}$$

the signal:

$$X_{n,i}^{\text{SIG}}, \tag{S2}$$

and the total fluorescence (SIG+BACK+NOISE):

$$X_{n,i}^{\text{SIG+BACK+NOISE}} = X_{n,i}^{\text{SIG}} + X_{n,i}^{\text{BACK}} + X_{n,i}^{\text{NOISE}}. \tag{S3}$$

S2.6.2 Measurement of signal, background, noise, and signal-to-background for HCR 1°IHC, HCR 2°IHC, and HCR RNA-ISH

Noise, background, and signal are characterized differently depending on the sample type and microscope type:

- For mouse brain sections imaged using an epifluorescence microscope, noise (NOISE) is characterized for pixels in a representative rectangular region with no sample, background plus noise (BACK+NOISE) is characterized for pixels in a representative rectangular region of no- or low-expression and the combination of signal plus background plus noise (SIG+BACK+NOISE) is characterized for pixels in a representative rectangular region of high expression. All of these measurements can be made based on an experiment of Type 1 (using the terminology of Tables S7A and S8A) using the full protocol with probes and hairpins.
- For cells on a slide imaged using a confocal microscope, instrument noise is negligible so we use the approximation NOISE $\approx$ 0. Signal plus background (SIG+BACK) is characterized for pixels in a representative rectangular region of high expression using an experiment of Type 1 (Tables S7A and S8A) employing the full protocol with probes and hairpins. For HCR 1°IHC experiments and HCR RNA-ISH experiments, background (BACK) is characterized for pixels in a representative rectangular region of maximum intensity using the standard protocol but omitting probes (experiment of Type 2 in Table S7B), yielding the partial background

*For experiments using an epifluorescence microscope (mouse brain sections), noise is non-negligible and we take it into account in our analyses. For experiments using a confocal microscope (cells on a slide, human breast tissue sections, and whole-mount zebrafish embryos), noise is negligible and we use the approximation NOISE $\approx$ 0.

Development • Supplementary information

estimate BACK ≈ AF+NSA. For HCR 2°IHC experiments, background (BACK) is characterized for pixels in a representative rectangular region of maximum intensity using the standard protocol including secondary antibody probes but omitting primary antibody probes (experiment of Type 4 in Table S8B), yielding the partial background estimate BACK ≈ NSD_{2°+AF+NSA.

- For human breast tissue sections and whole-mount zebrafish embryos imaged using a confocal microscope, instrument noise is negligible so we use the approximation NOISE ≈ 0. Background (BACK) is characterized for pixels in a representative rectangular region of no- or low-expression and the combination of signal plus background (SIG+BACK) is characterized for pixels in a representative rectangular region of high expression. All of these measurements can be made based on an experiment of Type 1 (using the terminology of Tables S7A and S8A) using the full protocol with probes and hairpins.

For the pixels in these regions, we characterize the distribution by plotting an intensity histogram and characterize average performance by calculating the mean pixel intensities

$$\bar{X}_n^{\text{NOISE}}, \qquad \bar{X}_n^{\text{BACK+NOISE}}, \qquad \bar{X}_n^{\text{SIG+BACK+NOISE}}$$

for replicate n. Performance across replicates is characterized by calculating the sample means

$$\bar{X}^{\text{NOISE}}, \qquad \bar{X}^{\text{BACK+NOISE}}, \qquad \bar{X}^{\text{SIG+BACK+NOISE}}$$

and standard error of the mean

$$s_{\bar{X}^{\text{NOISE}}}, \qquad s_{\bar{X}^{\text{BACK+NOISE}}}, \qquad s_{\bar{X}^{\text{SIG+BACK+NOISE}}}.$$

The mean background is estimated as

$$\bar{X}^{\text{BACK}} = \bar{X}^{\text{BACK+NOISE}} - \bar{X}^{\text{NOISE}} \tag{S4}$$

with the standard error of the mean estimated via uncertainty propagation as

$$s_{\bar{X}^{\text{BACK}}} \leq \sqrt{(s_{\bar{X}^{\text{BACK+NOISE}}})^2 + (s_{\bar{X}^{\text{NOISE}}})^2}. \tag{S5}$$

The upper bound on estimated standard error holds under the assumption that the correlation between BACK+NOISE and NOISE is non-negative. The mean signal is estimated as

$$\bar{X}^{\text{SIG}} = \bar{X}^{\text{SIG+BACK+NOISE}} - \bar{X}^{\text{BACK+NOISE}} \tag{S6}$$

with the standard error of the mean estimated via uncertainty propagation as

$$s_{\bar{X}^{\text{SIG}}} \leq \sqrt{(s_{\bar{X}^{\text{SIG+BACK+NOISE}}})^2 + (s_{\bar{X}^{\text{BACK+NOISE}}})^2}. \tag{S7}$$

The upper bound on estimated standard error holds under the assumption that the correlation between SIG+BACK+NOISE and BACK+NOISE is non-negative. The signal-to-background ratio is estimated as:

$$\bar{X}^{\text{SIG/BACK}} = \bar{X}^{\text{SIG}} / \bar{X}^{\text{BACK}} \tag{S8}$$

with standard error estimated via uncertainty propagation as

$$s^{\text{SIG/BACK}} \leq \bar{X}^{\text{SIG/BACK}} \sqrt{\left(\frac{s_{\bar{X}^{\text{SIG}}}}{\bar{X}^{\text{SIG}}}\right)^2 + \left(\frac{s_{\bar{X}^{\text{BACK}}}}{\bar{X}^{\text{BACK}}}\right)^2}. \tag{S9}$$

The upper bound on estimated standard error holds under the assumption that the correlation between SIG and BACK is non-negative.

Development • Supplementary information

S2.6.3 Measurement of background components for HCR 1°IHC and HCR 2°IHC

Calculation of the signal-to-background ratio (Section S2.6.2) requires only a Type 1 experiment (using the terminology of Tables S7A and S8A), yielding the values $\bar{X}^{\text{SIG+BACK+NOISE}}$, $\bar{X}^{\text{BACK+NOISE}}$, and $\bar{X}^{\text{NOISE}}$ that are needed to calculate SIG/BACK. If desired, additional control experiments that omit certain reagents can be used to characterize the individual components of background (AF, NSA, NSD). A Type 2 experiment (no probes, hairpins only) yields $\bar{X}^{\text{NSA+AF+NOISE}}$ and a Type 3 experiment (no probes, no hairpins) yields $\bar{X}^{\text{AF+NOISE}}$ (using the terminology of Tables S7B and S8B). The background components can then be estimated via calculations analogous to (S6) and (S7). The estimated means are:

$$\bar{X}^{\text{NSD}} = \bar{X}^{\text{BACK+NOISE}} - \bar{X}^{\text{NSA+AF+NOISE}} \tag{S10}$$

$$\bar{X}^{\text{NSA}} = \bar{X}^{\text{NSA+AF+NOISE}} - \bar{X}^{\text{AF+NOISE}} \tag{S11}$$

$$\bar{X}^{\text{AF}} = \bar{X}^{\text{AF+NOISE}} - \bar{X}^{\text{NOISE}} \tag{S12}$$

with estimated standard error of the means are:

$$s_{\bar{X}^{\text{NSD}}} \leq \sqrt{(s_{\bar{X}^{\text{BACK+NOISE}}})^2 + (s_{\bar{X}^{\text{NSA+AF+NOISE}}})^2} \tag{S13}$$

$$s_{\bar{X}^{\text{NSA}}} \leq \sqrt{(s_{\bar{X}^{\text{NSA+AF+NOISE}}})^2 + (s_{\bar{X}^{\text{AF+NOISE}}})^2} \tag{S14}$$

$$s_{\bar{X}^{\text{AF}}} \leq \sqrt{(s_{\bar{X}^{\text{AF+NOISE}}})^2 + (s_{\bar{X}^{\text{NOISE}}})^2}. \tag{S15}$$

These upper bounds on estimated standard errors hold under the assumption that the correlations are non-negative for the components being subtracted in the calculation of the mean.

For HCR 1°IHC experiments that employ initiator-labeled primary antibody probes (and no secondary antibody probes), we have by construction $X^{\text{NSD}_{2^\circ}} = 0$ and $X^{\text{NSD}_{1^\circ}} = X^{\text{NSD}}$, with all of the NSD background attributable to primary antibody probes.

For HCR 2°IHC experiments that employ unlabeled primary antibodies and initiator-labeled secondary antibody probes, NSD background arises from both primary and secondary antibody probes:

$$\bar{X}^{\text{NSD}} = \bar{X}^{\text{NSD}_{1^\circ}} + \bar{X}^{\text{NSD}_{2^\circ}}. \tag{S16}$$

A Type 4 experiment (Table S8B; no primary antibody probes, with initiator-labeled secondary antibody probes, with hairpins) yields $\bar{X}^{\text{NSD}_{2^\circ}\text{+NSA+AF+NOISE}}$. The estimated mean for NSD_{2° is:

$$\bar{X}^{\text{NSD}_{2^\circ}} = \bar{X}^{\text{NSD}_{2^\circ}\text{+NSA+AF+NOISE}} - \bar{X}^{\text{NSA+AF+NOISE}} \tag{S17}$$

with estimated standard error of the mean:

$$s_{\bar{X}^{\text{NSD}_{2^\circ}}} \leq \sqrt{(s_{\bar{X}^{\text{NSD}_{2^\circ}\text{+NSA+AF+NOISE}}})^2 + (s_{\bar{X}^{\text{NSA+AF+NOISE}}})^2} \tag{S18}$$

The estimated mean for NSD_{1° is then:

$$\bar{X}^{\text{NSD}_{1^\circ}} = \bar{X}^{\text{NSD+NSA+AF+NOISE}} - \bar{X}^{\text{NSD}_{2^\circ}\text{+NSA+AF+NOISE}} \tag{S19}$$

with estimated standard error of the mean:

$$s_{\bar{X}^{\text{NSD}_{1^\circ}}} \leq \sqrt{(s_{\bar{X}^{\text{NSD+NSA+AF+NOISE}}})^2 + (s_{\bar{X}^{\text{NSD}_{2^\circ}\text{+NSA+AF+NOISE}}})^2}. \tag{S20}$$

For a given quantity, if $\bar{X} < s_{\bar{X}}$, we instead report $\max(\bar{X}, 0) + s_{\bar{X}}$ as an estimated upper bound, and use this bound for uncertainty propagation.

If a Type 1 experiment demonstrates SIG $\gg$ BACK, as is typically the case using HCR imaging, then there is little motivation to perform the other experiment Types to characterize the individual background components (AF, NSA, NSD) as these are all bounded above by BACK.

Development • Supplementary information

S2.6.4 Measurement of HCR amplification gain (i.e., amplification polymer length)

To estimate HCR amplification gain (corresponding to the number of HCR hairpins per amplification polymer), an additional experiment type can be performed using h1 hairpins only (Type 4 in Table S7C; Type 5 in S8C) to yield $\bar{X}^{\mathrm{SIG_{h1}+BACK+NOISE}}$. HCR polymerization cannot proceed without hairpin h2 so each HCR initiator can tether only a single fluorescent h1 hairpin, corresponding to unamplified signal $\mathrm{SIG_{h1}}$. The mean $\mathrm{SIG_{h1}}$ is estimated as:

$$\bar{X}^{\mathrm{SIG_{h1}}} = \bar{X}^{\mathrm{SIG_{h1}+BACK+NOISE}} - \bar{X}^{\mathrm{BACK+NOISE}} \tag{S21}$$

with estimated standard error of the mean:

$$s_{\bar{X}^{\mathrm{SIG_{h1}}}} \leq \sqrt{(s_{\bar{X}^{\mathrm{SIG_{h1}+BACK+NOISE}}})^2 + (s_{\bar{X}^{\mathrm{BACK+NOISE}}})^2} \tag{S22}$$

The upper bound on estimated standard error holds under the assumption that the correlation between $\mathrm{SIG_{h1}}$+ BACK+NOISE and BACK+NOISE is non-negative. The ratio of amplified to unamplified signal provides an estimate of mean HCR polymer length:

$$\bar{X}^{\mathrm{SIG/SIG_{h1}}} = \bar{X}^{\mathrm{SIG}} / \bar{X}^{\mathrm{SIG_{h1}}} \tag{S23}$$

with standard error estimated via uncertainty propagation as

$$s^{\mathrm{SIG/SIG_{h1}}} \leq \bar{X}^{\mathrm{SIG/SIG_{h1}}} \sqrt{\left(\frac{s_{\bar{X}^{\mathrm{SIG}}}}{\bar{X}^{\mathrm{SIG}}}\right)^2 + \left(\frac{s_{\bar{X}^{\mathrm{SIG_{h1}}}}}{\bar{X}^{\mathrm{SIG_{h1}}}}\right)^2}. \tag{S24}$$

The upper bound on estimated standard error holds under the assumption that the correlation between SIG and $\mathrm{SIG_{h1}}$ is non-negative.

Development • Supplementary information

	Experiment type	Quantity	Reagents		Expression region
			1° Ab-init	Hairpins	in tissue
**A**	1	SIG+NSD+NSA+AF+NOISE = SIG+BACK+NOISE	✓	✓	high
	1	NSD+NSA+AF+NOISE = BACK+NOISE	✓	✓	no/low
	1	NOISE	✓	✓	no sample
**B**	2	NSA+AF+NOISE		✓	high
	3	AF+NOISE			high
**C**	4	SIG_{h1}+NSD+NSA+AF+NOISE = SIG_{h1}+BACK+NOISE	✓	h1 only	high

Table S7. Experiment types for HCR 1°IHC using initiator-labeled primary antibody probes. (A) Characterize signal, background, noise, and signal-to-background. (B) Characterize components of background (AF, NSA, NSD). (C) Characterize unamplified signal and polymer length.

	Experiment type	Quantity	Reagents			Expression region
			1° Ab	2° Ab-init	Hairpins	in tissue
**A**	1	SIG+NSD+NSA+AF+NOISE = SIG+BACK+NOISE	✓	✓	✓	high
	1	NSD+NSA+AF+NOISE = BACK+NOISE	✓	✓	✓	no/low
	1	NOISE	✓	✓	✓	no sample
**B**	2	NSA+AF+NOISE			✓	high
	3	AF+NOISE				high
	4	NSD_{2°+NSA+AF+NOISE		✓	✓	high
**C**	5	SIG_{h1}+NSD+NSA+AF+NOISE = SIG_{h1}+BACK+NOISE	✓	✓	h1 only	high

Table S8. Experiment types for HCR 2°IHC using unlabeled primary antibody probes and initiator-labeled secondary antibody probes. (A) Characterize signal, background, noise, and signal-to-background. (B) Characterize components of background (AF, NSA, NSD_{1°, NSD_{2°, NSD). (C) Characterize unamplified signal and polymer length.

Development • Supplementary information

S2.6.5 Normalized voxel intensities for qHCR imaging: protein relative quantitation with subcellular resolution in an anatomical context

For quantitative imaging using in situ HCR, precision increases with voxel size as long as the imaging voxels remain smaller than the features in the expression pattern (see Section S2.2 of (Trivedi *et al.*, 2018)). To increase precision, we calculate raw voxel intensities by averaging neighboring pixel intensities while still maintaining a subcellcular voxel size. To facilitate relative quantitation between voxels, we estimate the normalized HCR signal of voxel j in replicate n as:

$$x_{n,j} \equiv \frac{X_{n,j}^{\text{SIG+BACK+NOISE}} - X^{\text{BOT}}}{X^{\text{TOP}} - X^{\text{BOT}}}, \tag{S25}$$

which translates and rescales the data so that the voxel intensities in each channel fall in the interval [0,1]. Here,

$$X^{\text{BOT}} \equiv \bar{X}^{\text{BACK+NOISE}} \tag{S26}$$

is the mean background plus noise across replicates (see Section S2.6.2) and

$$X^{\text{TOP}} \equiv \max_{n,j} X_{n,j}^{\text{SIG+BACK+NOISE}} \tag{S27}$$

is the maximum total fluorescence for a voxel across replicates.

Pairwise expression scatter plots that each display normalized voxel intensities for two channels (e.g., Figures 4 and 5 of (Trivedi *et al.*, 2018)) provide a powerful quantitative framework for performing multidimensional read-out/read-in analyses (Figure 6 of (Trivedi *et al.*, 2018)). Read-out from anatomical space to expression space enables discovery of expression clusters of voxels with quantitatively related expression levels and ratios (amplitudes and slopes in the expression scatter plots), while read-in from expression space to anatomical space enables discovery of the corresponding anatomical locations of these expression clusters within the sample. The simple and practical normalization approach of (S25)–(S27) translates and rescales all voxels identically within a given channel (enabling comparison of amplitudes and slopes in scatter plots between replicates), and does not attempt to remove scatter in the normalized signal estimate that is caused by scatter in background or noise.

To validate relative protein quantitation with subcellular resolution (2×2 μm voxels) in FFPE mouse brain sections and FFPE human breast tissue sections, Figures 4C, S29C, S30C, and S31C display highly correlated normalized voxel intensities for 2-channel redundant detection of different protein targets. In this setting, accuracy corresponds to linearity with zero intercept, and precision corresponds to scatter around the line (Trivedi *et al.*, 2018).

S3 Protocols for HCR 1°IHC with/without HCR RNA-ISH

S3.1 Protocols for mammalian cells on a chambered slide

S3.1.1 Preparation of fixed mammalian cells on a chambered slide

1. Coat bottom of each chamber by applying 300 μL of 0.01% poly-D-lysine prepared in cell culture grade H_2O.
   NOTE: *A volume of 300 μL is sufficient per chamber on an 8-chamber slide. Scale volume accordingly if using a different slide format.*

2. Incubate for at least 30 min at room temperature.

3. Aspirate the coating solution and wash each chamber twice with molecular biology grade H_2O.

4. Plate desired number of cells in each chamber.

5. Grow cells to desired confluency for 24–48 h.

6. Aspirate growth media and wash each chamber with 300 μL of DPBS.
   NOTE: *avoid using calcium chloride and magnesium chloride in DPBS as this leads to increased autofluorescence.*

7. Add 300 μL of 4% formaldehyde to each chamber.
   CAUTION: *use formaldehyde with extreme care as it is a hazardous material.*

8. Incubate for 10 min at room temperature.

9. Remove fixative and wash each chamber with 2 $\times$ 300 μL of DPBS.

10. Aspirate DPBS and add 300 μL of ice-cold 70% ethanol (EtOH).

11. Permeabilize cells overnight (or longer) at -20 °C.

12. Proceed to HCR assay.

S3.1.2 Multiplexed HCR 1°ICC with/without HCR RNA-ISH using initiator-labeled primary antibody probes for protein targets, split-initiator DNA probes for RNA targets, and simultaneous HCR signal amplification for all targets

Protein detection stage

1. Aspirate EtOH and wash samples 2 × 5 min with 300 μL of 1× PBS.

2. Apply 300 μL antibody buffer to each chamber. Incubate at room temperature for 1 h with gentle agitation.

3. Prepare working concentration of primary antibodies in antibody buffer. Prepare 300 μL per chamber.
NOTE: *follow manufacturer's guidelines for primary antibody working concentration.*

4. Replace antibody solution with primary antibody solution and incubate overnight (>12 h) at 4 °C with gentle agitation.
NOTE: *Incubation may be optimized (e.g., 1–2 h at room temperature) depending on sample type and thickness.*

5. Remove excess antibodies by washing 3 × 5 min with PBST at room temperature with gentle agitation.

6. Proceed to **RNA detection stage** for co-detection of protein and RNA. Otherwise, proceed to **Amplification stage**.

RNA detection stage

1. Post-fix sample with 300 μL of 4% formaldehyde.
CAUTION: *use formaldehyde with extreme care as it is a hazardous material.*

2. Incubate for 10 min at room temperature.

3. Remove fixative and wash each chamber with 2 × 300 μL of PBS.

4. Wash sample with 300 μL of 2× SSC.

5. Pre-hybridize samples in 300 μL of probe hybridization buffer for 30 min at 37 °C.
CAUTION: *Probe hybridization buffer contains formamide, a hazardous material.*
NOTE: *pre-heat probe hybridization buffer to 37 °C before use.*

6. Prepare a 16 nM probe solution by adding 4.8 pmol of each probe mixture (e.g. 4.8 μL of 1 μM stock) to 300 μL of probe hybridization buffer at 37 °C.
NOTE: *This is the amount of probe set needed for each target on a single chamber of an 8-well chambered slide using 300 μL of incubation volume.*

7. Remove the pre-hybridization solution and add the probe solution.

8. Incubate samples overnight (>12 h) at 37 °C.

9. Remove excess probes by washing 4 × 5 min with 300 μL of probe wash buffer at 37 °C.
CAUTION: *Probe wash buffer contains formamide, a hazardous material.*
NOTE: *pre-heat probe wash buffer to 37 °C before use.*

10. Wash with 300 μL 5× SSCT at room temperature for 5 min.

11. Proceed to **Amplification stage**.

Development • Supplementary information

Amplification stage

1. Wash with 300 μL 5$\times$ SSCT at room temperature for 5 min.
2. Pre-amplify samples in 300 μL of amplification buffer for 30 min at room temperature.
   NOTE: *equilibrate amplification buffer to room temperature before use.*
3. Separately prepare 18 pmol of hairpin h1 and 18 pmol of hairpin h2 by snap cooling 6 μL of 3 μM stock (heat at 95 °C for 90 seconds and cool to room temperature in a dark drawer for 30 min).
   NOTE: *HCR hairpins h1 and h2 are provided in hairpin storage buffer ready for snap cooling. h1 and h2 should be snap cooled in separate tubes. This is the amount of hairpins needed for each target in a single sample using 300 μL of incubation volume.*
4. Prepare a 60 nM hairpin solution by adding all snap-cooled h1 hairpins and snap-cooled h2 hairpins to 300 μL of amplification buffer at room temperature per sample.
5. Remove the pre-amplification solution and add the hairpin solution.
6. Incubate the slide overnight (>12 h) in the dark at room temperature.
7. Remove excess hairpins by washing 5 $\times$ 5 min with 300 μL of 5$\times$ SSCT at room temperature.

Sample mounting for microscopy

1. Remove final wash and add 150 μL of mounting medium (e.g., Fluoromount-G with DAPI).
2. Slides can be stored at 4 °C protected from light prior to imaging.
   NOTE: *see Section S2.4 for details of confocal microscopes used to image mammalian cells on a chambered slide.*

S3.1.3 Buffers for HCR 1°ICC with/without HCR RNA-ISH

HCR probes (initiator-labeled antibody probes, split-initiator DNA probes), amplifiers, and buffers (antibody buffer, probe hybridization buffer, probe wash buffer, amplification buffer) are available from Molecular Instruments (`www.molecularinstruments.com`). Probe hybridization buffer, and probe wash buffer should be stored at -20 °C. Antibody buffer and amplification buffer should be stored at 4 °C. Make sure all solutions are well mixed before use.

**1× PBST**	For 40 mL of solution
1× phosphate-buffered saline (PBS)	4 mL of 10× PBS
0.1% Tween 20	400 μL of 10% Tween 20
	Fill up to 40 mL with ultrapure H_2O

**5× SSCT**	For 40 mL of solution
5× saline sodium citrate (SSC)	10 mL of 20× SSC
0.1% Tween 20	400 μL of 10% Tween 20
	Fill up to 40 mL with ultrapure H_2O

S3.1.4 Reagents and supplies

ibidi μ-slide ibitreat (ibidi Cat. # 80826)
Poly-D-lysine hydrobromide (Sigma-Aldrich Cat. # P7280)
Molecular biology grade H_2O (Corning Cat. # 46-000-CV)
DPBS, no calcium, no magnesium (Life Technologies Cat. # 14190144)
Image-iT Fixative Solution 4% (Thermo Fisher Scientific Cat. # FB002)
10× PBS (Ambion Cat. # AM9624)
10% Tween 20 (Teknova Cat. # T0710)
20× saline sodium citrate (SSC) (Life Technologies Cat. # 15557-044)
DAPI Fluoromount-G (SouthernBiotech Cat. # 0100-20)

S3.2 Protocols for FFPE mouse brain tissue sections

S3.2.1 Preparation of formalin-fixed paraffin-embedded (FFPE) mouse brain tissue sections

1. Bake slides in a dry oven for 1 h at 60 °C to improve sample adhesion to the slide.
2. In a fume hood, deparaffinize FFPE tissue by immersing in Pro-Par Clearant for 3 × 5 min. Move slides up and down occasionally.
   CAUTION: *use Pro-Par Clearant with care as it is a hazardous material.*
   NOTE: *Xylene can be used in place of Pro-Par Clearant.*
   NOTE: *Each 50 mL tube can fit two outward-facing slides. A volume of 30 mL is sufficient to immerse sections in the tube. If desired, a larger number of slides can be processed together using a Coplin jar.*
3. Incubate slides in 100% ethanol (EtOH) for 2 × 3 min at room temperature. Move slides up and down occasionally.
4. Rehydrate with a series of graded EtOH washes at room temperature.
   (a) 95% EtOH for 3 min
   (b) 70% EtOH for 3 min
   (c) 50% EtOH for 3 min
   (d) Nanopure water for 3 min
5. Bring 500 mL of 1× citrate buffer (pH 6.0) in a beaker to boil in a microwave.
   NOTE: *1× Tris-EDTA buffer (pH 9.0) can be used in place of citrate buffer (pH 6.0). Optimal antigen retrieval method may differ depending on the antigen/antibody used.*
6. Maintain citrate buffer at 90–95 °C on a hot plate.
7. Immerse slides for 15 min.
   NOTE: *Alternatively, slides may be immersed at 95–99 °C for 15 min in a steamer.*
8. Remove beaker from hot plate and add 100 mL of nanopure water every 5 min to allow temperature to decrease to 45 °C in 20 min.
9. Immerse slides in 400 mL of nanopure water in a separate container for 10 min at room temperature.
10. Immerse slides in 1× PBST for 2 × 2 min at room temperature.
    NOTE: *avoid using calcium chloride and magnesium chloride in PBS as this leads to increased autofluorescence in the tissue.*
11. Drain slide by blotting edges on a Kimwipe.
12. Wipe around the section with a Kimwipe and circle tissue with a hydrophobic pen.
13. Optional: Proceed to autofluorescence bleaching protocol if tissue sample has high autofluorescence. Otherwise, proceed to HCR assay.

S3.2.2 Buffer recipes for sample preparation

1× citrate buffer
1× citrate buffer

For 500 mL of solution
5 mL of 100× citrate buffer (pH 6.0)
Fill up to 500 mL with water

1× Tris-EDTA buffer
1× Tris-EDTA buffer

For 500 mL of solution
5 mL of 100× Tris-EDTA buffer (pH 9.0)
Fill up to 500 mL with water

PBST
1× PBS
0.1% Tween 20

For 50 mL of solution
5 mL of 10× PBS
500 μL of 10% Tween 20
Fill up to 50 mL with ultrapure H_2O

S3.2.3 Autofluorescence bleaching protocol

1. Prepare bleaching solution fresh before use.
   CAUTION: *Keep bleaching solution uncapped inside a fume hood as it produces gas.*

2. Add 200 μL of bleaching solution on top of tissue.

3. Place slide under a 240 W LED light. Keep slide 80 mm away from the light source.
   NOTE: *Perform bleaching inside a refrigerator to avoid overheating of sample.*

4. Expose tissue to maximum LED intensity for 3 h.
   NOTE: *Check slide every hour and re-apply fresh bleaching solution if necessary.*

5. Wash slide 4 $\times$ 10 min in PBST.

6. Proceed to HCR assay.

S3.2.4 Buffer recipes for autofluorescence bleaching protocol

**Bleaching solution**	For 1 mL of solution
4.5% hydrogen peroxide (H_2O_2)	150 μL 30% H_2O_2
24 mM NaOH	4.8 μL of 5 M NaOH
1$\times$ PBS	845.2 μL 1$\times$ PBS

Development • Supplementary information

S3.2.5 Multiplexed HCR 1°IHC with/without HCR RNA-ISH using initiator-labeled primary antibody probes for protein targets, split-initiator DNA probes for RNA targets, and simultaneous HCR signal amplification for all targets

Protein detection stage

1. Block tissue by applying 200 μL of antibody buffer on top of the sample. Incubate at room temperature for 1 h in a humidified chamber.
2. Prepare working concentration of initiator-labeled primary antibodies in antibody buffer. Prepare 100 μL per section.
   NOTE: *follow manufacturer's guidelines for primary antibody working concentration.*
3. Drain slide by blotting edges on a Kimwipe and wipe around the section with another Kimwipe.
4. Add primary antibody solution to each section and incubate overnight (>12 h) at 4 °C in a humidified chamber.
   NOTE: *Incubation may be optimized (e.g., 1–2 h at room temperature) depending on sample type and thickness.*
5. Remove excess antibodies by immersing slide in 1× PBST at room temperature for 3 × 5 min.
6. Proceed to **RNA detection stage** for co-detection of protein and RNA. Otherwise, proceed to **Amplification stage**.

RNA detection stage

1. Drain slide by blotting edges on a Kimwipe and wipe around the section with another Kimwipe.
2. Post-fix sample with 200 μL of 4% formaldehyde on the tissue.
   CAUTION: *use formaldehyde with extreme care as it is a hazardous material.*
3. Incubate slides for 10 min at room temperature.
4. Immerse slides for 2 × 5 min in PBST.
5. Immerse slides for 5 min in 5× SSCT.
6. Pre-warm a humidified chamber to 37 °C.
7. Drain slide by blotting edges on a Kimwipe and wipe around the section with another Kimwipe.
8. Add 200 μL of probe hybridization buffer on top of the tissue sample.
   CAUTION: *Probe hybridization buffer contains formamide, a hazardous material.*
   NOTE: *pre-heat probe hybridization buffer to 37 °C before use.*
9. Pre-hybridize for 10 min inside the humidified chamber.
10. Prepare a 16 nM probe solution by adding 1.6 pmol of each probe set (e.g. 1.6 μL of 1 μM stock) to 100 μL of probe hybridization buffer at 37 °C.
    NOTE: *This is the amount of probe set needed for each target on a single slide using 100 μL of incubation volume.*
11. Remove the pre-hybridization solution and drain excess buffer on slide by blotting edges on a Kimwipe.
12. Add 100 μL of the probe solution on top of the tissue sample.
13. Place a coverslip on the sample and incubate overnight (>12 h) in the 37 °C humidified chamber.
14. Immerse slide in probe wash buffer at 37 °C to float off coverslip.
    CAUTION: *Probe wash buffer contains formamide, a hazardous material.*
15. Remove excess probes by incubating slide at 37 °C in:
    (a) 75% of probe wash buffer / 25% 5× SSCT for 15 min
    (b) 50% of probe wash buffer / 50% 5× SSCT for 15 min
    (c) 25% of probe wash buffer / 75% 5× SSCT for 15 min
    (d) 100% 5× SSCT for 15 min

    NOTE: *Wash solutions should be pre-heated to 37 °C before use.*
16. Proceed to amplification stage.

Development • Supplementary information

Amplification stage

1. Immerse slide in 5× SSCT at room temperature for 5 min.
2. Drain slide by blotting edges on a Kimwipe and wipe around the section with another Kimwipe.
3. Add 200 μL of amplification buffer on top of the tissue sample and pre-amplify in a humidified chamber for 30 min at room temperature.
   NOTE: *equilibrate amplification buffer to room temperature before use.*
4. Separately prepare 6 pmol of hairpin h1 and 6 pmol of hairpin h2 by snap cooling 2 μL of 3 μM stock (heat at 95 °C for 90 seconds and cool to room temperature in a dark drawer for 30 min).
   NOTE: *HCR hairpins h1 and h2 are provided in hairpin storage buffer ready for snap cooling. h1 and h2 should be snap cooled in separate tubes. This is the amount of hairpins needed for each target on a single slide using 100 μL of incubation volume.*
5. Prepare hairpin solution by adding all snap-cooled h1 hairpins and snap-cooled h2 hairpins to 100 μL of amplification buffer at room temperature per section.
6. Remove the pre-amplification solution and drain excess buffer on slide by blotting edges on a Kimwipe.
7. Add 100 μL of the hairpin solution on top of the tissue sample.
8. Incubate overnight (>12 h) in a dark humidified chamber at room temperature.
9. Remove excess hairpins by immersing slide in 5× SSCT at room temperature for:
   (a) 1 × 5 min
   (b) 2 × 15 min
   (c) 1 × 5 min

Sample mounting for microscopy

1. Drain slide by blotting edges on a Kimwipe and dry around the section with another Kimwipe.
2. Apply 35 μL of Slowfade Diamond antifade mountant with DAPI on top of the tissue.
3. Place a 22 × 30 mm No. 1 coverslip on top carefully to prevent air bubbles.
4. Slides can be stored at 4 °C protected from light prior to imaging.
   NOTE: *see Section S2.5 for details of epifluorescence microscope used to image FFPE mouse brain tissue section.*

Development • Supplementary information

S3.2.6 Buffer for HCR 1°IHC with/without HCR RNA-ISH

HCR probes (initiator-labeled antibody probes, split-initiator DNA probes), amplifiers, and buffers (antibody buffer, probe hybridization buffer, probe wash buffer, amplification buffer) are available from Molecular Instruments (`www.molecularinstruments.com`). Probe hybridization buffer, and probe wash buffer should be stored at -20 °C. Antibody buffer and amplification buffer should be stored at 4 °C. Make sure all solutions are well mixed before use.

**5× SSCT**	For 40 mL of solution
5× saline sodium citrate (SSC)	10 mL of 20× SSC
0.1% Tween 20	400 μL of 10% Tween 20
	Fill up to 40 mL with ultrapure H_2O

S3.2.7 Reagents and supplies

Pro-Par Clearant (ANATECH LTD Cat. # 510)
100% Ethanol (EtOH) (VWR Cat. # 89125-172)
100× citrate buffer pH 6.0 (Abcam Cat. #ab93678)
100× Tris-EDTA buffer pH 9.0 (Abcam Cat. #ab93684)
10× Phosphate-buffered saline (PBS) (Invitrogen Cat. #AM9624)
30% hydrogen peroxide (Sigma Aldrich Cat. #H1009)
Sodium hydroxide (Fisher Scientific Cat. #S318-500)
20× saline sodium citrate (SSC) (Life Technologies Cat. # 15557-044)
10% Tween 20 (Teknova Cat. # T0710)
SlowFade Diamond Antifade Mountant with DAPI (Invitrogen Cat. # S36973)
22 mm × 30 mm No. 1 coverslip (VWR Cat. # 48393-026)
6 band 240 W LED vegetative grow light (HTG Supply Cat. # LED-6B240)

Development • Supplementary information

S4 Protocols for HCR 2°IHC with/without HCR RNA-ISH

S4.1 Protocols for mammalian cells on a chambered slide

S4.1.1 Preparation of fixed mammalian cells on a chambered slide

1. Coat bottom of each chamber by applying 300 μL of 0.01% poly-D-lysine prepared in cell culture grade H_2O.
   NOTE: *A volume of 300 μL is sufficient per chamber on an 8-chamber slide. Scale volume accordingly if using a different slide format.*
2. Incubate for at least 30 min at room temperature.
3. Aspirate the coating solution and wash each chamber twice with molecular biology grade H_2O.
4. Plate desired number of cells in each chamber.
5. Grow cells to desired confluency for 24–48 h.
6. Aspirate growth media and wash each chamber with 300 μL of DPBS.
   NOTE: *avoid using calcium chloride and magnesium chloride in DPBS as this leads to increased autofluorescence.*
7. Add 300 μL of 4% formaldehyde to each chamber.
   CAUTION: *use formaldehyde with extreme care as it is a hazardous material.*
8. Incubate for 10 min at room temperature.
9. Remove fixative and wash each chamber with 2 $\times$ 300 μL of DPBS.
10. Aspirate DPBS and add 300 μL of ice-cold 70% ethanol (EtOH).
11. Permeabilize cells overnight (or longer) at -20 °C.
12. Proceed to HCR assay.

Development • Supplementary information

S4.1.2 HCR 2°ICC with/without HCR RNA-ISH using unlabeled primary antibody probes and initiator-labeled secondary antibody probes for protein targets, split-initiator DNA probes for RNA targets, and simultaneous HCR signal amplification for all targets

Protein detection stage

1. Aspirate EtOH from sample and wash samples 2 × 5 min with 300 μL of 1× PBS.

2. Apply 300 μL antibody buffer to each chamber. Incubate at room temperature for 1 hr with gentle agitation.

3. Prepare working concentration of primary antibodies in antibody buffer. Prepare 300 μL per chamber.
NOTE: *follow manufacturer's guidelines for primary antibody working concentration.*

4. Replace antibody buffer with primary antibody solution and incubate overnight (>12 h) at 4 °C with gentle agitation.
NOTE: *Incubation may be optimized (e.g., 1–2 h at room temperature) depending on sample type and thickness.*

5. Remove excess antibodies by washing 3 × 5 min with 1× PBST at room temperature with gentle agitation.

6. Prepare working concentration of initiator-labeled secondary antibodies in antibody buffer. Prepare 300 μL per chamber.
NOTE: *We recommend starting with a 1 µg/mL working concentration.*

7. Add secondary antibody solution to each chamber and incubate 1 h at room temperature with gentle agitation.

8. Remove excess antibodies by washing 3 × 5 min with 1× PBST at room temperature with gentle agitation.

9. Proceed to **RNA detection stage** for co-detection of protein and RNA. Otherwise, proceed to **Amplification stage**.

RNA detection stage

1. Post-fix sample with 300 μL of 4% formaldehyde.
CAUTION: *use formaldehyde with extreme care as it is a hazardous material.*

2. Incubate for 10 min at room temperature.

3. Remove fixative and wash each chamber with 2 × 300 μL of PBS.

4. Wash sample with 300 μL of 2× SSC.

5. Pre-hybridize samples in 300 μL of probe hybridization buffer for 30 min at 37 °C.
CAUTION: *Probe hybridization buffer contains formamide, a hazardous material.*
NOTE: *pre-heat probe hybridization buffer to 37 °C before use.*

6. Prepare a 16 nM probe solution by adding 4.8 pmol of each probe mixture (e.g. 4.8 μL of 1 μM stock) to 300 μL of probe hybridization buffer at 37 °C.
NOTE: *This is the amount of probe set needed for each target on a single chamber of an 8-well chambered slide using 300 µL of incubation volume.*

7. Remove the pre-hybridization solution and add the probe solution.

8. Incubate samples overnight (>12 h) at 37 °C.

9. Remove excess probes by washing 4 × 5 min with 300 μL of probe wash buffer at 37 °C.
CAUTION: *Probe wash buffer contains formamide, a hazardous material.*
NOTE: *pre-heat probe wash buffer to 37 °C before use.*

Development • Supplementary information

10. Wash with 300 μL 5× SSCT at room temperature for 5 min.

11. Proceed to **Amplification stage**.

Amplification stage

1. Wash with 300 μL 5× SSCT at room temperature for 5 min.

2. Pre-amplify samples in 300 μL of amplification buffer for 30 min at room temperature.
   NOTE: *Equilibrate amplification buffer to room temperature before use.*

3. Separately prepare 18 pmol of hairpin h1 and 18 pmol of hairpin h2 by snap cooling 6 μL of 3 μM stock (heat at 95 °C for 90 seconds and cool to room temperature in a dark drawer for 30 min).
   NOTE: *HCR hairpins h1 and h2 are provided in hairpin storage buffer ready for snap cooling. h1 and h2 should be snap cooled in separate tubes. This is the amount of hairpins needed for each target in a single sample using 300 μL of incubation volume.*

4. Prepare a 60 nM hairpin solution by adding all snap-cooled h1 hairpins and snap-cooled h2 hairpins to 300 μL of amplification buffer at room temperature per sample.

5. Remove the pre-amplification solution and add the hairpin solution.

6. Incubate the slide overnight (>12 h) protected from light at room temperature.

7. Remove excess hairpins by washing 5 × 5 min with 300 μL of 5× SSCT at room temperature.

Sample mounting for microscopy

1. Remove final wash and add 150 μL of mounting medium (e.g., Fluoromount-G with DAPI).

2. Slides can be stored at 4 °C protected from light prior to imaging.
   NOTE: *see Section S2.4 for details of confocal microscopes used to image mammalian cells on a chambered slide.*

Development • Supplementary information

S4.1.3 Buffers for HCR 2°ICC with/without HCR RNA-ISH

HCR probes (initiator-labeled antibody probes, split-initiator DNA probes), amplifiers, and buffers (antibody buffer, probe hybridization buffer, probe wash buffer, amplification buffer) are available from Molecular Instruments (`www.molecularinstruments.com`). Probe hybridization buffer and probe wash buffer should be stored at -20 °C. Antibody buffer and amplification buffer should be stored at 4 °C. Make sure all solutions are well mixed before use.

**1× PBST**	For 40 mL of solution
1× phosphate-buffered saline (PBS)	4 mL of 10× PBS
0.1% Tween 20	400 μL of 10% Tween 20
	Fill up to 40 mL with ultrapure H_2O

**5× SSCT**	For 40 mL of solution
5× saline sodium citrate (SSC)	10 mL of 20× SSC
0.1% Tween 20	400 μL of 10% Tween 20
	Fill up to 40 mL with ultrapure H_2O

S4.1.4 Reagents and supplies

ibidi μ-slide ibitreat (ibidi Cat. # 80826)
Poly-D-lysine hydrobromide (Sigma-Aldrich Cat. # P7280)
Molecular biology grade H_2O (Corning Cat. # 46-000-CV)
DPBS, no calcium, no magnesium (Life Technologies Cat. # 14190144)
Image-iT Fixative Solution 4% (Thermo Fisher Scientific Cat. # FB002)
10× PBS (Ambion Cat. # AM9624)
10% Tween 20 (Teknova Cat. # T0710)
20× saline sodium citrate (SSC) (Life Technologies Cat. # 15557-044)
DAPI Fluoromount-G (SouthernBiotech Cat. # 0100-20)

S4.2 Protocols for FFPE mouse brain tissue sections

S4.2.1 Preparation of formalin-fixed paraffin-embedded (FFPE) mouse brain tissue sections

1. Bake slides in a dry oven for 1 h at 60 °C to improve sample adhesion to the slide.
2. In a fume hood, deparaffinize FFPE tissue by immersing in Pro-Par Clearant for 3 × 5 min. Move slides up and down occasionally.
   CAUTION: *use Pro-Par Clearant with care as it is a hazardous material.*
   NOTE: *Xylene can be used in place of Pro-Par Clearant.*
   NOTE: *Each 50 mL tube can fit two outward-facing slides. A volume of 30mL is sufficient to immerse sections in the tube. If desired, a larger number of slides can be processed together using a Coplin jar.*
3. Incubate slides in 100% ethanol (EtOH) for 2 × 3 min at room temperature. Move slides up and down occasionally.
4. Rehydrate with a series of graded EtOH washes at room temperature.
   (a) 95% EtOH for 3 min
   (b) 70% EtOH for 3 min
   (c) 50% EtOH for 3 min
   (d) Nanopure water for 3 min
5. Bring 500 mL of 1× citrate buffer (pH 6.0) in a beaker to boil in a microwave.
   NOTE: *1× Tris-EDTA buffer (pH 9.0) can be used in place of citrate buffer (pH 6.0). Optimal antigen retrieval method may differ depending on the antigen/antibody used.*
6. Maintain citrate buffer at 90–95 °C on a hot plate.
7. Immerse slides for 15 min.
   NOTE: *Alternatively, slides may be immersed at 95–99 °C for 15 min in a steamer.*
8. Remove beaker from hot plate and add 100 mL of nanopure water every 5 min to allow temperature to decrease to 45 °C in 20 min.
9. Immerse slides in 400 mL of nanopure water in a separate container for 10 min at room temperature.
10. Immerse slides in 1× PBST for 2 × 2 min at room temperature.
    NOTE: *avoid using calcium chloride and magnesium chloride in PBS as this leads to increased autofluorescence in the tissue.*
11. Drain slide by blotting edges on a Kimwipe.
12. Wipe around the section with a Kimwipe and circle tissue with a hydrophobic pen.
13. Optional: Proceed to autofluorescence bleaching protocol if tissue sample has high autofluorescence. Otherwise, proceed to HCR assay.

Development • Supplementary information

S4.2.2 Buffer recipes for sample preparation

**1× citrate buffer**	For 500 mL of solution
1× citrate buffer	5 mL of 100× citrate buffer (pH 6.0)
	Fill up to 500 mL with water
**1× Tris-EDTA buffer**	For 500 mL of solution
1× Tris-EDTA buffer	5 mL of 100× Tris-EDTA buffer (pH 9.0)
	Fill up to 500 mL with water
**PBST**	For 50 mL of solution
1× PBS	5 mL of 10× PBS
0.1% Tween 20	500 μL of 10% Tween 20
	Fill up to 50 mL with ultrapure H_2O

Development • Supplementary information

S4.2.3 Autofluorescence bleaching protocol

1. Prepare bleaching solution fresh before use.
   CAUTION: *Keep bleaching solution uncapped inside a fume hood as it produces gas.*

2. Add 200 μL of bleaching solution on top of tissue.

3. Place slide under a 240 W LED light. Keep slide 80 mm away from the light source.
   NOTE: *Perform bleaching inside a refrigerator to avoid overheating of sample.*

4. Expose tissue to maximum LED intensity for 3 h.
   NOTE: *Check slide every hour and re-apply fresh bleaching solution if necessary.*

5. Wash slide 4 $\times$ 10 min in PBST.

6. Proceed to HCR assay.

S4.2.4 Buffer recipes for autofluorescence bleaching protocol

**Bleaching solution**	For 1 mL of solution
4.5% hydrogen peroxide (H_2O_2)	150 μL 30% H_2O_2
24 mM NaOH	4.8 μL of 5 M NaOH
1$\times$ PBS	845.2 μL 1$\times$ PBS

Development • Supplementary information

S4.2.5 Multiplexed HCR 2°IHC with/without HCR RNA-ISH using unlabeled primary antibody probes and initiator-labeled secondary antibody probes for protein targets, split-initiator DNA probes for RNA targets, and simultaneous HCR signal amplification for all targets

Protein detection stage

1. Block tissue by applying 200 μL of antibody buffer on top of the sample. Incubate at room temperature for 1 h in a humidified chamber.
2. Prepare working concentration of primary antibodies in antibody buffer. Prepare 100 μL per section.
   NOTE: *follow manufacturer's guidelines for primary antibody working concentration.*
3. Drain slide by blotting edges on a Kimwipe and wipe around the section with another Kimwipe.
4. Add primary antibody solution to each section and incubate overnight (>12 h) at 4 °C in a humidified chamber.
   NOTE: *Incubation may be optimized (e.g., 1–2 h at room temperature) depending on sample type and thickness.*
5. Remove excess antibodies by immersing slide in 1× PBST at room temperature for 3 × 5 min.
6. Prepare working concentration of initiator-labeled secondary antibodies in antibody buffer. Prepare 100 μL per section.
7. Drain slide by blotting edges on a Kimwipe and wipe around the section with another Kimwipe.
8. Add secondary antibody solution to each section and incubate for 1 h at room temperature in a humidified chamber.
9. Remove excess antibodies by immersing slide in 1× PBST at room temperature for 3 × 5 min.
10. Proceed to **RNA detection stage** for co-detection of protein and RNA. Otherwise, proceed to **Amplification stage**.

RNA detection stage

1. Drain slide by blotting edges on a Kimwipe and wipe around the section with another Kimwipe.
2. Post-fix sample by adding 200 μL of 4% formaldehyde on the tissue.
   CAUTION: *use formaldehyde with extreme care as it is a hazardous material.*
3. Incubate slides for 10 min at room temperature.
4. Immerse slides for 2 × 5 min in PBST.
5. Immerse slides for 5 min in 5× SSCT.
6. Pre-warm a humidified chamber to 37 °C.
7. Drain slide by blotting edges on a Kimwipe and wipe around the section with another Kimwipe.
8. Add 200 μL of probe hybridization buffer on top of the tissue sample.
   CAUTION: *Probe hybridization buffer contains formamide, a hazardous material.*
   NOTE: *pre-heat probe hybridization buffer to 37 °C before use.*
9. Pre-hybridize for 10 min inside the humidified chamber.

Development • Supplementary information

10. Prepare a 16 nM probe solution by adding 1.6 pmol of each probe set (e.g. 1.6 μL of 1 μM stock) to 100 μL of probe hybridization buffer at 37 °C.
    NOTE: *This is the amount of probe set needed for each target on a single slide using 100 μL of incubation volume.*

11. Remove the pre-hybridization solution and drain excess buffer on slide by blotting edges on a Kimwipe.

12. Add 100 μL of the probe solution on top of the tissue sample.

13. Place a coverslip on the sample and incubate overnight ($>$12 h) in the 37 °C humidified chamber.

14. Immerse slide in probe wash buffer at 37 °C to float off coverslip.
    CAUTION: *Probe wash buffer contains formamide, a hazardous material.*

15. Remove excess probes by incubating slide at 37 °C in:
    (a) 75% of probe wash buffer / 25% 5× SSCT for 15 min
    (b) 50% of probe wash buffer / 50% 5× SSCT for 15 min
    (c) 25% of probe wash buffer / 75% 5× SSCT for 15 min
    (d) 100% 5× SSCT for 15 min

    NOTE: *Wash solutions should be pre-heated to 37 °C before use.*

16. Proceed to **Amplification stage**.

Development • Supplementary information

Amplification stage

1. Immerse slide in 5× SSCT at room temperature for 5 min.

2. Drain slide by blotting edges on a Kimwipe and wipe around the section with another Kimwipe.

3. Add 200 μL of amplification buffer on top of the tissue sample and pre-amplify in a humidified chamber for 30 min at room temperature.
   NOTE: *equilibrate amplification buffer to room temperature before use.*

4. Separately prepare 6 pmol of hairpin h1 and 6 pmol of hairpin h2 by snap cooling 2 μL of 3 μM stock (heat at 95 °C for 90 seconds and cool to room temperature in a dark drawer for 30 min).
   NOTE: *HCR hairpins h1 and h2 are provided in hairpin storage buffer ready for snap cooling. h1 and h2 should be snap cooled in separate tubes. This is the amount of hairpins needed for each target on a single slide using 100 μL of incubation volume.*

5. Prepare a 60 nM hairpin solution by adding all snap-cooled h1 hairpins and snap-cooled h2 hairpins to 100 μL of amplification buffer at room temperature per section.

6. Remove the pre-amplification solution and drain excess buffer on slide by blotting edges on a Kimwipe.

7. Add 100 μL of the hairpin solution on top of the tissue sample.

8. Incubate overnight (>12 h) in a dark humidified chamber at room temperature.

9. Remove excess hairpins by immersing slide in 5× SSCT at room temperature for:

   (a) 1 × 5 min
   (b) 2 × 15 min
   (c) 1 × 5 min

Sample mounting for microscopy

1. Drain slide by blotting edges on a Kimwipe and dry around the section with another Kimwipe.

2. Apply 35 μL of Slowfade Diamond antifade mountant with DAPI on top of the tissue.

3. Place a 22 × 30 mm No. 1 coverslip on top carefully to prevent air bubbles.

4. Slides can be stored at 4 °C protected from light prior to imaging.
   NOTE: *see Section S2.5 for details of epifluorescence microscope used to image FFPE mouse brain tissue section.*

Development • Supplementary information

S4.2.6 Buffers for HCR 2°IHC with/without HCR RNA-ISH

HCR probes (initiator-labeled antibody probes, split-initiator DNA probes), amplifiers, and buffers (antibody buffer, probe hybridization buffer, probe wash buffer, amplification buffer) are available from Molecular Instruments (`www.molecularinstruments.com`). Probe hybridization buffer, and probe wash buffer should be stored at -20 °C. Antibody buffer and amplification buffer should be stored at 4 °C. Make sure all solutions are well mixed before use.

**5× SSCT**	For 40 mL of solution
5× saline sodium citrate (SSC)	10 mL of 20× SSC
0.1% Tween 20	400 μL of 10% Tween 20
	Fill up to 40 mL with ultrapure H_2O

S4.2.7 Reagents and supplies

Pro-Par Clearant (ANATECH LTD Cat. # 510)
100% Ethanol (EtOH) (VWR Cat. # 89125-172)
100× citrate buffer pH 6.0 (Abcam Cat. # ab93678)
100× Tris-EDTA buffer pH 9.0 (Abcam Cat. # ab93684)
10× Phosphate-buffered saline (PBS) (Invitrogen Cat. # AM9624)
30% hydrogen peroxide (Sigma Aldrich Cat. #H1009)
Sodium hydroxide (Fisher Scientific Cat. #S318-500)
20× saline sodium citrate (SSC) (Life Technologies Cat. # 15557-044)
10% Tween 20 (Teknova Cat. # T0710)
SlowFade Diamond Antifade Mountant with DAPI (Invitrogen Cat. # S36973)
22 mm × 30 mm No. 1 coverslip (VWR Cat. # 48393-026)
6 band 240 W LED vegetative grow light (HTG Supply Cat. # LED-6B240)

S4.3 Protocols for FFPE human breast tissue sections

S4.3.1 Preparation of formalin-fixed paraffin-embedded (FFPE) human breast tissue sections

1. Bake slides in a dry oven for 1 h at 60 °C to improve sample adhesion to the slide.
2. In a fume hood, deparaffinize FFPE tissue by immersing in xylene for 2 × 5 min. Move slides up and down occasionally.
   CAUTION: *use xylene with care as it is a hazardous material.*
   NOTE: *Each 50 mL tube can fit two outward-facing slides. A volume of 30 mL is sufficient to immerse sections in the tube. If desired, a larger number of slides can be processed together using a Coplin jar.*
3. Incubate slides in 100% ethanol (EtOH) for 2 × 3 min at room temperature. Move slides up and down occasionally.
4. Rehydrate with a series of graded EtOH washes at room temperature.
   (a) 95% EtOH for 3 min
   (b) 70% EtOH for 3 min
   (c) Ultrapure water for 3 min
5. Remove slides from ultrapure water and gently tap off water.
6. Carefully dry around the tissue with a Kimwipe.
7. Draw a hydrophobic barrier around the tissue with a hydrophobic pen.
8. Apply 200 μL of 4 U/μL proteinase K solution for 7 min at room temperature.
   NOTE: *Proteolytic-Induced Epitope Retrieval (PIER) is used in place of Heat-Induced Epitope Retrieval (HIER). Optimal antigen retrieval method may differ depending on the antigen/antibody used.*
9. Gently tap off proteinase K solution and immerse slides in a Coplin jar with ultrapure water for 1 min.
10. Remove slides from ultrapure water and gently tap off water.
11. Carefully dry around the tissue with a Kimwipe.
12. Proceed to HCR assay.

S4.3.2 Buffer recipes for sample preparation

**Proteinase K solution**	For 800 μL of solution
4 U/μL proteinase K	4 μL of 800 U/μL proteinase K
	796 μL of 1× phosphate-buffered saline (PBS)

Development • Supplementary information

S4.3.3 Multiplexed HCR 2°IHC using unlabeled primary antibody probes and initiator-labeled secondary probes with simultaneous HCR signal amplification for all targets

Protein detection stage

1. Block tissue by adding 200 μL of antibody buffer on top of the sample. Incubate at room temperature for 1 h in a humidified chamber.
2. Prepare working concentration of primary antibodies in antibody buffer. Prepare 100 μL per section.
   NOTE: *follow manufacturer's guidelines for primary antibody working concentration.*
3. Drain slide by blotting edges on a Kimwipe and wipe around the section with another Kimwipe.
4. Add primary antibody solution to each section and incubate overnight (>12 h) at 4 °C in a humidified chamber.
   NOTE: *Incubation may be optimized (e.g., 1–2 h at room temperature) depending on sample type and thickness.*
5. Remove excess antibodies by washing 3 × 5 min with 100 μL of 1× PBST at room temperature.
6. Prepare working concentration of initiator-labeled secondary antibodies in antibody buffer. Prepare 100 μL per section.
7. Drain slide by blotting edges on a Kimwipe and wipe around the section with another Kimwipe.
8. Add secondary antibody solution to each section and incubate for 1 h at room temperature in a humidified chamber.
9. Remove excess antibodies by washing 3 × 5 min with 100 μL of 1× PBST at room temperature.

Amplification stage

1. Drain slide by blotting edges on a Kimwipe and wipe around the section with another Kimwipe.
2. Add 200 μL of amplification buffer on top of the tissue sample and pre-amplify in a humidified chamber for 30 min at room temperature.
   NOTE: *equilibrate amplification buffer to room temperature before use.*
3. Separately prepare 12 pmol of hairpin h1 and 12 pmol of hairpin h2 by snap cooling 4 μL of 3 μM stock (heat at 95 °C for 90 seconds and cool to room temperature in a dark drawer for 30 min).
   NOTE: *HCR hairpins h1 and h2 are provided in hairpin storage buffer ready for snap cooling. h1 and h2 should be snap cooled in separate tubes. This is the amount of hairpins needed for each target on a single slide using 200 μL of incubation volume.*
4. Prepare a 60 nM hairpin solution by adding all snap-cooled h1 hairpins and snap-cooled h2 hairpins to 200 μL of amplification buffer at room temperature per section.
5. Remove the pre-amplification solution and drain excess buffer on slide by blotting edges on a Kimwipe.
6. Add 200 μL of the hairpin solution on top of the tissue sample.
7. Incubate overnight (>12 h) in a dark humidified chamber at room temperature.
8. Remove excess hairpins by washing with 100 μL 5× SSCT at room temperature:
   (a) 2 × 5 min
   (b) 2 × 15 min
   (c) 1 × 5 min

Development • Supplementary information

Sample mounting for microscopy

1. Drain slide by blotting edges on a Kimwipe and dry around the section with another Kimwipe.
2. Apply 20 μL of Fluoromount-G with DAPI on top of the tissue.
3. Place a 22 $\times$ 30 mm No. 1.5 coverslip on top carefully to prevent air bubbles.
4. Seal the edges of the coverslip by applying nail polish hardener and allow it to dry for 30 min.
5. Slides can be stored at 4 °C protected from light prior to imaging.
   NOTE: *see Section S2.4 for details of confocal microscopes used to image FFPE human breast tissue sections.*

S4.3.4 Buffers for HCR 2°IHC

HCR probes (initiator-labeled antibody probes), amplifiers, and buffers (antibody buffer, amplification buffer) are available from Molecular Instruments (`www.molecularinstruments.com`). Antibody buffer and amplification buffer should be stored at 4 °C. Make sure all solutions are well mixed before use.

**PBST**	For 50 mL of solution
1× PBS	5 mL of 10× PBS
0.1% Tween 20	500 μL of 10% Tween 20
	Fill up to 50 mL with ultrapure H_2O

**5× SSCT**	For 40 mL of solution
5× saline sodium citrate (SSC)	10 mL of 20× SSC
0.1% Tween 20	400 μL of 10% Tween 20
	Fill up to 40 mL with ultrapure H_2O

S4.3.5 Reagents and supplies

Xylenes (Macron Cat. # 8668-16)
100% Ethanol (EtOH) (VWR Cat. # 89125-172)
Proteinase K, molecular biology grade (NEB Cat. # P8107S)
10× Phosphate-buffered saline (PBS) (Invitrogen Cat. # AM9624)
20× saline sodium citrate (SSC) (Life Technologies Cat. # 15557-044)
10% Tween 20 (Teknova Cat. # T0710)
Fluoromount-G with DAPI (SouthernBiotech Cat. # 0100-20)
22 mm × 30 mm No. 1.5 coverslip (VWR Cat. # 48393-151)

Development • Supplementary information

S4.4 Protocols for whole-mount zebrafish embryos

This protocol has been optimized for embryos at 27 hpf. Other developmental stages may require additional optimization.

S4.4.1 Preparation of whole-mount zebrafish embryos

1. Collect zebrafish embryos and incubate at 28 °C in a petri dish with egg H_2O.
2. Dechorionate embryos at 27 hpf and wash with fresh egg H_2O.
3. Transfer 40 embryos to a 2 mL eppendorf tube and remove excess egg H_2O.
4. Fix embryos in 2 mL of 4% paraformaldehyde (PFA) for 24 h at 4 °C.
CAUTION: *use PFA with extreme care as it is a hazardous material.*
NOTE: *use fresh PFA and cool to 4 °C before use to avoid increased autofluorescence.*
5. Wash embryos 3 × 5 min with 1 mL of 1× phosphate-buffered saline (PBS) to stop the fixation.
NOTE: *avoid using calcium chloride and magnesium chloride in PBS as this leads to increased autofluorescence in the samples.*
6. Dehydrate and permeabilize with a series of methanol (MeOH) washes at room temperature (1 mL each):
   (a) 100% MeOH for 4 × 10 min
   (b) 100% MeOH for 1 × 50 min
7. Store embryos at -20 °C overnight before use.
NOTE: *Embryos can be stored for six months at -20 °C.*
8. Rehydrate with a series of graded MeOH/PBST washes for 5 min each at room temperature (1 mL each):
   (a) 75% MeOH / 25% PBST
   (b) 50% MeOH / 50% PBST
   (c) 25% MeOH / 75% PBST
   (d) 5 × 100% PBST

S4.4.2 Buffer recipes for sample preparation

**4% Paraformaldehyde (PFA)**	For 25 mL of solution
4% PFA	1 g of PFA powder
1× PBS	25 mL of 1× PBS
	Heat to 50–60 °C to dissolve powder

**PBST**	For 50 mL of solution
1× PBS	5 mL of 10× PBS
0.1% Tween 20	500 μL of 10% Tween 20
	Fill up to 50 mL with ultrapure H_2O

Development • Supplementary information

S4.4.3 Multiplexed HCR 2°IHC using unlabeled primary antibody probes and initiator-labeled secondary antibody probes with simultaneous HCR signal amplification for all targets

Protein detection stage

1. Block embryos with 500 μL of antibody buffer for 4 h at 4 °C.
2. Transfer 8 embryos to a 1.5 mL Eppendorf tube for each sample.
3. Prepare working concentration of unlabeled primary antibodies in antibody buffer. Prepare 250 μL per sample.
NOTE: *follow manufacturer's guidelines for primary antibody working concentration.*
4. Remove antibody buffer and add primary antibody solution to embryos.
5. Incubate embryos overnight (>12 h) at 4 °C with gentle rotation (50 RPM).
6. Remove excess antibodies by washing 4 × 30 min with 500 μL of PBST at room temperature.
7. Prepare working concentration of initiator-labeled secondary antibodies in antibody buffer. Prepare 250 μL per sample.
8. Remove PBST and add secondary antibody solution to embryos.
9. Incubate embryos for 3 h at room temperature with gentle rotation (50 RPM).
10. Remove excess antibodies by washing 5 × 5 min with 500 μL of PBST at room temperature.
11. Wash 1 × 5 min with 500 μL of 5× SSCT at room temperature.

Amplification stage

1. Pre-amplify embryos with 350 μL of amplification buffer for 30 min at room temperature.
NOTE: *equilibrate amplification buffer to room temperature before use.*
2. Separately prepare 30 pmol of hairpin h1 and 30 pmol of hairpin h2 by snap cooling 10 μL of 3 μM stock (heat at 95 °C for 90 seconds and cool to room temperature in a dark drawer for 30 min).
NOTE: *HCR hairpins h1 and h2 are provided in hairpin storage buffer ready for snap cooling. h1 and h2 should be snap cooled in separate tubes. This is the amount of hairpins needed for each target on a single slide using 500 μL of incubation volume.*
3. Prepare a 60 nM hairpin solution by adding all snap-cooled h1 hairpins and snap-cooled h2 hairpins to 500 μL of amplification buffer at room temperature per sample.
4. Remove the pre-amplification solution and add the hairpin solution.
5. Incubate the samples overnight (>12 h) in the dark at room temperature.
6. Remove excess hairpins by washing with 500 μL of 5× SSCT at room temperature:
   (a) 2 × 5 min
   (b) 2 × 30 min
   (c) 1 × 5 min

Development • Supplementary information

S4.4.4 Sample mounting for microscopy

1. Make a chamber for mounting the embryos by aligning two stacks of Scotch tape (6 pieces per stack) 2 cm apart on a 25 mm $\times$ 75 mm SuperFrost Plus glass slide.

2. Pipet embryos onto glass slide with a cut P1000 pipet tip. Use a P200 pipet to remove excess 5$\times$ SSCT.

3. Gently add 50 μL Fluoromount-G onto the embryos.

4. Use an eyelash tool to gently position the embryos onto their side for lateral imaging.

5. Use fine forceps to gradually lower a 22 $\times$ 30 mm No. 1.5 coverslip onto the tape stacks.

6. Gently add Fluoromount-G via the open sides of the chamber until the chamber is full (approximately 100 μL).

7. Seal the edges of the coverslip by applying nail polish hardener.

8. Let nail polish hardener dry for 30 min.

9. Slides can be stored at 4 °C protected from light prior to imaging.
   NOTE: *see Section S2.4 for details of confocal microscopes used to image whole-mount zebrafish embryos.*

S4.4.5 Buffers for HCR 2°IHC

HCR probes (initiator-labeled antibody probes), amplifiers, and buffers (antibody buffer, amplification buffer) are available from Molecular Instruments (`www.molecularinstruments.com`). Antibody buffer and amplification buffer should be stored at 4 °C. Make sure all solutions are well mixed before use.

**PBST**	For 50 mL of solution
1× PBS	5 mL of 10× PBS
0.1% Tween 20	500 μL of 10% Tween 20
	Fill up to 50 mL with ultrapure H_2O

**5× SSCT**	For 40 mL of solution
5× saline sodium citrate (SSC)	10 mL of 20× SSC
0.1% Tween 20	400 μL of 10% Tween 20
	Fill up to 40 mL with ultrapure H_2O

S4.4.6 Reagents and supplies

Paraformaldehyde (Sigma Cat. # P6148)
10× Phosphate-buffered saline (PBS) (Invitrogen Cat. # AM9624)
Methanol (MeOH) (Mallinckrodt Chemicals Cat. # 3016-16)
20× saline sodium citrate (SSC) (Life Technologies Cat. # 15557-044)
10% Tween 20 (Teknova Cat. # T0710)
Fluoromount-G (SouthernBiotech Cat. # 0100-01)
25 mm × 75 mm SuperFrost Plus glass slide (VWR Cat. # 48311-703)
22 mm × 30 mm No. 1.5 coverslip (VWR Cat. # 48393-151)

Development • Supplementary information

S5 Additional studies

S5.1 Summary of signal-to-background estimates for HCR 1°IHC, HCR 2°IHC, and/or HCR RNA-ISH

Method	Sample	Microscopy	Target	Type	Probes	Amplifier	SIG/BACK	Plex	Figures	Table
HCR 1°ICC	mammalian cells on a slide	Confocal	HSP60	protein	1°mAb-init	B3-Alexa488	609 ± 18	3	2C, S1	S12
HCR 1°ICC	mammalian cells on a slide	Confocal	GM130	protein	1°mAb-init	B2-Alexa647	211 ± 15	3	2C, S1	S12
HCR 1°ICC	mammalian cells on a slide	Confocal	SC35	protein	1°mAb-init	B4-Alexa546	41 ± 2	3	2C, S1	S12
HCR 1°IHC	FFPE mouse brain section	Epifluorescence	TH	protein	1°mAb-init	B1-Alexa488	30 ± 5	4	2DE, S5	S13
HCR 1°IHC	FFPE mouse brain section	Epifluorescence	GFAP	protein	1°mAb-init	B3-Alexa546	290 ± 60	4	2DE, S5	S13
HCR 1°IHC	FFPE mouse brain section	Epifluorescence	MBP	protein	1°mAb-init	B5-Alexa647	58 ± 14	4	2DE, S5	S13
HCR 1°IHC	FFPE mouse brain section	Epifluorescence	MAP2	protein	1°mAb-init	B4-Alexa750	30 ± 8	4	2DE, S5	S13
HCR 2°ICC	mammalian cells on a slide	Confocal	PCNA	protein	1°mAb + 2°pAb-init	B5-Alexa647	87 ± 6	3	3C, S10	S14
HCR 2°ICC	mammalian cells on a slide	Confocal	HSP60	protein	1°mAb + 2°pAb-init	B3-Alexa546	106 ± 7	3	3C, S10	S14
HCR 2°ICC	mammalian cells on a slide	Confocal	SC35	protein	1°mAb + 2°pAb-init	B2-Alexa488	69 ± 6	3	3C, S10	S14
HCR 2°IHC	FFPE mouse brain section	Epifluorescence	TH	protein	1°pAb + 2°pAb-init	B4-Alexa488	72 ± 14	4	3DE, S14	S15
HCR 2°IHC	FFPE mouse brain section	Epifluorescence	GFAP	protein	1°pAb + 2°pAb-init	B1-Alexa546	23 ± 11	4	3DE, S14	S15
HCR 2°IHC	FFPE mouse brain section	Epifluorescence	PVALB	protein	1°mAb + 2°pAb-init	B5-Alexa647	170 ± 60	4	3DE, S14	S15
HCR 2°IHC	FFPE mouse brain section	Epifluorescence	MBP	protein	1°mAb + 2°pAb-init	B3-Alexa750	260 ± 40	4	3DE, S14	S15
HCR 2°IHC	whole-mount zebrafish embryo	Confocal	Elavl3/Elavl4	protein	1°mAb + 2°pAb-init	B1-Alexa647	15 ± 4	1	S19	S16
HCR 1°IHC	FFPE mouse brain section	Epifluorescence	TH	protein	1°mAb-init	B1-Alexa647	45 ± 9	2	4B, S29	S22
HCR 1°IHC	FFPE mouse brain section	Epifluorescence	TH	protein	1°mAb-init	B3-Alexa750	41 ± 9	2	4B, S29	S22
HCR 2°IHC	FFPE human breast section	Confocal	KRT17	protein	1°pAb + 2°pAb-init	B4-Alexa546	100 ± 30	2	4B, S30, S32	S24
HCR 2°IHC	FFPE human breast section	Confocal	KRT17	protein	1°pAb + 2°pAb-init	B3-Alexa647	110 ± 30	2	4B, S30, S32	S24
HCR 2°IHC	FFPE human breast section	Confocal	KRT19	protein	1°mAb + 2°pAb-init	B2-Alexa546	140 ± 10	2	S31, S32	S24
HCR 2°IHC	FFPE human breast section	Confocal	KRT19	protein	1°mAb + 2°pAb-init	B5-Alexa647	170 ± 10	2	S31, S32	S24
HCR 1°ICC + HCR RNA-ISH	mammalian cells on a slide	Confocal	PCNA	protein	1°mAb-init	B5-Alexa488	95 ± 6	4	5B, S33	S25
HCR 1°ICC + HCR RNA-ISH	mammalian cells on a slide	Confocal	HSP60	protein	1°mAb-init	B3-Alexa546	280 ± 10	4	5B, S33	S25
HCR 1°ICC + HCR RNA-ISH	mammalian cells on a slide	Confocal	*U6*	RNA	2 split-initiator pairs	B1-Alexa594	20.3 ± 1.3	4	5B, S33	S25
HCR 1°ICC + HCR RNA-ISH	mammalian cells on a slide	Confocal	*ACTB*	mRNA	10 split-initiator pairs	B2-Alexa647	107 ± 12	4	5B, S33	S25
HCR 1°IHC + HCR RNA-ISH	FFPE mouse brain section	Epifluorescence	TH	protein	1°mAb-init	B3-Alexa488	40 ± 6	4	5CD, S35	S26
HCR 1°IHC + HCR RNA-ISH	FFPE mouse brain section	Epifluorescence	MBP	protein	1°mAb-init	B5-Alexa546	37 ± 9	4	5CD, S35	S26
HCR 1°IHC + HCR RNA-ISH	FFPE mouse brain section	Epifluorescence	*Prkcd*	mRNA	31 split-initiator pairs	B1-Alexa647	160 ± 100	4	5CD, S35	S26
HCR 1°IHC + HCR RNA-ISH	FFPE mouse brain section	Epifluorescence	*Slc17a7*	mRNA	36 split-initiator pairs	B2-Alexa750	170 ± 50	4	5CD, S35	S26
HCR 2°ICC + HCR RNA-ISH	mammalian cells on a slide	Confocal	PCNA	protein	1°mAb + 2°pAb-init	B5-Alexa488	27 ± 2	4	6B, S41	S30
HCR 2°ICC + HCR RNA-ISH	mammalian cells on a slide	Confocal	HSP60	protein	1°mAb + 2°pAb-init	B4-Alexa546	240 ± 20	4	6B, S41	S30
HCR 2°ICC + HCR RNA-ISH	mammalian cells on a slide	Confocal	*U6*	RNA	2 split-initiator pairs	B1-Alexa594	279 ± 17	4	6B, S41	S30
HCR 2°ICC + HCR RNA-ISH	mammalian cells on a slide	Confocal	*HSP60*	mRNA	18 split-initiator pairs	B2-Alexa647	45 ± 6	4	6B , S41	S30
HCR 2°IHC + HCR RNA-ISH	FFPE mouse brain section	Epifluorescence	TH	protein	1°mAb + 2°pAb-init	B4-Alexa488	700 ± 300	4	6CD, S43	S31
HCR 2°IHC + HCR RNA-ISH	FFPE mouse brain section	Epifluorescence	MBP	protein	1°mAb + 2°pAb-init	B3-Alexa546	270 ± 30	4	6CD, S43	S31
HCR 2°IHC + HCR RNA-ISH	FFPE mouse brain section	Epifluorescence	*Prkcd*	mRNA	31 split-initiator pairs	B1-Alexa647	84 ± 20	4	6CD, S43	S31
HCR 2°IHC + HCR RNA-ISH	FFPE mouse brain section	Epifluorescence	*Slc17a7*	mRNA	36 split-initiator pairs	B2-Alexa750	80 ± 40	4	6CD, S43	S31

Table S9. Signal-to-background summary for protein imaging using HCR 1°IHC or HCR 2°IHC and for simultaneous protein and RNA imaging using HCR 1°IHC + HCR RNA-ISH or HCR 2°IHC + HCR RNA-ISH. Mean ± standard error of the mean. For mammalian cells on a slide, estimates are based on $N = 15$

Development • Supplementary information

Table S9 continued. representative rectangular regions (one rectangle in each of 5 individual cells in each of 3 replicate wells on a multi-well slide). For FFPE mouse brain sections and FFPE human breast sections, estimates are based on representative rectangular regions of $N = 3$ replicate sections. For whole-mount zebrafish embryos, estimates are based on representative rectangular regions of $N = 3$ replicate embryos.

	HCR 1°IHC	HCR 2°IHC	Overall
Mammalian cells on a slide	210 ± 170	87 ± 18	100 ± 40
FFPE mouse brain section	43 ± 13	120 ± 70	50 ± 20
FFPE human breast section	—	130 ± 20	130 ± 20
Whole-mount zebrafish embryo	—	15	15
Overall	45 ± 15	100 ± 40	90 ± 50

Table S10. Signal-to-background summary for protein imaging using HCR 1°IHC or HCR 2°IHC in mammalian cells on a slide, FFPE mouse brain sections, FFPE human breast sections, and whole-mount zebrafish embryos. Median ± median absolute deviation. The number of imaging scenarios for each combination of sample and method is as follows: (mammalian cells on a slide, HCR 1°IHC, $N = 3$), (FFPE mouse brain section, HCR 1°IHC, $N = 6$), (mammalian cells on a slide, HCR 2°IHC, $N = 3$), (FFPE mouse brain section, HCR 2°IHC, $N = 4$), (FFPE human breast section, HCR 2°IHC, $N = 4$), (whole-mount zebrafish embryo, HCR 2°IHC, $N = 1$). The total number of imaging scenarios is $N = 21$. See Table S9 for details.

	Target proteins	Target RNAs	Overall
Mammalian cells on a slide	170 ± 90	80 ± 40	100 ± 80
FFPE mouse brain section	160 ± 120	120 ± 40	120 ± 70
Overall	170 ± 120	100 ± 60	100 ± 70

Table S11. Signal-to-background summary for simultaneous protein and RNA imaging using HCR 1°IHC + HCR RNA-ISH or HCR 2°IHC + HCR RNA-ISH in mammalian cells on a slide or FFPE mouse brain sections. Median ± median absolute deviation. $N = 4$ imaging scenarios for each combination of sample and target types (two targets for each of two methods). The total number of imaging scenarios is $N = 16$. See Table S9 for details.

S5.2 Replicates, signal, background, background components, and noise for multiplexed HCR 1°IHC (cf. Figure 2)

S5.2.1 Mammalian cells on a slide

For 3-plex protein imaging using HCR 1°ICC in mammalian cells on a slide, the 4 channels are (3 proteins + DAPI):

- **Ch1:** Target protein HSP60, probe 1°mAb rabbit IgG anti-HSP60 labeled with B3 initiator, amplifier B3-Alexa488.
- **Ch2:** Target protein GM130, probe 1°mAb rabbit IgG anti-GM130 labeled with B2 initiator, amplifier B2-Alexa647.
- **Ch3:** Target protein SC35, probe 1°mAb mouse IgG1 anti-SC35 labeled with B4 initiator, amplifier B4-Alexa546.
- **Ch4:** DAPI.

Additional studies are presented as follows:

- Figure S1 displays 3-plex images for $N = 3$ replicate wells on a multi-well slide (cf. Figure 2C).
- Figures S2–S4 displays representative regions of individual channels used for measurement of signal and background for each target.
- Table S12 displays estimated values for signal, background, and signal-to-background for each target.

Protocol: HCR 1°ICC (Section S3.1) using initiator-labeled primary antibody probes with HCR signal amplification for all targets simultaneously.
Sample: HeLa cells.
Microscopy: Confocal.

Development • Supplementary information

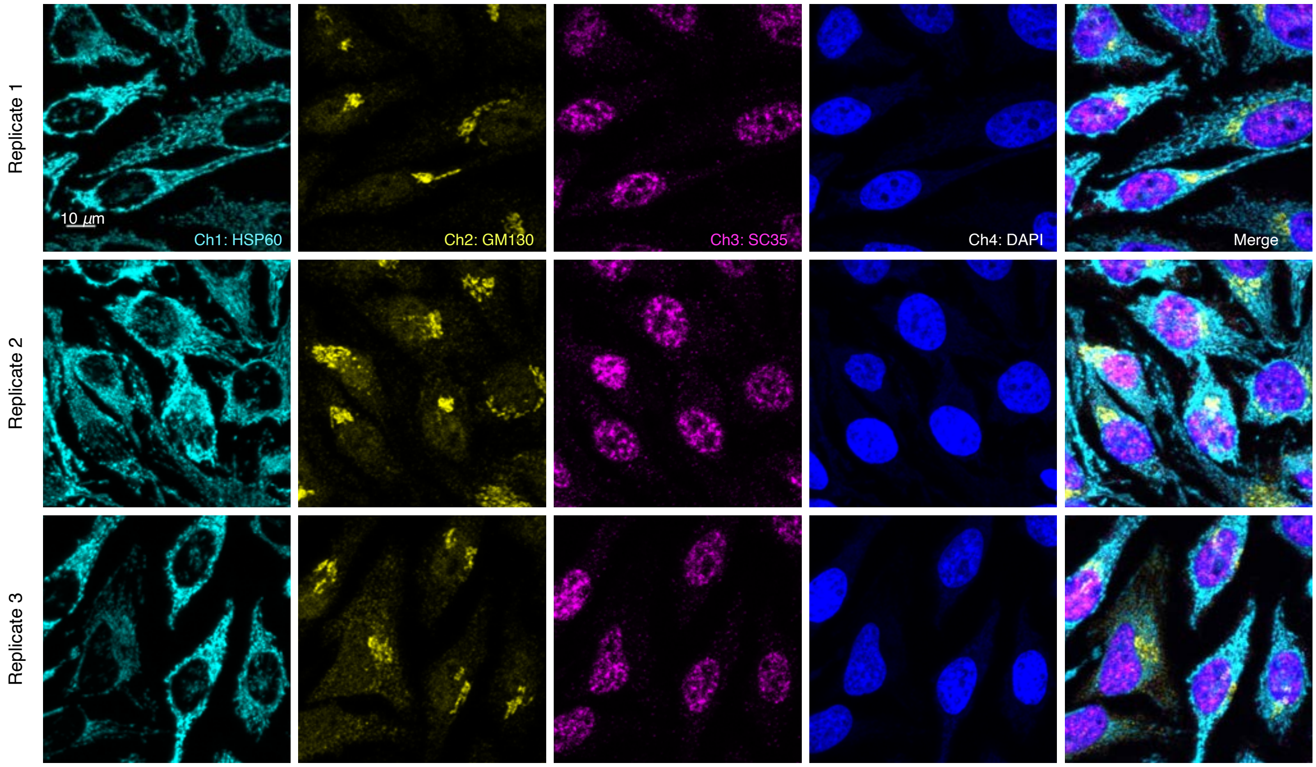

Figure S1. Replicates for 3-plex protein imaging using HCR 1°ICC in mammalian cells on a slide (cf. Figures 2C). 4-channel confocal images for 3 replicate wells on a multi-well slide; maximum intensity z-projection. Ch1: target protein HSP60 (Alexa488). Ch2: target protein GM130 (Alexa647). Ch3: target protein SC35 (Alexa546). Ch4: DAPI. Sample: HeLa cells.

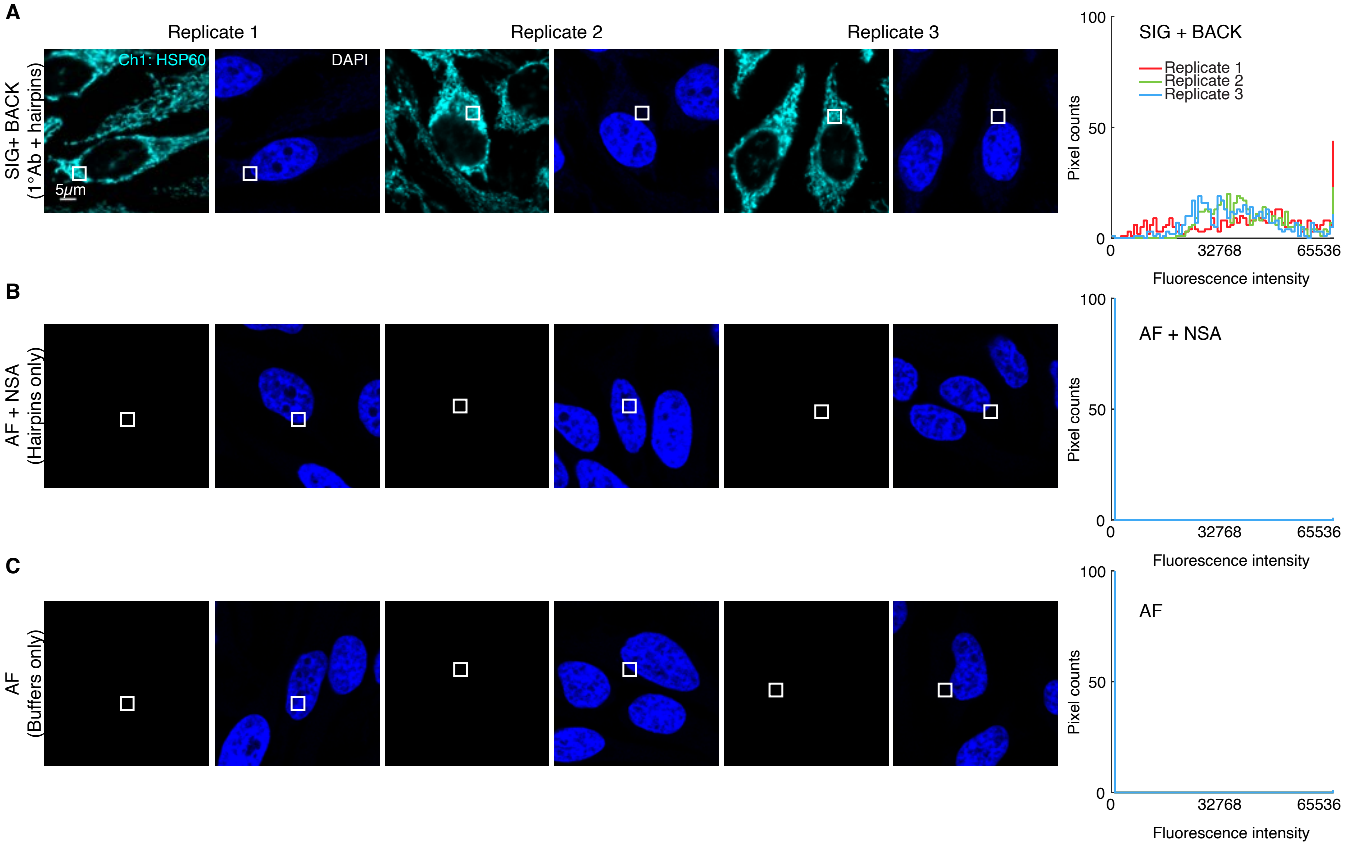

Figure S2. Measurement of signal, background, and background components for target protein HSP60 using HCR 1°ICC in mammalian cells on a slide (cf. Figure 2C). (A) Use experiment of Type 1 in Table S7A (1°Ab probe + hairpins) to measure SIG+BACK in a region of high expression. (B) Use experiment of Type 2 in Table S7B (no probes, hairpins only) to measure NSA+AF in a region of maximum background. (C) Use experiment of Type 3 in Table S7B (no probes, no hairpins) to measure AF in a region of maximum background. Left: confocal image collected with the microscope gain optimized to avoid saturating SIG+BACK pixels; DAPI channel facilitates placement of rectangles; single optical section. Right: pixel intensity histograms for representative regions (one rectangle in each of 5 individual cells in each of 3 replicate wells on a multi-well slide). Ch1: target protein HSP60 (Alexa488). Ch4: DAPI. Sample: HeLa cells.

Development • Supplementary information

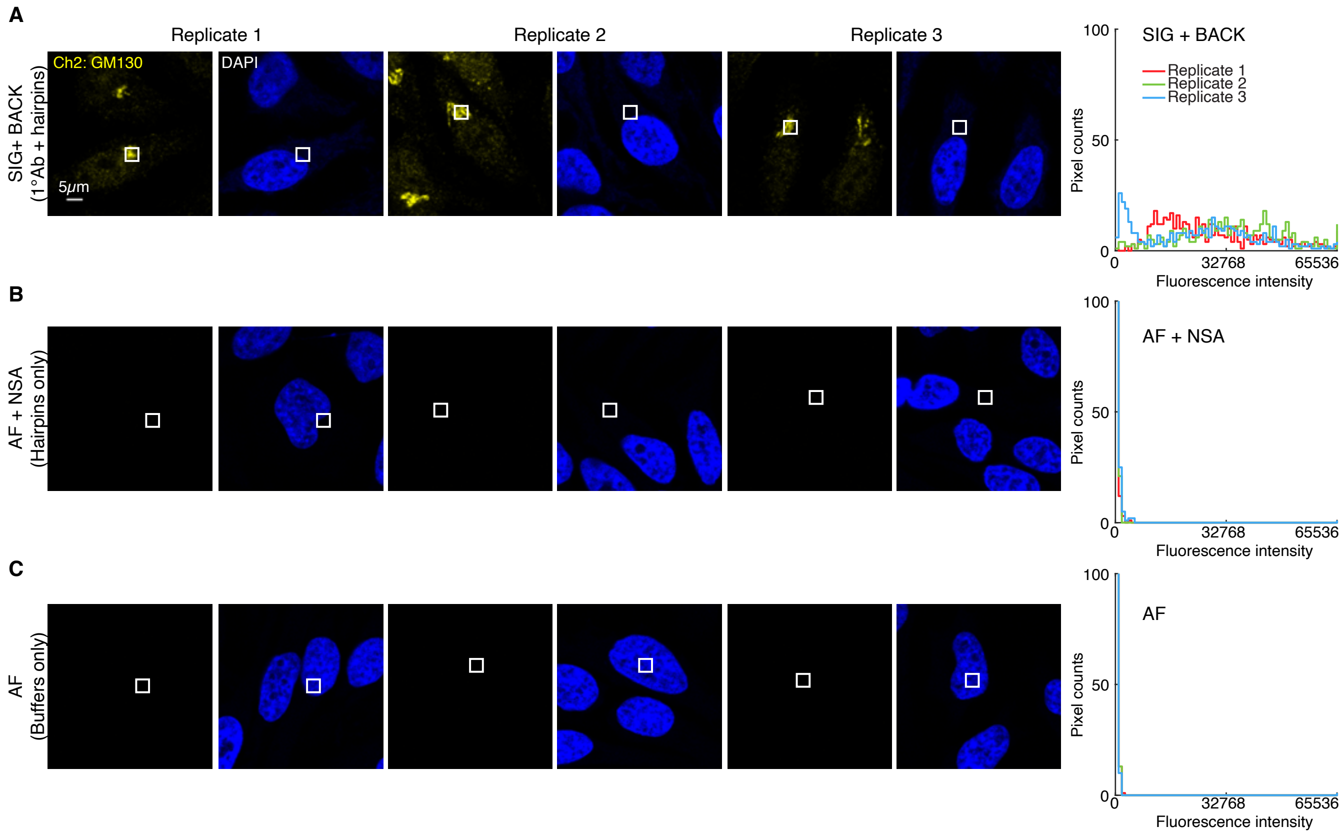

Figure S3. Measurement of signal, background, and background components for target protein GM130 using HCR 1°ICC in mammalian cells on a slide (cf. Figure 2C). (A) Use experiment of Type 1 in Table S7A (1°Ab probe + hairpins) to measure SIG+BACK in a region of high expression. (B) Use experiment of Type 2 in Table S7B (no probes, hairpins only) to measure NSA+AF in a region of maximum background. (C) Use experiment of Type 3 in Table S7B (no probes, no hairpins) to measure AF in a region of maximum background. Left: confocal image collected with the microscope gain optimized to avoid saturating SIG+BACK pixels; DAPI channel facilitates placement of rectangles; single optical section. Right: pixel intensity histograms for representative regions (one rectangle in each of 5 individual cells in each of 3 replicate wells on a multi-well slide). Ch2: target protein GM130 (Alexa 647). Ch4: DAPI. Sample: HeLa cells.

Development • Supplementary information

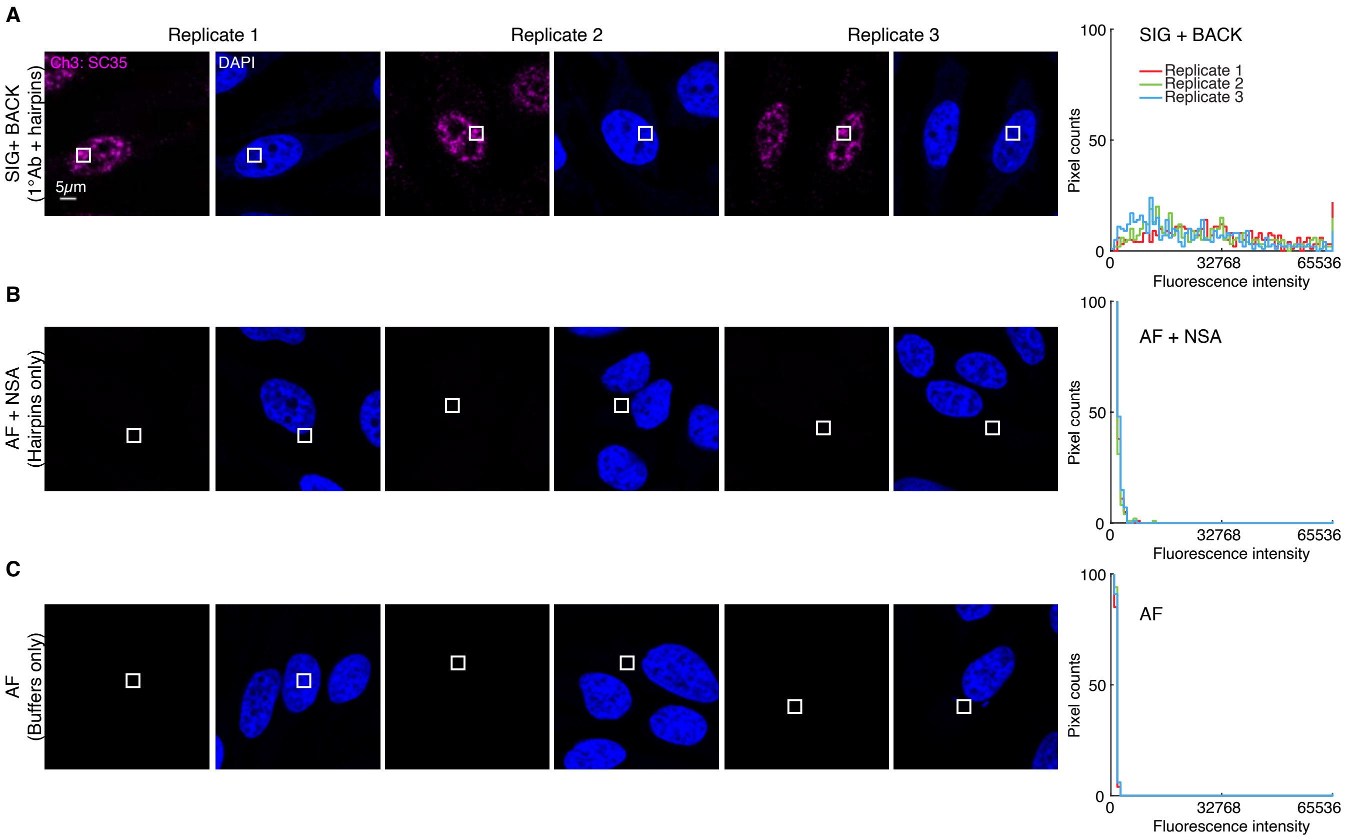

Figure S4. Measurement of signal, background, and background components for target protein SC35 using HCR 1°ICC in mammalian cells on a slide (cf. Figure 2C). (A) Use experiment of Type 1 in Table S7A (1°Ab probe + hairpins) to measure SIG+BACK in a region of high expression. (B) Use experiment of Type 2 in Table S7B (no probes, hairpins only) to measure NSA+AF in a region of maximum background. (C) Use experiment of Type 3 in Table S7B (no probes, no hairpins) to measure AF in a region of maximum background. Left: confocal image collected with the microscope gain optimized to avoid saturating SIG+BACK pixels; DAPI channel facilitates placement of rectangles; single optical section. Right: pixel intensity histograms for representative regions (one rectangle in each of 5 individual cells in each of 3 replicate wells on a multi-well slide). Ch3: target protein SC35 (Alexa546). Ch4: DAPI. Sample: HeLa cells.

Development • Supplementary information

	Quantity	Ch1: HSP60 B3-Alexa488	Ch2: GM130 B2-Alexa647	Ch3: SC35 B4-Alexa546	Reagents 1°Ab-init	Reagents Hairpins	Figure panel
**A**	SIG+BACK	39 400 ± 1100	29 400 ± 1500	27 300 ± 900	✓	✓	A
	SIG	39 400 ± 1100	29 300 ± 1500	26 700 ± 900			
	SIG/BACK	609 ± 18	211 ± 15	41 ± 2			
**B**	NSA+AF	64.6 ± 0.4	139 ± 6	650 ± 30		✓	B
	AF	66.3 ± 0.7	109.5 ± 1.8	257 ± 5			C
	NSA	< 0.8	29 ± 7	390 ± 30			

Table S12. Estimated signal-to-background and background components for 3-plex protein imaging using HCR 1°ICC in mammalian cells on a slide (cf. Figure 2C). (A) Estimated signal-to-background (SIG/BACK) based on methods of Section S2.6.2. The signal estimate SIG is calculated using the background approximation BACK ≈ NSA+AF. (B) Estimated background components (AF, NSA) based on methods of Section S2.6.3. Instrument noise is negligible using confocal microscopy so calculations use the approximation NOISE ≈ 0. Mean ± standard error of the mean, $N = 15$ representative rectangular regions (one rectangle in each of 5 individual cells in each of 3 replicate wells on a multi-well slide). Analysis based on representative rectangular regions (examples depicted in Figures S2–S4).

Development • Supplementary information

S5.2.2 FFPE mouse brain sections

For 4-plex protein imaging using HCR 1°IHC in FFPE mouse brain sections, the 5 channels are (4 proteins + DAPI):

- **Ch1:** Target protein TH, probe 1°mAb rabbit IgG anti-TH labeled with B1 initiator, amplifier B1-Alexa488.
- **Ch2:** Target protein GFAP, probe 1°mAb rabbit IgG anti-GFAP labeled with B3 initiator, amplifier B3-Alexa546.
- **Ch3:** Target protein MBP probe 1°mAb rabbit IgG anti-MBP labeled with B5 initiator, amplifier B5-Alexa647.
- **Ch4:** Target protein MAP2, probe 1°mAb rabbit IgG anti-MAP2 labeled with B4 initiator, amplifier B4-Alexa750.
- **Ch5:** DAPI.

Additional studies are presented as follows:

- Figure S5 displays 4-plex images for $N = 3$ replicate FFPE mouse brain sections (cf. Figures 2DE).
- Figures S6–S9 display representative regions of individual channels used for measurement of signal and background for each target.
- Table S13 displays estimated values for signal, background, and signal-to-background for each target.

Protocol: HCR 1°IHC (Section S3.2; without the optional autofluorescence bleaching protocol of Section S3.2.3) using initiator-labeled primary antibody probes with HCR signal amplification for all targets simultaneously.
Sample: FFPE C57BL/6 mouse brain section (coronal); thickness: 5 μm.
Microscopy: Epifluorescence.

Development • Supplementary information

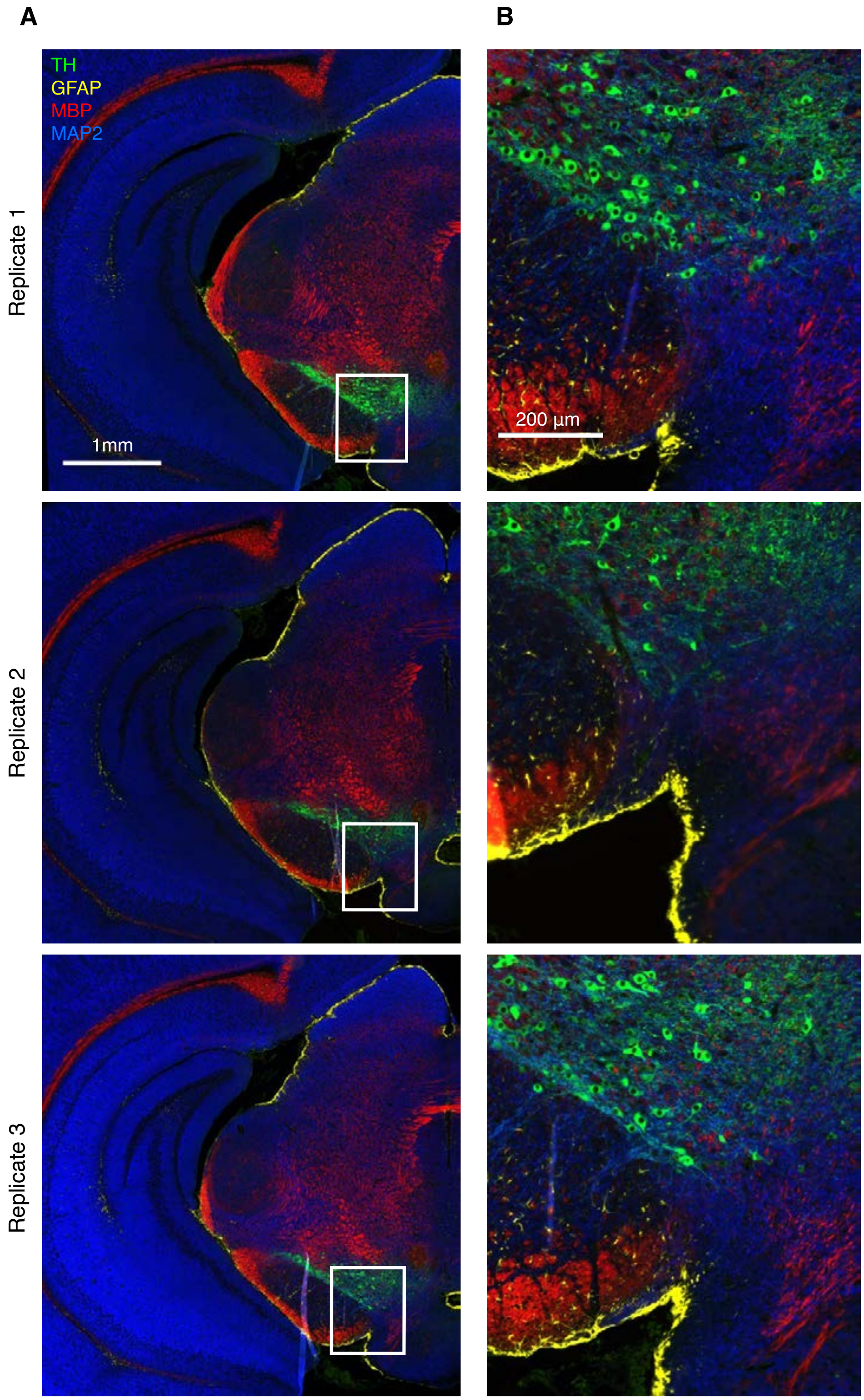

Figure S5. Replicates for 4-plex protein imaging using HCR 1°IHC in FFPE mouse brain sections (cf. Figures 2DE). (A) 4-channel epifluorescence images for 3 replicate FFPE mouse brain sections. (B) Zoom of the depicted region. Ch1: target protein TH (Alexa488). Ch2: target protein GFAP (Alexa546). Ch3: target protein MBP (Alexa647). Ch4: target protein MAP2 (Alexa750). Sample: FFPE C57BL/6 mouse brain section (coronal); thickness: 5 μm.

Development • Supplementary information

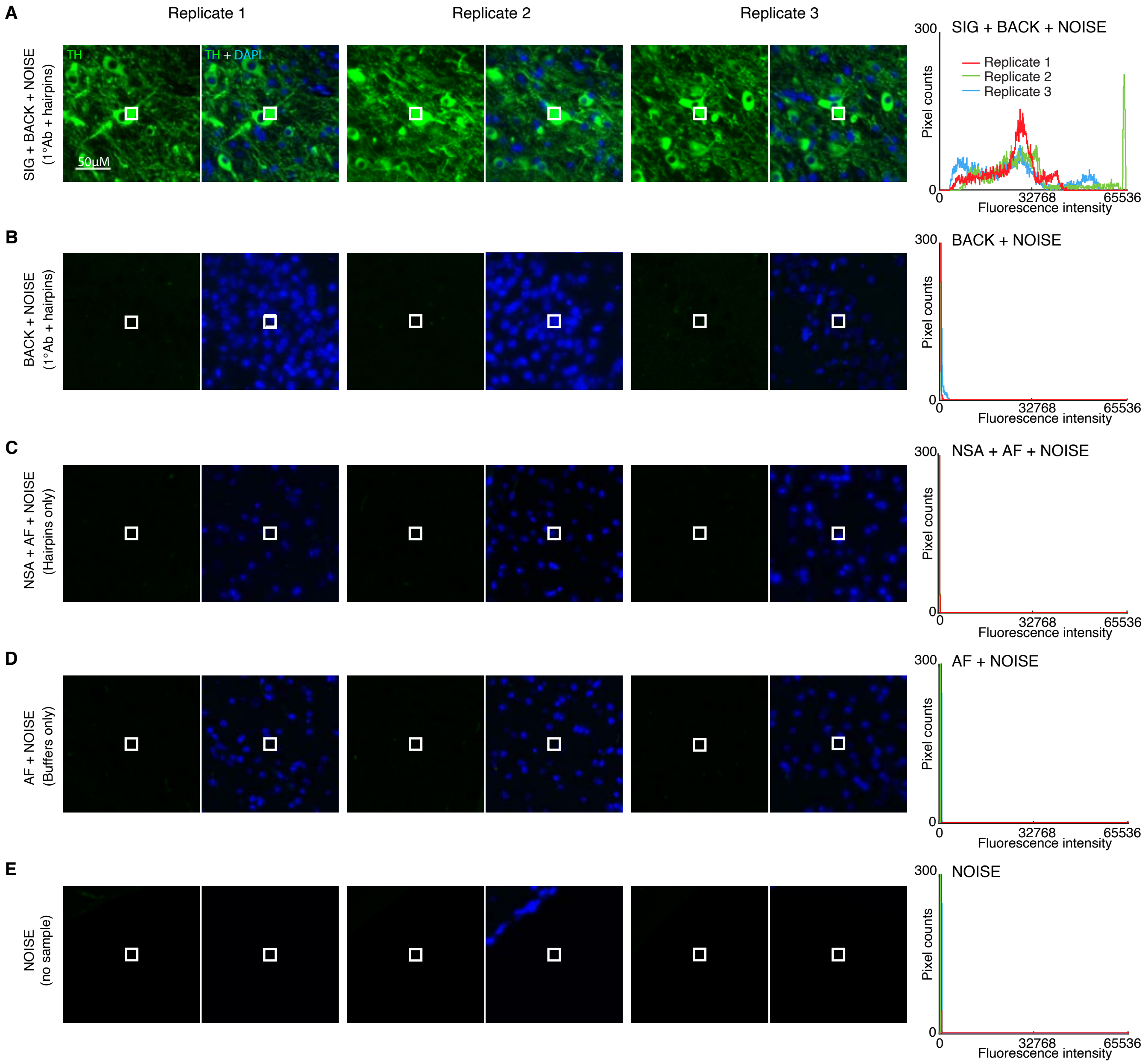

Figure S6. Measurement of signal, background, background components, and noise for target protein TH using HCR 1°IHC in FFPE mouse brain sections (cf. Figures 2DE). Use experiment of Type 1 in Table S7A (1°Ab probe + hairpins) to measure (A) SIG+BACK+NOISE in a region of high expression and (B) BACK+NOISE in a region of no/low expression. (C) Use experiment of Type 2 in Table S7B (no probes, hairpins only) to measure NSA+AF+NOISE in a region of high expression. Use experiment of Type 3 in Table S7B (no probes, no hairpins) to measure (D) AF+NOISE in a region of high expression and (E) NOISE in a region with no sample. Left: epifluorescence image collected with the microscope exposure time optimized to avoid saturating SIG+BACK+NOISE pixels; DAPI channel facilitates placement of rectangles. Right: pixel intensity histograms for representative regions (three rectangles per experiment type for each of three replicate FFPE mouse brain sections). Ch1: target protein TH (Alexa 488). Ch5: DAPI. Sample: FFPE C57BL/6 mouse brain section (coronal); thickness: 5 μm.

Development • Supplementary information

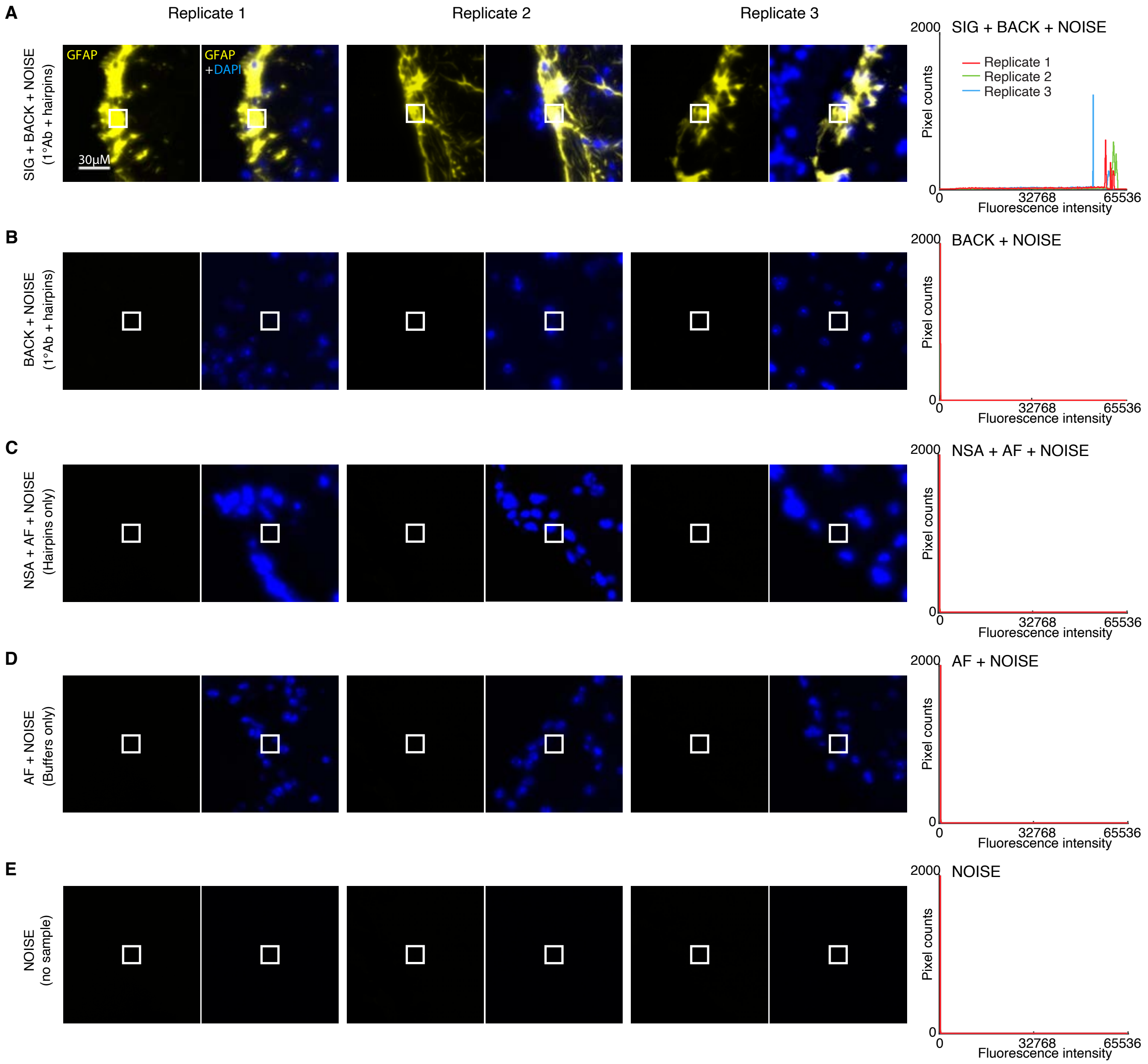

Figure S7. Measurement of signal, background, background components, and noise for target protein GFAP using HCR 1°IHC in FFPE mouse brain sections (cf. Figures 2DE). Use experiment of Type 1 in Table S7A (1°Ab probe + hairpins) to measure (A) SIG+BACK+NOISE in a region of high expression and (B) BACK+NOISE in a region of no/low expression. (C) Use experiment of Type 2 in Table S7B (no probes, hairpins only) to measure NSA+AF+NOISE in a region of high expression. Use experiment of Type 3 in Table S7B (no probes, no hairpins) to measure (D) AF+NOISE in a region of high expression and (E) NOISE in a region with no sample. Left: epifluorescence image collected with the microscope exposure time optimized to avoid saturating SIG+BACK+NOISE pixels; DAPI channel facilitates placement of rectangles. Right: pixel intensity histograms for representative regions (three rectangles per experiment type for each of three replicate FFPE mouse brain sections). Ch2: target protein GFAP (Alexa546). Ch5: DAPI. Sample: FFPE C57BL/6 mouse brain section (coronal); thickness: 5 μm.

Development • Supplementary information

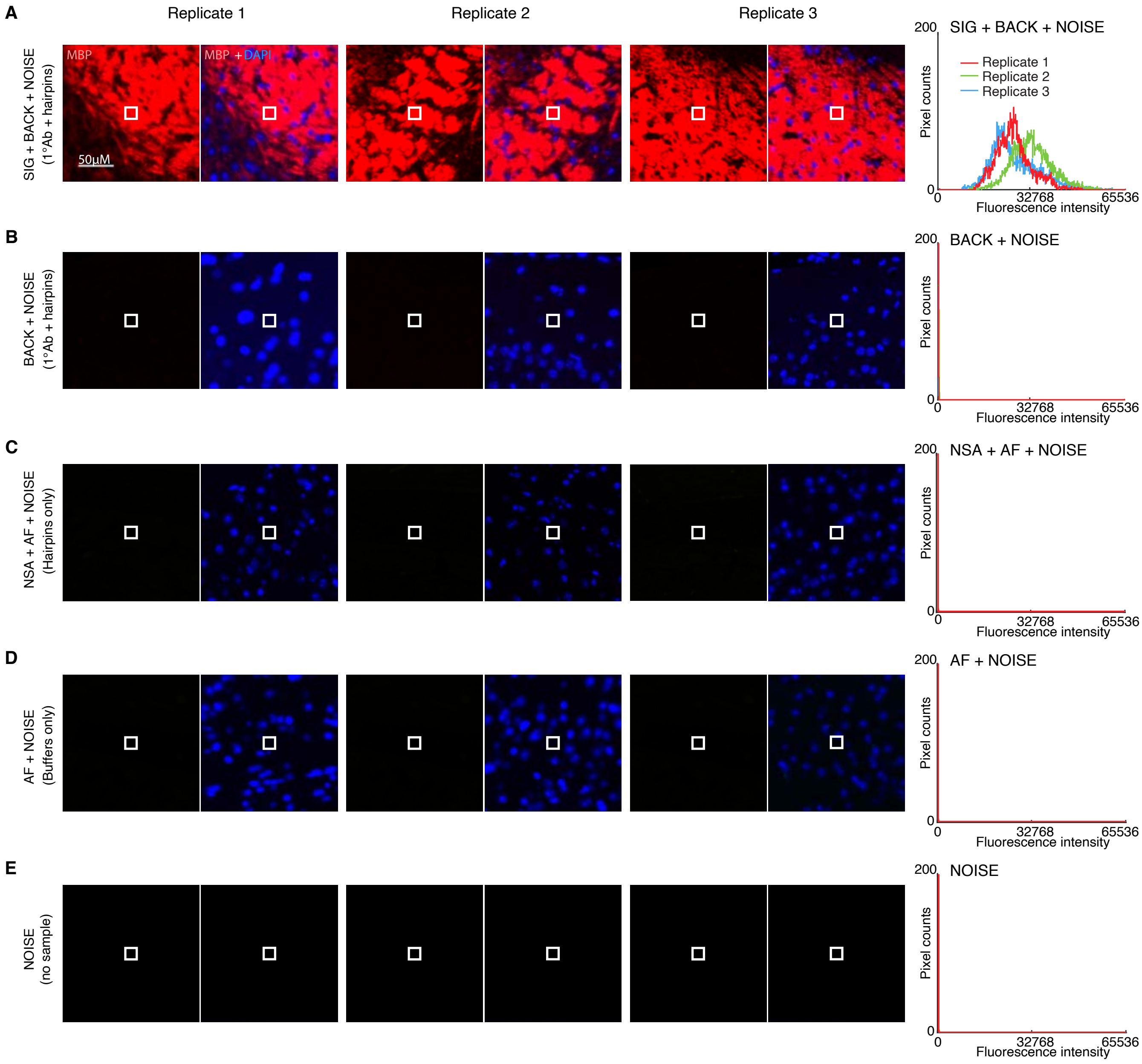

Figure S8. Measurement of signal, background, background components, and noise for target protein MBP using HCR 1°IHC in FFPE mouse brain sections (cf. Figures 2DE). Use experiment of Type 1 in Table S7A (1°Ab probe + hairpins) to measure (A) SIG+BACK+NOISE in a region of high expression and (B) BACK+NOISE in a region of no/low expression. (C) Use experiment of Type 2 in Table S7B (no probes, hairpins only) to measure NSA+AF+NOISE in a region of high expression. Use experiment of Type 3 in Table S7B (no probes, no hairpins) to measure (D) AF+NOISE in a region of high expression and (E) NOISE in a region with no sample. Left: epifluorescence image collected with the microscope exposure time optimized to avoid saturating SIG+BACK+NOISE pixels; DAPI channel facilitates placement of rectangles. Right: pixel intensity histograms for representative regions (three rectangles per experiment type for each of three replicate FFPE mouse brain sections). Ch3: target protein MBP (Alexa647). Ch5: DAPI. Sample: FFPE C57BL/6 mouse brain section (coronal); thickness: 5 μm.

Development • Supplementary information

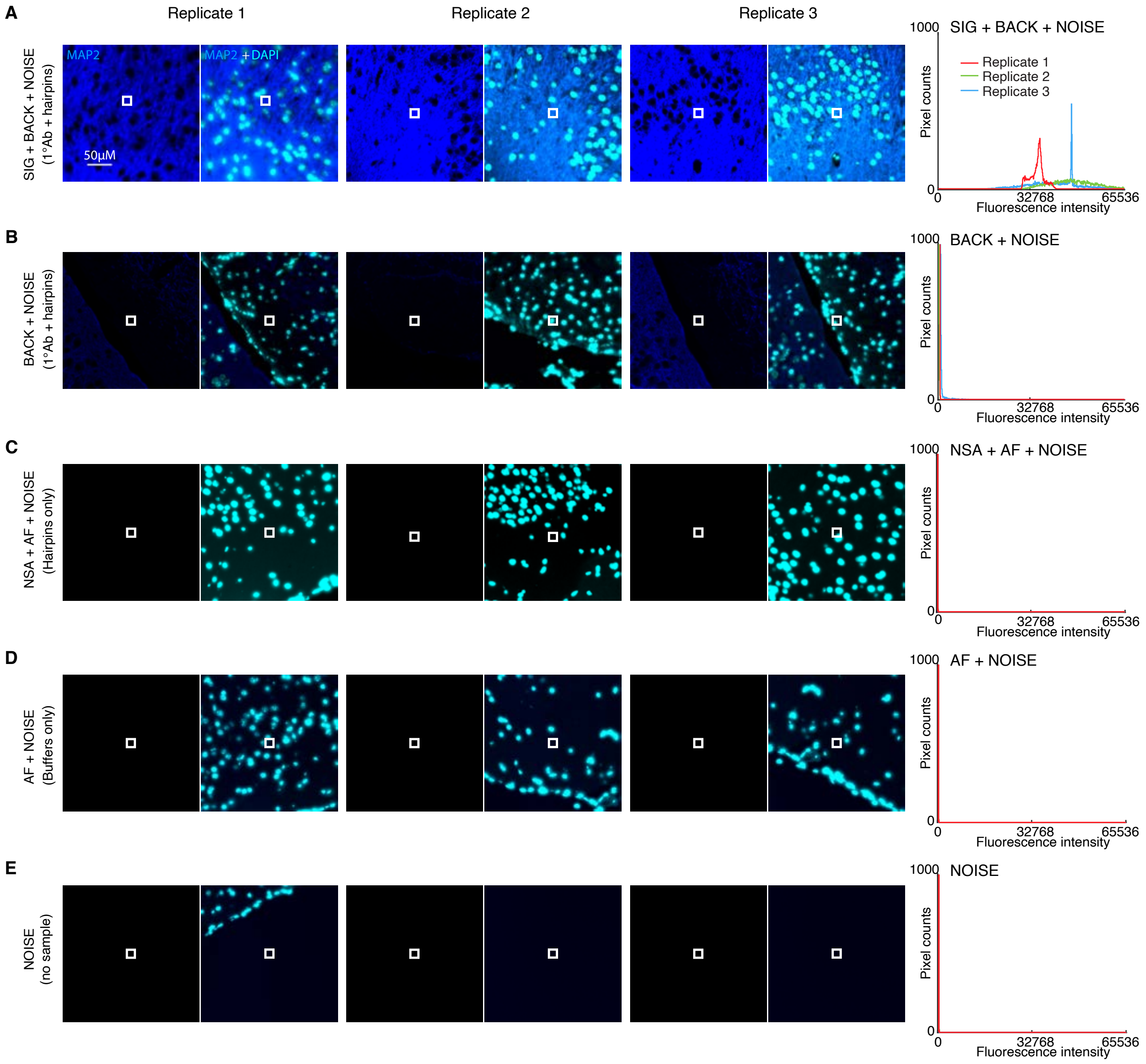

Figure S9. Measurement of signal, background, background components, and noise for target protein MAP2 using HCR 1°IHC in FFPE mouse brain sections (cf. Figures 2DE). Use experiment of Type 1 in Table S7A (1°Ab probe + hairpins) to measure (A) SIG+BACK+NOISE in a region of high expression and (B) BACK+NOISE in a region of no/low expression. (C) Use experiment of Type 2 in Table S7B (no probes, hairpins only) to measure NSA+AF+NOISE in a region of high expression. Use experiment of Type 3 in Table S7B (no probes, no hairpins) to measure (D) AF+NOISE in a region of high expression and (E) NOISE in a region with no sample. Left: epifluorescence image collected with the microscope exposure time optimized to avoid saturating SIG+BACK+NOISE pixels; DAPI channel facilitates placement of rectangles. Right: pixel intensity histograms for representative regions (three rectangles per experiment type for each of three replicate FFPE mouse brain sections). Ch4: target protein MAP2 (Alexa750). Ch5: DAPI. Sample: FFPE C57BL/6 mouse brain section (coronal); thickness: 5 μm.

	Quantity	Ch1: TH B1-Alexa488		Ch2: GFAP B3-Alexa546		Ch3: MBP B5-Alexa647		Ch4: MAP2 B4-Alexa750		Reagents 1°Ab-init	Reagents Hairpins	Expression region	Figure panel
**A**	SIG+BACK+NOISE	27 000	± 4000	48 000	± 5000	28 000	± 4000	41 000	± 6000	✓	✓	high	A
	BACK+NOISE	1060	± 90	260	± 30	720	± 90	1500	± 300	✓	✓	low/no	B
	NOISE	190	± 16	94	± 5	249	± 18	119	± 3	✓	✓	no sample	E
	SIG	26 000	± 4000	48 000	± 5000	28 000	± 4000	40 000	± 6000				
	BACK	870	± 90	170	± 30	500	± 100	1300	± 300				
	SIG/BACK	30	± 5	290	± 60	58	± 14	30	± 8				
**B**	NSA+AF+NOISE	650	± 20	145	± 13	380	± 30	147	± 8		✓	high	C
	AF+NOISE	600	± 20	128	± 13	303	± 3	115	± 2			high	D
	NSD	420	± 90	110	± 30	300	± 100	1300	± 300				
	NSA	50	± 30	18	± 18	80	± 30	30	± 8				
	AF	410	± 30	34	± 14	54	± 19	< 4					

Table S13. Estimated signal-to-background, background components, and noise for 4-plex protein imaging using HCR 1°IHC in FFPE mouse brain sections (cf. Figures 2DE). (A) Estimated signal-to-background (SIG/BACK) based on methods of Section S2.6.2. (B) Estimated background components (AF, NSA, NSD) based on methods of Section S2.6.3. Mean ± standard error of the mean, $N = 3$ replicate FFPE mouse brain sections. Analysis based on representative rectangular regions (examples depicted in Figures S6–S9).

S5.3 Replicates, signal, background, background components, and noise for multiplexed HCR 2°IHC (cf. Figure 3)

S5.3.1 Mammalian cells on a slide

For 3-plex protein imaging using HCR 2°ICC in mammalian cells on a slide, the 4 channels are (3 proteins + DAPI):

- **Ch1:** Target protein PCNA, probe 1°mAb mouse IgG2a anti-PCNA, probe 2°pAb goat anti-mouse Fcγ subclass 2a specific labeled with B5 initiator, amplifier B5-Alexa647.
- **Ch2:** Target protein HSP60, probe 1°mAb probe rabbit anti-HSP60, probe 2°pAb donkey anti-rabbit labeled with B3 initiator, amplifier B3-Alexa546.
- **Ch3:** Target protein SC35, probe 1°mAb mouse IgG1 anti-SC35, probe 2°pAb goat anti-mouse Fcγ subclass 1 specific labeled with B2 initiator, amplifier B2-Alexa488.
- **Ch4:** DAPI.

Additional studies are presented as follows:

- Figure S10 displays 3-plex images for $N = 3$ replicate wells on a multi-well slide (cf. Figure 3C).
- Figures S11–S13 displays representative regions of individual channels used for measurement of signal and background for each target.
- Table S14 displays estimated values for signal, background, and signal-to-background for each target.

Protocol: HCR 2°ICC (Section S4.1) using unlabeled primary antibody probes and initiator-labeled secondary antibody probes with HCR signal amplification for all targets simultaneously.
Sample: HeLa cells.
Microscopy: Confocal.

Development • Supplementary information

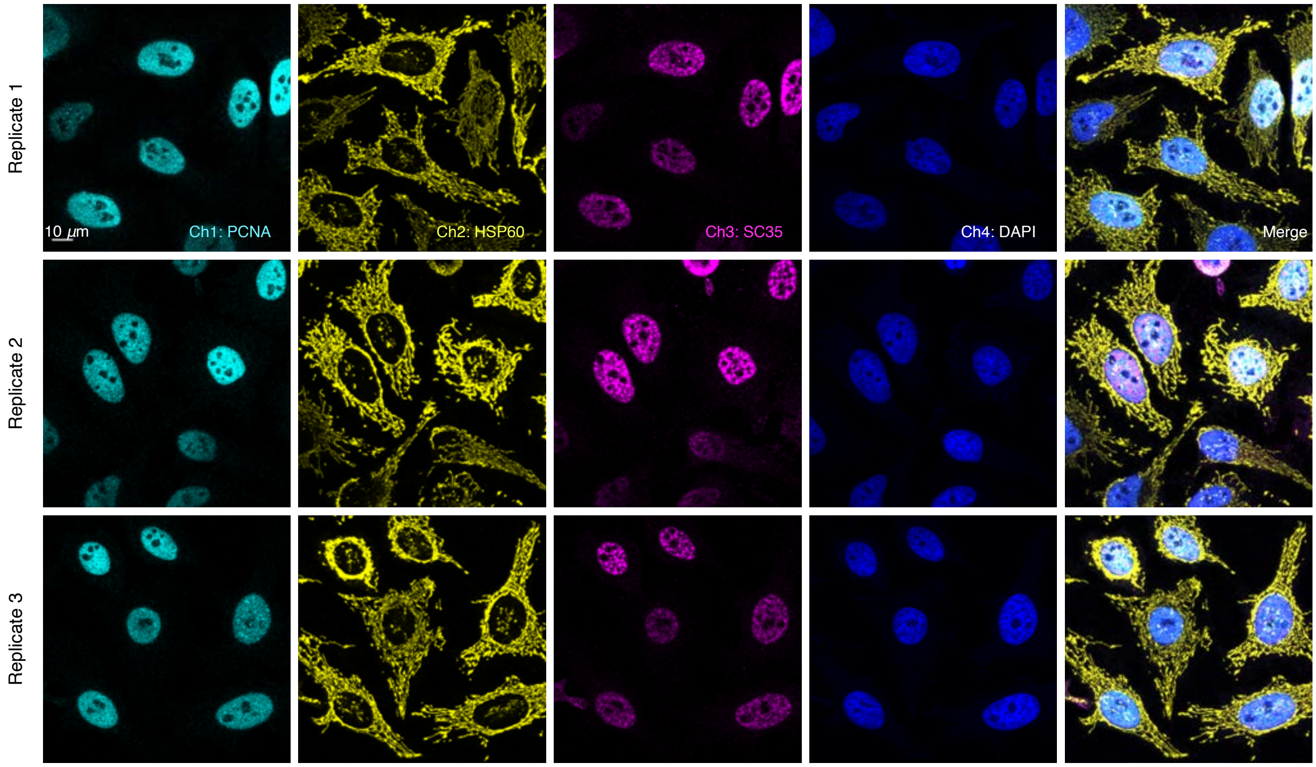

Figure S10. Replicates for 3-plex protein imaging using HCR 2°ICC in mammalian cells on a slide (cf. Figures 3C). 4-channel confocal images for 3 replicate wells on a multi-well slide; maximum intensity z-projection. Ch1: target protein PCNA (Alexa647). Ch2: target protein HSP60 (Alexa546). Ch3: target protein SC35 (Alexa488). Ch4: DAPI. Sample: HeLa cells.

Development • Supplementary information

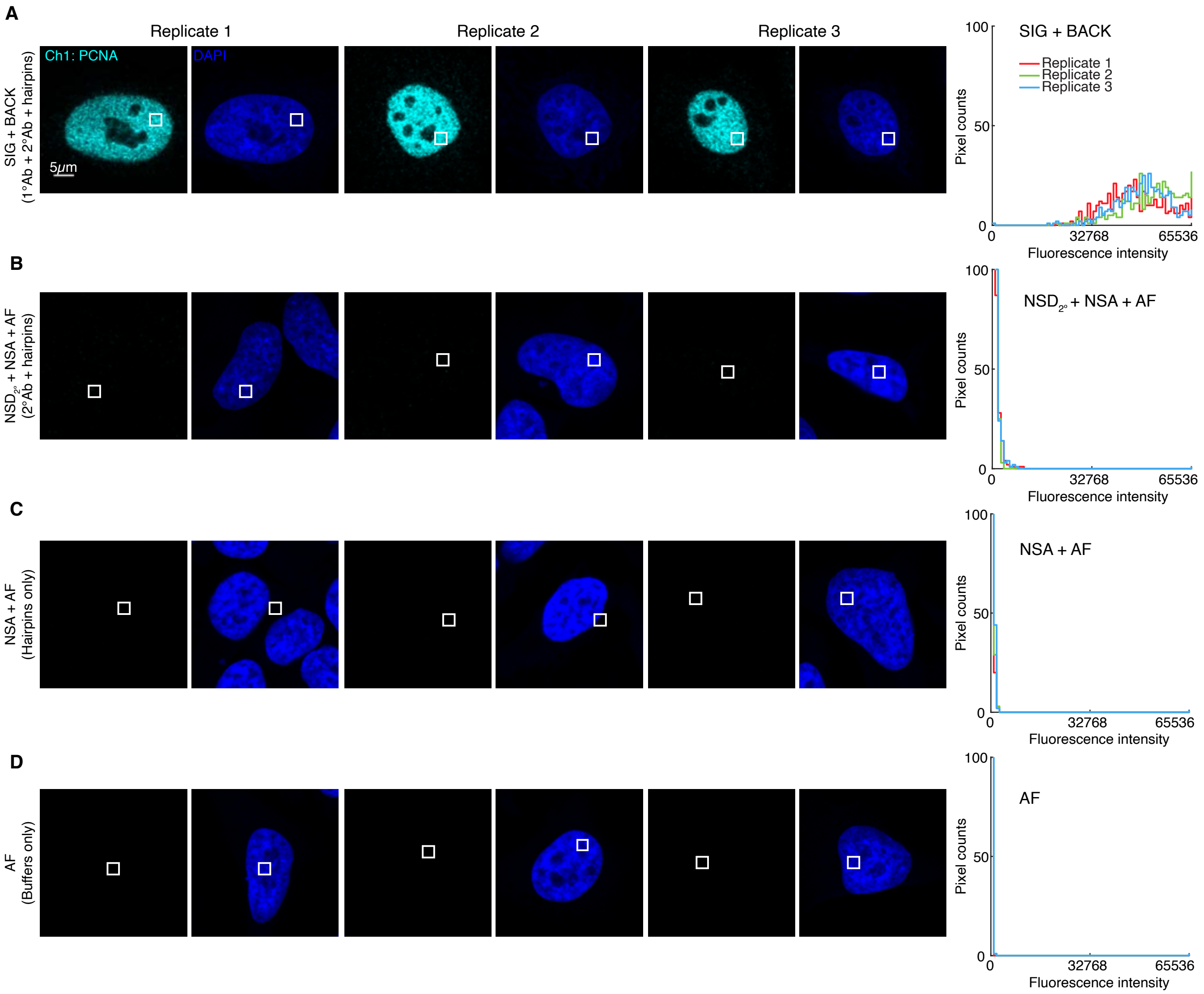

Figure S11. Measurement of signal, background, and background components for target protein PCNA using HCR 2°ICC in mammalian cells on a slide (cf. Figure 3C). (A) Use experiment of Type 1 in Table S8A (1°Ab probe + 2°Ab probe + hairpins) to measure SIG+BACK in a region of high expression. (B) Use experiment of Type 4 in Table S8B (2°Ab probes + hairpins) to measure NSD_{2°+NSA+AF in a region of maximum background. (C) Use experiment of Type 2 in Table S8B (no probes, hairpins only) to measure NSA+AF in a region of maximum background. (D) Use experiment of Type 3 in Table S8B (no probes, no hairpins) to measure AF in a region of maximum background. Left: confocal image collected with the microscope gain optimized to avoid saturating SIG+BACK pixels; DAPI channel facilitates placement of rectangles; single optical section. Right: pixel intensity histograms for representative regions (one rectangle in each of 5 individual cells in each of 3 replicate wells on a multi-well slide). Ch1: target protein PCNA (Alexa647). Ch4: DAPI. Sample: HeLa cells.

Development • Supplementary information

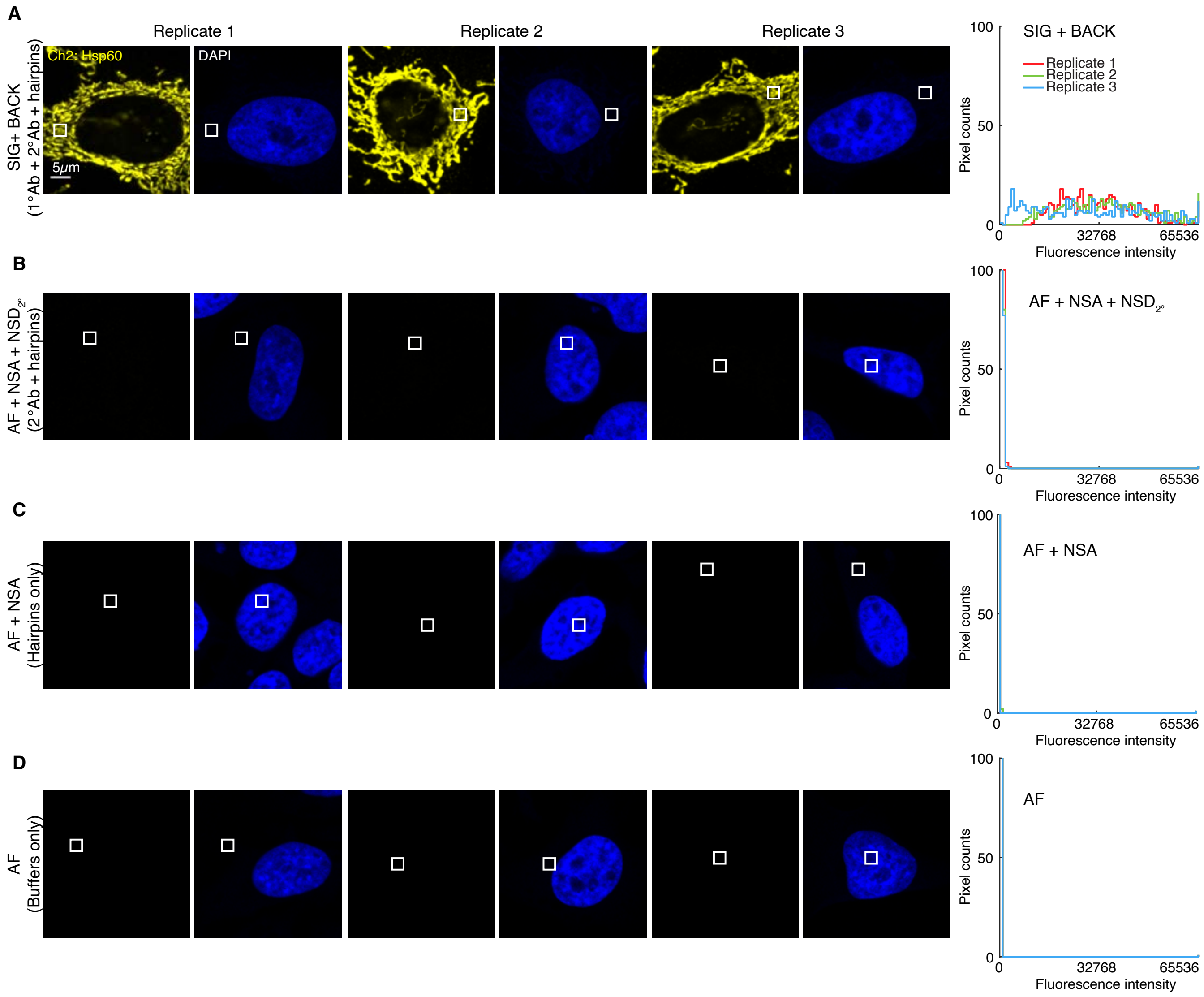

Figure S12. Measurement of signal, background, and background components for target protein HSP60 using HCR 2°ICC in mammalian cells on a slide (cf. Figure 3C). (A) Use experiment of Type 1 in Table S8A (1°Ab probe + 2°Ab probe + hairpins) to measure SIG+BACK in a region of high expression. (B) Use experiment of Type 4 in Table S8B (2°Ab probes + hairpins) to measure NSD_{2°+NSA+AF in a region of maximum background. (C) Use experiment of Type 2 in Table S8B (no probes, hairpins only) to measure NSA+AF in a region of maximum background. (D) Use experiment of Type 3 in Table S8B (no probes, no hairpins) to measure AF in a region of maximum background. Left: confocal image collected with the microscope gain optimized to avoid saturating SIG+BACK pixels; DAPI channel facilitates placement of rectangles; single optical section. Right: pixel intensity histograms for representative regions (one rectangle in each of 5 individual cells in each of 3 replicate wells on a multi-well slide). Ch2: target protein HSP60 (Alexa546). Ch4: DAPI. Sample: HeLa cells.

Development • Supplementary information

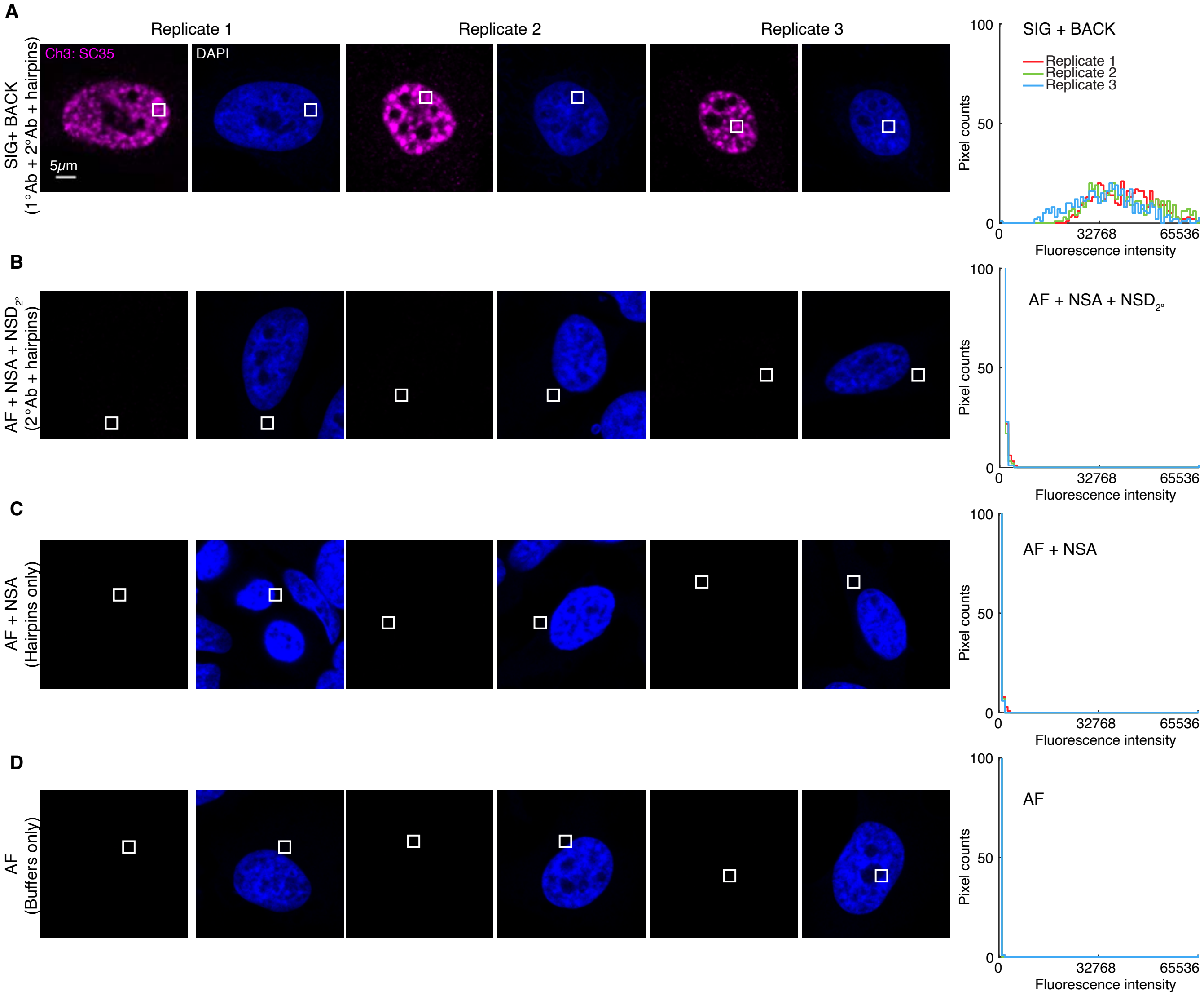

Figure S13. Measurement of signal, background, and background components for target protein SC35 using HCR 2°ICC in mammalian cells on a slide (cf. Figure 3C). (A) Use experiment of Type 1 in Table S8A (1°Ab probe + 2°Ab probe + hairpins) to measure SIG+BACK in a region of high expression. (B) Use experiment of Type 4 in Table S8B (2°Ab probes + hairpins) to measure $NSD_{2\circ}$+NSA+AF in a region of maximum background. (C) Use experiment of Type 2 in Table S8B (no probes, hairpins only) to measure NSA+AF in a region of maximum background. (D) Use experiment of Type 3 in Table S8B (no probes, no hairpins) to measure AF in a region of maximum background. Left: confocal image collected with the microscope gain optimized to avoid saturating SIG+BACK pixels; DAPI channel facilitates placement of rectangles; single optical section. Right: pixel intensity histograms for representative regions (one rectangle in each of 5 individual cells in each of 3 replicate wells on a multi-well slide). Ch3: target protein SC35 (Alexa488). Ch4: DAPI. Sample: HeLa cells.

Development • Supplementary information

		Ch1: PCNA	Ch2: Hsp60	Ch3: SC35	Reagents			Figure
	Quantity	B5-Alexa647	B3-Alexa546	B2-Alexa488	1°Ab	2°Ab-init	Hairpins	panel
**A**	SIG+BACK	43 300 ± 1700	31 500 ± 1200	33 000 ± 2000	✓	✓	✓	A
	SIG	42 800 ± 1700	31 200 ± 1200	33 000 ± 2000				
	SIG/BACK	87 ± 6	106 ± 7	69 ± 6				
**B**	NSD_{2°+NSA+AF	490 ± 30	293 ± 17	470 ± 30		✓	✓	B
	NSA+AF	138 ± 6	79 ± 3	113 ± 3			✓	C
	AF	64.5 ± 0.5	51.5 ± 0.3	100 ± 3				D
	NSD_{2°	360 ± 30	215 ± 17	360 ± 30				
	NSA	74 ± 6	27 ± 3	13 ± 5				

Table S14. Estimated signal-to-background and background components for 3-plex protein imaging using HCR 2°ICC in mammalian cells on a slide (cf. Figure 3C). (A) Estimated signal-to-background (SIG/BACK) based on methods of Section S2.6.2. The signal estimate SIG is calculated using the background approximation BACK ≈ NSD_{2°+NSA+AF. (B) Estimated background components (AF, NSA, NSD_{2°) based on methods of Section S2.6.3. Instrument noise is negligible using confocal microscopy so calculations use the approximation NOISE ≈ 0. Mean ± standard error of the mean, $N = 15$ representative rectangular regions (one rectangle in each of 5 individual cells in each of 3 replicate wells on a multi-well slide). Analysis based on rectangular regions depicted in Figures S11–S13.

S5.3.2 FFPE mouse brain sections

For 4-plex protein imaging using HCR 2°IHC in FFPE mouse brain sections, the 5 channels are (4 proteins + DAPI):

- **Ch1:** Target protein TH, probe 1°pAb sheep IgG anti-TH, probe 2°pAb donkey anti-sheep IgG labeled with B4 initiator, amplifier B4-Alexa488.
- **Ch2:** Target protein GFAP, probe 1°pAb chicken IgY anti-GFAP, probe 2°pAb donkey anti-chicken IgG labeled with B1 initiator, amplifier B1-Alexa546.
- **Ch3:** Target protein PVALB, probe 1°mAb rabbit IgG anti-PVALB, probe 2°pAb donkey anti-rabbit IgG labeled with B5 initiator, amplifier B5-Alexa647.
- **Ch4:** Target protein MBP, probe 1°mAb rat IgG2a anti-MBP, probe 2°pAb donkey anti-rat IgG labeled with B3 initiator, amplifier B3-Alexa750.
- **Ch5:** DAPI.

Additional studies are presented as follows:

- Figure S14 displays 4-plex images for $N = 3$ replicate FFPE mouse brain sections (cf. Figures 3DE).
- Figures S15–S18 display representative regions of individual channels used for measurement of signal and background for each target.
- Table S15 displays estimated values for signal, background, and signal-to-background for each target.

Protocol: HCR 2°IHC (Section S4.2; without the optional autofluorescence bleaching protocol of Section S4.2.3) using unlabeled primary antibody probes and initiator-labeled secondary antibody probes with HCR signal amplification for all targets simultaneously.
Sample: FFPE C57BL/6 mouse brain section (coronal); thickness: 5 μm.
Microscopy: Epifluorescence.

Development • Supplementary information

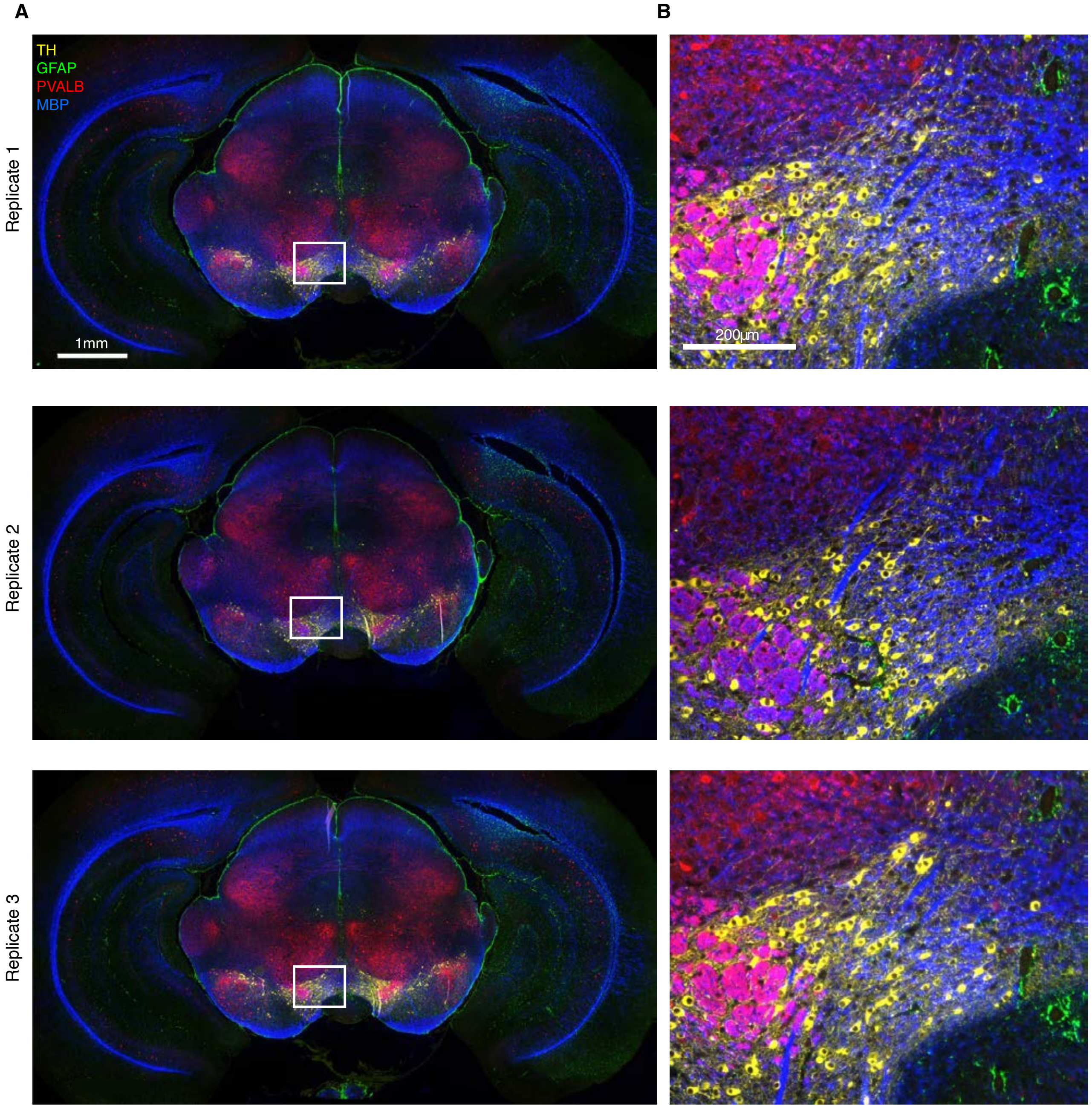

Figure S14. Replicates for 4-plex protein imaging using HCR 2°IHC in FFPE mouse brain sections (cf. Figures 3DE). (A) 4-channel epifluorescence images for 3 replicate FFPE mouse brain sections. (B) Zoom of the depicted region. Ch1: target protein TH (Alexa488). Ch2: target protein GFAP (Alexa546). Ch3: target protein PVALB (Alexa647). Ch4: target protein MBP (Alexa750). Sample: FFPE C57BL/6 mouse brain section (coronal); thickness: 5 μm.

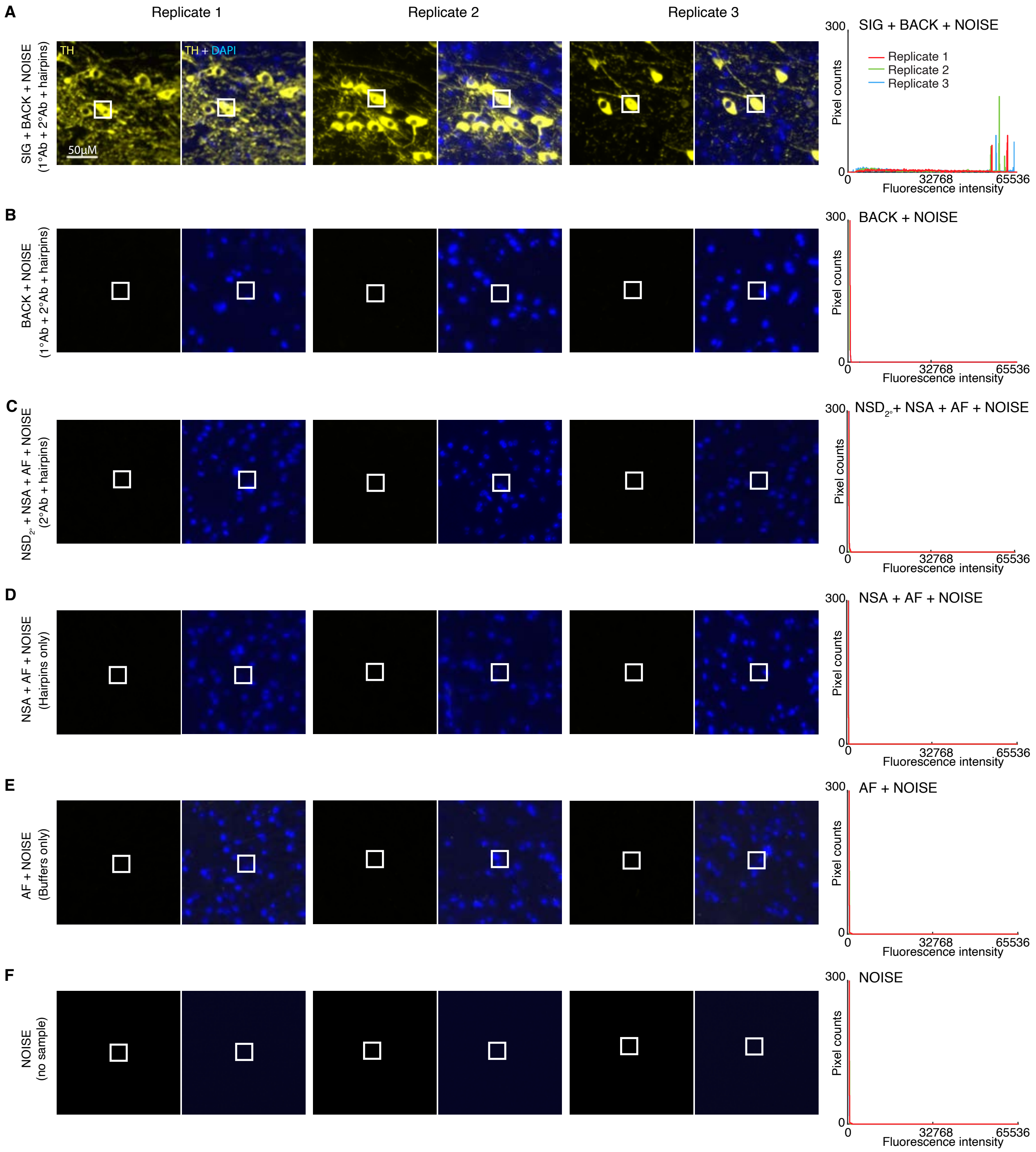

Figure S15. Measurement of signal, background, background components, and noise for protein target TH using HCR 2°IHC in FFPE mouse brain sections (cf. Figures 3DE). Use experiment of Type 1 in Table S8A (1°Ab probe + 2°Ab probe + hairpins) to measure (A) SIG+BACK+NOISE in a region of high expression and (B) BACK+NOISE in a region of no/low expression. (C) Use experiment of Type 4 in Table S8B (2°Ab probes + hairpins) to measure NSD_{2°+NSA+AF+NOISE in a region of high expression. (D) Use experiment of Type 2 in Table S8B (no probes, hairpins only) to measure NSA+AF+NOISE in a region of high expression. Use experiment of Type 3 in Table S8B (no probes, no hairpins) to measure (E) AF+NOISE in a region of high expression and (F) NOISE in a region with no sample. Left: epifluorescence image collected with the microscope exposure time optimized to avoid saturating SIG+BACK+NOISE pixels; DAPI channel facilitates placement of rectangles. Right: pixel intensity histograms for representative regions (three rectangles per experiment type for each of three replicate FFPE mouse brain sections). Ch1: target protein TH (Alexa488). Ch5: DAPI. Sample: FFPE C57BL/6 mouse brain section (coronal); thickness: 5 μm.

Development • Supplementary information

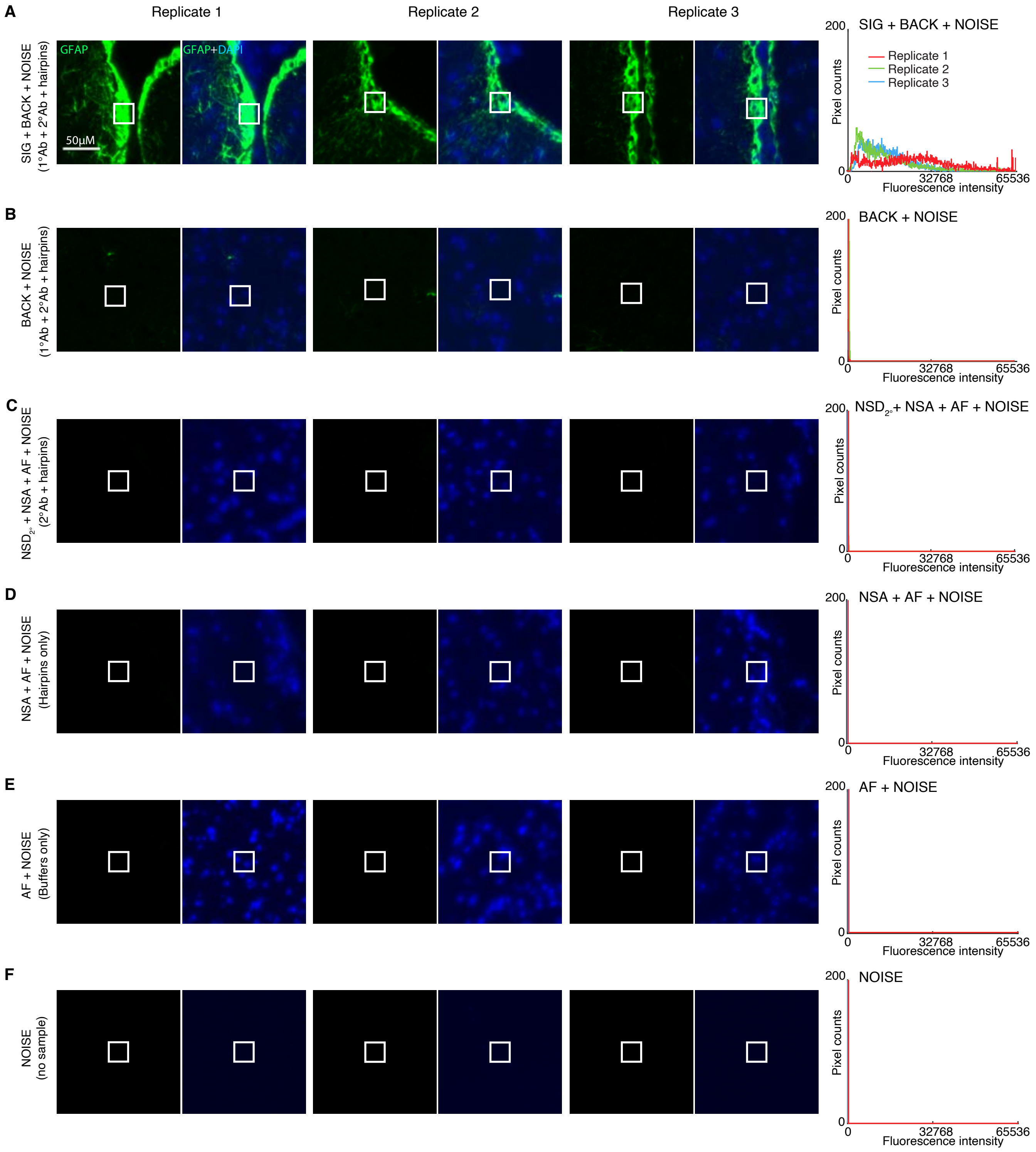

Figure S16. Measurement of signal, background, background components, and noise for protein target GFAP using HCR 2°IHC in FFPE mouse brain sections (cf. Figures 3DE). Use experiment of Type 1 in Table S8A (1°Ab probe + 2°Ab probe + hairpins) to measure (A) SIG+BACK+NOISE in a region of high expression and (B) BACK+NOISE in a region of no/low expression. (C) Use experiment of Type 4 in Table S8B (2°Ab probes + hairpins) to measure NSD_{2°+NSA+AF+NOISE in a region of high expression. (D) Use experiment of Type 2 in Table S8B (no probes, hairpins only) to measure NSA+AF+NOISE in a region of high expression. Use experiment of Type 3 in Table S8B (no probes, no hairpins) to measure (E) AF+NOISE in a region of high expression and (F) NOISE in a region with no sample. Left: epifluorescence image collected with the microscope exposure time optimized to avoid saturating SIG+BACK+NOISE pixels; DAPI channel facilitates placement of rectangles. Right: pixel intensity histograms for representative regions (three rectangles per experiment type for each of three replicate FFPE mouse brain sections). Ch2: target protein GFAP (Alexa546). Ch5: DAPI. Sample: FFPE C57BL/6 mouse brain section (coronal); thickness: 5 μm.

Development • Supplementary information

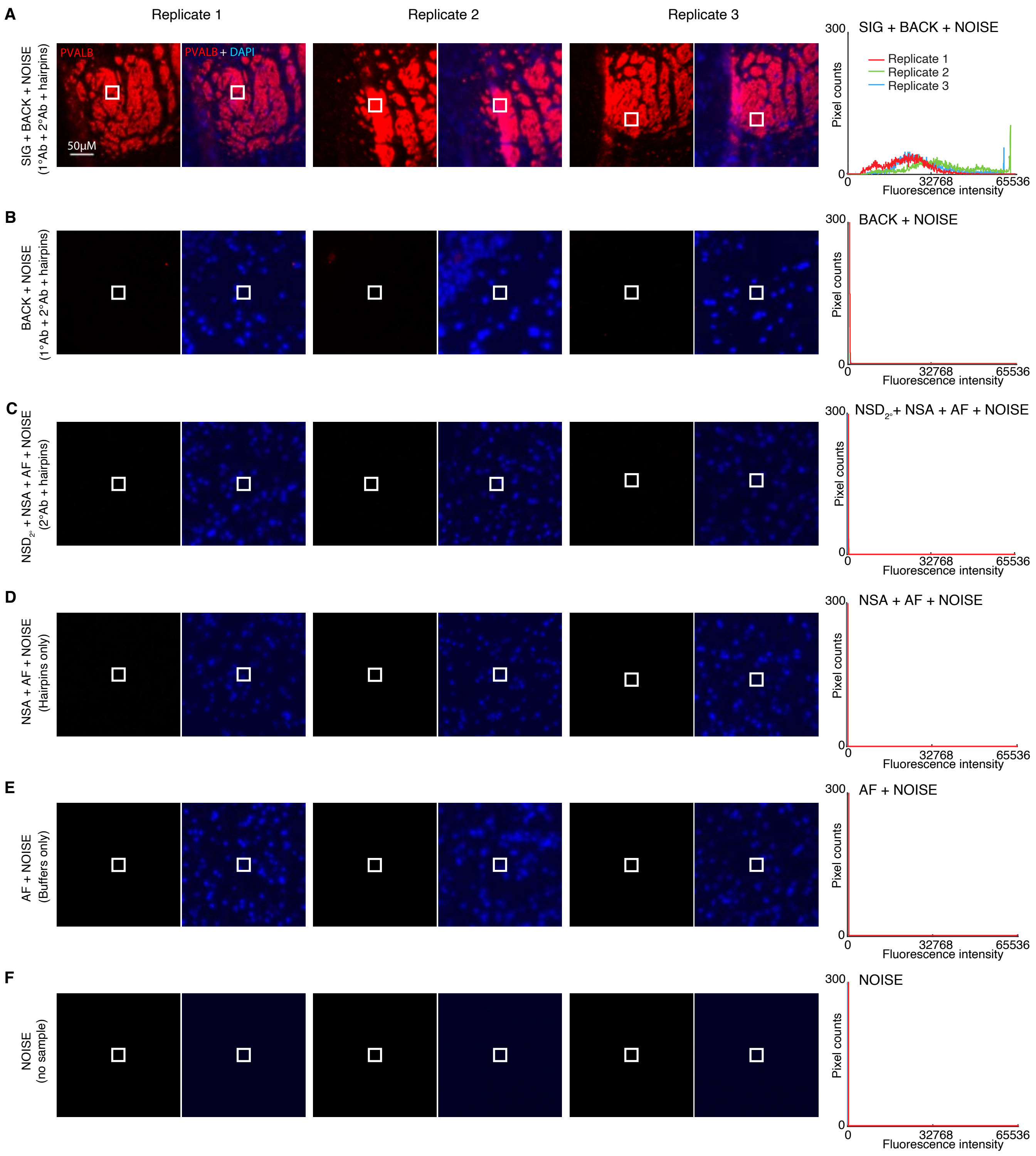

Figure S17. Measurement of signal, background, background components, and noise for protein target PVALB using HCR 2°IHC in FFPE mouse brain sections (cf. Figures 3DE). Use experiment of Type 1 in Table S8A (1°Ab probe + 2°Ab probe + hairpins) to measure (A) SIG+BACK+NOISE in a region of high expression and (B) BACK+NOISE in a region of no/low expression. (C) Use experiment of Type 4 in Table S8B (2°Ab probes + hairpins) to measure $NSD_{2°}$+NSA+AF+NOISE in a region of high expression. (D) Use experiment of Type 2 in Table S8B (no probes, hairpins only) to measure NSA+AF+NOISE in a region of high expression. Use experiment of Type 3 in Table S8B (no probes, no hairpins) to measure (E) AF+NOISE in a region of high expression and (F) NOISE in a region with no sample. Left: epifluorescence image collected with the microscope exposure time optimized to avoid saturating SIG+BACK+NOISE pixels; DAPI channel facilitates placement of rectangles. Right: pixel intensity histograms for representative regions (three rectangles per experiment type for each of three replicate FFPE mouse brain sections). Ch3: target protein PVALB (Alexa647). Ch5: DAPI. Sample: FFPE C57BL/6 mouse brain section (coronal); thickness: 5 μm.

Development • Supplementary information

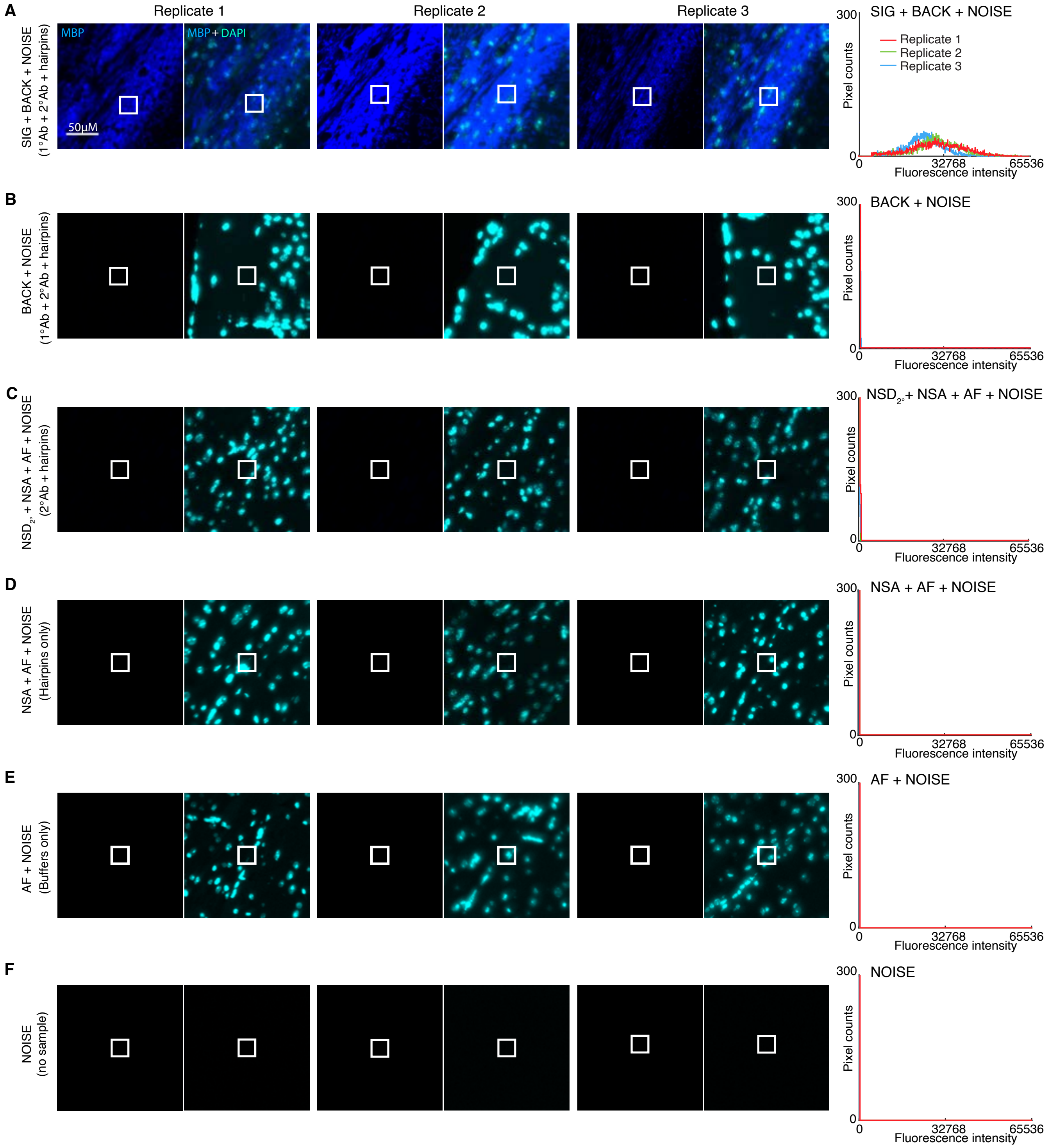

Figure S18. Measurement of signal, background, background components, and noise for protein target MBP using HCR 2°IHC in FFPE mouse brain sections (cf. Figures 3DE). Use experiment of Type 1 in Table S8A (1°Ab probe + 2°Ab probe + hairpins) to measure (A) SIG+BACK+NOISE in a region of high expression and (B) BACK+NOISE in a region of no/low expression. (C) Use experiment of Type 4 in Table S8B (2°Ab probes + hairpins) to measure $NSD_{2°}$+NSA+AF+NOISE in a region of high expression. (D) Use experiment of Type 2 in Table S8B (no probes, hairpins only) to measure NSA+AF+NOISE in a region of high expression. Use experiment of Type 3 in Table S8B (no probes, no hairpins) to measure (E) AF+NOISE in a region of high expression and (F) NOISE in a region with no sample. Left: epifluorescence image collected with the microscope exposure time optimized to avoid saturating SIG+BACK+NOISE pixels; DAPI channel facilitates placement of rectangles. Right: pixel intensity histograms for representative regions (three rectangles per experiment type for each of three replicate FFPE mouse brain sections). Ch4: target protein MBP (Alexa750). Ch5: DAPI. Sample: FFPE C57BL/6 mouse brain section (coronal); thickness: 5 μm.

Development • Supplementary information

	Quantity	Ch1: TH<br>B4-Alexa488	Ch2: GFAP<br>B1-Alexa546	Ch3: PVALB<br>B5-Alexa647	Ch4: MBP<br>B3-Alexa750	Reagents<br>1°Ab	Reagents<br>2°Ab-init	Expression<br>Hairpins	Figure<br>region	Figure<br>panel
**A**	SIG+BACK+NOISE	32 000 ± 3000	18 000 ± 6000	30 000 ± 8000	29 000 ± 3000	✓	✓	✓	high	A
	BACK+NOISE	640 ± 70	900 ± 200	340 ± 40	225 ± 12	✓	✓	✓	low/no	B
	NOISE	211 ± 16	114 ± 4	165 ± 2	116.5 ± 0.4	✓	✓	✓	no sample	F
	SIG	31 000 ± 3000	18 000 ± 6000	30 000 ± 8000	28 000 ± 3000					
	BACK	430 ± 80	800 ± 200	170 ± 40	109 ± 12					
	SIG/BACK	72 ± 14	23 ± 11	170 ± 60	260 ± 40					
**B**	$NSD_{2°}$+NSA+AF+NOISE	540 ± 30	185 ± 11	297 ± 11	240 ± 30		✓	✓	high	C
	NSA+AF+NOISE	500 ± 40	178 ± 2	237 ± 5	149 ± 4			✓	high	D
	AF+NOISE	490 ± 20	168 ± 6	217 ± 2	128 ± 3				high	E
	NSD	140 ± 50	700 ± 200	100 ± 40	110 ± 30					
	$NSD_{1°}$	110 ± 30	700 ± 200	40 ± 40	10 ± 30					
	$NSD_{2°}$	40 ± 40	< 17	60 ± 13	100 ± 30					
	NSA	10 ± 40	10 ± 6	19 ± 6	21 ± 5					
	AF	280 ± 30	54 ± 7	53 ± 3	12 ± 3					

Table S15. Estimated signal-to-background, background components, and noise for 4-plex protein imaging using HCR 2°IHC in FFPE mouse brain sections (cf. Figures 3DE). (A) Estimated signal-to-background (SIG/BACK) based on methods of Section S2.6.2. (B) Estimated background components (AF, NSA, $NSD_{1°}$, $NSD_{2°}$, NSD) based on methods of Section S2.6.3. Mean ± standard error of the mean, $N = 3$ replicate FFPE mouse brain sections. Analysis based on representative rectangular regions (examples depicted in Figures S15–S18).

Development • Supplementary information

S5.4 Protein imaging with high signal-to-background in whole-mount zebrafish embryos using HCR 2°IHC

Here, we demonstrate protein imaging in whole-mount vertebrate embryos using HCR 2°IHC (cf. Figure 3). The reagents for these 1-channel studies are:

- **Ch1:** target protein Elavl3/Elavl4, probe 1°mAb mouse IgG2b anti-Elavl3/Elavl4, probe 2°pAb goat anti-mouse IgG2b labeled with B1 initiator, amplifier B1-Alexa647.

Additional studies are presented as follows:

- Figure S19 displays confocal images depicting representative regions used for measurement of signal and background for $N = 3$ replicate whole-mount zebrafish embryos.
- Table S16 displays estimated values for signal, background, and signal-to-background.

Protocol: HCR 2°IHC (Section S4.4) using unlabeled primary antibody probes and initiator-labeled secondary antibody probes with HCR signal amplification.
Sample: Whole-mount zebrafish embryos; fixed 27 hpf.
Microscopy: Confocal.

Development • Supplementary information

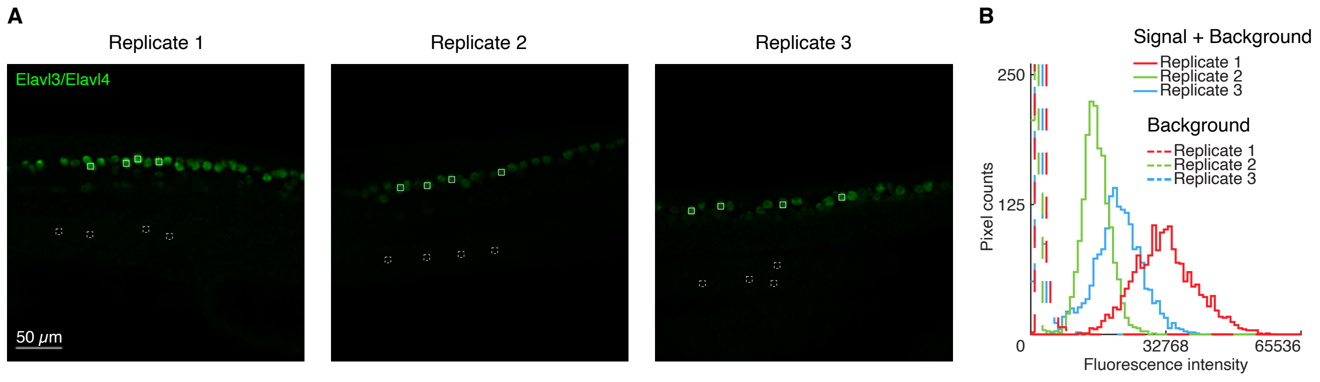

Figure S19. Measurement of signal and background for protein imaging using HCR 2°IHC in whole-mount zebrafish embryos (cf. Figure 3). Use experiment of Type 1 in Table S8A (1°Ab probe + 2°Ab probe + hairpins) to measure (A) SIG+BACK in regions of high expression and (B) BACK in regions of no/low expression. Confocal images. For each of three replicate embryos, a representative optical section was selected based on the expression depth of the target protein. Pixel size: 0.312×0.312 μm. (B) Pixel intensity histograms for SIG+BACK (pixels within solid boundary) and BACK (pixels within dashed boundary). Ch1: target protein Elavl3/Elavl4 (Alexa647). Sample: whole-mount zebrafish embryos; fixed 27 hpf.

Target protein	Fluorophore	BACK	SIG+BACK	SIG	SIG/BACK
Elavl3/Elavl4	Alexa647	1500 ± 300	$23\,000 \pm 4000$	$21\,000 \pm 4000$	15 ± 4

Table S16. Estimated signal-to-background for protein imaging using HCR 2°IHC in whole-mount zebrafish embryos (cf. Figure 3). Instrument noise is negligible using confocal microscopy so calculations use the approximation NOISE ≈ 0. Mean $\pm$ standard error of the mean, $N = 3$ replicate embryos. Analysis based on rectangular regions depicted in Figure S19 using methods of Section S2.6.2.

Development • Supplementary information

S5.5 Estimating HCR IHC polymer length (cf. Figures 2 and 3)

The gain due to HCR signal amplification corresponds to the mean HCR polymer length, which can be described in terms of the mean number of HCR hairpins per polymer. Here, we estimate HCR amplification gain in the context of HCR 1°IHC and HCR 2°IHC in both mammalian cells on a slide and FFPE mouse brain sections. For each method, we estimate:

- SIG using hairpins h1 and h2 so that HCR polymerization can proceed as normal.
- SIG_{h1} using only hairpin h1 so that each HCR initiator can bind only one HCR hairpin and polymerization cannot proceed.

The HCR amplification gain is then estimated as SIG/SIG_{h1}. Results are summarized in Table S17.

Method	Sample	Target protein	Amplifier	SIG	SIG_{h1}	SIG/SIG_{h1}	Table
HCR 1°ICC	mammalian cells on a slide	PCNA	B5-Alexa647	$39\,700 \pm 900$	290 ± 40	134 ± 17	S18
HCR 2°ICC	mammalian cells on a slide	PCNA	B5-Alexa647	$36\,000 \pm 2000$	280 ± 40	130 ± 20	S19
HCR 1°IHC	FFPE mouse brain sections	TH	B3-Alexa647	$12\,000 \pm 1100$	51 ± 4	230 ± 30	S20
HCR 2°IHC	FFPE mouse brain sections	TH	B3-Alexa647	$24\,000 \pm 3000$	53 ± 4	450 ± 50	S21

Table S17. Estimates of HCR amplification gain (mean polymer length) in the context of HCR 1°IHC and HCR 2°IHC in mammalian cells on a slide and FFPE mouse brain sections. Mean ± standard error of the mean. For mammalian cells on a slide, $N = 15$ representative rectangular regions (one rectangle in each of 5 individual cells on each of 3 replicate wells on a multi-well slide). For FFPE mouse brain sections, $N = 3$ replicate sections. Analysis based on representative rectangular regions (examples depicted in Figures S20-S23) using methods of Sections S2.6.2 and S2.6.4.

Development • Supplementary information

S5.5.1 HCR 1°ICC in mammalian cells on a slide

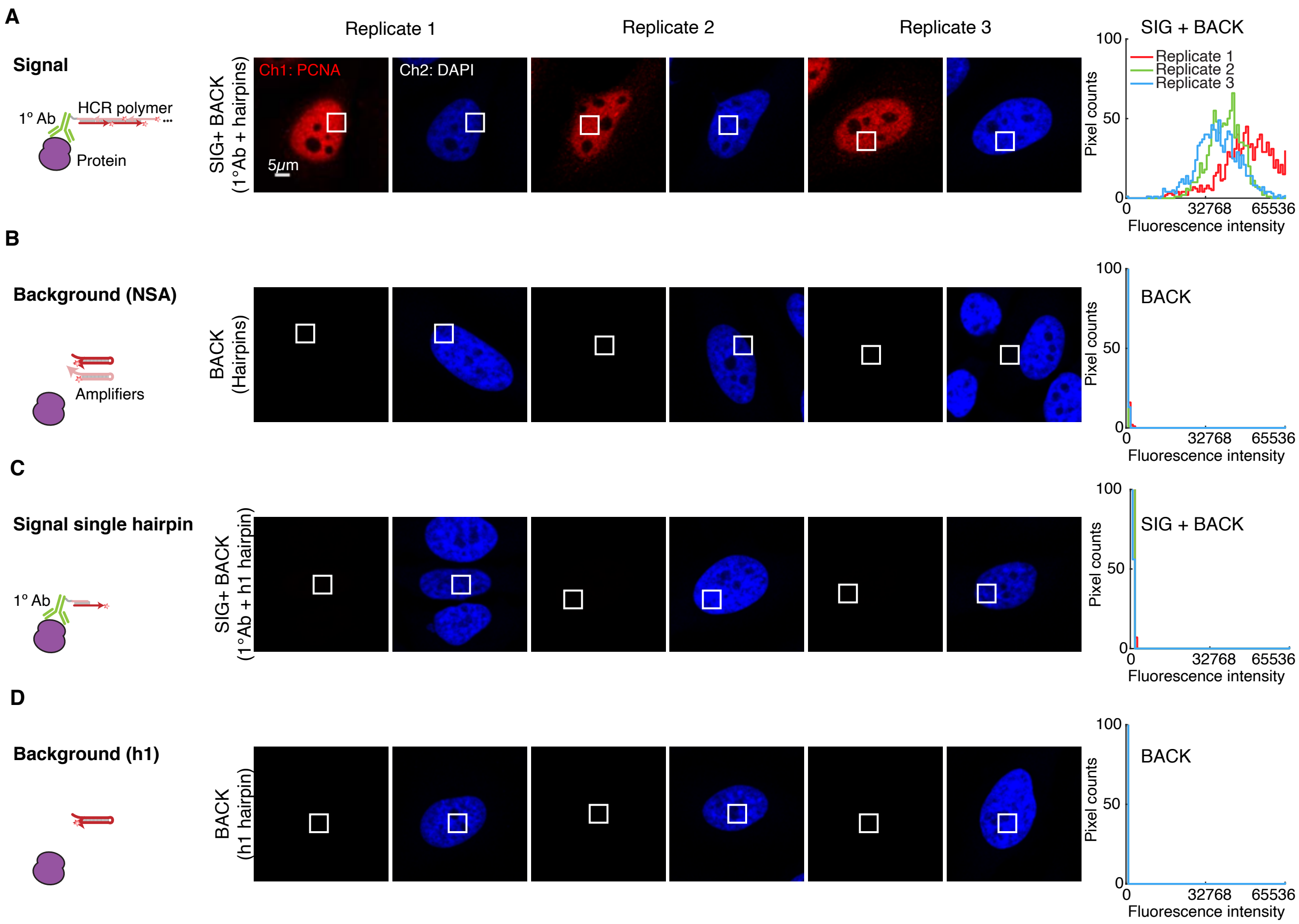

Figure S20. Measurement of HCR amplification gain (mean polymer length) for HCR 1°ICC in mammalian cells on a slide (cf. Figure 2C). (A,B) HCR 1°ICC. (C,D) HCR 1°ICC (h1 only). For each of 2 methods there are 2 rows: the top row measures SIG+BACK and the bottom row measures an approximation for BACK (see Table S18 for details on the background approximation used for each method). Left: Schematic of reagents used. Middle: confocal images collected with the microscope gain optimized to avoid saturating SIG+BACK pixels using HCR 1°ICC (panel A); DAPI channel facilitates placement of rectangles; single optical section. Pixel size: 0.198×0.198 μm. Right: pixel intensity histograms for representative regions (one rectangle in each of 5 individual cells in each of 3 replicate wells on a multi-well slide). Ch1: target protein PCNA (Alexa647). Ch2: DAPI. Sample: HeLa cells.

Development • Supplementary information

	Quantity	Reagents	Value		Figure
**A**	SIG+BACK	1°Ab-i1 + h1 + h2	39 700	± 900	S20A
	NSA+AF	h1 + h2	84	± 2	S20B
	SIG		39 600	± 900	
**B**	SIG_{h1}+BACK	1°Ab-i1 + h1	360	± 40	S20C
	NSA_{h1}+AF	h1	61.0	± 0.6	S20D
	SIG_{h1}		290	± 40	
**C**	SIG/SIG_{h1}		134	± 17	

Table S18. Estimate of HCR amplification gain (mean polymer length) for HCR 1°ICC in mammalian cells on a slide (cf. Figure 2C). (A) Estimated signal for HCR 1°ICC using HCR signal amplification (hairpins h1 and h2). The signal estimate SIG is calculated using the background approximation BACK $\approx$ NSA+AF. (B) Estimated signal SIG_{h1} without HCR signal amplification (using only hairpin h1 so that HCR polymerization is not possible and only a single h1 hairpin can bind initiator i1). The signal estimate SIG_{h1} is calculated using the background approximation $\text{BACK}_{\text{h1}} \approx \text{NSA}_{\text{h1}}$+AF. (C) Estimated HCR amplification gain SIG/SIG_{h1} (i.e., mean HCR polymer length). Instrument noise is negligible using confocal microscopy so calculations use the approximation NOISE ≈ 0. Mean $\pm$ standard error of the mean, $N = 15$ representative rectangular regions (one rectangle in each of 5 individual cells on each of 3 replicate wells on a multi-well slide). Analysis based on representative rectangular regions (examples depicted in Figures S20) using methods of Sections S2.6.2 and S2.6.4.

Development • Supplementary information

S5.5.2 HCR 2°ICC in mammalian cells on a slide

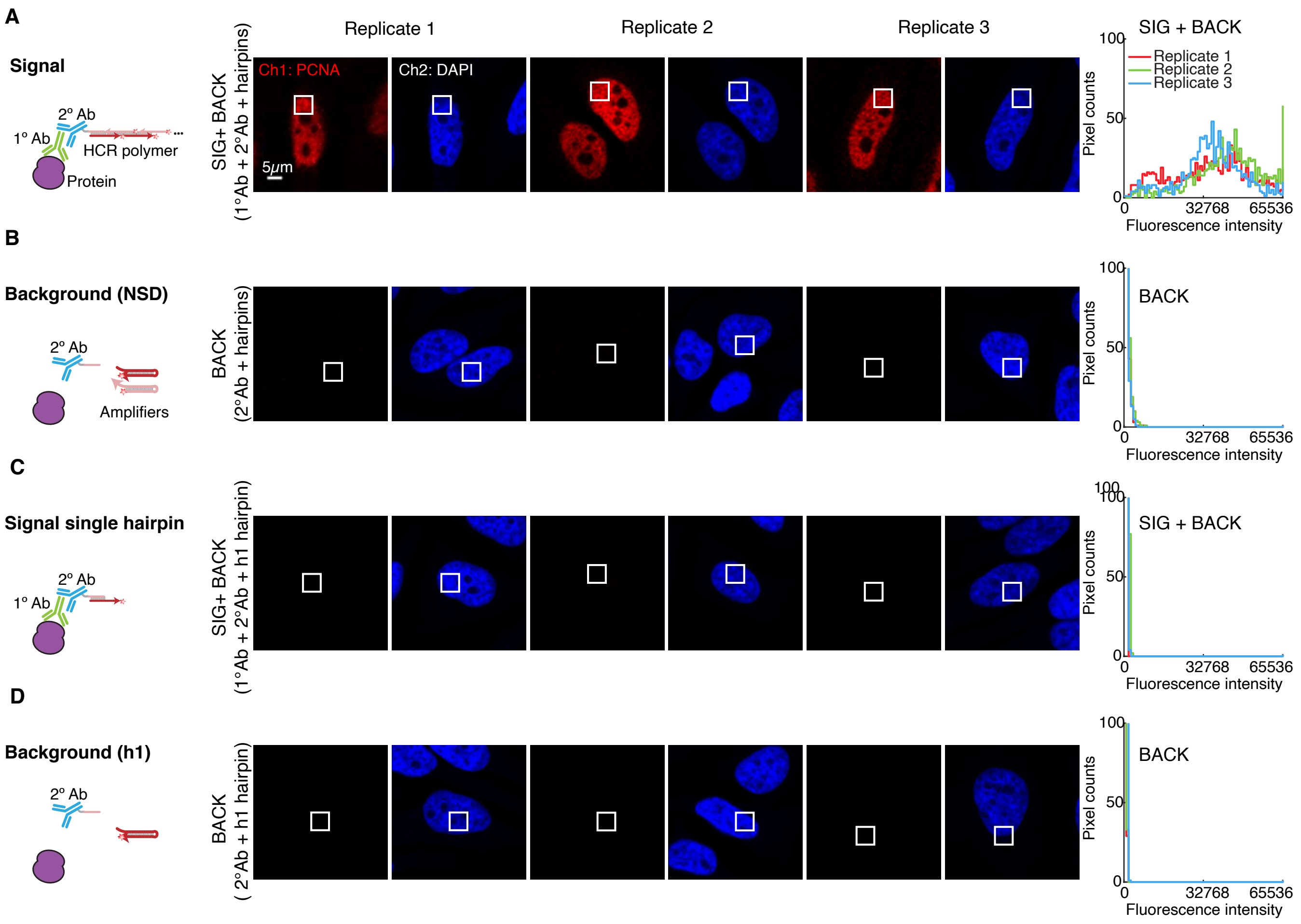

Figure S21. Measurement of HCR amplification gain (mean polymer length) for HCR 2°ICC in mammalian cells on a slide (cf. Figure 3C). (A,B) HCR 2°ICC. (C,D) HCR 2°ICC (h1 only). For each of 2 methods there are 2 rows: the top row measures SIG+BACK and the bottom row measures an approximation for BACK (see Table S19 for details on the background approximation used for each method). Left: Schematic of reagents used. Middle: confocal images collected with the microscope gain optimized to avoid saturating SIG+BACK pixels using HCR 2°ICC (panel A); DAPI channel facilitates placement of rectangles; single optical section. Pixel size: 0.198×0.198 μm. Right: pixel intensity histograms for representative regions (one rectangle in each of 5 individual cells in each of 3 replicate wells on a multi-well slide). Ch1: target protein PCNA (Alexa647). Ch2: DAPI. Sample: HeLa cells.

	Quantity	Reagents	Value		Figure
**A**	SIG+BACK	1°Ab + 2°Ab-i1 + h1 + h2	37 000	± 2000	S21A
	$NSD_{2°}$+NSA+AF	2°Ab-i1 + h1 + h2	460	± 20	S21B
	SIG		36 000	± 2000	
**B**	SIG_{h1}+$BACK_{h1}$	1°Ab + 2°Ab-i1 + h1	390	± 40	S21C
	$NSD_{2°h1}$+NSA_{h1}+AF	2°Ab-i1 + h1	119	± 6	S21D
	SIG_{h1}		280	± 40	
**C**	SIG/SIG_{h1}		130	± 20	

Table S19. Estimate of HCR amplification gain (mean polymer length) for HCR 2°ICC in mammalian cells on a slide (cf. Figure 3C). (A) Estimated signal for HCR 2°ICC using HCR signal amplification (hairpins h1 and h2). The signal estimate SIG is calculated using the background approximation BACK ≈ $NSD_{2°}$+NSA+AF. (B) Estimated signal SIG_{h1} without HCR signal amplification (using only hairpin h1 so that HCR polymerization is not possible and only a single h1 hairpin can bind initiator i1). The signal estimate SIG_{h1} is calculated using the background approximation BACK ≈ $NSD_{2°h1}$+NSA_{h1}+AF. (C) Estimated HCR amplification gain SIG/SIG_{h1} (i.e., mean HCR polymer length). Instrument noise is negligible using confocal microscopy so calculations use the approximation NOISE ≈ 0. Mean ± standard error of the mean, $N = 15$ representative rectangular regions (one rectangle in each of 5 individual cells on each of 3 replicate wells on a multi-well slide). Analysis based on representative rectangular regions (examples depicted in Figures S21) using methods of Sections S2.6.2 and S2.6.4.

Development • Supplementary information

S5.5.3 HCR 1°IHC in FFPE mouse brain sections

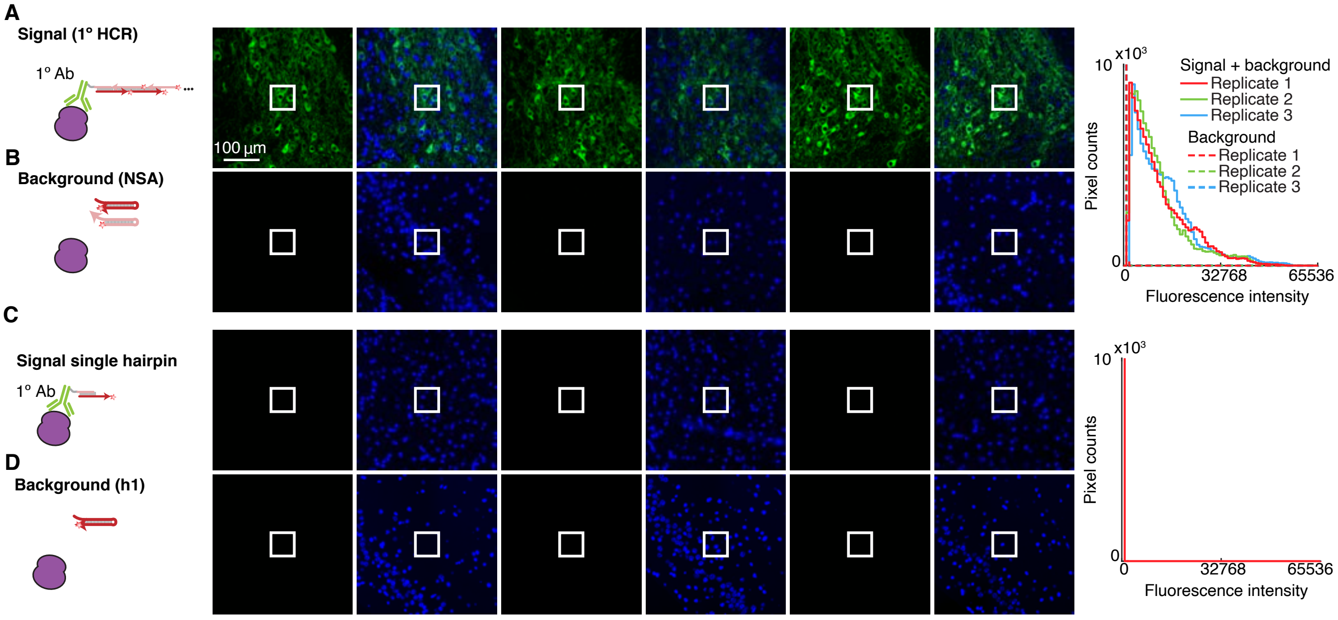

Figure S22. Measurement of HCR amplification gain (mean polymer length) for HCR 1°IHC in FFPE mouse brain sections (cf. Figures 2DE). (A,B) HCR 1°IHC. (C,D) HCR 1°IHC (h1 only). For each of 2 methods there are 2 rows: the top row measures SIG+BACK+NOISE in a region of high expression and the bottom row measures BACK+NOISE in a region of no/low expression. Left: Schematic of reagents used. Middle: epifluorescence images collected with the microscope exposure time optimized to avoid saturating SIG+BACK+NOISE pixels using HCR 1°IHC (panel A); DAPI channel facilitates placement of rectangles. Pixel size: 0.16×0.16 μm. Right: pixel intensity histograms for representative regions (three rectangles per experiment type for each of three replicate FFPE mouse brain sections). Ch1: target protein TH (Alexa647). Ch2: DAPI. Sample: FFPE C57BL/6 mouse brain section (coronal); thickness: 5 μm.

	Quantity	Expression region	Value		Figure
**A**	SIG+BACK	high	12 000	± 1100	S22A
	BACK	no/low	70	± 30	S22B
	SIG		11 900	± 1100	
**B**	SIG_{h1}+BACK	high	65	± 5	S22C
	BACK_{h1}	no/low	14	± 1	S22D
	SIG_{h1}		51	± 4	
**C**	$\text{SIG}/\text{SIG}_{\text{h1}}$		230	± 30	

Table S20. Estimate of HCR amplification gain (mean polymer length) for HCR 1°IHC in FFPE mouse brain sections (cf. Figure 2DE). (A) Estimated signal for HCR 1°IHC using HCR signal amplification (hairpins h1 and h2). SIG+BACK characterized for rectangular regions of high expression; BACK characterized for rectangular regions of no/low expression. (B) Estimated signal SIG_{h1} without HCR signal amplification (using only hairpin h1 so that HCR polymerization is not possible and only a single h1 hairpin can bind initiator i1). SIG_{h1}+BACK_{h1} characterized for rectangular regions of high expression; BACK_{h1} characterized for rectangular regions of no/low expression. (C) Estimated HCR amplification gain $\text{SIG}/\text{SIG}_{\text{h1}}$ (i.e., mean HCR polymer length). Mean ± standard error of the mean, $N = 3$ replicate FFPE mouse brain sections. Analysis based on representative rectangular regions (examples depicted in Figures S22) using methods of Sections S2.6.2 and S2.6.4.

S5.5.4 HCR 2°IHC in FFPE mouse brain sections

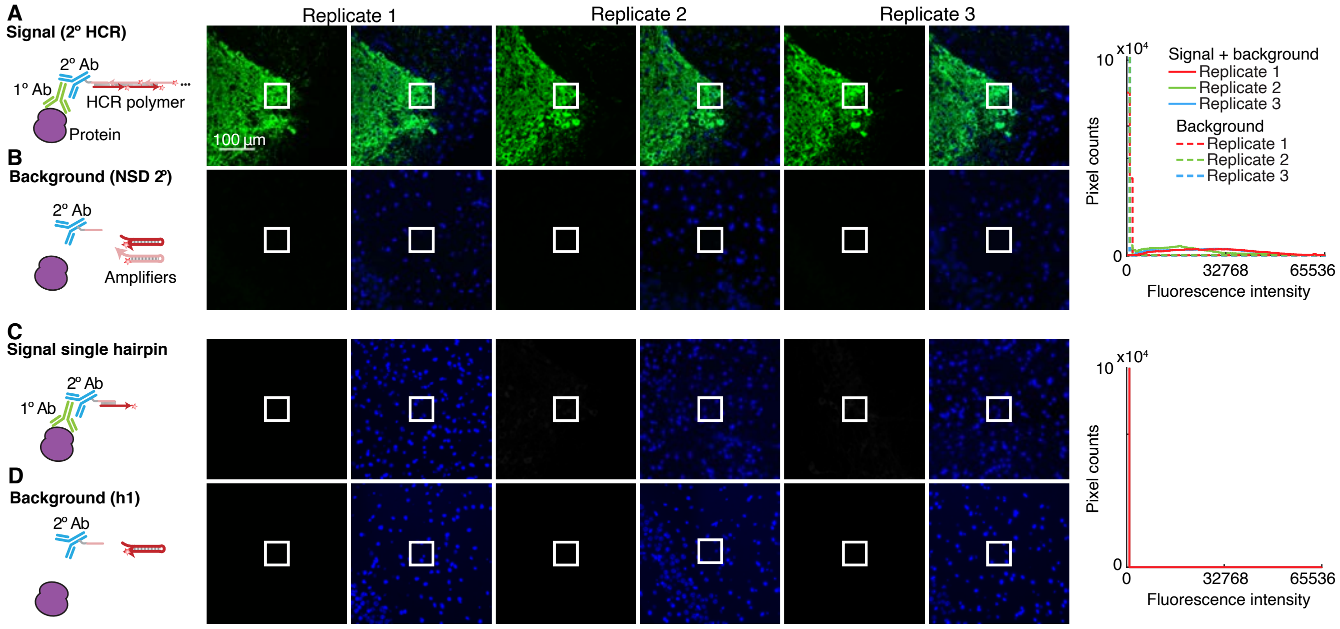

Figure S23. Measurement of HCR amplification gain (mean polymer length) for HCR 2°IHC in FFPE mouse brain sections (cf. Figure 3DE). (A,B) HCR 2°IHC. (C,D) HCR 2°IHC (h1 only). For each of 2 methods there are 2 rows: the top row measures SIG+BACK+NOISE in a region of high expression and the bottom row measures BACK+NOISE in a region of no/low expression. Left: Schematic of reagents used. Middle: epifluorescence images collected with the microscope exposure time optimized to avoid saturating SIG+BACK+NOISE pixels using HCR 1°IHC (panel A); DAPI channel facilitates placement of rectangles. Pixel size: 0.16×0.16 μm. Right: pixel intensity histograms for representative regions (three rectangles per experiment type for each of three replicate FFPE mouse brain sections). Ch1: target protein TH (Alexa647). Ch2: DAPI. Sample: FFPE C57BL/6 mouse brain section (coronal); thickness: 5 μm.

	Quantity	Expression region	Value		Figure
**A**	SIG+BACK	high	24 000	± 3000	S23A
	BACK	no/low	170	± 90	S23B
	SIG		24 000	± 3000	
**B**	$\mathrm{SIG_{h1}+BACK_{h1}}$	high	57	± 6	S23C
	$\mathrm{BACK_{h1}}$	no/low	4	± 3	S23D
	$\mathrm{SIG_{h1}}$		53	± 4	
**C**	$\mathrm{SIG/SIG_{h1}}$		450	± 50	

Table S21. Estimate of HCR amplification gain (mean polymer length) for HCR 2°IHC in FFPE mouse brain sections (cf. Figure 3DE). (A) Estimated signal for HCR 2°IHC using HCR signal amplification (hairpins h1 and h2). SIG+BACK characterized for rectangular regions of high expression; BACK characterized for rectangular regions of no/low expression. (B) Estimated signal $\mathrm{SIG_{h1}}$ without HCR signal amplification (using only hairpin h1 so that HCR polymerization is not possible and only a single h1 hairpin can bind initiator i1). $\mathrm{SIG_{h1}+BACK_{h1}}$ characterized for rectangular regions of high expression; $\mathrm{BACK_{h1}}$ characterized for rectangular regions of no/low expression. (C) Estimated HCR amplification gain $\mathrm{SIG/SIG_{h1}}$ (i.e., mean HCR polymer length). Mean ± standard error of the mean, $N = 3$ replicate FFPE mouse brain sections. Analysis based on representative rectangular regions (examples depicted in Figures S23) using methods of Sections S2.6.2 and S2.6.4.

Development • Supplementary information

S5.6 qHCR imaging: protein relative quantitation with subcellular resolution in an anatomical context (cf. Figure 4)

Additional studies are presented as follows:

- Section S5.6.1 presents a crowding study to test whether HCR amplification polymers for different targets interact within the cell.
- Section S5.6.2 provides replicates for redundant 2-channel imaging of target protein TH using HCR 1°IHC in FFPE mouse brain sections.
- Section S5.6.3 provides replicates for redundant 2-channel imaging of target proteins KRT17 and KRT19 using HCR 2°IHC in FFPE human breast tissue sections.

S5.6.1 Testing for a crowding effect

In order to perform multiplexed quantitative imaging using HCR, it is important that there is not a crowding effect in which amplification polymers tethered to one target molecule affect the signal intensity for a different target molecule. To test for a possible crowding effect, we imaged two target proteins (SC35 and PCNA) that are highly expressed in the nucleus individually (1-target studies) and also simultaneously (2-target studies) within HeLa cells. Figure S24 compares the signal intensity distributions for 1-target and 2-target studies, revealing similar intensity distributions whether targets were detected alone or together, suggesting that there is not a significant crowding effect (either antagonistic or synergistic). Intensities are plotted for subcellular $2.0\times2.0\times2.5$ μm voxels that fall entirely within a cell nucleus as determined based on a DAPI mask.

Development • Supplementary information

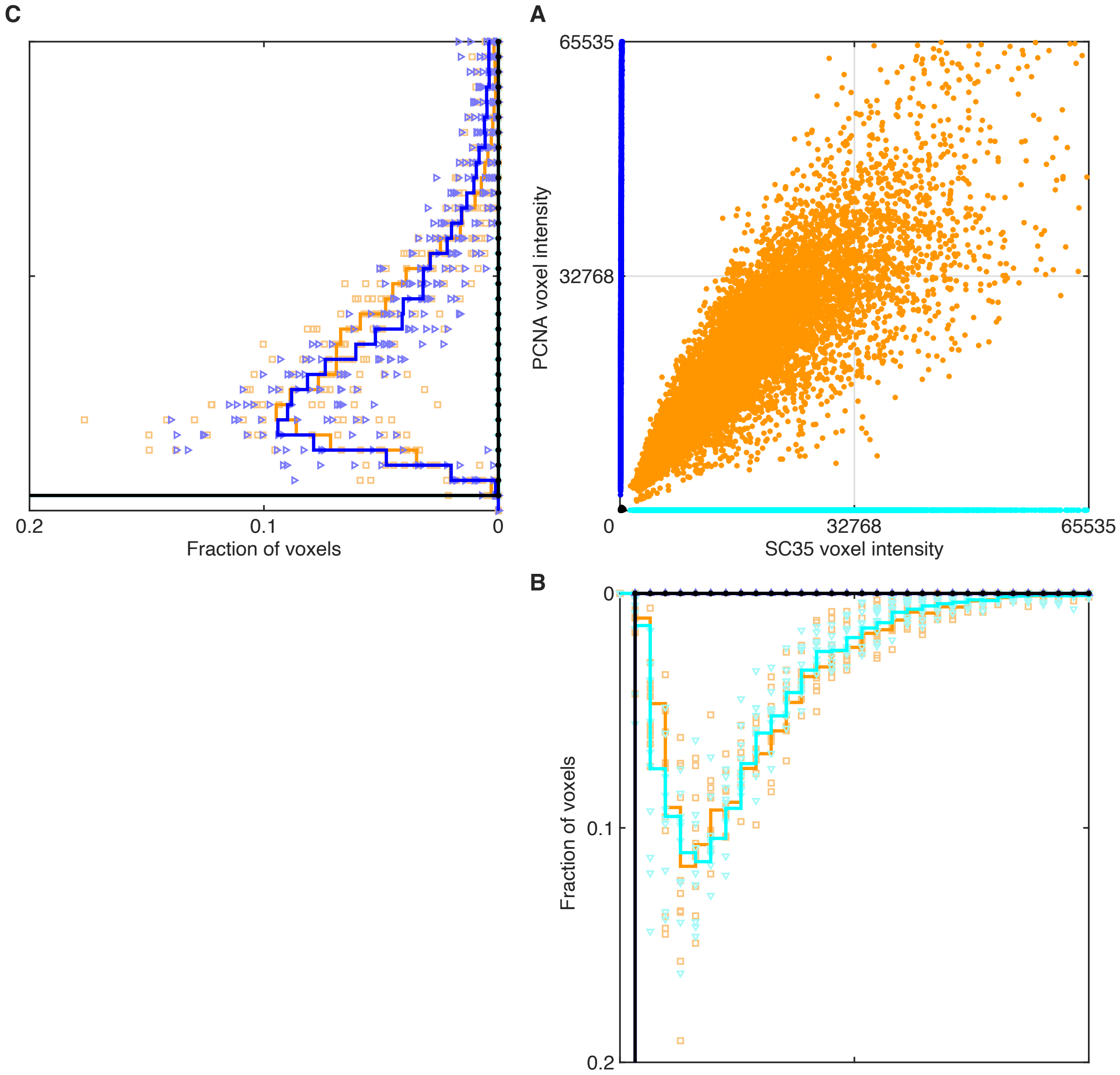

Figure S24. Comparison of fluorescence intensity distributions for one-target and two-target experiments. Detection of target proteins SC35 and PCNA with unlabeled primary antibodies and initiator-labeled secondary antibodies that trigger orthogonal spectrally-distinct HCR amplifiers. Ch1: Target protein SC35, probe 1°mAb mouse IgG1 anti-SC35, probe 2°pAb goat anti-mouse IgG1-B2, amplifier B2-Alexa546. Ch2: Target protein PCNA, probe 1°mAb mouse IgG2a anti-PCNA, probe 2°pAb goat anti-mouse IgG2a-B5, amplifier B5-Alexa647. (A) Raw voxel intensity scatter plot: SC35 vs PCNA. (B) Raw voxel intensity histogram for SC35. (C) Raw voxel intensity histogram for PCNA. In panels B and C, solid lines denote average histograms over cells in 10 replicate wells on a multi-well slide while symbols denote individual histograms (1 histogram per replicate well). Orange data: signal plus background for SC35 and PCNA (Figure S25). Cyan data: signal plus background for SC35 and background for PCNA (Figure S26). Blue data: background for SC35 and signal plus background for PCNA (Figure S27). Black data (near origin): background for SC35 and PCNA (Figure S28). Voxel size: 2.0×2.0×2.5 μm. Sample: HeLa cells.

Development • Supplementary information

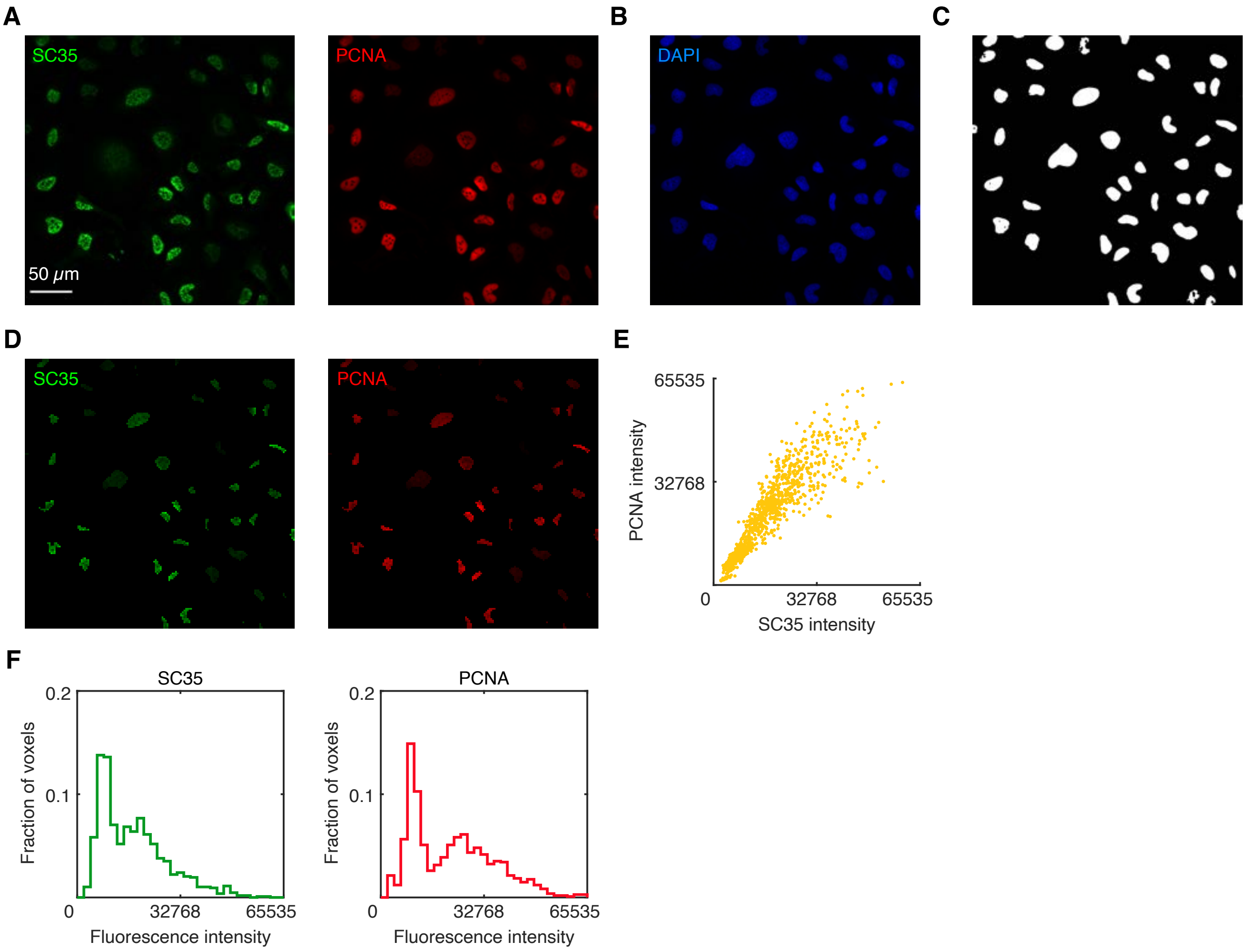

Figure S25. Characterizing signal plus background for SC35 and PCNA in a 2-target experiment. Detection of target proteins SC35 and PCNA with two unlabeled primary antibodies and two initiator-labeled secondary antibodies that trigger orthogonal spectrally-distinct HCR amplifiers. Ch1: Target protein SC35, probe 1°mAb mouse IgG1 anti-SC35, probe 2°pAb goat anti-mouse IgG1-B2, amplifier B2-Alexa546. Ch2: Target protein PCNA, probe 1°mAb mouse IgG2a anti-PCNA, probe 2°pAb goat anti-mouse IgG2a-B5, amplifier B5-Alexa647. (A) SC35 and PCNA channels from 3-channel confocal image; single optical section. Pixel size: 0.31×0.31 μm. (B) DAPI channel from 3-channel confocal image; single optical section. Pixel size: 0.31×0.31 μm. (C) Nuclear mask based on the DAPI staining of panel B; Gaussian blur filter followed by pixel thresholding. (D) Subcellular voxels falling entirely within the mask of panel C. Voxel size: $2.0\times2.0\times2.5$ μm. (E) Raw voxel intensity scatter plots for the masked regions of panel D representing signal plus background for SC35 and PCNA. (F) Raw voxel intensity histograms for the masked regions of panel D representing signal plus background for SC35 and PCNA. Same microscope settings used for all replicates in Figures S25–S28. Sample: HeLa cells.

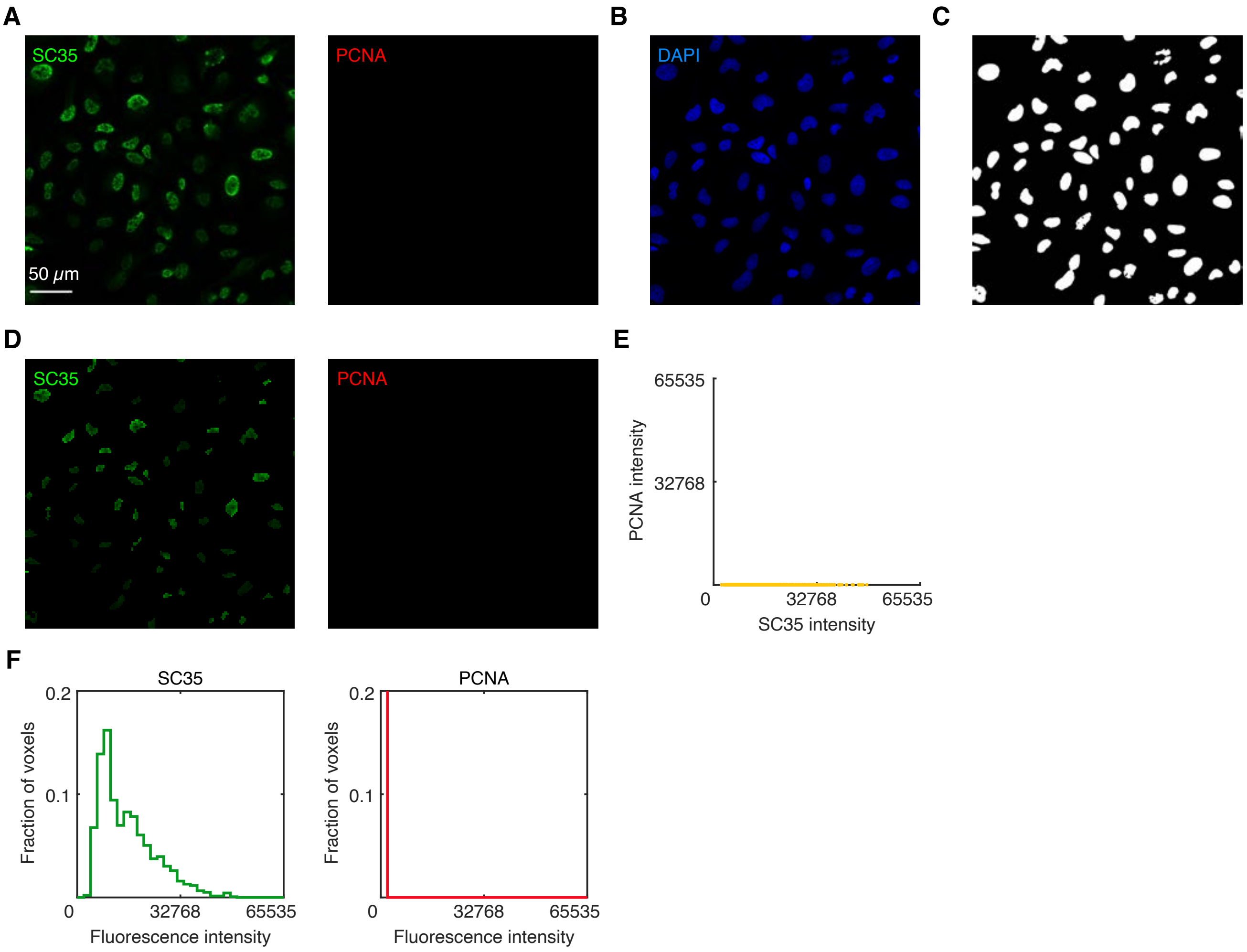

Figure S26. Characterizing signal plus background for SC35 in a 1-target experiment. Detection of target protein SC35 with an unlabeled primary antibody probe and initiator-labeled secondary antibody probe. Ch1: Target protein SC35, probe 1°mAb mouse IgG1 anti-SC35, probe 2°pAb goat anti-mouse IgG1-B2, amplifier B2-Alexa546. Ch2: no probe, no amplifier. (A) SC35 and PCNA channels from 3-channel confocal image; single optical section. Pixel size: 0.31×0.31 μm. (B) DAPI channel from 3-channel confocal image; single optical section. Pixel size: 0.31×0.31 μm. (C) Nuclear mask based on the DAPI staining of panel B; Gaussian blur filter followed by pixel thresholding. (D) Subcellular voxels falling entirely within the mask of panel C. Voxel size: $2.0 \times 2.0 \times 2.5$ μm. (E) Raw voxel intensity scatter plots for the masked regions of panel D representing signal plus background for SC35 and background for PCNA. (F) Raw voxel intensity histograms for the masked regions of panel D representing signal plus background for SC35 and background for PCNA. Same microscope settings used for all replicates in Figures S25–S28. Sample: HeLa cells.

Development • Supplementary information

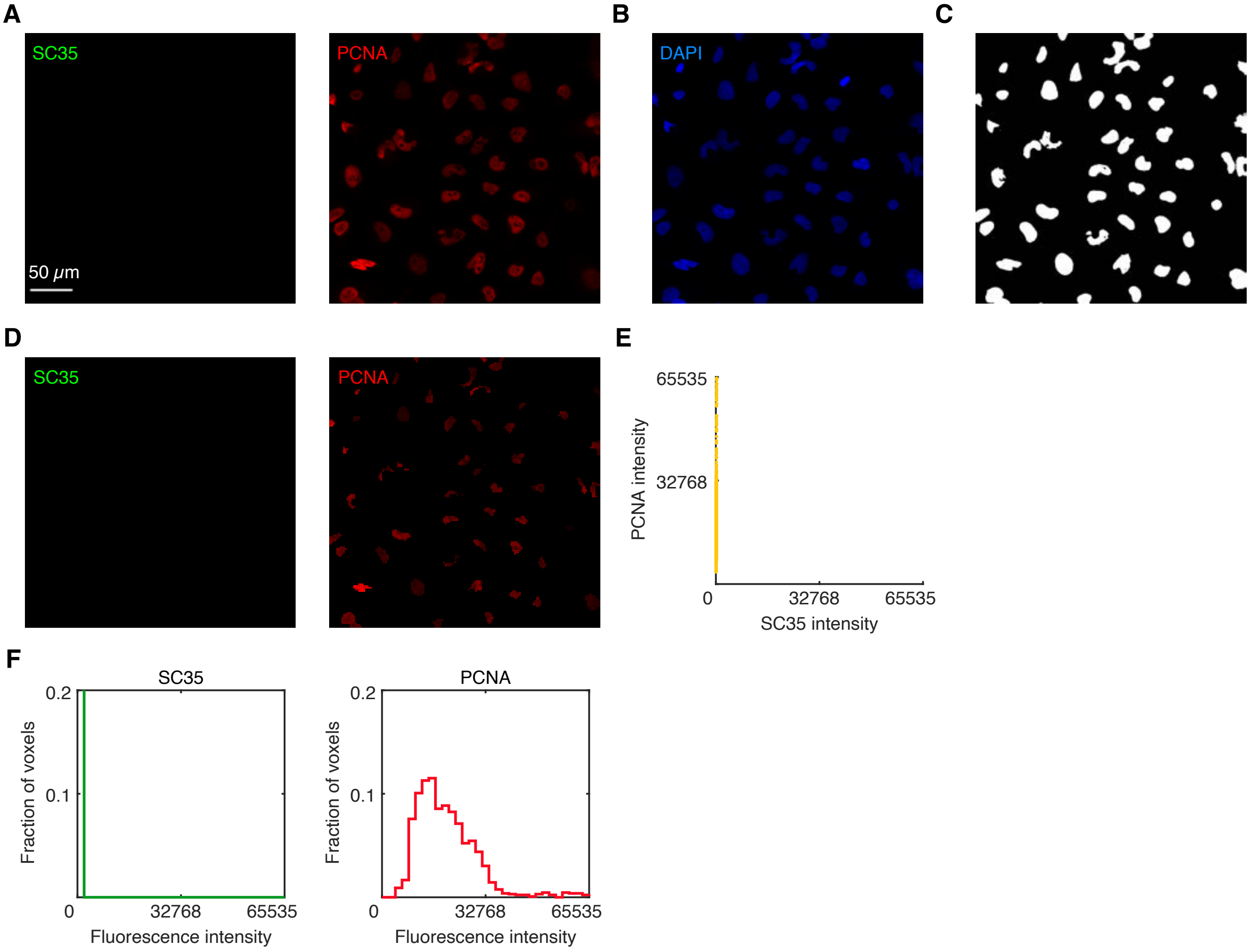

Figure S27. Characterizing signal plus background for PCNA in a 1-target experiment. Detection of target protein PCNA with an unlabeled primary antibody probe and initiator-labeled secondary antibody probe. Ch1: no probe, no amplifier. Ch2: Target protein PCNA, probe 1°mAb mouse IgG2a anti-PCNA, probe 2°pAb goat anti-mouse IgG2a-B5, amplifier B5-Alexa647. (A) SC35 and PCNA channels from 3-channel confocal image; single optical section. Pixel size: 0.31×0.31 μm. (B) DAPI channel from 3-channel confocal image; single optical section. Pixel size: 0.31×0.31 μm. (C) Nuclear mask based on the DAPI staining of panel B; Gaussian blur filter followed by pixel thresholding. (D) Subcellular voxels falling entirely within the mask of panel C. Voxel size: $2.0\times2.0\times2.5$ μm. (E) Raw voxel intensity scatter plots for the masked regions of panel D representing background for SC35 and signal plus background for PCNA. (F) Raw voxel intensity histograms for the masked regions of panel D representing background for SC35 and signal plus background for PCNA. Same microscope settings used for all replicates in Figures S25–S28. Sample: HeLa cells.

Development • Supplementary information

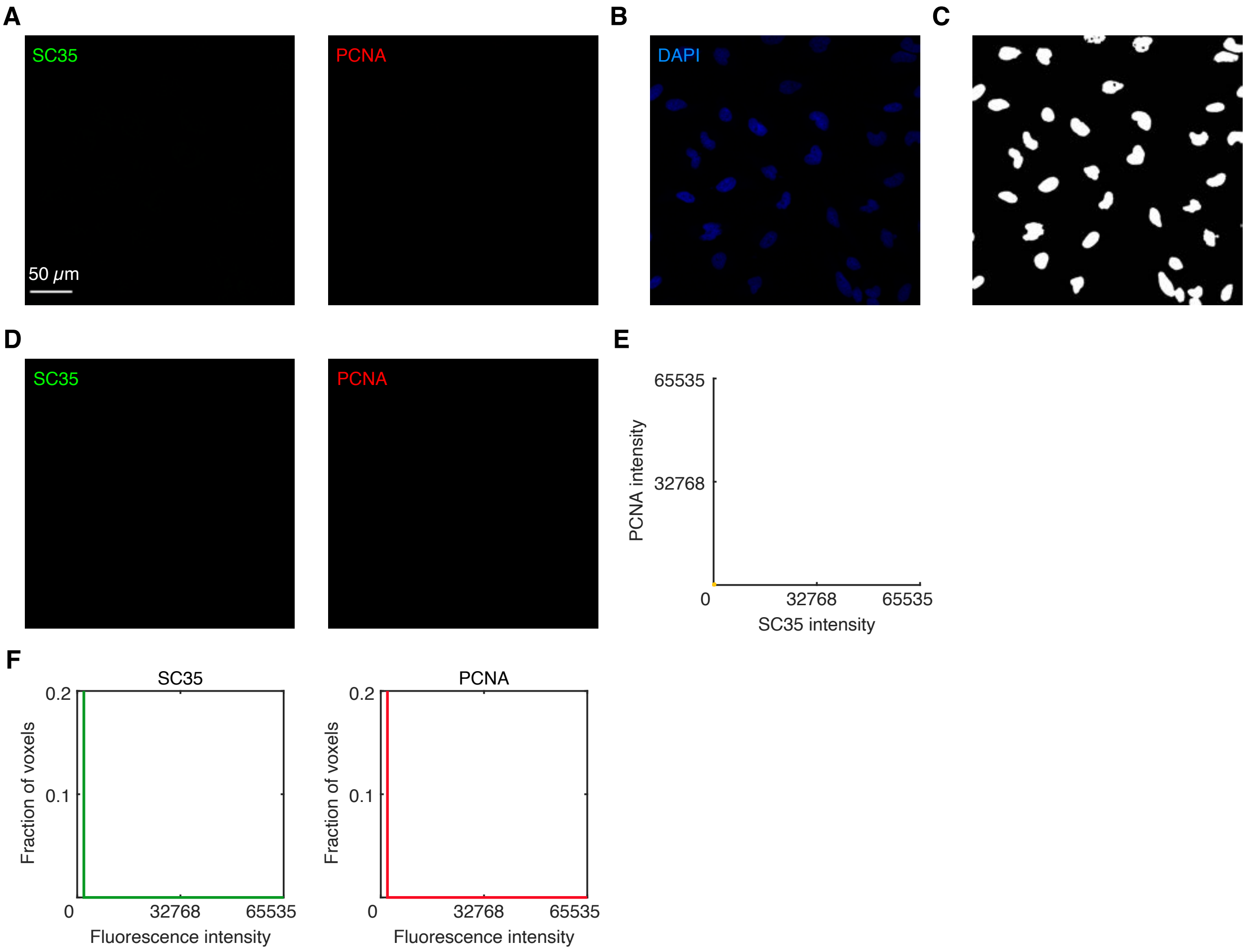

Figure S28. Characterizing background for SC35 and PCNA. Background is estimated using the standard HCR 2°ICC protocol omitting probes (BACK $\approx$ NSD_{2°+NSA+AF+NOISE; see Section S2.6 for definitions). (A) SC35 and PCNA channels from 3-channel confocal image; single optical section. Pixel size: 0.31$\times$0.31 μm. (B) DAPI channel from 3-channel confocal image; single optical section. Pixel size: 0.31$\times$0.31 μm. (C) Nuclear mask based on the DAPI staining of panel B; Gaussian blur filter followed by pixel thresholding. (D) Subcellular voxels falling entirely within the mask of panel C. Voxel size: 2.0$\times$2.0$\times$2.5 μm. (E) Raw voxel intensity scatter plots for the masked regions of panel D representing background for SC35 and PCNA. (F) Raw voxel intensity histograms for the masked regions of panel D representing background for SC35 and PCNA. Same microscope settings used for all replicates in Figures S25–S28. Sample: HeLa cells.

Development • Supplementary information

S5.6.2 Redundant 2-channel imaging of target protein TH using HCR 1°IHC in FFPE mouse brain sections

Here, we perform redundant 2-channel imaging of target protein TH using HCR 1°IHC in FFPE mouse brain sections. The target is detected with two initiator-labeled primary antibodies that bind different epitopes (e1 and e2) on the target protein and trigger orthogonal spectrally-distinct HCR amplifiers. The reagents for this 2-channel experiment are:

- **Ch1:** Target protein TH, probe 1°mAb$_{e1}$ EP1533Y anti-TH labeled with B1 initiator, amplifier B1-Alexa647.
- **Ch2:** Target protein TH, probe 1°mAb$_{e2}$ EP1532Y anti-TH labeled with B3 initiator, amplifier B3-Alexa750.

Additional studies are presented as follows:

- Figure S29 displays 2-plex images and 2-channel voxel intensity scatter plots for $N = 3$ replicate FFPE mouse brain sections.
- Table S22 displays estimated values for signal, background, and signal-to-background for each channel.

Protocol: HCR 1°IHC (Section S3.2; without the optional autofluorescence bleaching protocol of Section S3.2.3) using initiator-labeled primary antibody probes and HCR signal amplification.
Sample: FFPE C57BL/6 mouse brain section (coronal); thickness: 5 μm.
Microscopy: Epifluorescence.

Development • Supplementary information

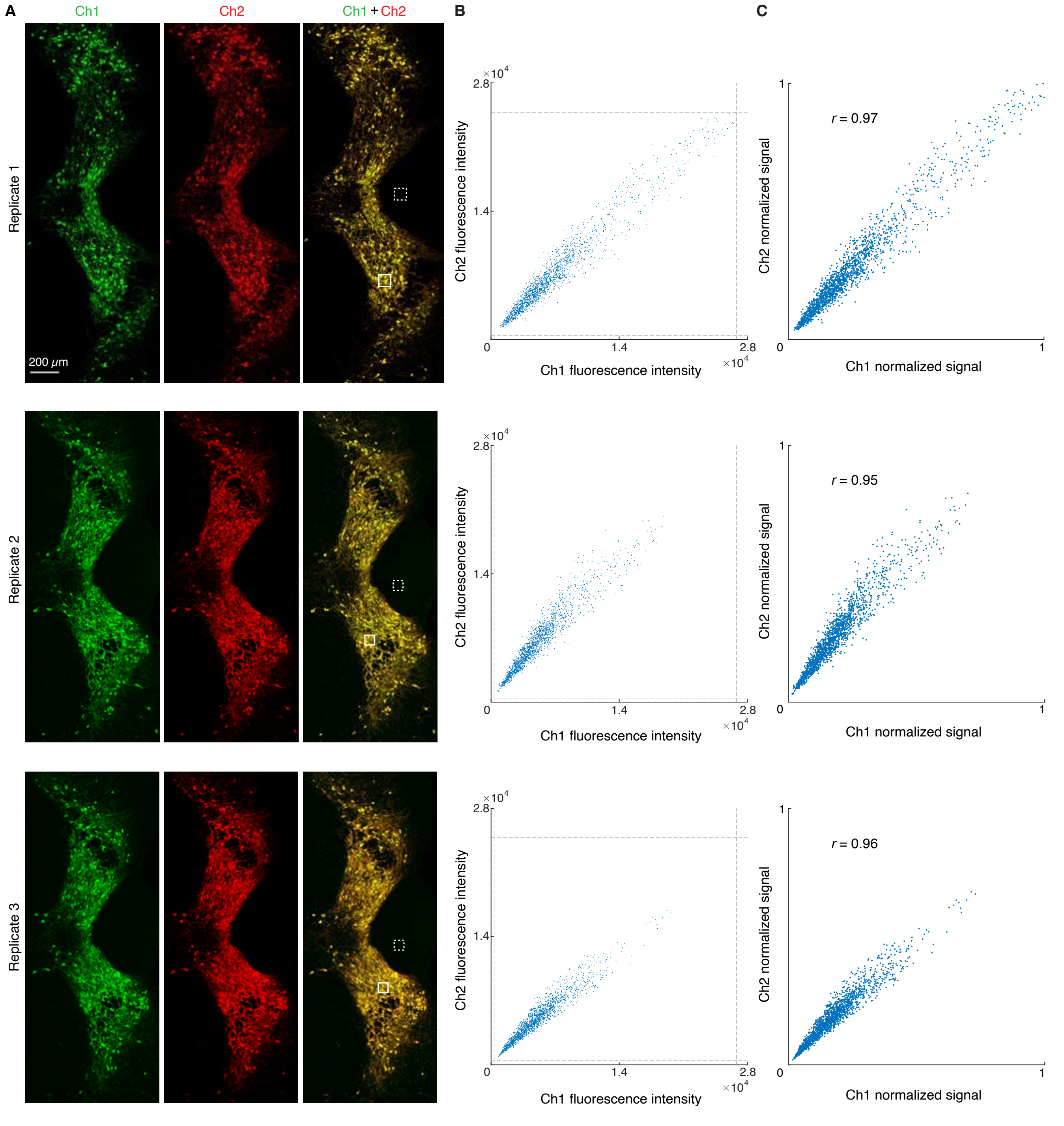

Figure S29. Redundant 2-channel detection of target protein TH in FFPE mouse brain sections (cf. Figure 4). (A) Epi-fluorescence images: individual channels and merge. Solid boundaries denote representative regions of high expression; dashed boundaries denote representative regions of no/low expression. Pixel size: 0.2×0.2 μm. (B) Raw voxel intensity scatter plots representing signal plus background plus noise for voxels within solid boundaries of panel A. Voxel size: 2.0×2.0 μm in 5 μm sections using epifluorescence microscopy. Dashed lines represent BOT and TOP values (Table S22) used to normalize data for panel C using methods of Section S2.6.5. (C) Normalized voxel intensity scatter plots representing estimated normalized signal (Pearson correlation coefficient, r). Ch1: target protein TH (Alexa647). Ch2: target protein TH (Alexa750). Sample: FFPE C57BL/6 mouse brain section (coronal); thickness: 5 μm.

Development • Supplementary information

Channel	Target protein	Fluorophore	BACK+NOISE	SIG+BACK+NOISE	SIG	SIG/BACK	BOT	TOP
Ch1	TH	Alexa647	253 ± 8	5600 ± 700	5400 ± 700	45 ± 9	253	26800
Ch2	TH	Alexa750	270 ± 20	6000 ± 1000	6000 ± 1000	41 ± 9	270	25000

Table S22. Estimated signal-to-background for redundant 2-channel detection of target protein TH in FFPE mouse brain sections (cf. Figure 4). Mean $\pm$ standard error, $N = 3$ replicate embryos. Analysis based on rectangular regions depicted in Figure S29A using methods of Section S2.6.2. BOT and TOP values used to calculate normalized voxel intensities for scatter plots of Figures 4C and S29C using methods of Section S2.6.5.

S5.6.3 Redundant 2-channel imaging of target proteins KRT17 and KRT19 using HCR 2°IHC in FFPE human breast tissue sections

Here, we perform redundant 2-channel imaging of target proteins KRT17 and KRT19 using HCR 2°IHC in FFPE human breast tissue sections. In each case, the target protein is detected with a 1°Ab probe, which is then redundantly detected by two batches of 2°Ab probes labeled with different HCR initiators that trigger orthogonal spectrally distinct HCR amplifiers. For redundant 2-channel imaging of KRT17, the reagents are:

- **Ch1:** Target protein KRT17, probe 1°pAb rabbit IgG anti-KRT17, probe 2°pAb donkey anti-rabbit labeled with B4 initiator, amplifier B4-Alexa546.
- **Ch2:** Target protein KRT17, probe 1°pAb rabbit IgG anti-KRT17, probe 2°pAb donkey anti-rabbit labeled with B3 initiator, amplifier B3-Alexa647.

For redundant 2-channel imaging of KRT19, the reagents are:

- **Ch1:** Target protein KRT19, probe 1°mAb mouse IgG1 anti-KRT19, probe 2°pAb goat anti-mouse IgG1 labeled with B2 initiator, amplifier B2-Alexa546.
- **Ch2:** Target protein KRT19, probe 1°mAb mouse IgG1 anti-KRT19, probe 2°pAb goat anti-mouse IgG1 labeled with B5 initiator, amplifier B5-Alexa647.

Additional studies are presented as follows:

- Figure S30 displays 2-plex images and 2-channel voxel intensity scatter plots for target protein KRT17 for $N = 3$ replicate FFPE human breast tissue sections.
- Figure S31 displays 2-plex images and 2-channel voxel intensity scatter plots for target protein KRT19 for $N = 3$ replicate FFPE human breast tissue sections.
- Table S23 displays values used for signal normalization in Figures S30 and S31.
- Figure S32 display representative regions used for measurement of signal and background for protein targets KRT17 and KRT19.
- Table S24 displays estimated values for signal, background, and signal-to-background for each channel for both KRT17 and KRT19.

Protocol: HCR 2°IHC (Section S4.3) using unlabeled primary antibody probes and initiator-labeled secondary antibody probes with HCR signal amplification.
Sample: FFPE human breast tissue section; thickness: 5 μm.
Microscopy: Confocal.

Development • Supplementary information

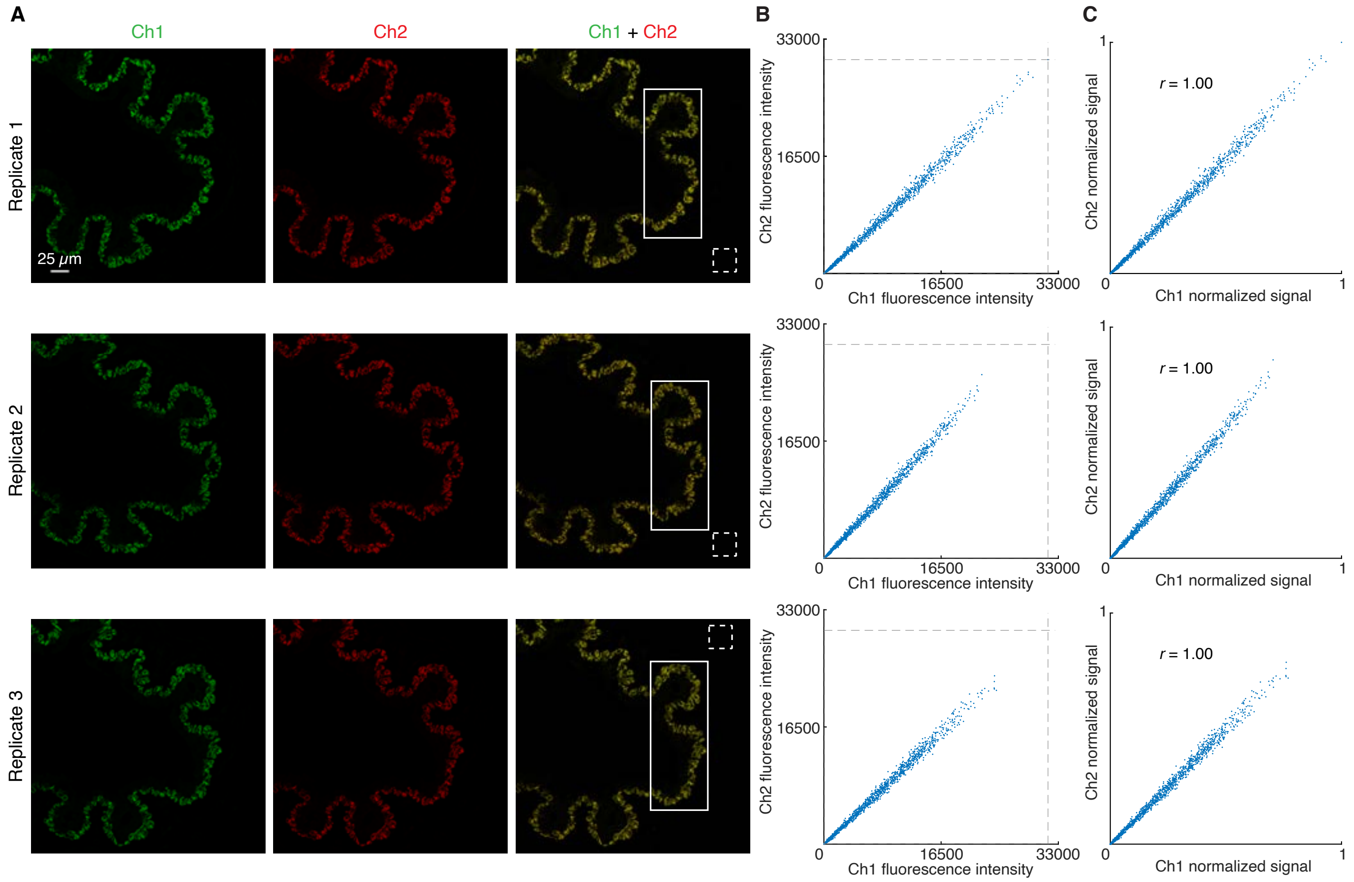

Figure S30. Redundant 2-channel detection of target protein KRT17 in FFPE human breast tissue sections (cf. Figure 4). (A) Confocal images: individual channels and merge; single optical section. Solid boundaries denote regions of variable expression; dashed boundaries denote regions of no/low expression. Pixel size: 0.312×0.312 μm. Sample: FFPE human breast tissue section; thickness: 5 μm. (B) Raw voxel intensity scatter plots representing signal plus background for voxels within solid boundaries of panel A. Voxel size: $2.0 \times 2.0 \times 2.5$ μm. Dashed lines represent BOT and TOP values (Table S23) used to normalize data for panel C using methods of Section S2.6.5. (C) Normalized voxel intensity scatter plots representing estimated normalized signal (Pearson correlation coefficient, r). Ch1: KRT17 (Alexa546). Ch2: KRT17 (Alexa647). Sample: FFPE human breast tissue section; thickness: 5 μm.

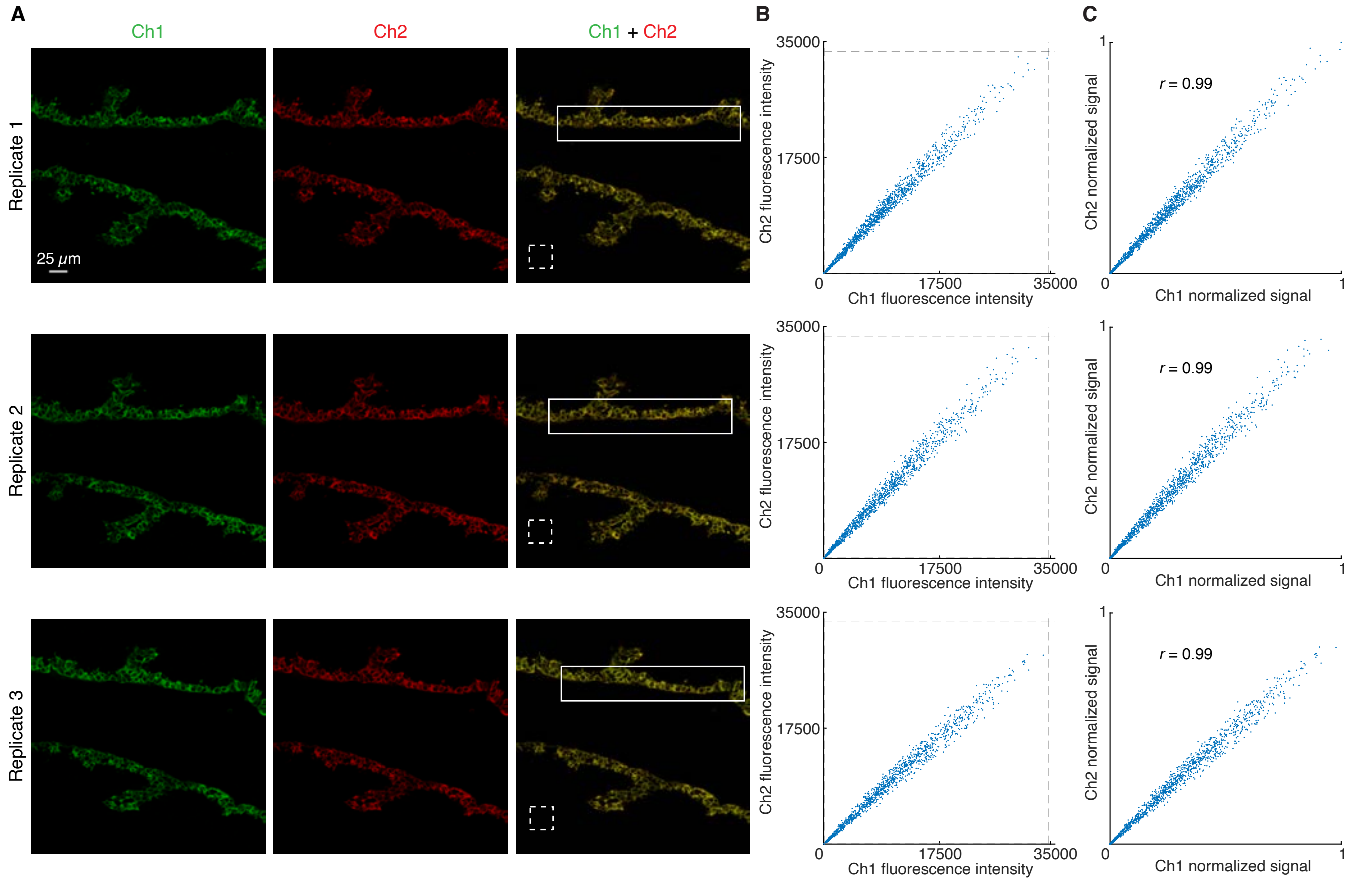

Figure S31. Redundant 2-channel detection of target protein KRT19 in FFPE human breast tissue sections (cf. Figure 4). (A) Confocal images: individual channels and merge; single optical section. Solid boundaries denote regions of variable expression; dashed boundaries denote regions of no/low expression. Pixel size: 0.312×0.312 μm. Sample: FFPE human breast tissue section. Thickness: 5 μm. (B) Raw voxel intensity scatter plots representing signal plus background for voxels within solid boundaries of panel A. Voxel size: 2.0×2.0×2.5 μm. Dashed lines represent BOT and TOP values (Table S23) used to normalize data for panel C using methods of Section S2.6.5. (C) Normalized voxel intensity scatter plots representing estimated normalized signal (Pearson correlation coefficient, r). Ch1: KRT19 (Alexa546). Ch2: KRT19 (Alexa647). Sample: FFPE human breast tissue section; thickness: 5 μm.

Channel	Target protein	Fluorophore	BOT	TOP
Ch1	KRT17	Alexa546	70	31500
Ch2	KRT17	Alexa647	70	30100
Ch1	KRT19	Alexa546	70	34000
Ch2	KRT19	Alexa647	63	33500

Table S23. BOT and TOP values used to calculate normalized voxel intensities for scatter plots of Figures 4C, S30C, and S31C using methods of Section S2.6.5. Analysis based on rectangular regions depicted in Figures S30A and S31A.

Development • Supplementary information

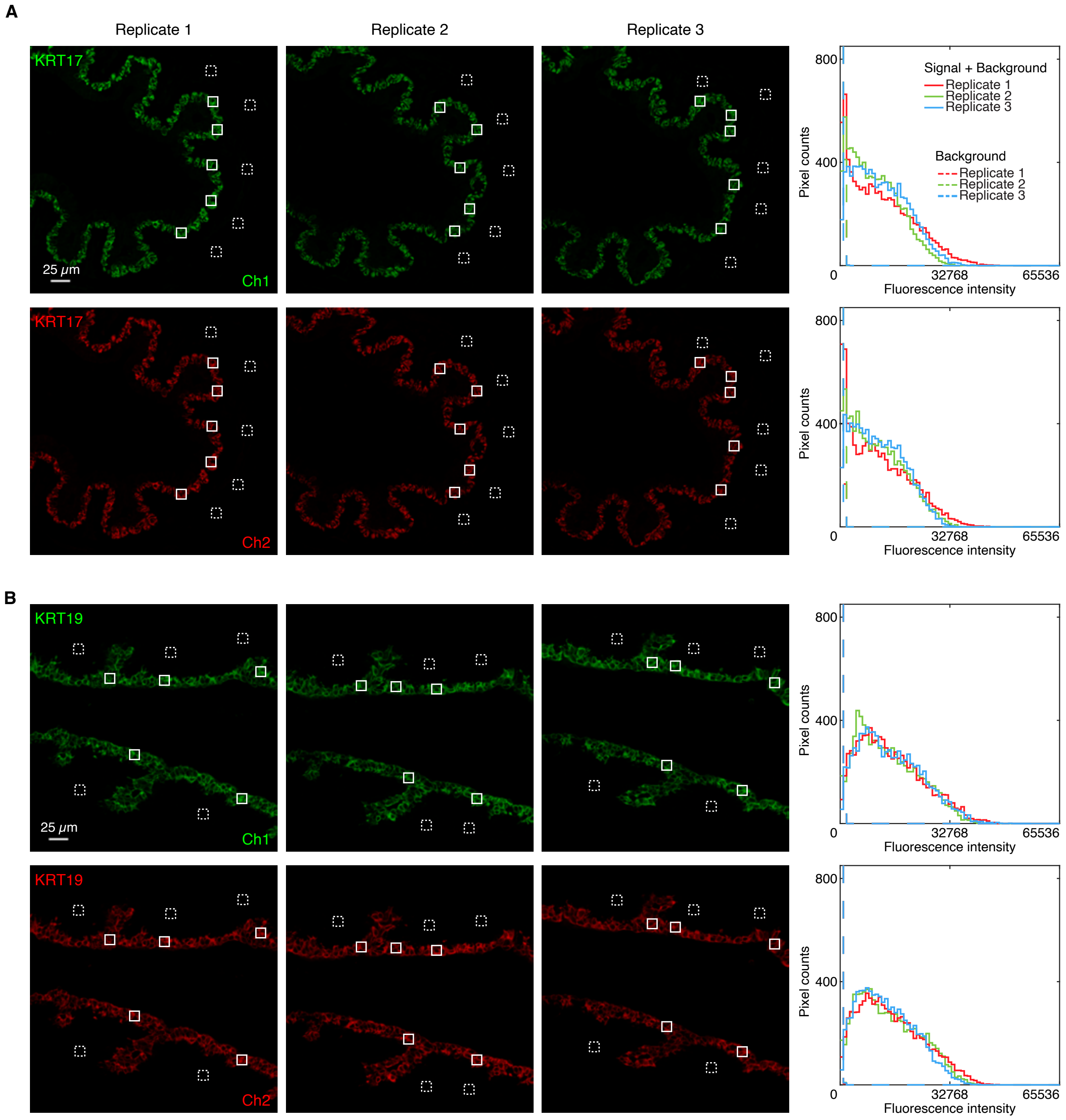

Figure S32. Measurement of signal and background for redundant 2-channel detection of target proteins KRT17 and KRT19 in FFPE human breast tissue sections (cf. Figure 4). (A) Redundant 2-channel imaging of target protein KRT17. Ch1: Alexa546. Ch2: Alexa647. (B) Redundant 2-channel imaging of target protein KRT19. Ch1: Alexa546. Ch2: Alexa647. Left: confocal images; single optical section. Solid boundaries denote representative regions of high expression; dashed boundaries denote representative regions of no/low expression. Right: pixel intensity histograms for the depicted representative regions. Sample: FFPE human breast tissue section; thickness: 5 μm.

Development • Supplementary information

Channel	Target protein	Fluorophore	BACK	SIG+BACK	SIG	SIG/BACK
Ch1	KRT17	Alexa546	110 ± 30	$11\,200 \pm 600$	$11\,000 \pm 600$	100 ± 30
Ch2	KRT17	Alexa647	100 ± 20	$11\,000 \pm 500$	$10\,900 \pm 500$	110 ± 30
Ch1	KRT19	Alexa546	99 ± 6	$14\,000 \pm 1000$	$14\,000 \pm 1000$	140 ± 10
Ch2	KRT19	Alexa647	83 ± 4	$14\,200 \pm 900$	$14\,100 \pm 900$	170 ± 10

Table S24. Estimated signal-to-background for redundant 2-channel detection of target proteins KRT17 and KRT19 in FFPE human breast tissue sections (cf. Figure 4). Instrument noise is negligible using confocal microscopy so calculations use the approximation NOISE ≈ 0. Mean $\pm$ standard error, $N = 3$ replicate FFPE human breast tissue sections. Analysis based on rectangular regions depicted in Figure S32 using methods of Section S2.6.2.

Development • Supplementary information

S5.7 Replicates and signal-to-background measurements for simultaneous multiplexed protein and mRNA imaging using HCR 1°IHC and HCR RNA-ISH (cf. Figure 5)

S5.7.1 Mammalian cells on a slide

For 4-plex simultaneous protein and mRNA imaging using HCR 1°ICC + HCR RNA-ISH in mammalian cells on a slide, the 5 channels are (2 RNAs + 2 proteins + DAPI):

- **Ch1:** Target protein PCNA, probe 1°mAb mouse IgG2a anti-PCNA labeled with B5 initiator, amplifier B5-Alexa488.
- **Ch2:** Target protein HSP60, probe 1°mAb rabbit IgG anti-HSP60 labeled with B3 initiator, amplifier B3-Alexa546.
- **Ch3:** Target RNA *U6*, probe set with 2 split-initiator probe pairs, amplifier B1-Alexa594.
- **Ch4:** Target mRNA *ACTB*, probe set with 10 split-initiator pairs, amplifier B2-Alexa647.
- **Ch5:** DAPI.

Additional studies are presented as follows:

- Figure S33 displays 4-plex images for $N = 3$ replicate wells on a multi-well slide (cf. Figure 5B).
- Figure S34 displays representative regions of individual channels used for measurement of signal and background for each target.
- Table S25 displays estimated values for signal, background, and signal-to-background for each target.

Protocol: Simultaneous HCR 1°ICC + HCR RNA-ISH (Section S3.1) using initiator-labeled primary antibody probes for protein targets, split-initiator DNA probes for RNA targets, and simultaneous HCR signal amplification for all targets.
Sample: HeLa cells.
Microscopy: Confocal.

Development • Supplementary information

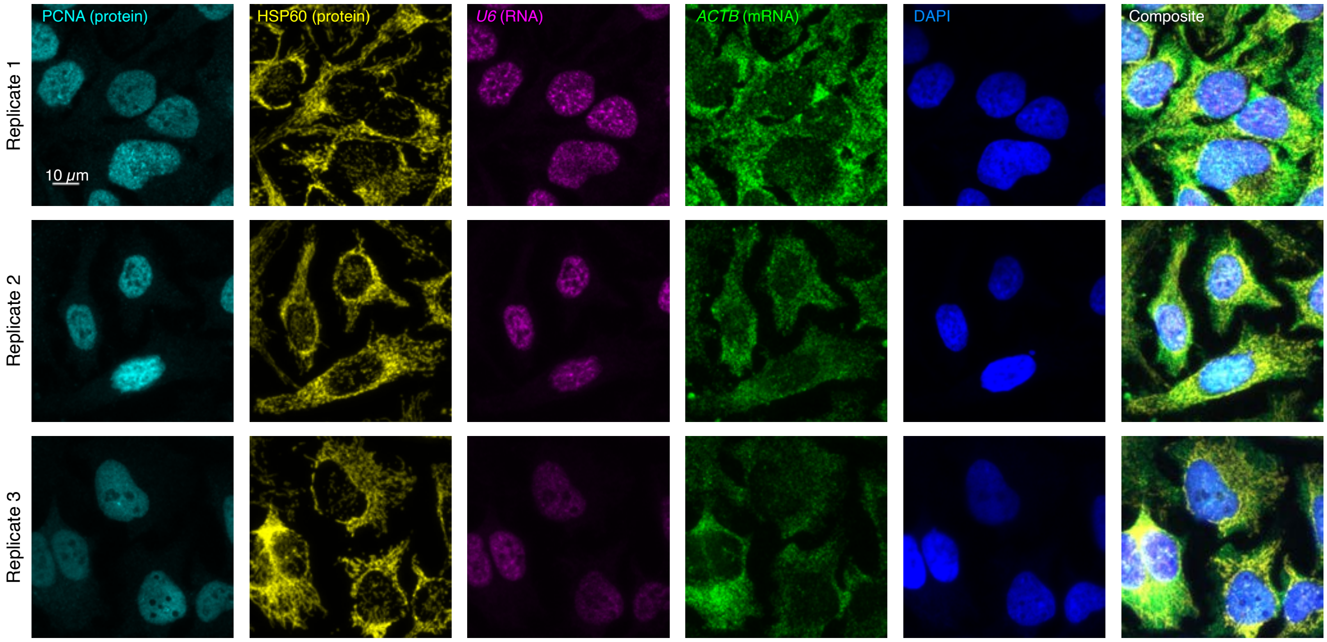

Figure S33. Replicates for 4-plex simultaneous protein and mRNA imaging using HCR 1°ICC and HCR RNA-ISH in mammalian cells on slides (cf. Figures 5B). 5-channel confocal images for 3 replicate wells on a multi-well slide; maximum intensity z-projection. Ch1: target protein PCNA (Alexa488). Ch2: target protein HSP60 (Alexa546). Ch3: target RNA *U6* (Alexa594). Ch4: target mRNA *ACTB* (Alexa647). Ch5: DAPI. Sample: HeLa cells.

Development • Supplementary information

Channel	Target	Type	Fluorophore	BACK	SIG+BACK	SIG	SIG/BACK
Ch1	PCNA	protein	Alexa488	276 ± 4	26 600 ± 1700	26 400 ± 1700	95 ± 6
Ch2	HSP60	protein	Alexa546	134.4 ± 1.3	37 100 ± 1300	37 000 ± 1300	280 ± 10
Ch3	*U6*	RNA	Alexa594	100.9 ± 0.7	2150 ± 130	2050 ± 130	20.3 ± 1.3
Ch4	*ACTB*	mRNA	Alexa647	182 ± 8	20 000 ± 2000	19 000 ± 2000	107 ± 12

Table S25. Estimated signal-to-background for 4-plex simultaneous protein and mRNA imaging using HCR 1°ICC and HCR RNA-ISH in mammalian cells on a slide (cf. Figure 5B). The signal estimate SIG is calculated using the background approximation BACK ≈ NSA+AF. Instrument noise is negligible using confocal microscopy so calculations use the approximation NOISE ≈ 0. Mean ± standard error of the mean, $N = 15$ representative rectangular regions (one rectangle in each of 5 individual cells in each of 3 replicate wells on a multi-well slide). Analysis based on representative rectangular regions (examples depicted in Figure S34) using methods of Section S2.6.2.

Development • Supplementary information

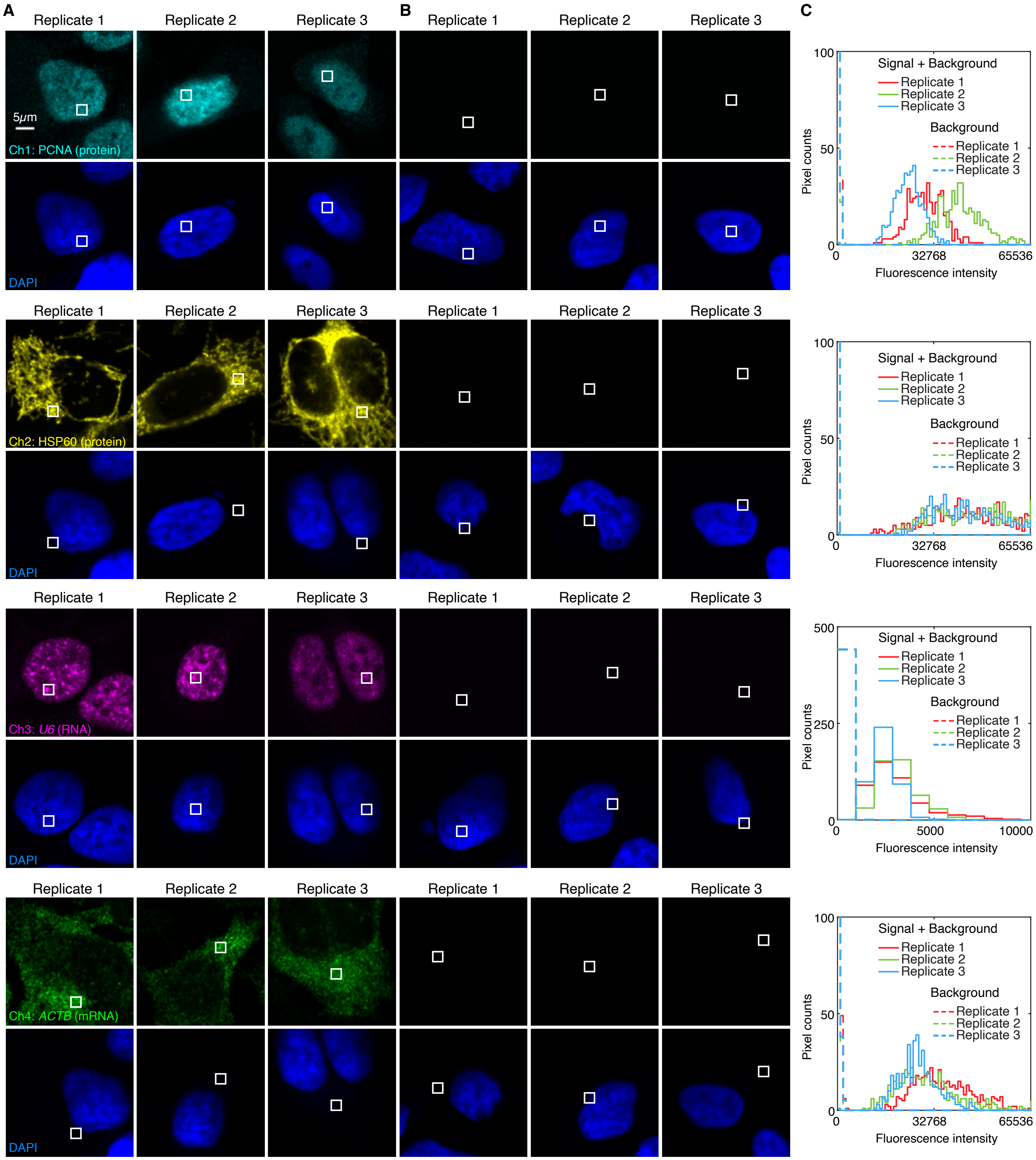

Figure S34. Measurement of signal and background for 4-plex simultaneous protein and mRNA imaging using HCR 1°ICC and HCR RNA-ISH in mammalian cells on a slide (cf. Figure 5B). (A) Use experiment of Type 1 in Table S7A (1°Ab probe + hairpins) to measure SIG+BACK in a region of high expression. (B) Use experiment of Type 2 in Table S7B (hairpins only) to measure NSA+AF in a region of maximum background. (C) Pixel intensity histograms for representative regions (one rectangle in each of 5 individual cells in each of 3 replicate wells on a multi-well slide). Confocal images collected with the microscope gain optimized to avoid saturating SIG+BACK pixels; DAPI channel facilitates placement of representative rectangles; single optical section. Ch1: target protein PCNA (Alexa488). Ch2: target protein HSP60 (Alexa546). Ch3: target RNA *U6* (Alexa594). Ch4: target mRNA *ACTB* (Alexa647). Ch5: DAPI. Sample: HeLa cells.

Development • Supplementary information

S5.7.2 FFPE mouse brain sections

For 4-plex simultaneous protein and mRNA imaging using HCR 1°IHC + HCR RNA-ISH in FFPE mouse brain sections, the 5 channels are (2 proteins + 2 RNAs + DAPI):

- **Ch1:** Target protein TH, probe 1°Ab rabbit IgG monoclonal anti-TH labeled with B3 initiator, amplifier B3-Alexa488.
- **Ch2:** Target protein MBP, probe 1°Ab rabbit IgG monoclonal anti-MB labeled with B5 initiator, amplifier B5-Alexa546.
- **Ch3:** Target mRNA *Prkcd*, probe set with 31 split-initiator probe pairs, amplifier B1-Alexa647.
- **Ch4:** Target mRNA *Slc17a7*, probe set with 36 split-initiator pairs, amplifier B2-Alexa750.
- **Ch5:** DAPI.

Additional studies are presented as follows:

- Figure S35 displays 4-plex images for $N = 3$ replicate FFPE mouse brain sections (cf. Figures 5CD).
- Figure S36 displays representative regions of individual channels used for measurement of signal and background for each target.
- Table S26 displays estimated values for signal, background, noise, and signal-to-background for each target.

Protocol: Simultaneous HCR 1°IHC + HCR RNA-ISH (Section S3.2; with the optional autofluorescence bleaching protocol of Section S3.2.3) using initiator-labeled primary antibody probes for protein targets, split-initiator DNA probes for RNA targets, and simultaneous HCR signal amplification for all targets.
Sample: FFPE C57BL/6 mouse brain section (coronal); thickness: 5 μm.
Microscopy: Epifluorescence.

Development • Supplementary information

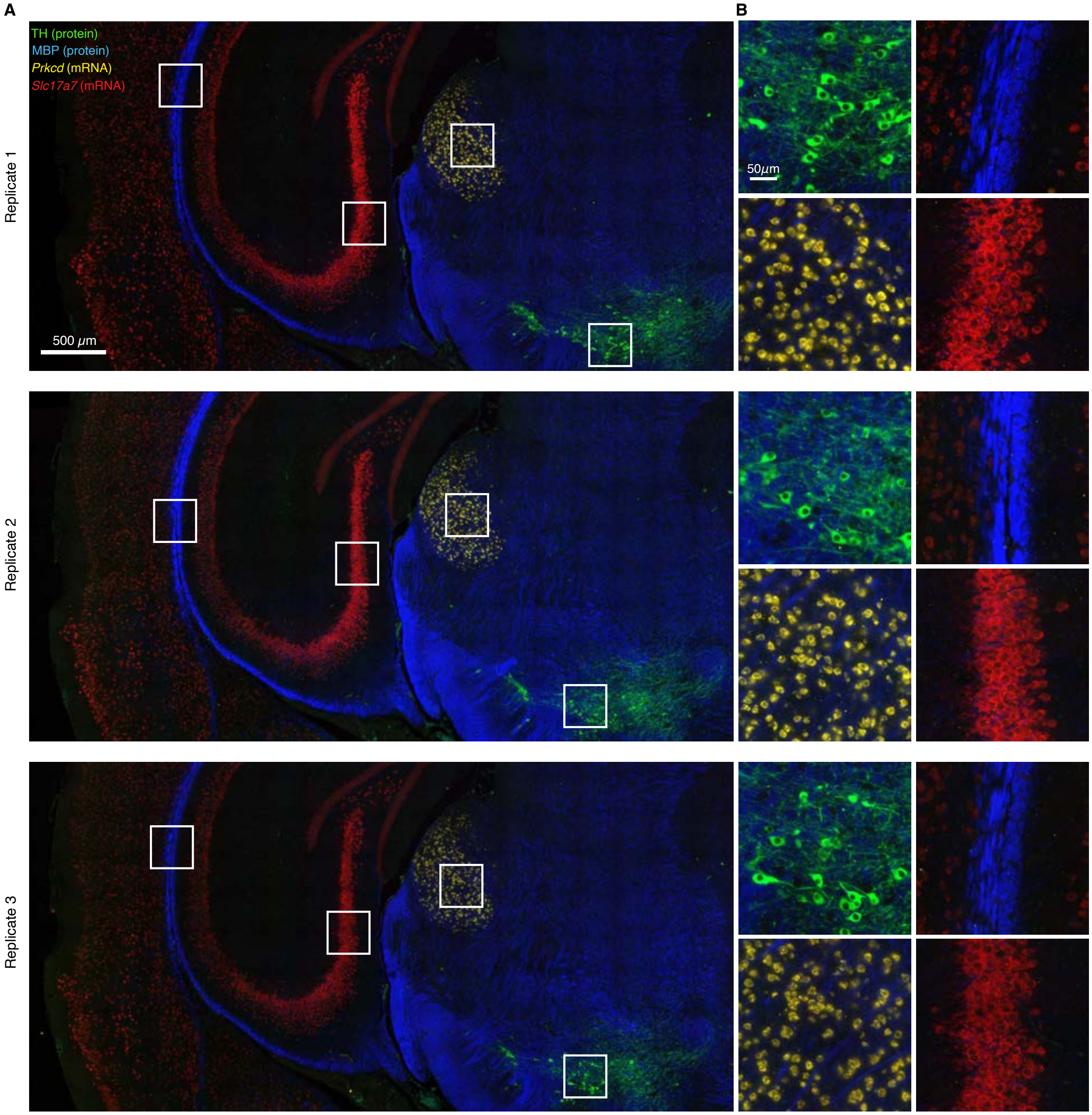

Figure S35. Replicates for 4-plex simultaneous protein and mRNA imaging using HCR 1°IHC and HCR RNA-ISH in FFPE mouse brain sections (cf. Figures 5CD). (A) 4-channel epifluorescence images for 3 replicate FFPE mouse brain sections. (B) Zoom of the depicted regions. Ch1: target protein TH (Alexa488). Ch2: target protein MBP (Alexa546). Ch3: target mRNA *Prkcd* (Alexa647). Ch4: target mRNA *Slc17a7* (Alexa750). Sample: FFPE C57BL/6 mouse brain section (coronal); thickness: 5 μm.

Development • Supplementary information

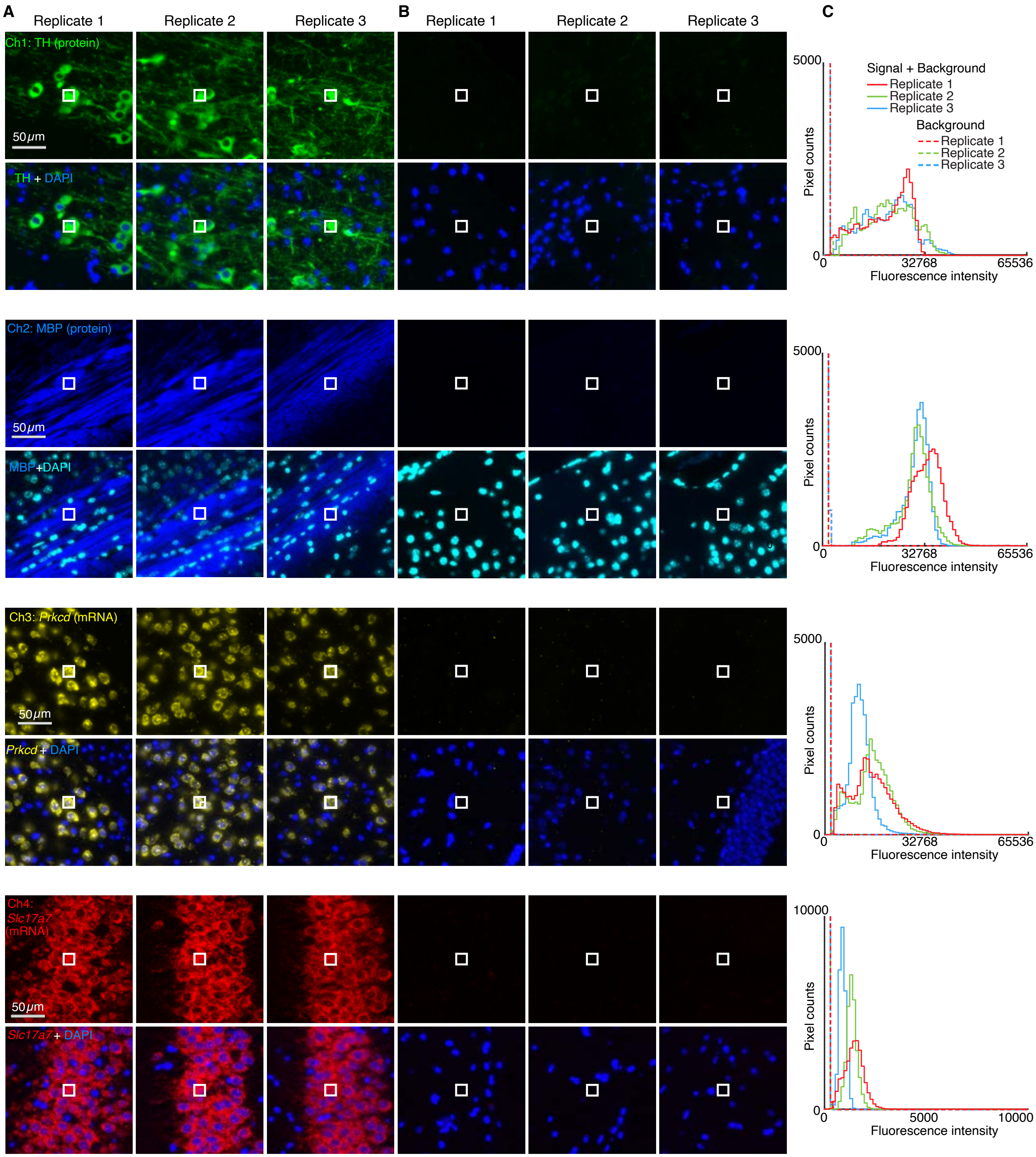

Figure S36. Measurement of signal, background, and noise for 4-plex simultaneous protein and mRNA imaging using HCR 1°IHC and HCR RNA-ISH in FFPE mouse brain sections (cf. Figure 5CD). Use experiment of Type 1 in Table S7A (1°Ab probe + hairpins) to measure: (A) SIG+BACK+NOISE (region of high expression), (B) BACK+NOISE (region of no/low expression) and NOISE (region with no sample; not depicted). (C) Pixel intensity histograms for representative regions (three rectangles per experiment type for each of 3 replicate mouse brain sections). Epifluorescence images collected with the microscope exposure time optimized to avoid saturating SIG+BACK pixels; DAPI channel facilitates placement of representative rectangles. Ch1: target protein TH (Alexa488). Ch2: target protein MBP (Alexa546). Ch3: target mRNA *Prkcd* (Alexa647). Ch4: target mRNA *Slc17a7* (Alexa750). Ch5: DAPI. Sample: FFPE C57BL/6 mouse brain section (coronal); thickness: 5 μm.

Development • Supplementary information

Channel	Target	Type	Fluorophore	NOISE	BACK+NOISE	SIG+BACK+NOISE	BACK	SIG	SIG/BACK
Ch1	TH	protein	Alexa488	450 ± 40	920 ± 60	$19\,600 \pm 700$	470 ± 70	$18\,700 \pm 700$	40 ± 6
Ch2	MBP	protein	Alexa546	520 ± 120	1310 ± 120	$30\,000 \pm 3000$	790 ± 170	$29\,000 \pm 3000$	37 ± 9
Ch3	*Prkcd*	mRNA	Alexa647	490 ± 20	570 ± 40	$13\,000 \pm 2000$	80 ± 50	$13\,000 \pm 2000$	160 ± 100
Ch4	*Slc17a7*	mRNA	Alexa750	180 ± 20	185 ± 19	1868 ± 19	10 ± 3	1680 ± 30	170 ± 50

Table S26. Estimated signal-to-background for 4-plex simultaneous protein and mRNA imaging using HCR 1°IHC and HCR RNA-ISH in FFPE mouse brain sections (cf. Figure 5CD). Mean ± standard error of the mean, $N = 3$ replicate FFPE mouse brain sections. Analysis based on representative rectangular regions (examples depicted in Figure S36) using methods of Section S2.6.2.

Development • Supplementary information

S5.8 Testing whether protein imaging using HCR 1°IHC is affected by RNA imaging using HCR RNA-ISH and vice versa (cf. Figure 5)

Here, we test whether protein imaging using HCR 1°IHC is affected by RNA imaging using HCR RNA-ISH and vice versa. Results are summarized in Table S27. We observe high signal-to-background in all cases. The PCNA target protein illustrates that combining HCR 1°IHC with HCR RNA-FISH can sometimes reduce signal for a target protein, presumably in cases where the target:probe complex is only marginally stable, allowing subsequent RNA-ISH washes to remove a fraction of the antibody probes from the sample.

	Method	Sample	Target	Type	Fluorophore	SIG	BACK	SIG/BACK	Table
**A**	HCR 1°ICC	mammalian cells on a slide	HSP60	protein	Alexa546	31 100 ± 1300	69.5 ± 1.3	450 ± 20	S28
	HCR 1°ICC + HCR RNA-ISH	mammalian cells on a slide	HSP60	protein	Alexa546	26 200 ± 900	55.6 ± 0.8	471 ± 18	S28
	HCR 1°ICC	mammalian cells on a slide	PCNA	protein	Alexa647	45 800 ± 600	70.0 ± 1.1	655 ± 14	S28
	HCR 1°ICC + HCR RNA-ISH	mammalian cells on a slide	PCNA	protein	Alexa647	12 100 ± 600	65.5 ± 0.8	185 ± 9	S28
	HCR 1°IHC	FFPE mouse brain section	TH	protein	Alexa488	35 000 ± 4000	1700 ± 200	21 ± 3	S29
	HCR 1°IHC + HCR RNA-ISH	FFPE mouse brain section	TH	protein	Alexa488	23 900 ± 900	1470 ± 50	16 ± 1	S29
	HCR 1°IHC	FFPE mouse brain section	MBP	protein	Alexa546	20 200 ± 600	900 ± 300	22 ± 2	S29
	HCR 1°IHC + HCR RNA-ISH	FFPE mouse brain section	MBP	protein	Alexa546	22 000 ± 2000	670 ± 70	33 ± 5	S29
**B**	HCR RNA-ISH	mammalian cells on a slide	*U6*	RNA	Alexa647	40 100 ± 1000	120 ± 10	340 ± 30	S28
	HCR 1°ICC + HCR RNA-ISH	mammalian cells on a slide	*U6*	RNA	Alexa647	40 900 ± 1000	100 ± 6	400 ± 30	S28
	HCR RNA-ISH	mammalian cells on a slide	*ACTB*	mRNA	Alexa546	28 800 ± 1000	940 ± 180	31 ± 6	S28
	HCR 1°ICC + HCR RNA-ISH	mammalian cells on a slide	*ACTB*	mRNA	Alexa546	19 200 ± 1000	1600 ± 60	12.0 ± 0.8	S28
	HCR RNA-ISH	FFPE mouse brain section	*Prkcd*	mRNA	Alexa647	13 900 ± 1400	1200 ± 600	11 ± 5	S29
	HCR 1°IHC + HCR RNA-ISH	FFPE mouse brain section	*Prkcd*	mRNA	Alexa647	13 200 ± 1100	960 ± 170	14 ± 3	S29
	HCR RNA-ISH	FFPE mouse brain section	*Slc17a7*	mRNA	Alexa750	1230 ± 130	45 ± 11	27 ± 7	S29
	HCR 1°IHC + HCR RNA-ISH	FFPE mouse brain section	*Slc17a7*	mRNA	Alexa750	1710 ± 120	42 ± 14	41 ± 14	S29

Table S27. Summary of signal, background, and signal-to-background for protein imaging using HCR 1°IHC, RNA imaging using HCR RNA-ISH, or both (cf. Figure 5). (A) Protein imaging using 1°IHC HCR with and without HCR RNA-ISH. (B) RNA imaging using HCR RNA-ISH with and without 1°IHC HCR. Mean ± standard error of the mean. For mammalian cells on a slide, estimates are based on $N = 15$ representative rectangular regions (one rectangle in each of 5 individual cells in each of 3 replicate wells on a multi-well slide; examples depicted in Figure S37 and S38). For FFPE mouse brain sections, estimates are based on representative rectangular regions of $N = 3$ replicate sections (examples depicted in Figure S39 and S40). See Tables S28 and S29 for details.

Development • Supplementary information

S5.8.1 Mammalian cells on a slide

In mammalian cells, we image 2 target proteins:

- Target protein HSP60, probe 1°mAb rabbit IgG anti-HSP60 labeled with B3 initiator, amplifier B3-Alexa546.
- Target protein PCNA, probe 1°mAb mouse IgG2a anti-PCNA labeled with B5 initiator, amplifier B5-Alexa647.

and 2 target RNAs:

- Target RNA *U6*, probe set with 2 split-initiator probe pairs, amplifier B1-Alexa647.
- Target mRNA *ACTB*, probe set with 10 split-initiator pairs, amplifier B2-Alexa546.

Additional studies are presented as follows:

- Figure S37 compares protein imaging using HCR 1°ICC with and without HCR RNA-ISH for 2 target proteins.
- Figure S38 compares RNA imaging using RNA-ISH with and without HCR 1°ICC for 2 target RNAs.
- Table S28 displays estimated values for signal, background, and signal-to-background for each target.

Protocol: HCR 1°ICC only, or simultaneous HCR 1°ICC + HCR RNA-ISH, or HCR RNA-ISH only (Section S3.1) using initiator-labeled primary antibody probes for protein targets, split-initiator DNA probes for RNA targets, and simultaneous HCR signal amplification for all targets.
Sample: HeLa cells.
Microscopy: Confocal.

Development • Supplementary information

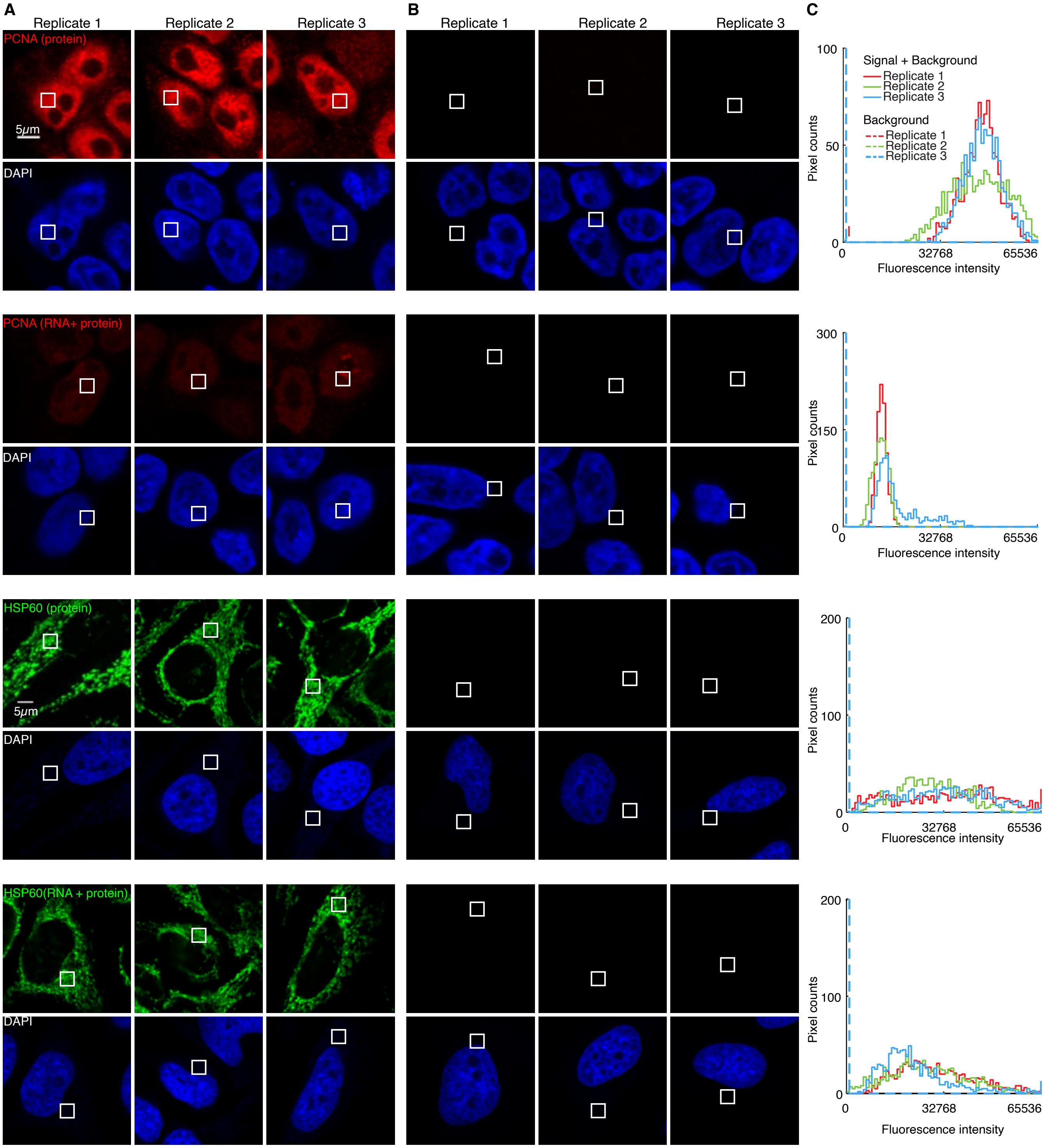

Figure S37. Measurement of signal and background for target proteins using HCR 1°ICC with or without HCR RNA-ISH in mammalian cells on a slide. (A) Use experiment of Type 1 in Table S7A (1°Ab probe + hairpins) to measure SIG+BACK in a region of high expression. (B) Use experiment of Type 2 in Table S7B (hairpins only) to measure NSA+AF in a region of maximum background. (C) Pixel intensity histograms for representative regions (one rectangle in each of 5 individual cells in each of 3 replicate wells on a multi-well slide). For each of 2 target proteins (PCNA or HSP60), data is presented using HCR 1°ICC only or HCR 1°ICC + HCR RNA-ISH. Confocal images collected with the microscope gain optimized to avoid saturating SIG+BACK pixels for HCR 1°ICC; DAPI channel facilitates placement of representative rectangles; single optical section. Target proteins: PCNA (Alexa647) and HSP60 (Alexa546). Sample: HeLa cells.

Development • Supplementary information

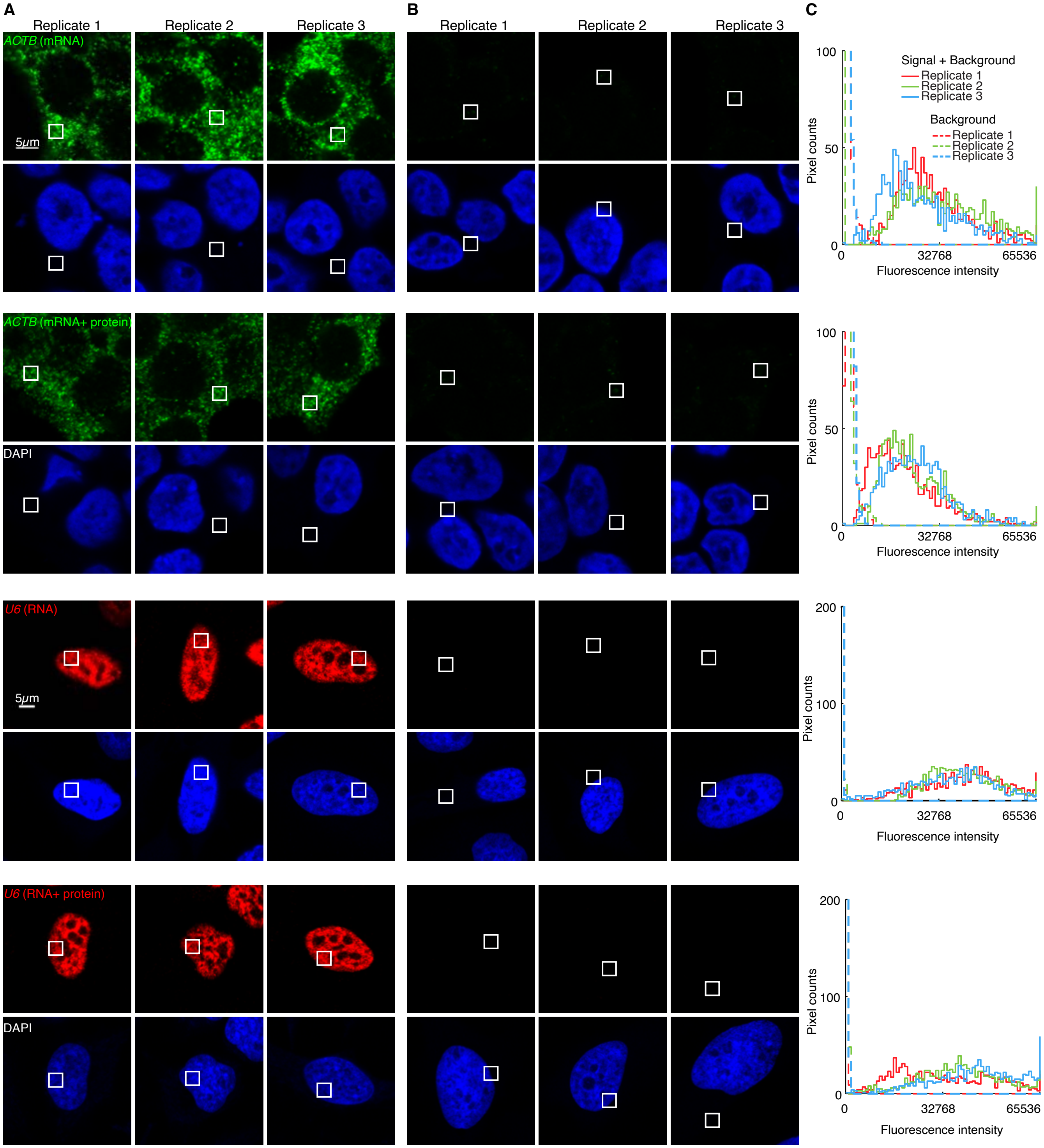

Figure S38. Measurement of signal and background for target RNAs using HCR RNA-ISH with or without HCR 1°ICC in mammalian cells on a slide. (A) Use experiment of Type 1 in Table S7A (probe set + hairpins) to measure SIG+BACK in a region of high expression. (B) Use experiment of Type 2 in Table S7B (hairpins only) to measure NSA+AF in a region of maximum background. (C) Pixel intensity histograms for representative regions (one rectangle in each of 5 individual cells in each of 3 replicate wells on a multi-well slide). For each of 2 target RNAs (*ACTB* or *U6*), data is presented using HCR RNA-ISH only or HCR 1°ICC + HCR RNA-ISH. Confocal images collected with the microscope gain optimized to avoid saturating SIG+BACK pixels for HCR RNA-ISH; DAPI channel facilitates placement of representative rectangles; single optical section. Target RNAs: *ACTB* (Alexa546) and *U6* (Alexa647). Sample: HeLa cells.

Development • Supplementary information

Target	Type	Method	Fluorophore	SIG+BACK	SIG	BACK	SIG/BACK	Experiment
HSP60	protein	HCR 1°ICC	Alexa546	31 100 ± 1300	31 100 ± 1300	69.5 ± 1.3	450 ± 20	1
HSP60	protein	HCR 1°ICC + HCR RNA-ISH	Alexa546	26 300 ± 900	26 200 ± 900	55.6 ± 0.8	471 ± 18	3
PCNA	protein	HCR 1°ICC	Alexa647	45 900 ± 600	45 800 ± 600	70.0 ± 1.1	655 ± 14	4
PCNA	protein	HCR 1°ICC + HCR RNA-ISH	Alexa647	12 200 ± 600	12 100 ± 600	65.5 ± 0.8	185 ± 9	6
U6	RNA	HCR RNA-ISH	Alexa647	40 200 ± 1000	40 100 ± 1000	120 ± 10	340 ± 30	2
U6	RNA	HCR 1°ICC + HCR RNA-ISH	Alexa647	41 000 ± 1000	40 900 ± 1000	100 ± 6	400 ± 30	3
ACTB	mRNA	HCR RNA-ISH	Alexa546	29 800 ± 1000	28 800 ± 1000	940 ± 180	31 ± 6	5
ACTB	mRNA	HCR 1°ICC + HCR RNA-ISH	Alexa546	20 800 ± 1000	19 200 ± 1000	1600 ± 60	12.0 ± 0.8	6

Table S28. Estimated signal, background, and signal-to-background for protein imaging using HCR 1°ICC, RNA imaging using HCR RNA-ISH, or both in mammalian cells on a slide (cf. Figure 5B). The signal estimate SIG is calculated using the background approximation BACK ≈ NSA+AF. Instrument noise is negligible using confocal microscopy so calculations use the approximation NOISE ≈ 0. Mean ± standard error of the mean, $N = 15$ representative rectangular regions (one rectangle in each of 5 individual cells on each of 3 replicate wells on a multi-well slide). Analysis based on representative rectangular regions (examples depicted in Figures S37–S38) using methods of Section S2.6.2. Experiment number designates which target proteins and RNAs were imaged together.

Development • Supplementary information

S5.8.2 FFPE mouse brain sections

In FFPE mouse brain sections, we image 2 target proteins:

- Target protein TH, probe 1°mAb rabbit IgG anti-TH labeled with B1 initiator, amplifier B1-Alexa488.
- Target protein MBP, probe 1°mAb rabbit IgG anti-MB labeled with B5 initiator, amplifier B5-Alexa546.

and 2 target RNAs:

- Target mRNA *Prkcd*, probe set with 31 split-initiator probe pairs, amplifier B2-Alexa647.
- Target mRNA *Slc17a7*, probe set with 36 split-initiator pairs, amplifier B4-Alexa750.

Additional studies are presented as follows:

- Figure S39 compares protein imaging using HCR 1°IHC with and without HCR RNA-ISH for 2 target proteins.
- Figure S40 compares RNA imaging using HCR RNA-ISH with and without HCR 1°IHC for 2 target RNAs.
- Table S29 displays estimated values for signal, background, and signal-to-background for each target.

Protocol: HCR 1°IHC only, or simultaneous HCR 1°IHC + HCR RNA-ISH, or HCR RNA-ISH only (Section S3.2; without the optional autofluorescence bleaching protocol of Section S3.2.3) using initiator-labeled primary antibody probes for protein targets, split-initiator DNA probes for RNA targets, and simultaneous HCR signal amplification for all targets.
Sample: FFPE C57BL/6 mouse brain section (coronal); thickness: 5 μm.
Microscopy: Epifluorescence.

Development • Supplementary information

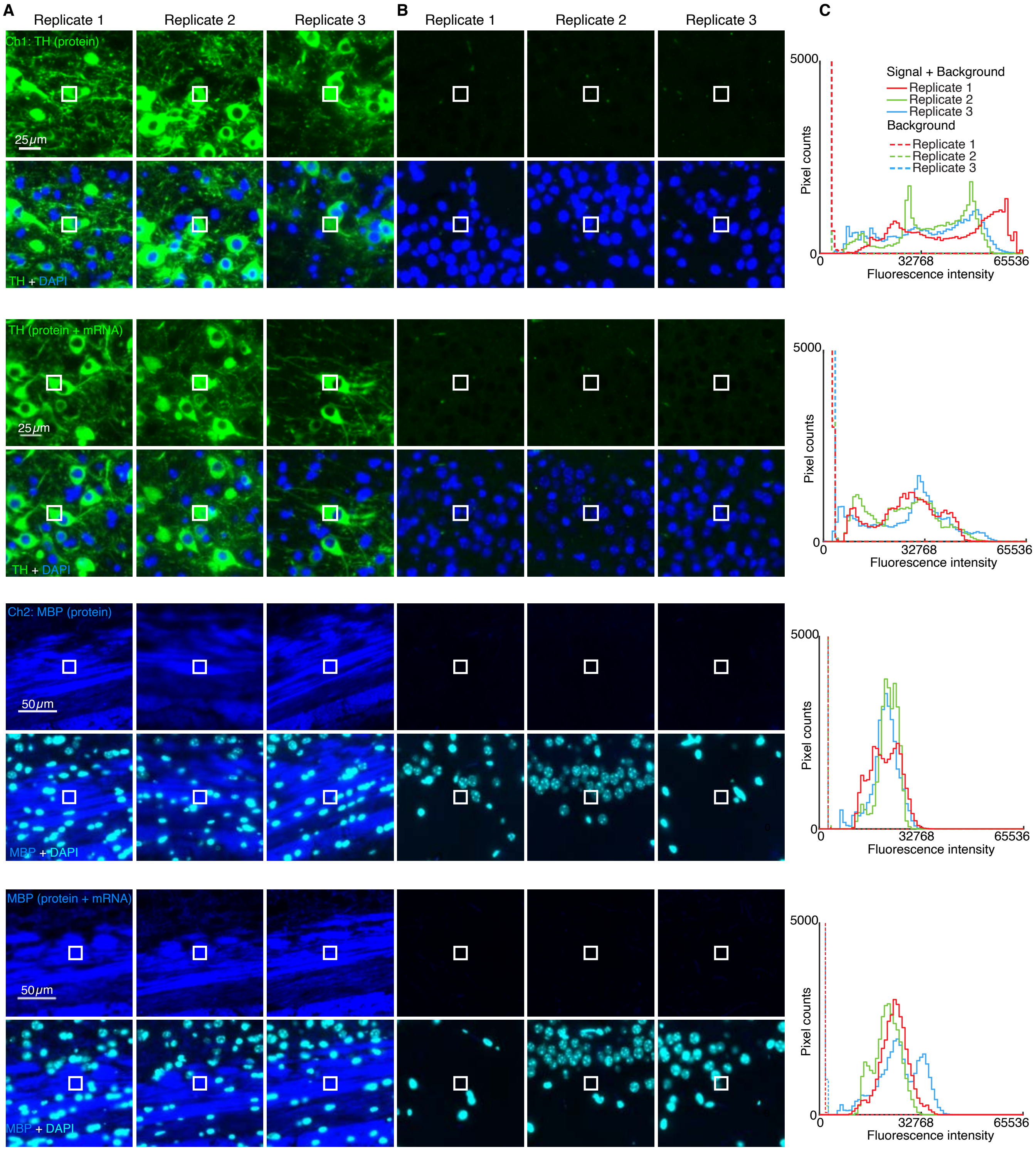

Figure S39. Measurement of signal and background for target proteins using HCR 1°IHC with or without HCR RNA-ISH in FFPE mouse brain sections. Use experiment of Type 1 in Table S7A (1°Ab probe + hairpins) to measure: (A) SIG+BACK+NOISE in a region of high expression, (B) BACK+NOISE in a region of no/low expression, and NOISE in a region with no sample (not depicted). (C) Pixel intensity histograms for representative regions (three rectangles per experiment type for each of 3 replicate mouse brain sections). For each of 2 target proteins (TH or MBP), data is presented using HCR 1°IHC only or HCR 1°IHC + HCR RNA-ISH. Epifluorescence images collected with the microscope exposure time optimized to avoid saturating SIG+BACK pixels for HCR 1°IHC; DAPI channel facilitates placement of representative rectangles. Target proteins: TH (Alexa488) and MBP (Alexa546). Sample: FFPE C57BL/6 mouse brain section (coronal); thickness: 5 μm.

Development • Supplementary information

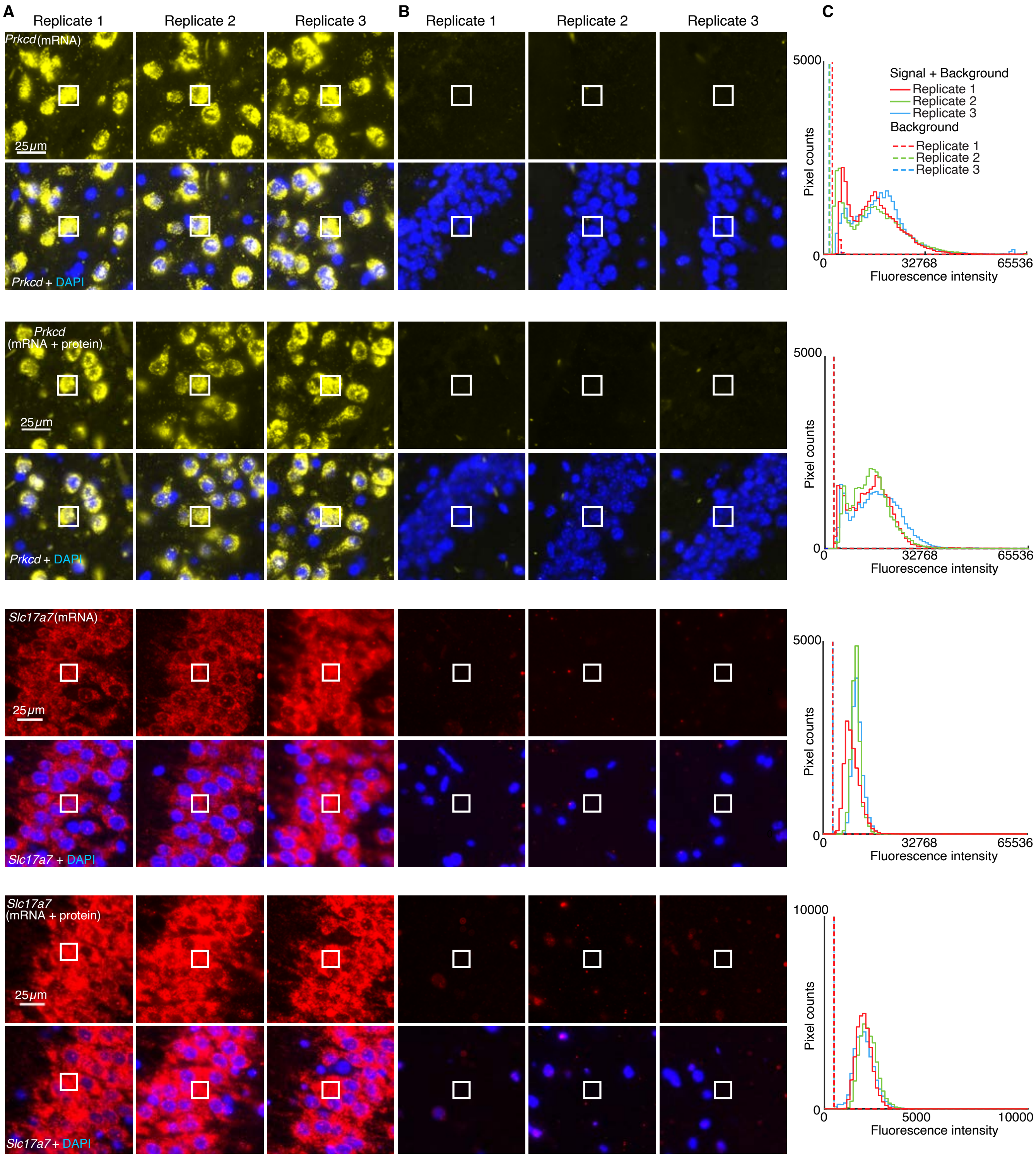

Figure S40. Measurement of signal and background for target RNAs using HCR RNA-ISH with or without HCR 1°IHC in FFPE mouse brain sections. Use experiment of Type 1 in Table S7A (probe set + hairpins) to measure: (A) SIG+BACK+NOISE in a region of high expression, (B) BACK+NOISE in a region of no/low expression, and NOISE in a region with no sample (not depicted). (C) Pixel intensity histograms for representative regions (three rectangles per experiment type for each of 3 replicate mouse brain sections). For each of 2 target RNAs (*Prkcd* or *Slc17a7*), data is presented using HCR RNA-ISH only or HCR 1°IHC + HCR RNA-ISH. Epifluorescence images collected with the microscope exposure time optimized to avoid saturating SIG+BACK pixels for HCR RNA-ISH; DAPI channel facilitates placement of representative rectangles. Target mRNAs: *Prkcd* (Alexa647) and *Slc17a7* (Alexa750). Sample: FFPE C57BL/6 mouse brain section (coronal); thickness: 5 μm.

Development • Supplementary information

Target	Type	Method	Fluorophore	NOISE	BACK+NOISE	SIG+BACK+NOISE	SIG	BACK	SIG/BACK	Experiment
TH	protein	HCR 1°IHC	Alexa488	530 ± 160	2240 ± 140	37 000 ± 4000	35 000 ± 4000	1700 ± 200	21 ± 3	1
TH	protein	HCR 1°IHC + HCR RNA-ISH	Alexa488	425 ± 7	1900 ± 50	25 800 ± 900	24 000 ± 900	1470 ± 50	16 ± 1	3
MBP	protein	HCR 1°IHC	Alexa546	700 ± 200	1610 ± 160	20 900 ± 600	20 200 ± 600	900 ± 300	22 ± 2	1
MBP	protein	HCR 1°IHC + HCR RNA-ISH	Alexa546	500 ± 60	1160 ± 40	23 000 ± 2000	22 000 ± 2000	670 ± 70	33 ± 5	3
Prkcd	mRNA	HCR RNA-ISH	Alexa647	1350 ± 140	2600 ± 500	16 500 ± 1300	13 900 ± 1400	1200 ± 600	11 ± 5	2
Prkcd	mRNA	HCR 1°IHC + HCR RNA-ISH	Alexa647	800 ± 100	1720 ± 140	14 900 ± 1100	13 200 ± 1100	960 ± 170	14 ± 3	3
Slc17a7	mRNA	HCR RNA-ISH	Alexa750	225 ± 8	270 ± 7	1500 ± 130	1230 ± 130	45 ± 11	27 ± 7	2
Slc17a7	mRNA	HCR 1°IHC + HCR RNA-ISH	Alexa750	200 ± 10	240 ± 10	1950 ± 120	1700 ± 120	42 ± 14	41 ± 14	3

Table S29. Estimated signal, background, and signal-to-background for protein imaging using HCR 1°IHC, RNA imaging using HCR RNA-ISH, or both in FFPE mouse brain sections (cf. Figure 5CD). Mean ± standard error of the mean, $N = 3$ replicate FFPE mouse brain sections. Analysis based on representative rectangular regions (examples depicted in Figures S39–S40) using methods of Section S2.6.2. Experiment number designates which target proteins and RNAs were imaged together.

S5.9 Replicates and signal-to-background measurements for simultaneous multiplexed protein and mRNA imaging using HCR 2°IHC and HCR RNA-ISH (cf. Figure 6)

S5.9.1 Mammalian cells on a slide

For 4-plex simultaneous protein and mRNA imaging using HCR 2°ICC + HCR RNA-ISH in mamalian cells on a slide, the 5 channels are (2 RNAs + 2 proteins + DAPI):

- **Ch1:** Target protein PCNA, probe 1°mAb mouse IgG2a anti-PCNA , probe 2°pAb goat anti-mouse IgG2a labeled with B5 initiator, amplifier B5-Alexa488.
- **Ch2:** Target protein HSP60, probe 1°mAb rabbit anti-Hsp60, probe 2°pAb donkey anti-rabbit labeled with B4 initiator, amplifier B4-Alexa546.
- **Ch3:** Target RNA *U6*, probe set with 2 split-initiator probe pairs, amplifier B1-Alexa594.
- **Ch4:** Target mRNA *HSP60*, probe set with 18 split-initiator probe pairs, amplifier B2-Alexa647.
- **Ch5:** DAPI.

Additional studies are presented as follows:

- Figure S41 displays 4-plex images for $N = 3$ replicate wells on a multi-well slide (cf. Figure 6B).
- Figure S42 displays representative regions of individual channels used for measurement of signal and background for each target.
- Table S30 displays estimated values for signal, background, and signal-to-background for each target.

Protocol: Simultaneous HCR 2°ICC + HCR RNA-ISH (Section S4.1) using unlabeled primary antibody probes and initiator-labeled secondary antibody probes for protein targets, split-initiator DNA probes for RNA targets, and simultaneous HCR signal amplification for all targets.
Sample: HeLa cells.
Microscopy: Confocal.

Development • Supplementary information

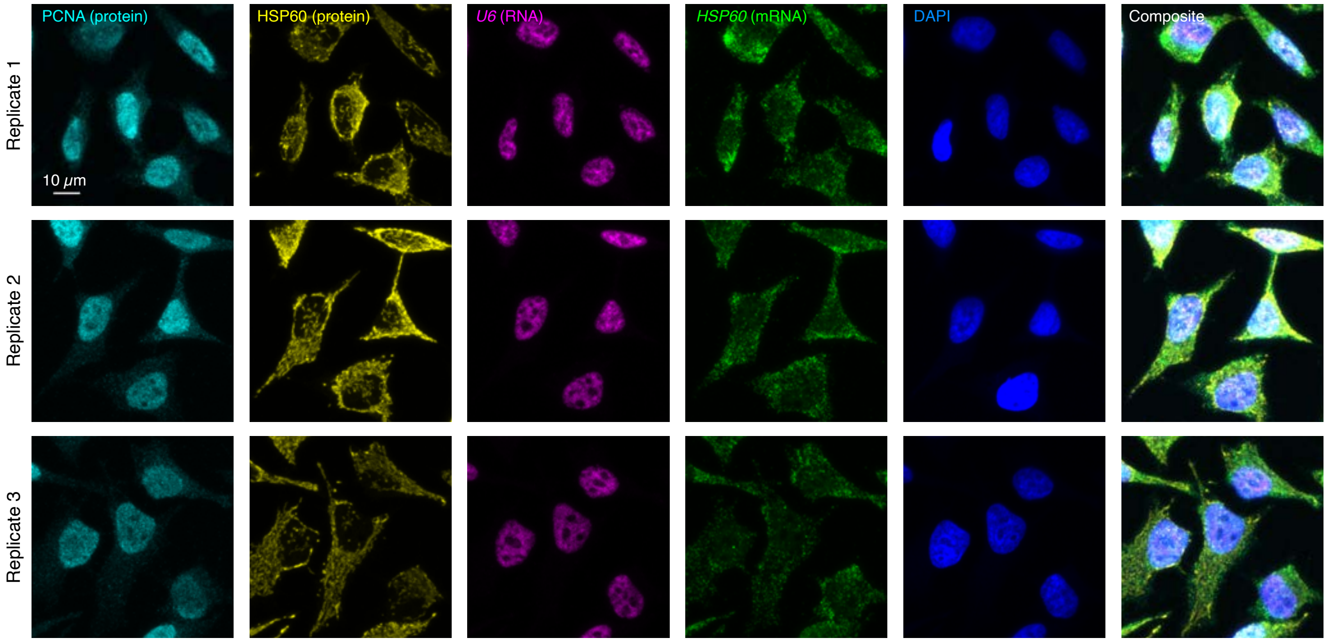

Figure S41. Replicates for 4-plex simultaneous protein and mRNA imaging using HCR 2°ICC and HCR RNA-ISH in mammalian cells on slides (cf. Figures 6B). 5-channel confocal images for 3 replicate wells in a multi-well slide; maximum intensity z-projection. Ch1: target protein PCNA (Alexa488). Ch2: target protein Hsp60 (Alexa546). Ch3: target RNA *U6* (Alexa594). Ch4: target mRNA *Hsp60* (Alexa647). Ch5: DAPI. Sample: HeLa cells.

Channel	Target	Type	Fluorophore	BACK	SIG+BACK	SIG	SIG/BACK
Ch1	PCNA	protein	Alexa488	453 ± 12	$13\,700 \pm 1000$	$12\,200 \pm 1000$	27 ± 2
Ch2	Hsp60	protein	Alexa546	158 ± 5	$38\,000 \pm 3000$	$38\,000 \pm 3000$	240 ± 20
Ch3	*U6*	RNA	Alexa594	135 ± 3	$38\,000 \pm 2000$	$38\,000 \pm 2000$	279 ± 17
Ch4	*Hsp60*	mRNA	Alexa647	202 ± 9	9300 ± 1100	9100 ± 1100	45 ± 6

Table S30. Estimated signal-to-background for 4-plex simultaneous protein and mRNA imaging using HCR 2°ICC and HCR RNA-ISH in mammalian cells on a slide (cf. Figure 6B). For protein targets, the signal estimate SIG is calculated using the background approximation BACK $\approx$ $NSD_{2°}$+NSA+AF. For RNA targets, the signal estimate SIG is calculated using the background approximation BACK $\approx$ NSA+AF. Instrument noise is negligible using confocal microscopy so calculations use the approximation NOISE ≈ 0. Mean $\pm$ standard error of the mean, $N = 15$ representative rectangular regions (one rectangle in each of 5 individual cells in each of 3 replicate wells on a multi-well slide). Analysis based on representative rectangular regions (examples depicted in Figure S42) using methods of Section S2.6.2.

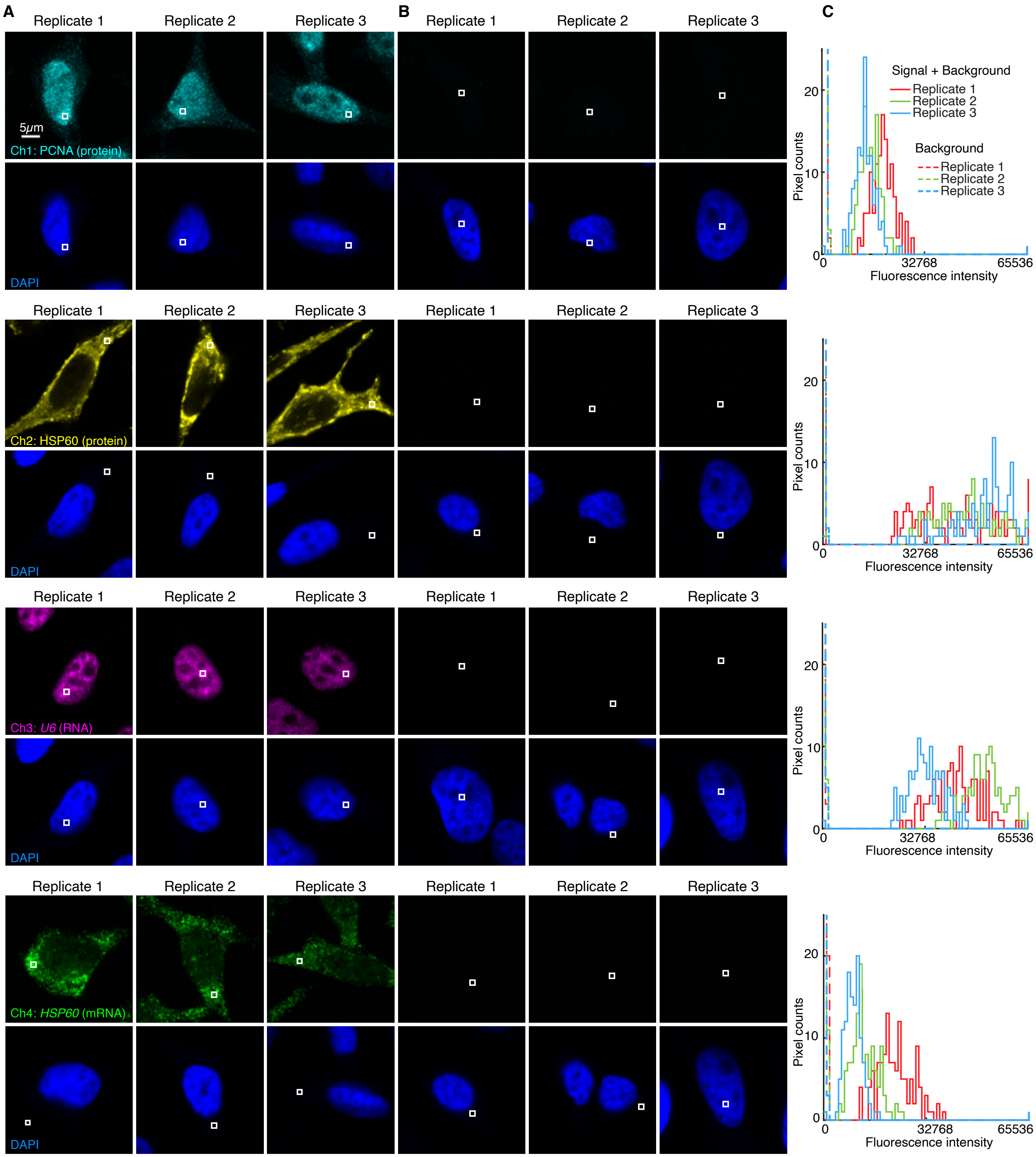

Figure S42. Measurement of signal and background for 4-plex simultaneous protein and mRNA imaging using HCR 2°ICC and HCR RNA-ISH in mammalian cells on a slide (cf. Figure 6B). (A) Use experiment of Type 1 in Table S8A (1°Ab probe + 2°Ab probe + hairpins) to measure SIG+BACK in a region of high expression. (B) For RNA targets, use experiment of Type 2 in Table S8B (2°Ab probe + hairpins) to measure NSA+AF in a region of maximum background. For protein targets, use experiment of Type 4 in Table S8B (2°Ab probe + hairpins) to measure $NSD_{2°}$+NSA+AF in a region of maximum background. (C) Pixel intensity histograms for representative regions (one rectangle in each of 5 individual cells in each of 3 replicate wells on a multi-well slide). Confocal images collected with the microscope gain optimized to avoid saturating SIG+BACK pixels; DAPI channel facilitates placement of representative rectangles; single optical section. Ch1: target protein PCNA (Alexa488). Ch2: target protein HSP60 (Alexa546). Ch3: target RNA *U6* (Alexa594). Ch4: target mRNA *Hsp60* (Alexa647). Ch5: DAPI. Sample: HeLa cells.

S5.9.2 FFPE mouse brain sections

For 4-plex simultaneous protein and mRNA imaging using HCR 2°IHC + HCR RNA-ISH in FFPE mouse brain sections, the 5 channels are (2 proteins + 2 RNAs + DAPI):

- **Ch1:** Target protein TH, probe 1°pAb probe sheep IgG anti-TH, probe 2°pAb donkey anti-sheep labeled with B4 initiator, amplifier B4-Alexa488.
- **Ch2:** Target protein MBP, probe 1°mAb probe rat IgG2A anti-MBP, probe 2°pAb donkey anti-rat IgG labeled with B3 initiator, amplifier B3-Alexa546.
- **Ch3:** Target mRNA *Prkcd*, probe set with 31 split-initiator probe pairs, amplifier B1-Alexa647.
- **Ch4:** Target mRNA *Slc17a7*, probe set with 36 split-initiator probe pairs, amplifier B2-Alexa750.
- **Ch5:** DAPI.

Additional studies are presented as follows:

- Figure S43 displays 4-plex images for $N = 3$ replicate FFPE mouse brain sections (cf. Figures 6CD).
- Figure S44 displays representative regions of individual channels used for measurement of signal and background for each target.
- Table S31 displays estimated values for signal, background, noise, and signal-to-background for each target.

Protocol: Simultaneous HCR 2°IHC + HCR RNA-ISH (Section S4.2; with the optional autofluorescence bleaching protocol of Section S4.2.3) using unlabeled primary antibody probes and initiator-labeled secondary antibody probes for protein targets, split-initiator DNA probes for RNA targets, and simultaneous HCR signal amplification for all targets.
Sample: FFPE C57BL/6 mouse brain section (coronal); thickness: 5 μm.
Microscopy: Epifluorescence.

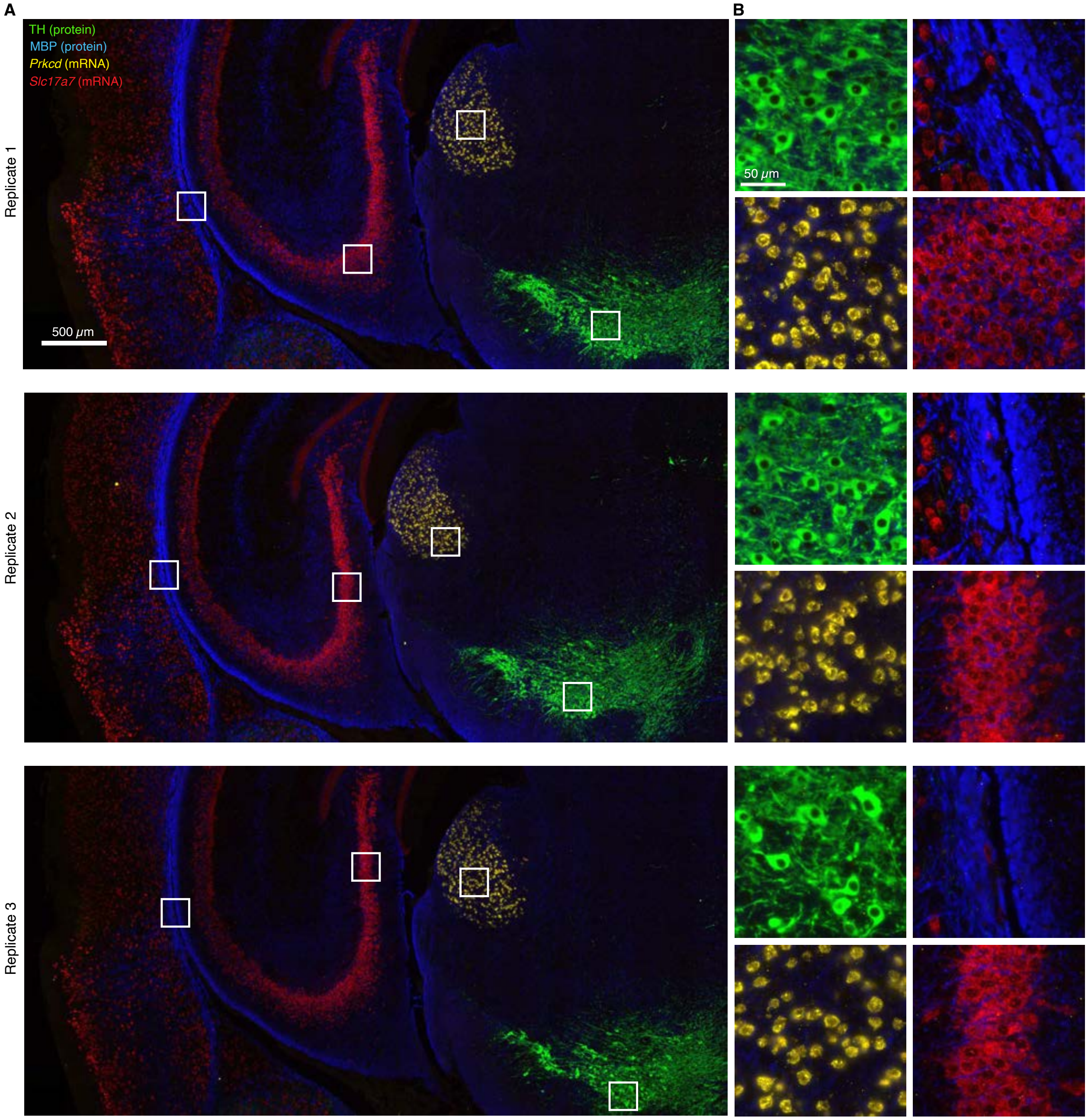

Figure S43. Replicates for 4-plex simultaneous protein and mRNA imaging using HCR 2°IHC and HCR RNA-ISH in FFPE mouse brain sections (cf. Figures 6CD). (A) 4-channel epifluorescence images for 3 replicate FFPE mouse brain sections. (B) Zoom of the depicted regions. Ch1: target protein TH (Alexa488). Ch2: target protein MBP (Alexa546). Ch3: target mRNA *Prkcd* (Alexa647). Ch4: target mRNA *Slc17a7* (Alexa750). Sample: FFPE C57BL/6 mouse brain section (coronal); thickness: 5 μm.

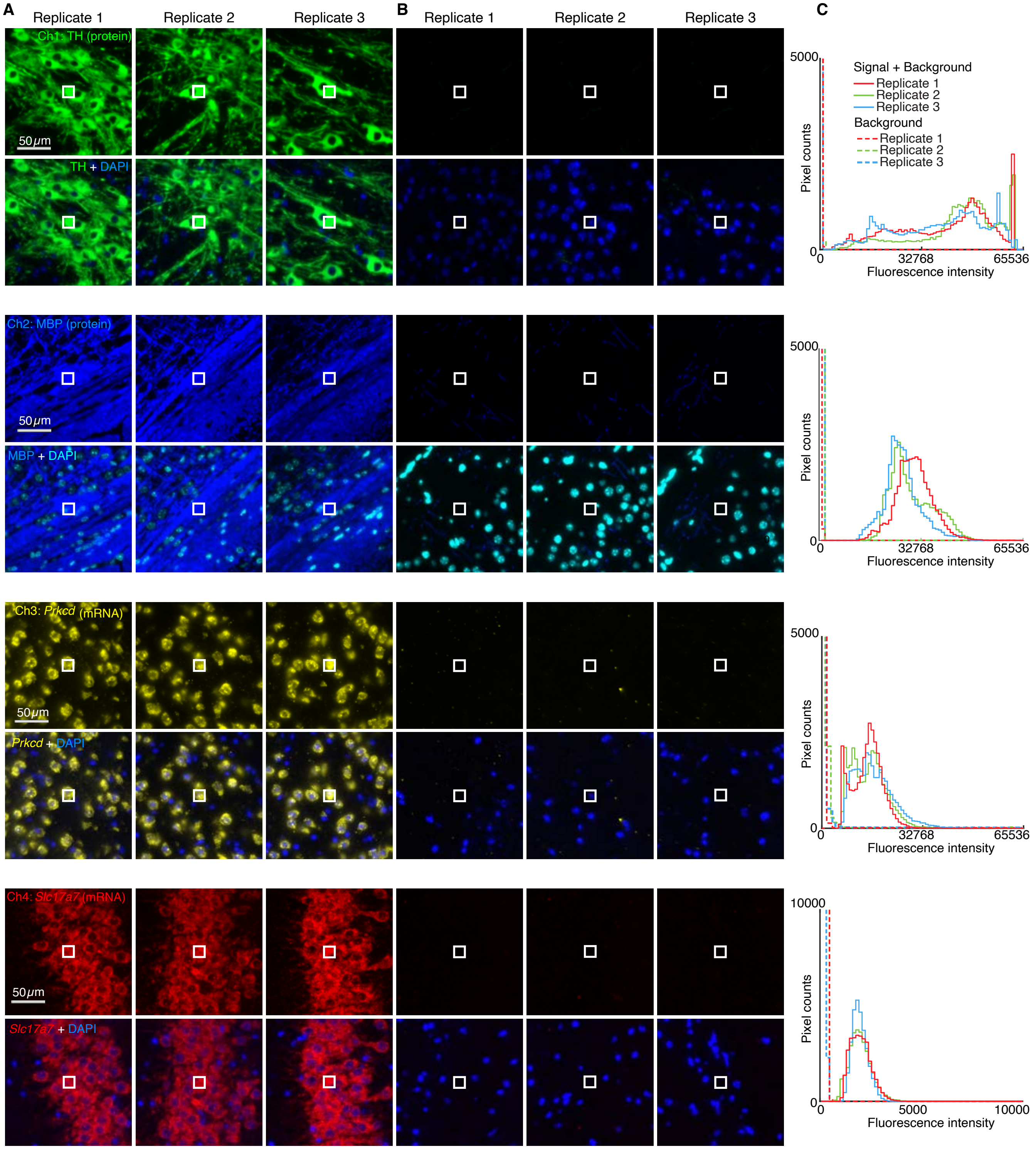

Figure S44. Measurement of signal, background, and noise for 4-plex simultaneous protein and mRNA imaging using HCR 2°IHC and HCR RNA-ISH in FFPE mouse brain sections (cf. Figure 6CD). Use experiment of Type 1 in Table S8A (1°Ab probe + 2°Ab probe + hairpins) to measure (A) SIG+BACK+NOISE in a region of high expression, (B) BACK+NOISE in a region of no/low expression, and NOISE in a region with no sample (not depicted). (C) Pixel intensity histograms for representative regions (three rectangles per experiment type for each of 3 replicate mouse brain sections). Epifluorescence images collected with the microscope exposure time optimized to avoid saturating SIG+BACK pixels; DAPI channel facilitates placement of representative rectangles. Ch1: target protein TH (Alexa488). Ch2: target protein MBP (Alexa546). Ch3: target mRNA *Prkcd* (Alexa647). Ch4: target mRNA *Slc17a7* (Alexa750). Ch5: DAPI. Sample: FFPE C57BL/6 mouse brain section (coronal); thickness: 5 μm.

Development • Supplementary information

Channel	Target	Type	Fluorophore	NOISE	BACK+NOISE	SIG+BACK+NOISE	BACK	SIG	SIG/BACK
Ch1	TH	protein	Alexa488	119 ± 7	180 ± 20	$41\,000 \pm 4000$	60 ± 20	$40\,000 \pm 4000$	700 ± 300
Ch2	MBP	protein	Alexa546	138 ± 3	242 ± 9	$28\,000 \pm 2000$	104 ± 9	$28\,000 \pm 2000$	270 ± 30
Ch3	*Prkcd*	mRNA	Alexa647	376 ± 6	510 ± 30	$11\,600 \pm 1200$	130 ± 30	$11\,100 \pm 1200$	84 ± 20
Ch4	*Slc17a7*	mRNA	Alexa750	149 ± 8	163.5 ± 1.5	1430 ± 130	15 ± 8	1270 ± 130	80 ± 40

Table S31. Estimated signal-to-background for 4-plex simultaneous protein and mRNA imaging using HCR 2°IHC and HCR RNA-ISH in FFPE mouse brain sections (cf. Figure 6CD). Mean ± standard error of the mean, $N = 3$ replicate mouse brain sections. Analysis based on a representative rectangular regions (examples depicted in Figure S44) using methods of Section S2.6.2.

Development • Supplementary information

S5.10 Testing whether protein imaging using HCR 2°IHC is affected by RNA imaging using HCR RNA-ISH and vice versa (cf. Figure 6)

Here, we test whether protein imaging using HCR 2°IHC is affected by RNA imaging using HCR RNA-ISH and vice versa. Results are summarized in Table S32. We observe a high signal-to-background ratio in all cases. The PCNA target protein illustrates that combining HCR 2°IHC with HCR RNA-FISH can sometimes reduce signal for a target protein, presumably in cases where the target:probe complex is only marginally stable, allowing subsequent RNA-ISH washes to remove a fraction of the antibody probes from the sample.

	Method	Sample	Target	Type	Fluorophore	SIG	BACK	SIG/BACK	Table
**A**	HCR 2°ICC	mammalian cells on a slide	HSP60	protein	Alexa546	15 700 ± 1500	142 ± 5	110 ± 11	S33
	HCR 2°ICC + HCR RNA-ISH	mammalian cells on a slide	HSP60	protein	Alexa546	14 400 ± 1200	72.3 ± 1.8	199 ± 18	S33
	HCR 2°ICC	mammalian cells on a slide	PCNA	protein	Alexa647	31 300 ± 1300	410 ± 20	76 ± 5	S33
	HCR 2°ICC + HCR RNA-ISH	mammalian cells on a slide	PCNA	protein	Alexa647	6300 ± 600	210 ± 10	30 ± 3	S33
	HCR 2°IHC	FFPE mouse brain section	TH	protein	Alexa488	10 000 ± 4000	270 ± 30	38 ± 14	S34
	HCR 2°IHC + HCR RNA-ISH	FFPE mouse brain section	TH	protein	Alexa488	15 000 ± 3000	234 ± 5	65 ± 17	S34
	HCR 2°IHC	FFPE mouse brain section	MBP	protein	Alexa546	13 000 ± 4000	500 ± 300	25 ± 18	S34
	HCR 2°IHC + HCR RNA-ISH	FFPE mouse brain section	MBP	protein	Alexa546	11 000 ± 3000	270 ± 80	40 ± 16	S34
**B**	HCR RNA-ISH	mammalian cells on a slide	*U6*	RNA	Alexa647	34 400 ± 1000	270 ± 30	127 ± 16	S33
	HCR 2°ICC + HCR RNA-ISH	mammalian cells on a slide	*U6*	RNA	Alexa647	34 900 ± 1000	182 ± 13	192 ± 15	S33
	HCR RNA-ISH	mammalian cells on a slide	*ACTB*	mRNA	Alexa546	27 000 ± 2000	440 ± 30	60 ± 6	S33
	HCR 2°ICC + HCR RNA-ISH	mammalian cells on a slide	*ACTB*	mRNA	Alexa546	24 000 ± 2000	335 ± 16	73 ± 7	S33
	HCR RNA-ISH	FFPE mouse brain section	*Prkcd*	mRNA	Alexa647	17 200 ± 1800	1000 ± 1000	15 ± 12	S34
	HCR 2°IHC + HCR RNA-ISH	FFPE mouse brain section	*Prkcd*	mRNA	Alexa647	21 000 ± 3000	1000 ± 300	22 ± 8	S34
	HCR RNA-ISH	FFPE mouse brain section	*Slc17a7*	mRNA	Alexa750	1500 ± 180	50 ± 20	33 ± 15	S34
	HCR 2°IHC + HCR RNA-ISH	FFPE mouse brain section	*Slc17a7*	mRNA	Alexa750	1800 ± 300	30 ± 20	50 ± 30	S34

Table S32. Summary of signal, background, and signal-to-background for protein imaging using HCR 2°IHC, RNA imaging using HCR RNA-ISH, or both (cf. Figure 6). (A) Protein imaging using 2°IHC HCR with and without HCR RNA-ISH. (B) RNA imaging using HCR RNA-ISH with and without 2°IHC HCR. Mean ± standard error of the mean. For mammalian cells on a slide, estimates are based on $N = 15$ representative rectangular regions (one rectangle in each of 5 individual cells in each of 3 replicate wells on a multi-well slide; examples depicted in Figure S45 and S46). For FFPE mouse brain sections, estimates are based on representative rectangular regions of $N = 3$ replicate sections (examples depicted in Figure S47 and S48). See Tables S33 and S34 for details.

Development • Supplementary information

S5.10.1 Mammalian cells on a slide

In mammalian cells, we image 2 target proteins:

- Target protein PCNA, probe 1°mAb mouse IgG2a anti-PCNA , probe 2°pAb goat anti-mouse IgG2a labeled with B5 initiator, amplifier B5-Alexa647.
- Target protein Hsp60, probe 1°mAb rabbit anti-Hsp60, probe 2°pAb donkey anti-rabbit labeled with B4 initiator, amplifier B4-Alexa546.

and 2 target RNAs:

- Target RNA *U6*, probe set with 2 split-initiator probe pairs, amplifier B1-Alexa647.
- Target mRNA *ACTB*, probe set with 10 split-initiator probe pairs, amplifier B2-Alexa546.

Additional studies are presented as follows:

- Figure S45 compares protein imaging using HCR 2°ICC with and without HCR RNA-ISH for 2 target proteins.
- Figure S46 compares RNA imaging using RNA-ISH with and without HCR 2°ICC for 2 target RNAs.
- Table S33 displays estimated values for signal, background, and signal-to-background for each target.

Protocol: HCR 2°ICC only, or simultaneous HCR 2°ICC + HCR RNA-ISH, or HCR RNA-ISH only (Section S4.1) using unlabeled primary antibody probes and initiator-labeled secondary antibody probes for protein targets, split-initiator DNA probes for RNA targets, and simultaneous HCR signal amplification for all targets.
Sample: HeLa cells.
Microscopy: Confocal.

Development • Supplementary information

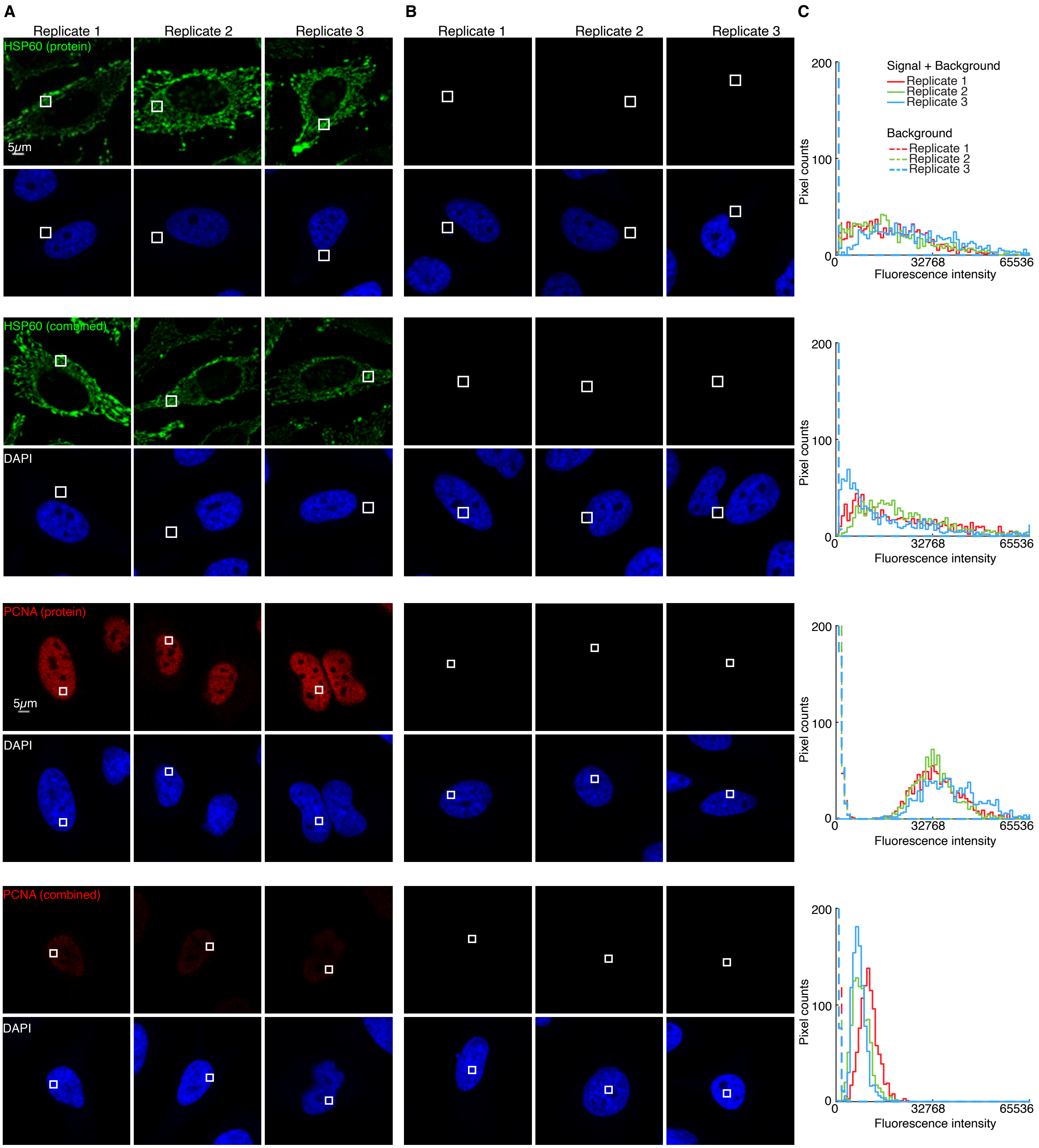

Figure S45. Measurement of signal and background for target proteins using HCR 2°ICC with or without HCR RNA-ISH in mammalian cells on a slide. (A) Use experiment of Type 1 in Table S8A (1°Ab probe + 2°Ab probe + hairpins) to measure SIG+BACK in a region of high expression. (B) Use experiment of Type 4 in Table S8B (2°Ab probe + hairpins) to measure NSD_{2°+NSA+AF in a region of maximum background. (C) Pixel intensity histograms for representative regions (one rectangle in each of 5 individual cells in each of 3 replicate wells on a multi-well slide). For each of 2 target proteins (PCNA or HSP60), data is presented using HCR 2°IHC only or HCR 2°IHC + HCR RNA-ISH. Confocal images collected with the microscope gain optimized to avoid saturating SIG+BACK pixels for HCR 2°IHC; DAPI channel facilitates placement of representative rectangles; single optical section. Target proteins: HSP60 (Alexa546) and PCNA (Alexa647). Sample: HeLa cells.

Development • Supplementary information

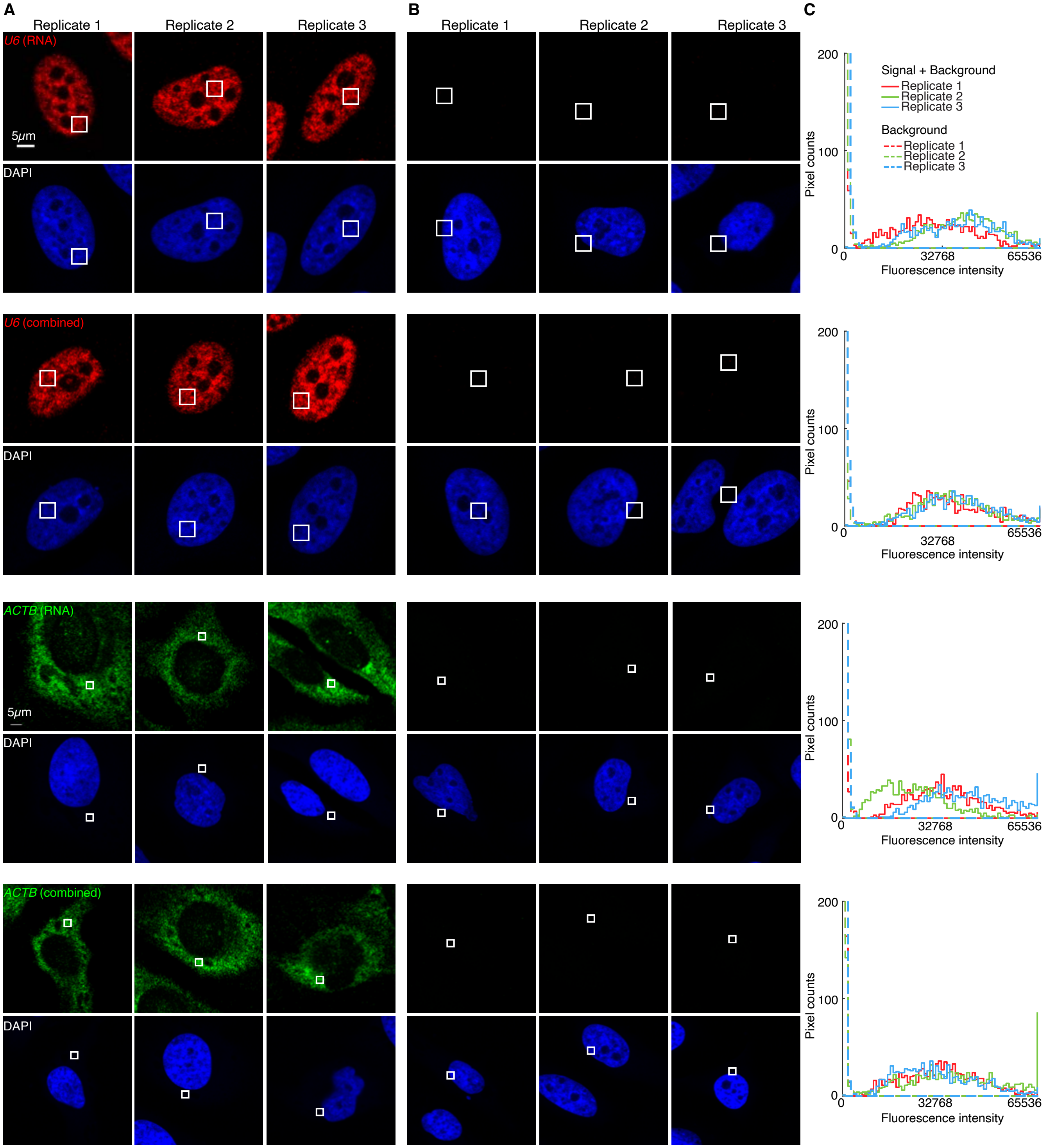

Figure S46. Measurement of signal and background for target RNAs using HCR RNA-ISH with or without HCR 2°ICC in mammalian cells on a slide. (A) Use experiment of Type 1 in Table S8A (probe set + hairpins) to measure SIG+BACK in a region of high expression. (B) Use experiment of Type 2 in Table S8B (hairpins only) to measure NSA+AF in a region of maximum background. (C) Pixel intensity histograms for representative regions (one rectangle in each of 5 individual cells in each of 3 replicate wells on a multi-well slide). For each of 2 target RNAs (*U6* or *ACTB*), data is presented using HCR RNA-ISH only or HCR 2°ICC + HCR RNA-ISH. Confocal images collected with the microscope gain optimized to avoid saturating SIG+BACK pixels for HCR RNA-ISH; DAPI channel facilitates placement of representative rectangles; single optical section. Target RNAs: *U6* (Alexa647) and *ACTB* (Alexa546). Sample: HeLa cells.

Development • Supplementary information

Target	Type	Method	Fluorophore	SIG+BACK	SIG	BACK	SIG/BACK	Experiment
HSP60	protein	HCR 2°ICC	Alexa546	15 900 ± 1500	15 700 ± 1500	142 ± 5	110 ± 11	1
HSP60	protein	HCR 2°ICC + HCR RNA-ISH	Alexa546	14 500 ± 1200	14 400 ± 1200	72.3 ± 1.8	199 ± 18	3
PCNA	protein	HCR 2°ICC	Alexa647	31 800 ± 1300	31 300 ± 1300	410 ± 20	76 ± 5	4
PCNA	protein	HCR 2°ICC + HCR RNA-ISH	Alexa647	6500 ± 600	6300 ± 600	210 ± 10	31 ± 3	6
U6	mRNA	HCR RNA-ISH	Alexa647	34 600 ± 1000	34 400 ± 1000	270 ± 30	127 ± 16	2
U6	mRNA	HCR 2°ICC + HCR RNA-ISH	Alexa647	35 100 ± 1000	34 900 ± 1000	182 ± 13	192 ± 15	3
ACTB	mRNA	HCR RNA-ISH	Alexa546	27 000 ± 2000	27 000 ± 2000	440 ± 30	60 ± 6	5
ACTB	mRNA	HCR 2°ICC + HCR RNA-ISH	Alexa546	25 000 ± 2000	24 000 ± 2000	330 ± 20	73 ± 7	6

Table S33. Estimated signal, background, and signal-to-background for protein imaging using HCR 2°ICC, RNA imaging using HCR RNA-ISH, or both in mammalian cells on a slide (cf. Figure 6B). For protein targets, the signal estimate SIG is calculated using the background approximation BACK ≈ NSD_{2°+NSA+AF. For RNA targets, the signal estimate SIG is calculated using the background approximation BACK ≈ NSA+AF. Instrument noise is negligible using confocal microscopy so calculations use the approximation NOISE ≈ 0. Mean ± standard error of the mean, $N = 15$ representative rectangular regions (one rectangle in each of 5 individual cells on each of 3 replicate wells on a multi-well slide). Analysis based on representative rectangular regions (examples depicted in Figures S45–S46) using methods of Section S2.6.2. Experiment number designates which target proteins and RNAs were imaged together.

Development • Supplementary information

S5.10.2 FFPE mouse brain sections

In FFPE mouse brain sections, we image 2 target proteins:

- Target protein TH, probe 1°pAb probe sheep IgG anti-TH, probe 2°pAb donkey anti-sheep IgG labeled with B4 initiator, amplifier B4-Alexa488.
- Target protein MBP, probe 1°mAb probe rat IgG2A anti-MBP, probe 2°pAb donkey anti-rat IgG labeled with B3 initiator, amplifier B3-Alexa546.

and 2 target RNAs:

- Target mRNA *Prkcd*, probe set with 31 split-initiator probe pairs, amplifier B1-Alexa647.
- Target mRNA *Slc17a7*, probe set with 36 split-initiator probe pairs, amplifier B2-Alexa750.

Additional studies are presented as follows:

- Figure S47 compares protein imaging using HCR 2°IHC with and without HCR RNA-ISH for 2 target proteins.
- Figure S48 compares RNA imaging using RNA-ISH with and without HCR 2°IHC for 2 target RNAs.
- Table S34 displays estimated values for signal, background, and signal-to-background for each target.

Protocol: HCR 2°IHC only, or simultaneous HCR 2°IHC + HCR RNA-ISH, or HCR RNA-ISH only (Section S4.2; without the optional autofluorescence bleaching protocol of Section S4.2.3) using initiator-labeled primary antibody probes for protein targets, split-initiator DNA probes for RNA targets, and simultaneous HCR signal amplification for all targets.
Sample: FFPE C57BL/6 mouse brain section (coronal); thickness: 5 μm.
Microscopy: Epifluorescence.

Development • Supplementary information

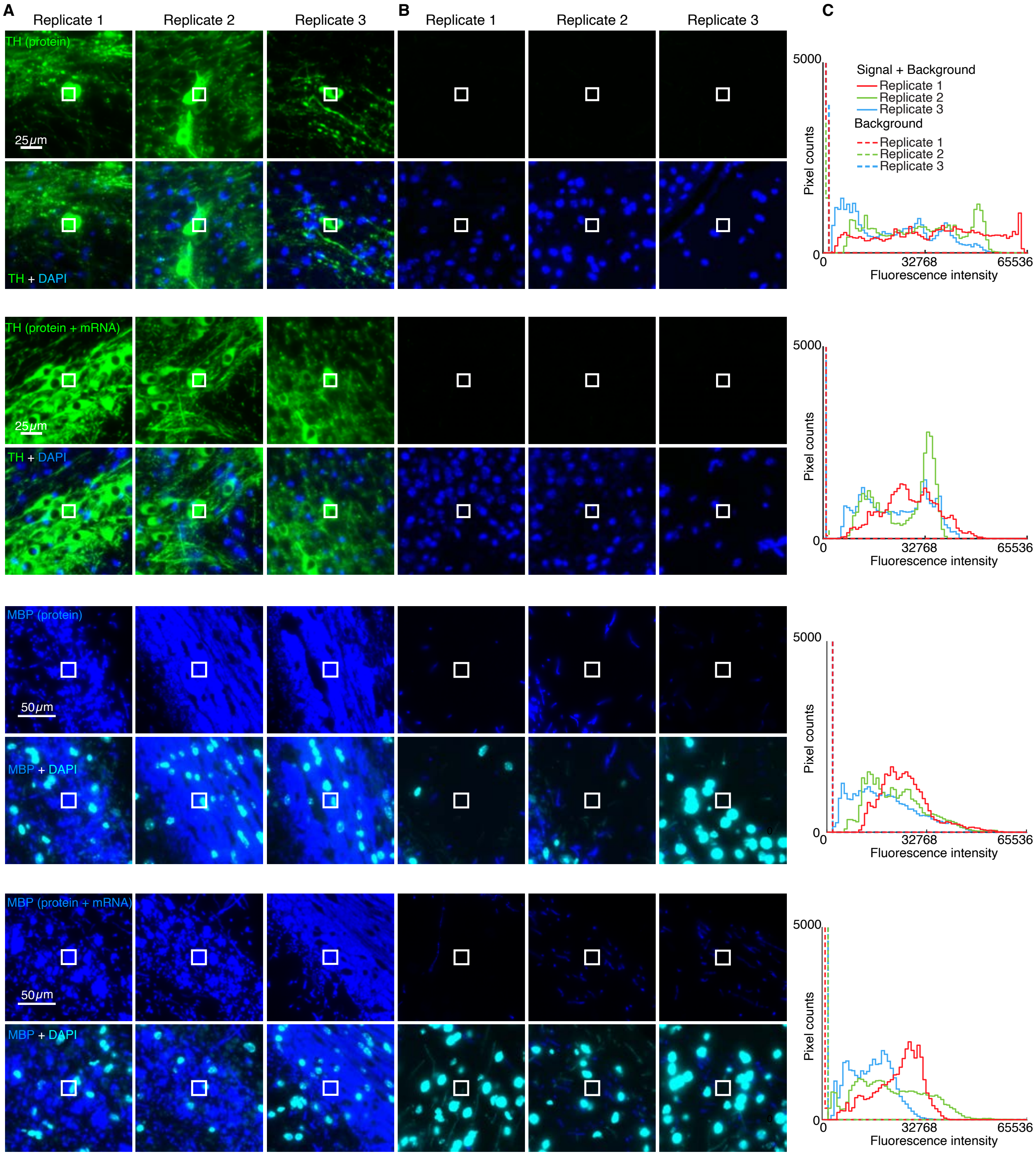

Figure S47. Measurement of signal and background for target proteins using HCR 2°IHC with or without HCR RNA-ISH in FFPE mouse brain sections. Use experiment of Type 1 in Table S8A (1°Ab probe + 2°Ab probe + hairpins) to measure: (A) SIG+BACK+NOISE in a region of high expression, (B) BACK+NOISE in a region of no/low expression, and NOISE in a region with no sample (not depicted). (C) Pixel intensity histograms for representative regions (three rectangles per experiment type for each of 3 replicate mouse brain sections). For each of 2 target proteins (TH or MBP), data is presented using HCR 2°IHC only or HCR 2°IHC + HCR RNA-ISH. Epifluorescence images collected with the microscope exposure time optimized to avoid saturating SIG+BACK pixels for HCR 2°IHC; DAPI channel facilitates placement of representative rectangles. Target proteins: TH (Alexa488) and MBP (Alexa546). Sample: FFPE C57BL/6 mouse brain section (coronal); thickness: 5 μm.

Development • Supplementary information

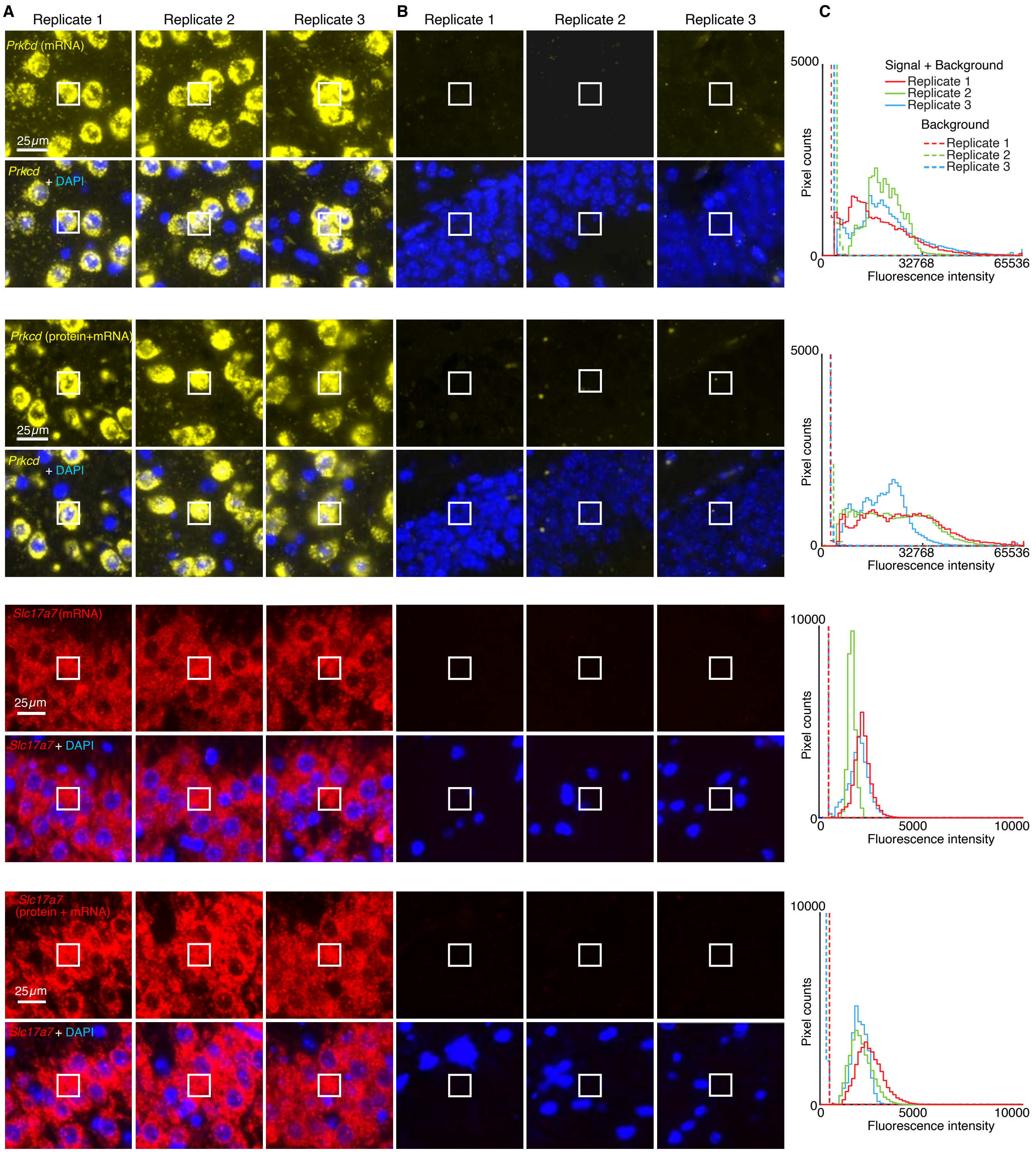

Figure S48. Measurement of signal and background for target RNAs using HCR RNA-ISH with or without HCR 2°IHC in FFPE mouse brain sections. Use experiment of Type 1 in Table S8A (probe set + hairpins) to measure: (A) SIG+BACK+NOISE in a region of high expression, (B) BACK+NOISE in a region of no/low expression, and NOISE in a region with no sample (not depicted). (C) Pixel intensity histograms for representative regions (three rectangles per experiment type for each of 3 replicate mouse brain sections). For each of 2 target RNAs (*Prkcd* or *Slc17a7*), data is presented using HCR RNA-ISH only or HCR 2°IHC + HCR RNA-ISH. Epifluorescence images collected with the microscope exposure time optimized to avoid saturating SIG+BACK pixels for HCR RNA-ISH; DAPI channel facilitates placement of representative rectangles. Target mRNAs: *Prkcd* (Alexa647) and *Slc17a7* (Alexa750). Sample: FFPE C57BL/6 mouse brain section (coronal); thickness: 5 μm.

Development • Supplementary information

Target	Type	Method	Fluorophore	NOISE	BACK+NOISE	SIG+BACK+NOISE	SIG	BACK	SIG/BACK	Experiment
TH	protein	HCR 2°IHC	Alexa488	180 ± 30	445 ± 7	$11\,000 \pm 4000$	$10\,000 \pm 4000$	270 ± 30	38 ± 14	1
TH	protein	HCR 2°IHC + HCR RNA-ISH	Alexa488	160 ± 40	390 ± 30	$16\,000 \pm 3000$	$15\,000 \pm 3000$	234 ± 5	65 ± 17	3
MBP	protein	HCR 2°IHC	Alexa546	330 ± 80	900 ± 300	$14\,000 \pm 4000$	$13\,000 \pm 4000$	500 ± 300	25 ± 18	1
MBP	protein	HCR 2°IHC + HCR RNA-ISH	Alexa546	220 ± 120	490 ± 40	$11\,000 \pm 3000$	$11\,000 \pm 3000$	270 ± 80	40 ± 16	3
Prkcd	mRNA	HCR RNA-ISH	Alexa647	1400 ± 500	2600 ± 800	$19\,800 \pm 1800$	$17\,200 \pm 1900$	1000 ± 1000	15 ± 12	2
Prkcd	mRNA	HCR 2°IHC + HCR RNA-ISH	Alexa647	770 ± 120	1700 ± 300	$23\,000 \pm 3000$	$21\,000 \pm 3000$	1000 ± 300	22 ± 8	3
Slc17a7	mRNA	HCR RNA-ISH	Alexa750	250 ± 14	296 ± 15	1790 ± 180	1500 ± 180	50 ± 20	33 ± 15	2
Slc17a7	mRNA	HCR 2°IHC + HCR RNA-ISH	Alexa750	195 ± 8	229 ± 18	2100 ± 300	1800 ± 300	30 ± 20	50 ± 30	3

Table S34. Estimated signal, background, and signal-to-background for protein imaging using HCR 2°IHC, RNA imaging using HCR RNA-ISH, or both in FFPE mouse brain sections (cf. Figure 6CD). Mean ± standard error of the mean, $N = 3$ replicate FFPE mouse brain sections. Analysis based on representative rectangular regions (examples depicted in Figures S47–S48) using methods of Section S2.6.2. Experiment number designates which target proteins and RNAs were imaged together.

Development • Supplementary information

References

Ali, S., Signor, S. A., Kozlov, K., & Nuzhdin, S. V. (2019). Novel approach to quantitative spatial gene expression uncovers genetic stochasticity in the developing *Drosophila* eye. *Evol. Dev.*, **21**(3), 157–171.

Alon, S., Goodwin, D. R., Sinha, A., Wassie, A. T., Chen, F., Daugharthy, E. R., Bando, Y., Kajita, A., Xue, A. G., Marrett, K., Prior, R., Cui, Yi, Payne, A. C., Yao, C.-C., Suk, H. J., Wang, R., Yu, C. C., Tillberg, P., Reginato, P., Pak, N., Liu, S., Punthambaker, S., Iyer, E. P. R., Kohman, R. E., Miller, J. A., Lein, E. S., Lako, A., Cullen, N., Rodig, S., Helvie, K., Abravanel, D. L., Wagle, N., Johnson, B. E., Klughammer, J., Slyper, M., Waldman, J., Jané-Valbuena, J., Rozenblatt-Rosen, O., Regev, A., IMAXT-Consortium, Church, G. M., Marblestone, A. H., & Boyden, E. S. (2021). Expansion sequencing: Spatially precise in situ transcriptomics in intact biological systems. *Science*, **371**(6528).

Andalman, A. S., Burns, V. M., Lovett-Barron, M., Broxton, M., Poole, B., Yang, S. J., Grosenick, L., Lerner, T. N., Chen, R., Benster, T., Mourrain, P., Levoy, M., Rajan, K., & Deisseroth, K. (2019). Neuronal dynamics regulating brain and behavioral state transitions. *Cell*, **177**(4), 970–985.e20.

Anderson, M. J., Magidson, V., Kageyama, R., & Lewandoski, M. (2020). Fgf4 maintains Hes7 levels critical for normal somite segmentation clock function. *eLife*, **9**(Nov.), e55608.

Anderson, S. R., Roberts, J. R., Zhang, J., Steele, M. R., Romero, C. O., Bosco, A., & Vetter, M. L. (2019). Developmental apoptosis promotes a disease-related gene signature and independence from CSF1R signaling in retinal microglia. *Cell Rep.*, **27**(7), 2002–2013.e5.

Arshadi, C., Günther, U., Eddison, M., Harrington, K. I. S., & Ferreira, T. A. (2021). SNT: A unifying toolbox for quantification of neuronal anatomy. *Nat. Methods*, **18**(4), 374–377.

Askary, A., Sanchez-Guardado, L., Linton, J. M., Chadly, D. M., Budde, M. W., Cai, L., Lois, C., & Elowitz, M. B. (2020). In situ readout of DNA barcodes and single base edits facilitated by in vitro transcription. *Nat. Biotechnol.*, **38**(1), 66–75.

Aztekin, C., Hiscock, T. W., Gurdon, J., Jullien, J., Marioni, J., & Simons, B. D. (2021). Secreted inhibitors drive the loss of regeneration competence in *Xenopus* limbs. *Development*, dev.199158.

Baxter, B. D., Larson, E. D., Merle, L., Feinstein, P., Polese, A. G., Bubak, A. N., Niemeyer, C. S., Hassell, J., Shepherd, D., Ramakrishnan, V. R., Nagel, M. A., & Restrepo, D. (2021). Transcriptional profiling reveals potential involvement of microvillous TRPM5-expressing cells in viral infection of the olfactory epithelium. *BMC Genomics*, **22**(1), 224.

Benkafadar, N., Janesick, A., Scheibinger, M., Ling, A. H., Jan, T. A., & Heller, S. (2021). Transcriptomic characterization of dying hair cells in the avian cochlea. *Cell Rep.*, **34**(12), 108902.

Bennett, B. D., Essock-Burns, T., & Ruby, E. G. (2020). HbtR, a heterofunctional homolog of the virulence regulator TcpP, facilitates the transition between symbiotic and planktonic lifestyles in *Vibrio fischeri*. *mBio*, **11**(5).

Bruce, H. S., & Patel, N. H. (2020). Knockout of crustacean leg patterning genes suggests that insect wings and body walls evolved from ancient leg segments. *Nat Ecol Evol*, **4**(12), 1703–1712.

Bruce, H. S., Jerz, G., Kelly, S., McCarth, J., Pomerantz, A., Senevirathne, G., Sherrard, A., Sun, D. A., Wolff, C., & Patel, N. H. (2021). Hybridization chain reaction (HCR) in situ protocol. *protocols.io*, 10.17504/protocols.io.bunznvf6.

Callahan, R. A., Roberts, R., Sengupta, M., Kimura, Y., Higashijima, S., & Bagnall, M. W. (2019). Spinal V2b neurons reveal a role for ipsilateral inhibition in speed control. *eLife*, **8**, e47837.

Carriere, C. H., Wang, W. X., Sing, A. D., Fekete, A., Jones, Brian E., Yee, Y., Ellegood, J., Maganti, H., Awofala, L., Marocha, J., Aziz, A., Wang, L. Y., Lerch, J. P., & Lefebvre, J. L. (2020). The γ-protocadherins regulate the survival of GABAergic Interneurons during developmental cell death. *J. Neurosci.*, **40**(45), 8652–8668.

Cayuso, J., Xu, Q., Addison, M., & Wilkinson, D. G. (2019). Actomyosin regulation by Eph receptor signaling couples boundary cell formation to border sharpness. *eLife*, **8**, e49696.

Chen, F., Wassie, A. T., Cote, A. J., Sinha, A., Alon, S., Asano, S., Daugharthy, E. R., Chang, J.-B., Marblestone, A., Church, G. M., Raj, A., & Boyden, E. S. (2016). Nanoscale imaging of RNA with expansion

microscopy. *Nat. Methods*, **13**(8), 679–684.

Chen, R., Gore, F., Nguyen, Q., Ramakrishnan, C., Patel, S., Kim, S. H., Raffiee, M., Kim, Y. S., Hsueh, B., Krook-Magnusson, E., Soltesz, I., & Deisseroth, K. (2021). Deep brain optogenetics without intracranial surgery. *Nat. Biotechnol.*, **39**(2), 161–164.

Choi, H. M. T., Chang, J. Y., Trinh, L. A., Padilla, J. E., Fraser, S. E., & Pierce, N. A. (2010). Programmable in situ amplification for multiplexed imaging of mRNA expression. *Nat. Biotechnol.*, **28**(11), 1208–12.

Choi, H. M. T., Beck, V. A., & Pierce, N. A. (2014). Next-generation in situ hybridization chain reaction: Higher gain, lower cost, greater durability. *ACS Nano*, **8**(5), 4284–4294.

Choi, H. M. T., Calvert, C. R., Husain, N., Huss, D., Barsi, J. C., Deverman, B. E., Hunter, R. C., Kato, M., Lee, S. M., Abelin, A. C. T., Rosenthal, A. Z., Akbari, O. S., Li, Y., Hay, B. A., Sternberg, P. W., Patterson, P. H., Davidson, E. H., Mazmanian, S. K., Prober, D. A., van de Rijn, M., Leadbetter, J. R., Newman, D. K., Readhead, C., Bronner, M. E., Wold, B., Lansford, R., Sauka-Spengler, T., Fraser, S. E., & Pierce, N. A. (2016). Mapping a multiplexed zoo of mRNA expression. *Development*, **143**, 3632–3637.

Choi, H. M. T., Schwarzkopf, M., Fornace, M. E., Acharya, A., Artavanis, G., Stegmaier, J., Cunha, A., & Pierce, N. A. (2018). Third-generation in situ hybridization chain reaction: Multiplexed, quantitative, sensitive, versatile, robust. *Development*, **145**, dev165753.

Cleary, B., Simonton, B., Bezney, J., Murray, E., Alam, S., Sinha, A., Habibi, E., Marshall, J., Lander, E. S., Chen, F., & Regev, A. (2021). Compressed sensing for highly efficient imaging transcriptomics. *Nat. Biotechnol.*, 1–7.

Crabtree, J. R., Macagno, A. L. M., Moczek, A. P., Rohner, P. T., & Hu, Y. (2020). Notch signaling patterns head horn shape in the bull-headed dung beetle *Onthophagus taurus*. *Dev. Genes Evol.*, **230**(3), 213–225.

Criswell, K. E., & Gillis, J. A. (2020). Resegmentation is an ancestral feature of the gnathostome vertebral skeleton. *eLife*, **9**, e51696.

Dar, D., Thomashow, L. S., Weller, D. M., & Newman, D. K. (2020). Global landscape of phenazine biosynthesis and biodegradation reveals species-specific colonization patterns in agricultural soils and crop microbiomes. *eLife*, **9**, e59726.

DePas, W. H., Starwalt-Lee, R., Sambeek, L. V., Kumar, S. R., Gradinaru, V., & Newman, D. K. (2016). Exposing the three-dimensional biogeography and metabolic states of pathogens in cystic fibrosis sputum via hydrogel embedding, clearing, and rRNA labeling. *mBio*, **7**(5), e00796–16.

Deryckere, A., Styfhals, R., Elagoz, A. M., Maes, G. E., & Seuntjens, E. (2021). Identification of neural progenitor cells and their progeny reveals long distance migration in the developing octopus brain. *eLife*, **10**(Aug.), e69161.

Diaz, G. H., & Heller, S. (2021). Fluorescent in situ mRNA detection in the adult mouse cochlea. *STAR Protocols*, **2**(3), 100711.

Diaz Soria, C. L., Lee, J., Chong, T., Coghlan, A., Tracey, A., Young, M. D., Andrews, T., Hall, C., Ng, B. L., Rawlinson, Kate, Doyle, S. R., Leonard, S., Lu, Z., Bennett, H. M., Rinaldi, G., Newmark, P. A., & Berriman, M. (2020). Single-cell atlas of the first intra-mammalian developmental stage of the human parasite *Schistosoma mansoni*. *Nat. Commun.*, **11**(1), 6411.

Domsch, K., Schröder, J., Janeschik, M., Schaub, C., & Lohmann, I. (2021). The Hox transcription factor Ubx ensures somatic myogenesis by suppressing the mesodermal master regulator Twist. *Cell Rep.*, **34**(1), 108577.

Emert, B. L., Cote, C. J., Torre, E. A., Dardani, I. P., Jiang, C. L., Jain, N., Shaffer, S. M., & Raj, A. (2021). Variability within rare cell states enables multiple paths toward drug resistance. *Nat. Biotechnol.*, 1–12.

Freitas, P. D., Lovely, A. M., & Monaghan, J. R. (2019). Investigating Nrg1 signaling in the regenerating axolotl spinal cord using multiplexed FISH. *Dev. Neurobiol.*, **79**(5), 453–467.

Gainett, G., González, V. L., Ballesteros, J. A., Setton, E. V. W., Baker, C. M., Barolo Gargiulo, L., Santibáñez-López, C. E., Coddington, J. A., & Sharma, P. P. (2021). The genome of a daddy-long-legs (Opiliones) illuminates the evolution of arachnid appendages. *Proc. R. Soc. B Biol. Sci.*, **288**(1956), 20211168.

Gallagher, T. L., Tietz, K. T., Morrow, Z. T., McCammon, J. M., Goldrich, M. L., Derr, N. L., & Amacher, S. L. (2017). Pnrc2 regulates 3'UTR-mediated decay of segmentation clock-associated transcripts during zebrafish segmentation. *Dev. Biol.*, **429**(1), 225–239.

Development • Supplementary information

Gallego-Hernandez, A. L., DePas, W. H., Park, J. H., Teschler, J. K., Hartmann, R., Jeckel, H., Drescher, K., Beyhan, S., Newman, D. K., & Yildiz, F. H. (2020). Upregulation of virulence genes promotes *Vibrio cholerae* biofilm hyperinfectivity. *Proc. Natl. Acad. Sci.*, **117**(20), 11010–11017.

Gandhi, S., Hutchins, E. J., Maruszko, K., Park, J. H., Thomson, M., & Bronner, M. E. (2020). Bimodal function of chromatin remodeler Hmga1 in neural crest induction and Wnt-dependent emigration. *eLife*, **9**, e57779.

Gandhi, S., Li, Y., Tang, W., Christensen, J. B., Urrutia, H. A., Vieceli, F. M., Piacentino, M. L., & Bronner, M. E. (2021). A single-plasmid approach for genome editing coupled with long-term lineage analysis in chick embryos. *Development*, **148**(dev193565).

Gasperini, M., Hill, A. J., McFaline-Figueroa, J. L., Martin, B., Kim, S., Zhang, M. D., Jackson, D., Leith, A., Schreiber, J., Noble, W. S., Trapnell, C., Ahituv, N., & Shendure, J. (2019). A genome-wide framework for mapping gene regulation via cellular genetic screens. *Cell*, **176**(1), 377–390.e19.

Glineburg, M. R., Zhang, Y., Krans, A., Tank, E. M., Barmada, S. J., & Todd, P. K. (2021). Enhanced detection of expanded repeat mRNA foci with hybridization chain reaction. *Acta Neuropathol. Commun.*, **9**(1), 73.

Goffredi, S. K., Motooka, C., Fike, D. A., Gusmão, L. C., Tilic, E., Rouse, G. W., & Rodríguez, E. (2021). Mixotrophic chemosynthesis in a deep-sea anemone from hydrothermal vents in the Pescadero Basin, Gulf of California. *BMC Biol.*, **19**(1), 8.

Grancharova, T., Gerbin, K. A., Rosenberg, A. B., Roco, C. M., Arakaki, Joy E., DeLizo, C. M., Dinh, S. Q., Donovan-Maiye, R. M., Hirano, M., Nelson, A. M., Tang, J., Theriot, J. A., Yan, C., Menon, V., Palecek, S. P., Seelig, G., & Gunawardane, R. N. (2021). A comprehensive analysis of gene expression changes in a high replicate and open-source dataset of differentiating hiPSC-derived cardiomyocytes. *Sci Rep*, **11**(1), 15845.

Herrera-Úbeda, C., Marín-Barba, M., Navas-Pérez, E., Gravemeyer, J., Albuixech-Crespo, B., Wheeler, G. N., & Garcia-Fernàndez, J. (2019). Microsyntenic clusters reveal conservation of lncRNAs in chordates despite absence of sequence conservation. *Biology*, **8**(3), 61.

Hinzke, T., Kleiner, M., Meister, M., Schlüter, R., Hentschker, C., Pané-Farré, J., Hildebrandt, P., Felbeck, H., Sievert, S. M., Bonn, F., Völker, U., Becher, D., Schweder, T., & Markert, S. (2021). Bacterial symbiont subpopulations have different roles in a deep-sea symbiosis. *eLife*, **10**, e58371.

Hockman, D., Chong-Morrison, V., Green, S. A., Gavriouchkina, D., Candido-Ferreira, I., Ling, I. T. C., Williams, R. M., Amemiya, C. T., Smith, J. J., Bronner, M. E., & Sauka-Spengler, T. (2019). A genome-wide assessment of the ancestral neural crest gene regulatory network. *Nat. Commun.*, **10**(1), 4689.

Howard, IV, A. G. A, Baker, P. A., Ibarra-García-Padilla, R., Moore, J. A., Rivas, L. J., Tallman, J. J., Singleton, E. W., Westheimer, J. L., Corteguera, J. A., & Uribe, R. A. (2021). An atlas of neural crest lineages along the posterior developing zebrafish at single-cell resolution. *eLife*, **10**, e60005.

Hu, Y., Linz, D. M., & Moczek, A. P. (2019). Beetle horns evolved from wing serial homologs. *Science*, **366**(6468), 1004–1007.

Huss, D., Choi, H. M. T., Readhead, C., Fraser, S. E., Pierce, N. A., & Lansford, R. (2015). Combinatorial analysis of mRNA expression patterns in mouse embryos using hybridization chain reaction. *Cold Spring Harb. Protoc.*, **3**, 259–268.

Huss, D. J., Saias, S., Hamamah, S., Singh, J. M., Wang, J., Dave, M., Kim, J., Eberwine, J., & Lansford, R. (2019). Avian primordial germ cells contribute to and interact with the extracellular matrix during early migration. *Front. Cell Dev. Biol.*, **7**.

Janesick, A., Scheibinger, M., Benkafadar, N., Kirti, S., Ellwanger, D. C., & Heller, S. (2021). Cell-type identity of the avian cochlea. *Cell Rep.*, **34**(12), 108900.

Jensen, T. B., Giunta, P., Schultz, N. G., Griffiths, J. M., Duerr, T. J., Kyeremateng, Y., Wong, H., Adesina, A., & Monaghan, J. R. (2021). Lung injury in axolotl salamanders induces an organ-wide proliferation response. *Dev. Dyn.*, **250**(6), 866–879.

Jimenez, E., Slevin, C. C., Colón-Cruz, L., & Burgess, S. M. (2021). Vestibular and auditory hair cell regeneration following targeted ablation of hair cells with diphtheria toxin in zebrafish. *Front. Cell. Neurosci.*, **15**, 333.

Jorth, P., Spero, M. A., Livingston, J., & Newman, D. K. (2019). Quantitative visualization of gene expression in mucoid and nonmucoid *Pseudomonas aeruginosa* aggregates reveals localized peak expression of alginate in

the hypoxic zone. *mBio*, **10**(6).

Kahan, A., Greenbaum, A., Jang, M. J., Robinson, J. E., Cho, J. R., Chen, X., Kassraian, P., Wagenaar, D. A., & Gradinaru, V. (2021). Light-guided sectioning for precise in situ localization and tissue interface analysis for brain-implanted optical fibers and GRIN lenses. *Cell Reports*, **36**(13), 109744.

Kamermans, A., Verhoeven, T., van het Hof, B., Koning, J. J., Borghuis, L., Witte, M., van Horssen, J., de Vries, H. E., & Rijnsburger, M. (2019). Setmelanotide, a novel, selective melanocortin receptor-4 agonist exerts anti-inflammatory actions in astrocytes and promotes an anti-inflammatory macrophage phenotype. *Front. Immunol.*, **10**.

Kinney, B. A., Anber, B. A., Row, R. H., Tseng, Y. J., Weidmann, M. D., Knaut, H., & Martin, B. L. (2020). Sox2 and canonical Wnt signaling interact to activate a developmental checkpoint coordinating morphogenesis with mesoderm fate acquisition. *Cell Rep.*, **33**(4).

Kourakis, M. J., Borba, C., Zhang, A., Newman-Smith, E., Salas, P., Manjunath, B., & Smith, W. C. (2019). Parallel visual circuitry in a basal chordate. *eLife*, **8**, e44753.

Kramer, E. E., Steadman, P. E., Epp, J. R., Frankland, P. W., & Josselyn, S. A. (2018). Assessing individual neuronal activity across the intact brain: Using hybridization chain reaction (HCR) to detect Arc mRNA localized to the nucleus in volumes of cleared brain tissue. *Curr. Protoc. Neurosci.*, **84**(1), e49.

Krienen, F. M., Goldman, M., Zhang, Q., C. H. del Rosario, R., Florio, M., Machold, R., Saunders, A., Levandowski, K., Zaniewski, H., Schuman, B., Wu, C., Lutservitz, A., Mullally, C. D., Reed, N., Bien, E., Bortolin, L., Fernandez-Otero, M., Lin, J. D., Wysoker, A., Nemesh, J., Kulp, D., Burns, M., Tkachev, V., Smith, R., Walsh, C. A., Dimidschstein, J., Rudy, B., S. Kean, L., Berretta, S., Fishell, G., Feng, G., & McCarroll, S. A. (2020). Innovations present in the primate interneuron repertoire. *Nature*, **586**(7828), 262–269.

Kumar, V., Krolewski, D. M., Hebda-Bauer, E. K., Parsegian, A., Martin, B., Foltz, M., Akil, H., & Watson, S. J. (2021). Optimization and evaluation of fluorescence in situ hybridization chain reaction in cleared fresh-frozen brain tissues. *Brain Struct. Funct.*, **226**(2), 481–499.

Lacin, H., Chen, H. M., Long, X., Singer, R. H., Lee, T., & Truman, J. W. (2019). Neurotransmitter identity is acquired in a lineage-restricted manner in the Drosophila CNS. *eLife*, **8**, e43701.

Li, Y., He, X., Kawaguchi, R., Zhang, Y., Wang, Q., Monavarfeshani, A., Yang, Z., Chen, B., Shi, Z., Meng, H., Zhou, S., Zhu, J., Jacobi, A., Swarup, V., Popovich, P. G., Geschwind, D. H., & He, Z. (2020). Microglia-organized scar-free spinal cord repair in neonatal mice. *Nature*, **587**(7835), 613–618.

Lignell, A., Kerosuo, L., Streichan, S. J., Cai, L., & Bronner, M. E. (2017). Identification of a neural crest stem cell niche by spatial genomic analysis. *Nat. Commun.*, **8**(1), 1830.

Ling, I. T. C., & Sauka-Spengler, T. (2019). Early chromatin shaping predetermines multipotent vagal neural crest into neural, neuronal and mesenchymal lineages. *Nat. Cell Biol.*, **21**(12), 1504–1517.

Liu, Y., Zou, R. S., He, S., Nihongaki, Y., Li, X., Razavi, S., Wu, B., & Ha, T. (2020). Very fast CRISPR on demand. *Science*, **368**(6496), 1265–1269.

Lovett-Barron, M., Chen, R., Bradbury, S., Andalman, A. S., Wagle, M., Guo, S., & Deisseroth, K. (2020). Multiple convergent hypothalamus–brainstem circuits drive defensive behavior. *Nat. Neurosci.*, **23**(8), 959–967.

Mantri, M., Scuderi, G. J., Abedini-Nassab, R., Wang, M. F. Z., McKellar, D., Shi, H., Grodner, B., Butcher, J. T., & De Vlaminck, I. (2021). Spatiotemporal single-cell RNA sequencing of developing chicken hearts identifies interplay between cellular differentiation and morphogenesis. *Nat. Commun.*, **12**(1), 1771.

Marconi, A., Hancock-Ronemus, A., & Gillis, J. A. (2020). Adult chondrogenesis and spontaneous cartilage repair in the skate, Leucoraja erinacea. *eLife*, **9**(May), e53414.

May-Zhang, A. A., Tycksen, E., Southard-Smith, A. N., Deal, K. K., Benthal, J. T., Buehler, D. P., Adam, M., Simmons, A. J., Monaghan, J. R., Matlock, B. K., Flaherty, D. K., Potter, S. S., Lau, K. S., & Southard-Smith, E. M. (2021). Combinatorial transcriptional profiling of mouse and human enteric neurons identifies shared and disparate subtypes in situ. *Gastroenterology*, **160**(3), 755–770.e26.

Mayerl, S., Chen, J., Salveridou, E., Boelen, A., Darras, V. M., & Heuer, H. (2021). Thyroid hormone transporter deficiency in mice impacts multiple stages of GABAergic interneuron development. *Cerebral Cortex*, July.

McLennan, R., Schumacher, L. J., Morrison, J. A., Teddy, J. M., Ridenour, D. A., Box, A. C., Semerad, C. L., Li, H., McDowell, W., Kay, D., Maini, P. K., Baker, R. E., & Kulesa, P. M. (2015). Neural crest migration is driven by a few trailblazer cells with a unique molecular signature narrowly confined to the invasive front. *Development*, **142**(11), 2014–2025.

Meinecke, L., Sharma, P. P., Du, H., Zhang, L., Nie, Q., & Schilling, T. F. (2018). Modeling craniofacial development reveals spatiotemporal constraints on robust patterning of the mandibular arch. *PLOS Comput. Biol.*, **14**(11), e1006569.

Metcalfe, K. S., Murali, R., Mullin, S. W., Connon, S. A., & Orphan, V. J. (2021). Experimentally-validated correlation analysis reveals new anaerobic methane oxidation partnerships with consortium-level heterogeneity in diazotrophy. *ISME J.*, **15**(2), 377–396.

Mich, J. K., Graybuck, L. T., Hess, E. E., Mahoney, J. T., Kojima, Y., Ding, Yi, Somasundaram, S., Miller, J. A., Kalmbach, B. E., Radaelli, C., Gore, B. B., Weed, N., Omstead, V., Bishaw, Y., Shapovalova, N. V., Martinez, R. A., Fong, O., Yao, S., Mortrud, M., Chong, P., Loftus, L., Bertagnolli, D., Goldy, J., Casper, T., Dee, N., Opitz-Araya, X., Cetin, A., Smith, K. A., Gwinn, R. P., Cobbs, C., Ko, A. L., Ojemann, J. G., Keene, C. D., Silbergeld, D. L., Sunkin, S. M., Gradinaru, V., Horwitz, G. D., Zeng, H., Tasic, B., Lein, E. S., Ting, J. T., & Levi, B. P. (2021). Functional enhancer elements drive subclass-selective expression from mouse to primate neocortex. *Cell Rep.*, **34**(13), 108754.

Michael, V., Goffinet, J., Pearson, J., Wang, F., Tschida, K., & Mooney, R. (2020). Circuit and synaptic organization of forebrain-to-midbrain pathways that promote and suppress vocalization. *eLife*, **9**, e63493.

Michki, N. S., Li, Y., Sanjasaz, K., Zhao, Y., Shen, F. Y., Walker, L. A., Cao, W., Lee, C.-Y., & Cai, D. (2021). The molecular landscape of neural differentiation in the developing *Drosophila* brain revealed by targeted scRNA-seq and multi-informatic analysis. *Cell Rep.*, **35**(4), 109039.

Milewska, A., Kula-Pacurar, A., Wadas, J., Suder, A., Szczepanski, A., Dabrowska, A., Owczarek, K., Marcello, A., Ochman, M., Stacel, T., Rajfur, Z., Sanak, M., Labaj, P., Branicki, W., & Pyrc, K. (2020). Replication of severe acute respiratory syndrome coronavirus 2 in human respiratory epithelium. *J. Virol.*, **94**(15), e00957–20.

Moriano-Gutierrez, S., Bongrand, C., Essock-Burns, T., Wu, L., McFall-Ngai, Margaret J., & Ruby, E. G. (2020). The noncoding small RNA SsrA is released by *Vibrio fischeri* and modulates critical host responses. *PLOS Biol.*, **18**(11), e3000934.

Mu, W., Li, S., Xu, J., Guo, X., Wu, H., Chen, Z., Qiao, L., Helfer, G., Lu, F., Liu, C., & Wu, Q. F. (2021). Hypothalamic Rax + tanycytes contribute to tissue repair and tumorigenesis upon oncogene activation in mice. *Nat. Commun.*, **12**(1), 2288.

Nandagopal, N., Santat, L. A., & Elowitz, M. B. (2019). Cis-activation in the Notch signaling pathway. *eLife*, **8**, e37880.

Nikolakakis, K., Lehnert, E., McFall-Ngai, M. J., & Ruby, E. G. (2015). Use of hybridization chain reaction-fluorescent in situ hybridization to track gene expression by both partners during initiation of symbiosis. *Appl. Environ. Microbiol.*, **81**(14), 4728–4735.

O'Brown, N. M., Megason, S. G., & Gu, C. (2019). Suppression of transcytosis regulates zebrafish blood-brain barrier function. *eLife*, **8**, e47326.

Park, Y.-G., Sohn, C. H., Chen, R., McCue, M., Yun, D. H., Drummond, G. T., Ku, T., Evans, N. B., Oak, H. C., Trieu, W., Choi, H., Jin, X., Lilascharoen, V., Wang, J., Truttmann, M. C., Qi, H. W., Ploegh, H. L., Golub, T. R., Chen, S.-C., Frosch, M. P., Kulik, H. J., Lim, B. K., & Chung, K. (2018). Protection of tissue physicochemical properties using polyfunctional crosslinkers. *Nat. Biotechnol.*, **37**(1), 73–83.

Patriarchi, T., Cho, J. R., Merten, K., Howe, M. W., Marley, A., Xiong, W. H., Folk, R. W., Broussard, G. J., Liang, R., Jang, M. J., Zhong, H., Dombeck, D., Zastrow, M. V., Nimmerjahn, A., Gradinaru, V., Williams, J. T., & Tian, L. (2018). Ultrafast neuronal imaging of dopamine dynamics with designed genetically encoded sensors. *Science*, **360**(6396).

Pond, A. J. R., Hwang, S., Verd, B., & Steventon, B. (2021). A deep learning approach for staging embryonic tissue isolates with small data. *PLOS ONE*, **16**(1), e0244151.

Ren, J., Isakova, A., Friedmann, D., Zeng, J., Grutzner, S. M., Pun, A., Zhao, G. Q., Kolluru, S. S., Wang,

Development • Supplementary information

R., Lin, R., Li, P., Li, A., Raymond, J. L., Luo, Q., Luo, M., Quake, S. R., & Luo, L. (2019). Single-cell transcriptomes and whole-brain projections of serotonin neurons in the mouse dorsal and median raphe nuclei. *eLife*, **8**, e49424.

Rodriguez, C. M., Wright, S. E., Kearse, M. G., Haenfler, J. M., Flores, B. N., Liu, Y., Ifrim, M. F., Glineburg, M. R., Krans, A., Jafar-Nejad, P., Sutton, M. A., Bassell, G. J., Parent, J. M., Rigo, F., Barmada, S. J., & Todd, P. K. (2020). A native function for RAN translation and CGG repeats in regulating fragile X protein synthesis. *Nat. Neurosci.*, **23**(3), 386–397.

Rosenthal, A. Z., Zhang, X. N., Lucey, K. S., Ottesen, E. A., Trivedi, V., Choi, H. M. T., Pierce, N. A., & Leadbetter, J. R. (2013). Localizing transcripts to single cells suggests an important role of uncultured deltaproteobacteria in the termite gut hydrogen economy. *Proc. Natl. Acad. Sci. U. S. A.*, **110**(40), 16163–16168.

Schloissnig, S., Kawaguchi, A., Nowoshilow, S., Falcon, F., Otsuki, L., Tardivo, P., Timoshevskaya, N., Keinath, M. C., Smith, J. J., Voss, S. R., & Tanaka, E. M. (2021). The giant axolotl genome uncovers the evolution, scaling, and transcriptional control of complex gene loci. *Proc. Natl. Acad. Sci.*, **118**(15).

Shah, S., Lubeck, E., Zhou, W., & Cai, L. (2016a). In situ transcription profiling of single cells reveals spatial organization of cells in the mouse hippocampus. *Neuron*, **92**, 342–357.

Shah, S., Lubeck, E., Schwarzkopf, M., He, T.-F., Greenbaum, A., Sohn, C. H., Lignell, A., Choi, H. M. T., Gradinaru, V., Pierce, N. A., & Cai, L. (2016b). Single-molecule RNA detection at depth via hybridization chain reaction and tissue hydrogel embedding and clearing. *Development*, **143**, 2862–2867.

Simões, F. C., Cahill, T. J., Kenyon, A., Gavriouchkina, D., Vieira, J. M., Sun, X., Pezzolla, D., Ravaud, C., Masmanian, E., Weinberger, M., Mayes, S., Lemieux, M. E., Barnette, D. N., Gunadasa-Rohling, M., Williams, R. M., Greaves, D. R., Trinh, L. A., Fraser, S. E., Dallas, S. L., Choudhury, R. P., Sauka-Spengler, T., & Riley, P. R. (2020). Macrophages directly contribute collagen to scar formation during zebrafish heart regeneration and mouse heart repair. *Nat. Commun.*, **11**(1), 600.

Sui, Q., Zhu, J., Li, X., Knight, G. E., He, C., Burnstock, G., Yuan, H., & Xiang, Z. (2016). A modified protocol for the detection of three different mRNAs with a new-generation in situ hybridization chain reaction on frozen sections. *J. Mol. Histol.*, **47**(6), 511–529.

Sylwestrak, E. L., Rajasethupathy, P., Wright, M. A., Jaffe, A., & Deisseroth, K. (2016). Multiplexed intact-tissue transcriptional analysis at cellular resolution. *Cell*, **164**(4), 792–804.

Thomson, Lewis, Muresan, Leila, & Steventon, Benjamin. (2021). The zebrafish presomitic mesoderm elongates through compaction-extension. *Cells & Development*, Sept., 203748.

Tidswell, Olivia R. A., Benton, Matthew A., & Akam, Michael. (2021). The neuroblast timer gene nubbin exhibits functional redundancy with gap genes to regulate segment identity in Tribolium. *Development*, **148**(16).

Ton, Q. V., Leino, D., Mowery, S. A., Bredemeier, N. O., Lafontant, P. J., Lubert, A., Gurung, S., Farlow, J. L., Foroud, T. M., Broderick, J., & Sumanas, S. (2018). Collagen COL22A1 maintains vascular stability and mutations in COL22A1 are potentially associated with intracranial aneurysms. *Dis. Model. Mech.*, **11**(dmm033654).

Trivedi, V., Choi, H. M. T., Fraser, S. E., & Pierce, N. A. (2018). Multidimensional quantitative analysis of mRNA expression within intact vertebrate embryos. *Development*, **145**, dev156869.

Tsai, T. Y.-C., Sikora, M., Xia, P., Colak-Champollion, T., Knaut, H., Heisenberg, C., & Megason, S. G. (2020). An adhesion code ensures robust pattern formation during tissue morphogenesis. *Science*, **370**(6512), 113–116.

Tu, R., Duan, B., Song, X., Chen, S., Scott, A., Hall, K., Blanck, J., DeGraffenreid, D., Li, H., Perera, A., Haug, J., & Xie, T. (2021). Multiple niche compartments orchestrate stepwise germline stem cell progeny differentiation. *Curr. Biol.*, **31**(4), 827–839.e3.

van den Brink, S. C., Alemany, A., van Batenburg, V., Moris, N., Blotenburg, M., Vivié, J., Baillie-Johnson, P., Nichols, J., Sonnen, K. F., Martinez Arias, A., & van Oudenaarden, A. (2020). Single-cell and spatial transcriptomics reveal somitogenesis in gastruloids. *Nature*, **582**(7812), 405–409.

van Houcke, J., Mariën, V., Zandecki, C., Vanhunsel, S., Moons, L., Ayana, R., Seuntjens, E., & Arckens, L. (2021). Aging impairs the essential contributions of non-glial progenitors to neurorepair in the dorsal telencephalon of the Killifish Nothobranchius furzeri. *Aging Cell*, **n/a**(n/a), e13464.

von Buchholtz, L. J., Lam, R. M., Emrick, J. J., Chesler, A. T., & Ryba, N. J. P. (2020). Assigning transcriptomic

class in the trigeminal ganglion using multiplex in situ hybridization and machine learning. *Pain*, **161**(9), 2212–2224.

Wang, H., Holland, P. W. H., & Takahashi, T. (2019). Gene profiling of head mesoderm in early zebrafish development: Insights into the evolution of cranial mesoderm. *EvoDevo*, **10**(1), 14.

Weinberger, M, Simões, F. C., Patient, R., Sauka-Spengler, T., & Riley, P. R. (2020). Functional heterogeneity within the developing zebrafish epicardium. *Dev. Cell*, **52**(5), 574–590.e6.

Wells, A. I., Grimes, K. A., Kim, K., Branche, E., Bakkenist, C. J., DePas, W. H., Shresta, S., & Coyne, C. B. (2021). Human FcRn expression and Type I Interferon signaling control Echovirus 11 pathogenesis in mice. *PLOS Pathog.*, **17**(1), e1009252.

Williams, R. M., Candido-Ferreira, I., Repapi, E., Gavriouchkina, D., Senanayake, U., Ling, I. T. C., Telenius, J., Taylor, S., Hughes, J., & Sauka-Spengler, T. (2019). Reconstruction of the global neural crest gene regulatory network in vivo. *Dev. Cell*, **51**(2), 255–276.e7.

Wurster, S., Ruiz, O. E., Samms, K. M., Tatara, A. M., Albert, N. D., Kahan, P. H., Nguyen, A. T., Mikos, A. G., Kontoyiannis, D. P., & Eisenhoffer, G. T. (2021). EGF-mediated suppression of cell extrusion during mucosal damage attenuates opportunistic fungal invasion. *Cell Rep.*, **34**(12), 108896.

Yamaguchi, T., Kawakami, S., Hatamoto, M., Imachi, H., Takahashi, M., Araki, N., Yamaguchi, T., & Kubota, K. (2015). In situ DNA-hybridization chain reaction (HCR): A facilitated in situ HCR system for the detection of environmental microorganisms. *Environ. Microbiol.*, **17**(7), 2532–2541.

Young, W. S., & Song, J. (2020). Characterization of oxytocin receptor expression within various neuronal populations of the mouse dorsal hippocampus. *Front. Mol. Neurosci.*, **13**.

Zhuang, P., Zhang, H., Welchko, R. M., Thompson, R. C., Xu, S., & Turner, D. L. (2020). Combined microRNA and mRNA detection in mammalian retinas by in situ hybridization chain reaction. *Sci. Rep.*, **10**(1), 351.
